# Synthesis of C_3_-Substituted *N*1-*tert*-Butyl 1,2,4-Triazinium Salts via the Liebeskind–Srogl Reaction for Fluorogenic Labeling of Live Cells

**DOI:** 10.1021/acs.joc.3c02454

**Published:** 2024-01-15

**Authors:** Veronika Šlachtová, Simona Bellová, Milan Vrabel

**Affiliations:** Institute of Organic Chemistry and Biochemistry of the Czech Academy of Sciences, Flemingovo nám. 2, 16000 Prague, Czech Republic

## Abstract

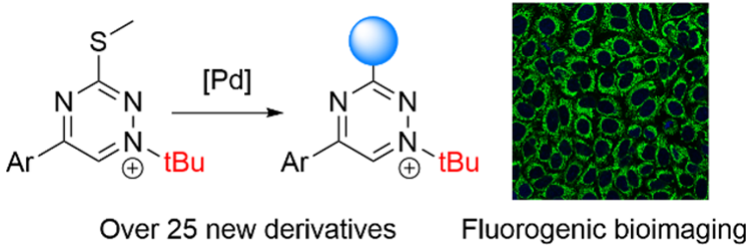

We recently described the development and application of a new bioorthogonal conjugation, the triazinium ligation. To explore the wider application of this reaction, in this work, we introduce a general method for synthesizing C_3_-substituted triazinium salts based on the Liebeskind–Srogl cross-coupling reaction and catalytic thioether reduction. These methods enabled the synthesis of triazinium derivatives for investigating the effect of different substituents on the ligation kinetics and stability of the compounds under biologically relevant conditions. Finally, we demonstrate that the combination of a coumarin fluorophore attached to position C_3_ with a C_5_-(4-methoxyphenyl) substituent yields a fluorogenic triazinium probe suitable for no-wash, live-cell labeling. The developed methodology represents a promising synthetic approach to the late-stage modification of triazinium salts, potentially widening their applications in bioorthogonal reactions.

## Introduction

Chemical reactions compatible with biological systems have applications in chemical biology,^1^ biomedicine,^2^ imaging,^3^ diagnosis,^4−6^ and therapy.^7−11^ Among the many bioorthogonal reactions now available, the inverse electron demand Diels–Alder reaction of 1,2,4,5-tetrazines with strained dienophiles meets the strict criteria for use in living organisms,^12−16^ including humans.^17^ In recent years, researchers have made considerable efforts to fine-tune this bioorthogonal reaction for use in diverse applications.^1,8,18,19^ In addition to other promising synthetic methods,^20,21^ cross-coupling reactions involving tetrazines provide many opportunities for modifying these heterodienes. Successful examples are the Suzuki reaction of 6-*N,N*-dialkyl-substituted 3-chlorotetrazines, which leads to various unsymmetrical tetrazines in high yields,^22^ and Sonogashira cross-coupling of bromotetrazines and various terminal alkynes for the synthesis of alkynyl tetrazines.^23^ Devaraj and colleagues developed a class of 3-substituted 6-mesyloxyethyl-tetrazine building blocks that react with a variety of aryl halides through an in situ elimination–Heck cascade reaction to obtain a series of π-conjugated tetrazine derivatives.^24^ Other valuable building blocks are thiotetrazines, which can react with (hetero)arylboronic acids to form substituted tetrazines via a Ag(I)-mediated Liebeskind–Srogl (L–S) cross-coupling reaction.^25,26^

However, despite these achievements, the demand for more advanced bioorthogonal reactions and reagents remains. To meet this challenge, we recently introduced *N*1-alkyl triazinium salts (**Trz**^**+**^**s**).^27^ These charged heterodienes react rapidly with strained alkynes in a process we call “triazinium ligation”. Due to their high reactivity and excellent stability under biological conditions, the *N*1-*tert-*butylated triazinium salts are particularly valuable derivatives.

**Figure 1:**
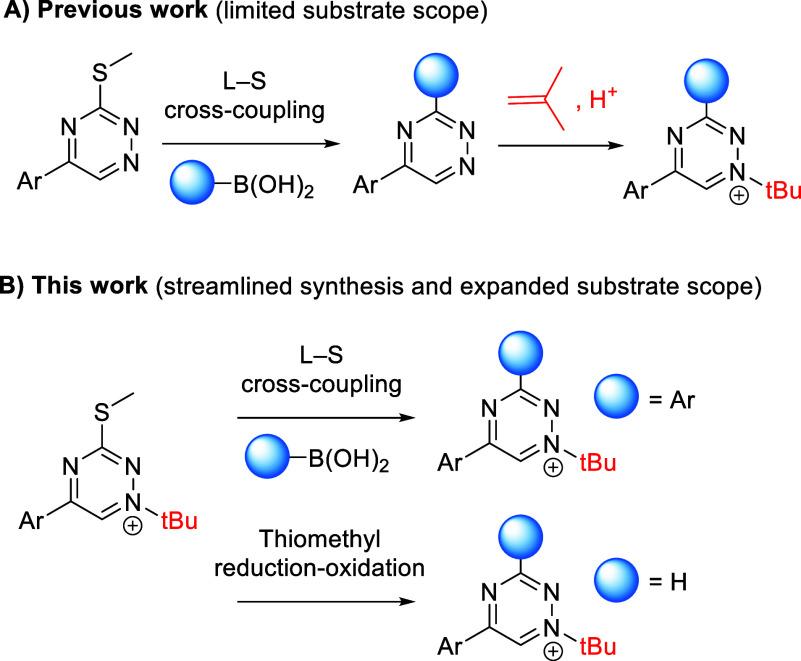
(A) Previously reported synthesis of *N*1-*tert*-butylated triazinium salts. (B) Synthetic strategy for late-stage modification of *N*1-*tert*-butylated triazinium salts at position C_3_.

In this work, we present a synthetic method involving the production of *N*1-*tert*-butylated triazinium salts with various substituents at the C_3_ position (Figure 1), which is achieved by optimizing the L–S cross-coupling reaction and a one-pot thiomethyl reduction–oxidation reaction sequence (Figure 1B). Having access to new derivatives, we examine the effect of different substituents on the reactivity with a strained alkyne dienophile and the stability of these triazinium salts under biologically relevant conditions. We also demonstrate the utility of these heterodienes in the synthesis of coumarin dye conjugates attached to the C_3_ positions of the triazinium core using a conjugated phenylene linker. Suitable for labeling live cells under no-wash conditions, these fluorogenic probes have the potential to be broadly applied in biological research.^28^

## Results and Discussion

In our previous work, we prepared *t*Bu-triazinium salts from 1,2,4-triazines by *tert*-butylation with isobutylene in the presence of triflic acid (Figure 1A).^27^ However, the substrate scope of this method is inherently restricted to compounds that survive acidic conditions. Moreover, overalkylation can pose a problem for compounds containing additional reactive nucleophilic sites. To overcome these limitations, we sought to develop a late-stage modification strategy based on the L–S cross-coupling reaction, which we previously used for the modification of 1,2,4-triazines at position C_3_.^29^ Our initial experiments revealed that *N*1-methylated and *N*1-ethylated triazinium salts were not compatible with the previously applied conditions.^29^ However, the *N*1-*tert*-butyl triazinium was well tolerated, and the desired C_3_-phenyl-substituted triazinium **Trz**^**+**^**2a** formed in 74% yield (Table 1, entry 1). To explore the impact of various parameters on the reaction outcome, we systematically varied the composition of the catalytic system, nucleophiles, ligands, mediators, temperature, and solvents (Table 1 and Table S1). For instance, decreasing the temperature to 60 °C increased the yield to 81% (Table S1). Conversely, changing the catalytic system to Pd(dppf)Cl_2_ or Pd_2_dba_3_ proved to be less successful (Table S1). Dimethylformamide was found to be an inefficient solvent, and switching to tetrahydrofuran did not significantly enhance the reaction (Table S1). Unlike the L–S reactions involving thioalkyl tetrazines,^25^ the use of Ag_2_O failed to promote the reaction with triazinium salts. The same held true for Cu_2_O, which yielded only trace amounts of the product. Employing 2 equiv of phenylboronic acid was sufficient, resulting in a yield of 89% (Table 1, entry 2). Interestingly, employing the same number of equivalents of potassium phenyltrifluoroborate significantly reduced the yield (Table 1, entry 6). The optimal conditions for substituting thiomethyl triazinium at position C_3_ involved conducting the reaction at 60 °C in 1,4-dioxane, utilizing a combination of Pd(PPh_3_)_4_ and CuTC, along with 2 equiv of the boronic acid (Table 1, entry 2).

**Table 1:**
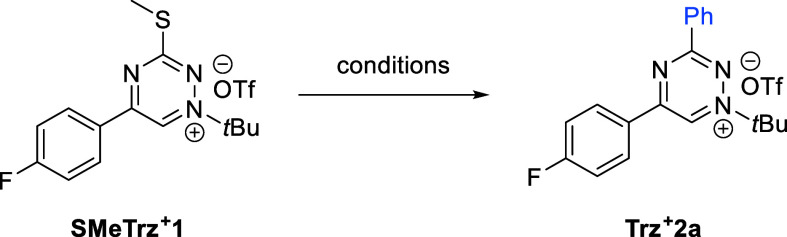
Optimization of the Reaction Conditions for Liebeskind–Srogl Cross-Coupling of **SMeTrz^+^1**^a^

entry/nucleophile (equiv)	ligand	mediator	solvent [*T* (°C)]	yield (%)
**1**/PhB(OH)_2_ (2.5)	Pd(PPh_3_)_4_	CuTC	dioxane (95)	74
**2**/PhB(OH)_2_ (2.0)	Pd(PPh_3_)_4_	CuTC	dioxane (60)	89
**3**/PhB(OH)_2_ (2.0)	Pd(PPh_3_)_4_	Ag_2_O	dioxane (60)	13
**4**/PhB(OH)_2_ (2.0)	Pd_2_(dba)_3_	CuTC	dioxane (60)	53
**5**/PhB(OH)_2_ (2.0)	Pd(PPh_3_)_4_	CuTC	DMF (60)	62
**6**/PhBF_3_^–^K^+^ (2.0)	Pd(PPh_3_)_4_	CuTC	dioxane (60)	28

a Conditions: 0.02 mM **SMeTrz**^**+**^**1**, nucleophile (equiv), ligand (10 mol %), mediator (2.2 equiv), solvent (1.0 mL), *T* (°C), 4 h. Yield calculated from HPLC-MS at 278 nm. Caffeine was used as a standard. CuTC = copper(I) thiophene-2-carboxylate, and dba = dibenzylideneacetone. Extended data can be found in Table S1.

**Scheme 1:**
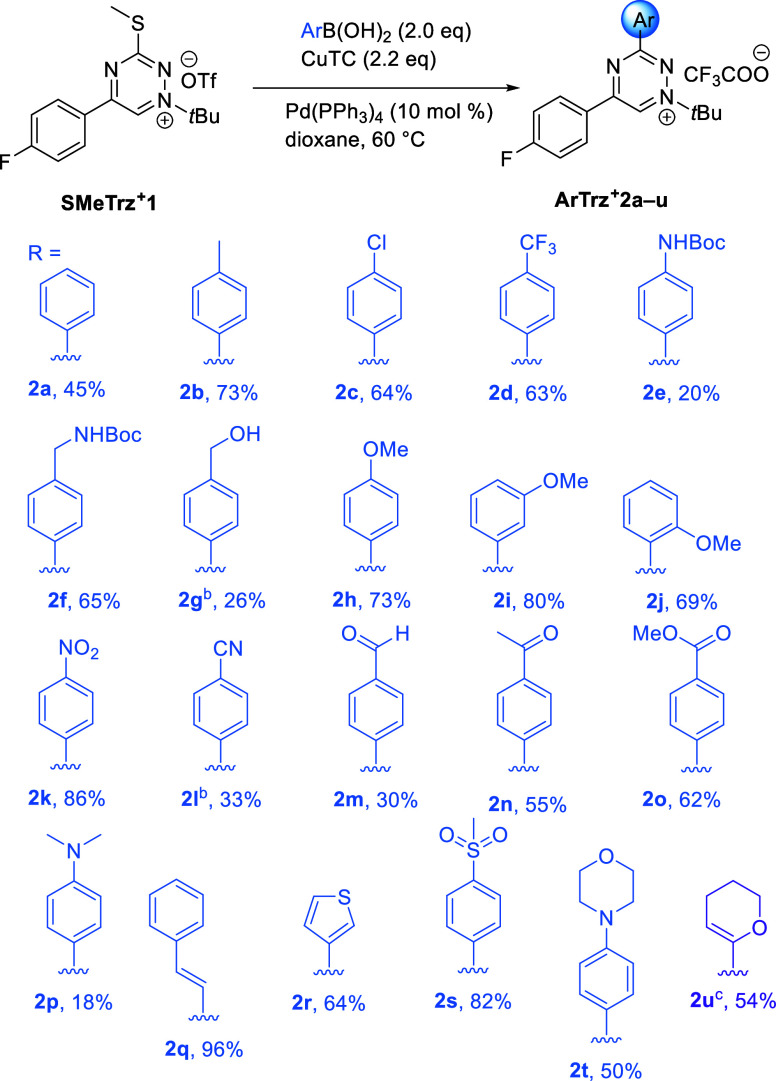
Scope of the L–S Cross-Coupling Reaction of **SMeTrz^+^1** under the Optimized Conditions Performed on a 0.117 mmol scale. Yield of the isolated product. Additional RB(OH)_2_ (2.0 equiv) and heating (90 °C) for 4 h. Tributyl(5,6-dihydro-4*H*-pyran-2-yl)stannane was used in the reaction.

Having established the reaction conditions, we proceeded to investigate the scope of the Cu-mediated, Pd-catalyzed L–S reaction by utilizing a range of arylboronic acids. These results are summarized in Scheme 1. Successful reactions were obtained in most cases. Specifically, unsubstituted phenyl (**2a**), *p*-tolyl (**2b**), *p*-chloro (**2c**), *p*-trifluoromethyl (**2d**), *p*-*N*Boc-aminomethyl (**2f**), *p*-methoxy (**2h**), *p*-nitro (**2k**), *p*-methyl carboxylate (**2o**), *p*-methanesulfonyl (**2s**), styryl (**2q**), *p*-morpholino (**2t**), and thiophene (**2r**) boronic acids provided coupling products after isolation in good to very good yields. In the case of the methoxyphenylboronic acid, all three *ortho*, *meta*, and *para* substitutions (**2h**–**2j**, respectively) were tolerated. Importantly, we successfully performed the C_3_-arylation with [4-(methoxycarbonyl)phenyl]boronic acid on a 1 mmol scale and isolated product **2o** in 71% yield (Supporting Information). However, several functional groups proved to be problematic. This was the case for the phenylhydroxymethyl (**2g**) and 4-cyanophenyl (**2l**) boronic acids, which exhibited diminished reactivity and provided the respective products after adding more boronic acid and increasing the temperature to 90 °C in lower yields. The electron-donating dimethylamino (**2p**) and *N*-Boc-amino (**2e**) substituents led to the formation of byproducts that complicated the separation of the desired cross-coupling product. Interestingly, the NMR analyses of products **2m** and **2n** containing the aldehyde or ketone group showed that these products spontaneously convert to the corresponding acetal (from **2m**) and to the hydrate (from **2n**) possibly as a result of the electron-withdrawing character of the triazinium moiety (for details, see the Supporting Information).

Our efforts to use 2-pyridyl- and 4-pyridylboronic acids, which have also proved to be problematic in other cross-coupling reactions,^30^ were unsuccessful. Inspired by a recent report on the considerable effect of intramolecular O–N repulsion on the reactivity of tetrazines,^31^ we installed a dihydropyran moiety at position C_3_ of the triazinium scaffold. Proceeding under optimal conditions, the reaction with tributyl(5,6-dihydro-4*H*-pyran-2-yl)stannane resulted in a 54% yield of the desired product. Unfortunately, our attempts to utilize other stannanes in the reaction and to further optimize the conditions were unsuccessful.

**Scheme 2:**
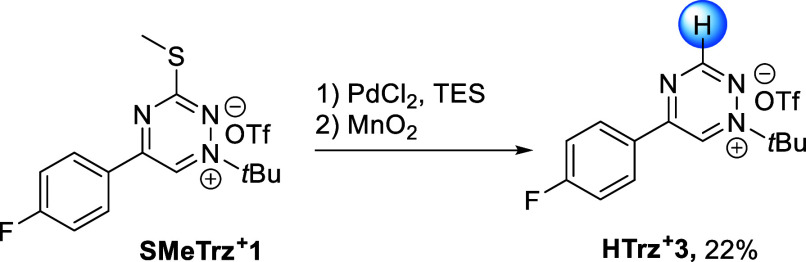
Synthesis of **HTrz^+^3** Conditions: 0.03 mM **SMeTrz**^**+**^**1**, TES (6.0 equiv), Pd(II)Cl_2_ (20 mol %), dioxane (1.0 mL), 55 °C, 17 h, then MnO_2_ (10 equiv), room temperature, 2 h (isolated in 22% yield).

Due to their high reactivity, H-substituted tetrazines are considered valuable bioorthogonal reagents.^26,32^ In this study, we prepared analogous monosubstituted H-triaziniums, which were accessed using a one-pot, two-step C_3_-thiomethyl reduction of **SMeTrz**^**+**^**1** followed by the reoxidation of the reduced intermediate. Inspired by a similar transformation reported for tetrazines,^26^ we speculated that the use of triethylsilane (TES) as the reductant and Pd(II)Cl_2_ as the catalyst would provide suitable starting conditions.^33^ Indeed, NMR spectroscopy confirmed the formation of the desired reduced **HTrz**^**+**^**3** under these conditions (Supporting Information). To optimize the reaction conditions, we focused on the reductant, catalyst, temperature, and solvent, along with different oxidizing agents (Table S2). Among the different conditions investigated, TES in combination with Pd(II)Cl_2_ proved to be most efficient for thiomethyl reduction; DDQ was the most effective oxidant (43% based on HPLC-MS analysis). However, for conducting the reaction on a preparative scale, heterogeneous oxidation with MnO_2_ was preferred as it facilitated the isolation of the product (Supporting Information).^34^ The optimal conditions involved performing the reaction in 1,4-dioxane at 55 °C using an excess of TES (6.0 equiv) as the reductant, Pd(II)Cl_2_ (20 mol %) as the catalyst, and the addition of MnO_2_ in one pot. Under these conditions, **HTrz**^**+**^**3** was isolated from the reaction mixture in 22% yield (Scheme 2).

**Figure 2:**
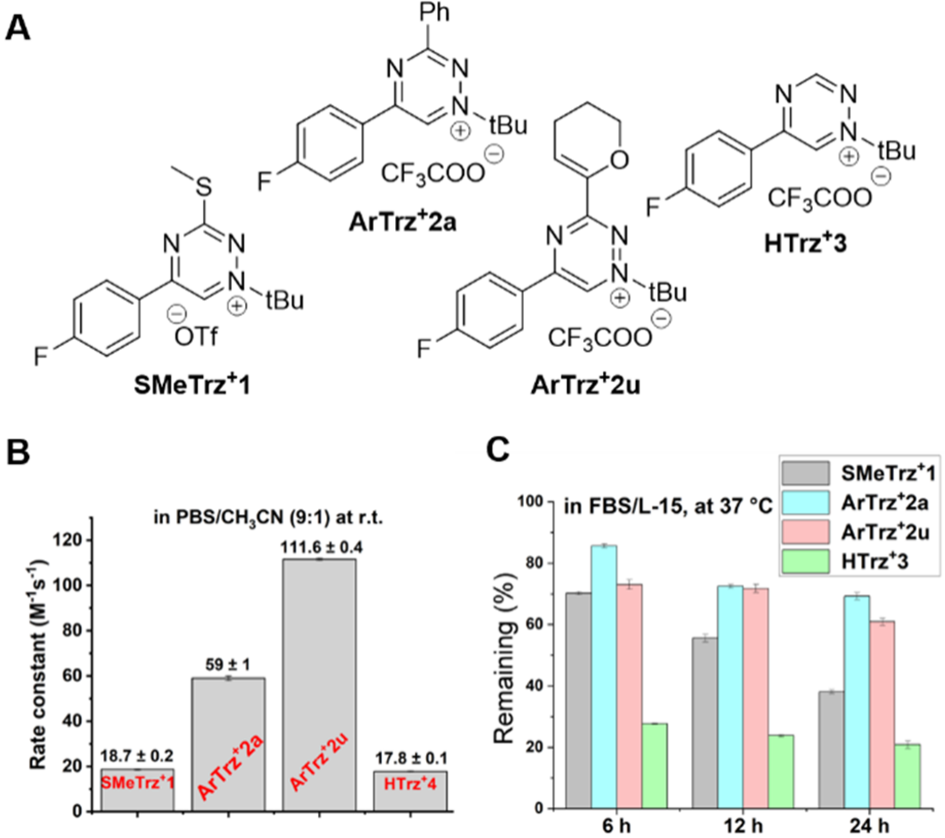
(A) Structures of the triazinium salts investigated. (B) Second-order rate constants (in M^–1^ s^–1^) for reactions of C_3_-substituted triaziniums with *endo*-BCN determined in a PBS/CH_3_CN (9:1) mixture at room temperature. (C) Stability of the same compounds at 37 °C in Leibovitz’s L-15 medium containing 10% FBS.

To study the impact of different modifications on ligation kinetics, second-order rate constants for representative derivatives were determined at room temperature in a PBS/CH_3_CN (9:1) mixture using an excess of *endo*-bicyclo[6.1.0]non-4-yn-9-ylmethanol (*endo*-BCN) (Supporting Information). On the basis of the measurements obtained, the reactivity of **ArTrz**^**+**^**2a** was ∼3 times higher than that of parent **SMeTrz**^**+**^**1** (Figure 2A,B), which shows that attaching the aryl substituent to position C_3_ of the triazinium ring accelerated the reaction. Monosubstituted triazinium **HTrz**^**+**^**3** proved to be as reactive as **SMeTrz**^**+**^**1**, which is interesting considering that H-tetrazines have proven to be more reactive than analogous derivatives bearing aromatic substituents or electron-donating groups.^35,36^ Finally, the fastest derivative in the series, (3,4-dihydro-3*H*-pyran-6-yl)-bearing **ArTrz**^**+**^**2u**, reacted with *endo*-BCN to provide a rate constant *k*_2_ of 111.6 ± 0.4 M^–1^ s^–1^. This finding indicates that intramolecular O–N repulsion, similar to what has been observed for tetrazines,^31^ is a viable strategy for increasing the click reactivity of triazinium salts.

Because the relationship between reactivity and stability is an interconnected feature of bioorthogonal reagents, we sought to examine how different C_3_ substituents would affect the stability of the compounds. According to our experimental results, approximately 60–70% of the most reactive **ArTrz**^**+**^**2a** and **ArTrz**^**+**^**2u** remained intact in full cell growth medium (Leibovitz’s L-15 medium) containing 10% fetal bovine serum (FBS) when incubated at 37 °C for 24 h (Figure 2C). Under these conditions, 55% of **SMeTrz**^**+**^**1** was present in the mixture after 12 h, whereas ∼40% of the compound remained unaffected after 24 h. Monosubstituted **HTrz**^**+**^**3** was the least stable derivative in the series. After the compound progressively degraded in the cell medium, only ∼25% of the compound remained in the solution after 24 h. As the C_3_-H-triaziniums exhibited rather low reactivity with *endo*-BCN, poor stability, and difficulties during synthesis, we deemed them unsuitable for applications under biological conditions.

**Scheme 3:**
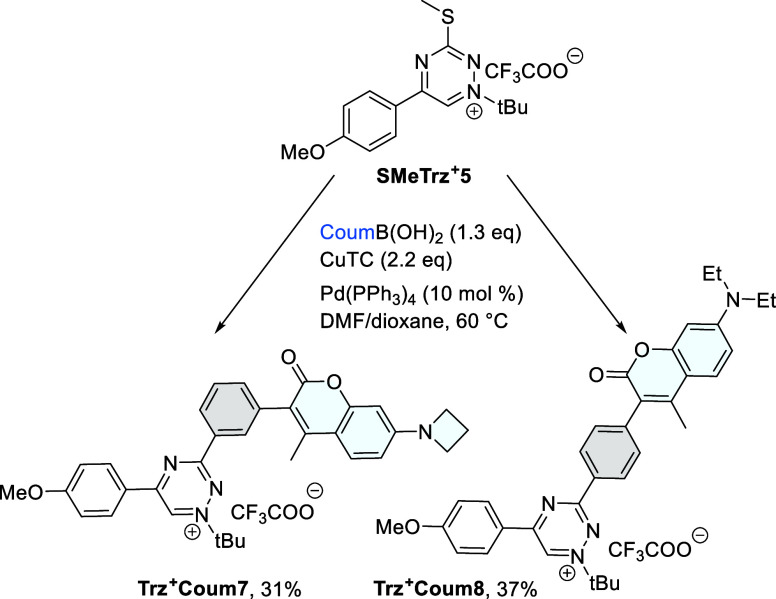
Synthesis of Fluorogenic **Trz^+^Coum** Probes

To further explore the use of triazinium salts in biological systems, we employed our L–S functionalization protocol for the synthesis of fluorescent probes. From the panel of dye conjugates previously prepared,^27^ we were particularly interested in coumarin derivatives, which we successfully used in live-cell labeling experiments. In our previous study, we established that a C_5_-phenyl-substituted triazinium, with diethylaminocoumarin attached through a phenylmethylene amino linker at position C_3_, exhibits a 6.5-fold increase in fluorescence upon reaction with *endo*-BCN.^27^ We discovered that substituting the phenyl group at C_5_ with an electron-donating 4-methoxyphenyl yielded triazinium derivative **Trz**^**+**^**Coum6**, which exhibited a notable enhancement in fluorescence upon reaction with *endo*-BCN in phosphate-buffered saline (PBS). On the basis of these findings, we designed a second generation of fluorogenic **Trz**^**+**^**Coum** probes containing the dye attached to the triazinium core through the conjugated phenyl ring. A similar strategy has been successfully used to enhance the fluorogenic properties of tetrazine dyes.^37−39^ To prepare the desired triazinium–dye conjugates, we used two coumarin-derived boronic acids in the L–S cross-coupling reaction, isolating the desired **Trz**^**+**^**Coum7** and **Trz**^**+**^**Coum8** after preparative HPLC in 31% and 37% yields, respectively (Scheme 3).

**Figure 3:**
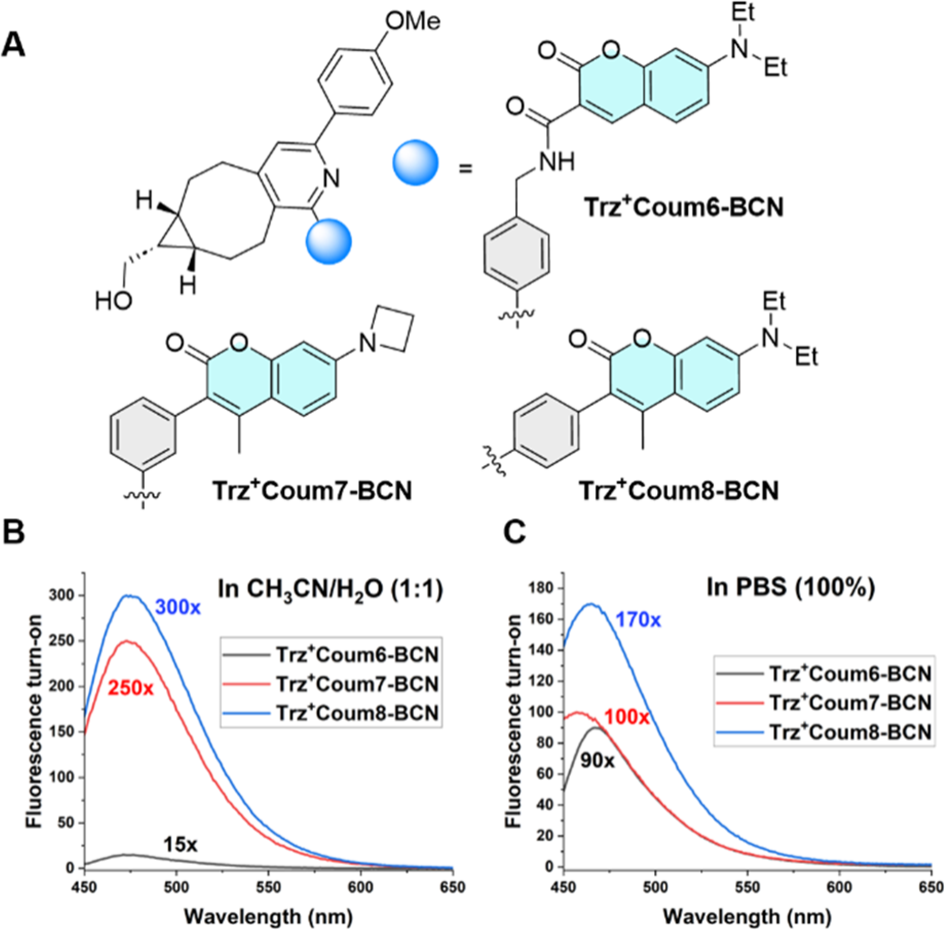
(A) Structures of the **Trz**^**+**^**Coum–BCN** conjugates. Graphs showing the fluorescence values of the click products formed in (B) a CH_3_CN/H_2_O (1:1) mixture and (C) PBS (100%).

The spectral properties of the compounds were studied in two different solvents: PBS and a CH_3_CN/H_2_O (1:1) mixture. The absorption maxima were red-shifted in the buffered system (Figure S6). Measuring the fluorescence turn-on values required fresh purification of the compounds by analytical HPLC, which removed traces of fluorescent impurities (Figure S7).^38^ The freshly purified **Trz**^**+**^ compounds were then mixed with an excess of *endo*-BCN. Finally, the fluorescence spectra of the resulting **Trz**^**+**^**Coum–BCN** conjugates were recorded (Figure 3B,C).

The fluorescence of all **Trz**^**+**^**Coum** probes was efficiently quenched before the reaction, and all compounds became strongly emissive after the click ligation with BCN (Figure 3 and Figure S8). Specifically, the probe containing coumarin attached to the triazinium core via the nonconjugated linker (**Trz**^**+**^**Coum6)** exhibited a 90-fold increase in fluorescence in 100% PBS and a 15-fold increase in fluorescence in the CH_3_CN/H_2_O mixture. Shortening the interchromophore distance in **Trz**^**+**^**Coum7** and **Trz**^**+**^**Coum8** resulted in notably higher fluorescence turn-on values of 250- and 300-fold in a CH_3_CN/H_2_O (1:1) mixture and 100- and 170-fold in PBS, respectively (Figure 3). These data show that attaching the coumarin to the charged triazinium via the conjugated phenyl ring enhances the fluorogenic properties of the conjugates.

**Figure 4:**
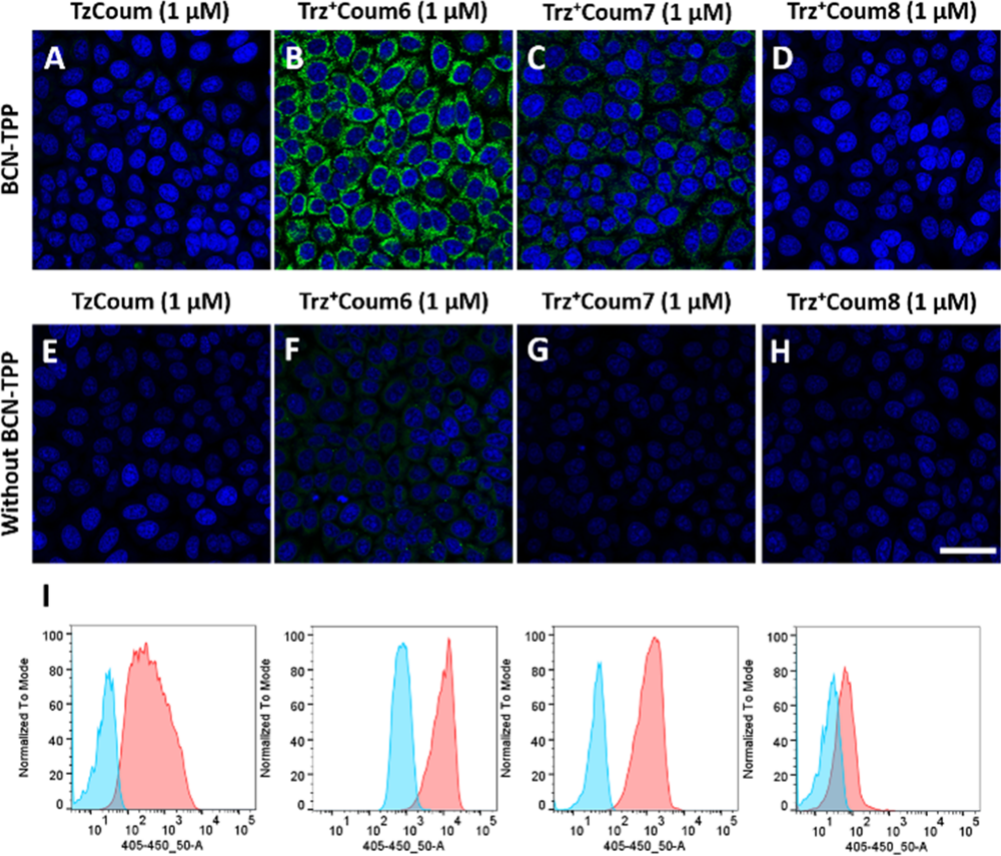
Comparison of fluorogenic triazinium and tetrazine ligations in intracellular bioimaging experiments. Living HeLa cells were treated with **BCN–TPP** (5 μM) for 10 min and then with (A) **TzCoum** (1 μM), (B) **Trz**^**+**^**Coum6** (1 μM), (C) **Trz**^**+**^**Coum7** (1 μM), and (D) **Trz**^**+**^**Coum8** (1 μM) for an additional 15 min. (E–H) Corresponding controls. All pictures were acquired without additional washing steps. The subsequent experiments, performed at different concentrations and microscope settings, are available in the Supporting Information (Figures S13 and S14). Cell nuclei were stained with the DRAQ5 dye (blue). The scale bar is 50 μm. (I) Flow cytometry analysis of cells labeled with coumarin probes.

We subsequently tested the compounds in a no-wash live-cell imaging experiment. To compare the triazinium reagents with the more established 1,2,4,5-tetrazines, we decided to use the structurally similar **TzCoum**, a fluorogenic compound that we previously selected from a pool of tetrazine–coumarin conjugates.^38^ The fluorescence turn-on of this probe in reaction with *endo*-BCN was 200-fold. In the first experiment, we performed the reaction inside living cells treated with a BCN–triphenylphosphonium conjugate (**BCN–TPP**), which we previously employed in intracellular fluorescence labeling studies.^40^ Living HeLa cells were treated with **BCN–TPP** for 10 min and subsequently washed to remove any extracellular compound. Cells that were not treated with **BCN–TPP** were used as controls. Next, the fluorogenic probes were added for 15 min at a low concentration of 1 μM, and the cells were inspected on a confocal microscope without additional washing steps. Under these conditions, the most intensive fluorescence signal formed in cells treated with **Trz**^**+**^**Coum6** followed by **Trz**^**+**^**Coum7** (Figure 4). In the case of **Trz**^**+**^**Coum6**, even a 100 nM concentration of the compound was sufficient for fluorescent cell labeling when the laser intensity of the microscope was increased from 1.5% to 2.5% and the detector sensitivity was increased from 650 to 750 V (Figure S13). These data indicate the utility of this derivative in biological imaging, contingent upon the correct configuration of the microscope. For quantitative comparison, we analyzed the cells using flow cytometry (Figure 4I). We found that the background fluorescent signal in cells treated with **Trz**^**+**^**Coum6** was higher than the signal in cells treated with other probes. The highest signal-to-background ratio was observed for **Trz**^**+**^**Coum7**, which was quenched in the cells, but then became highly fluorescent after the intracellular reaction with **BCN–TPP**. A closer comparison of **Trz**^**+**^**Coum7** and **TzCoum** using the same microscope settings showed that the two compounds were similarly efficient in labeling the dienophile inside living cells (Figure S14). Therefore, our experiments demonstrate that the triazinium moiety not only has the ability to quench the fluorescence of the attached coumarin dye but also exhibits a substantial restoration of fluorescence after the bioorthogonal reaction with the BCN dienophile. This enables the fluorogenic labeling of BCN-bearing probes inside living cells.

## Conclusion

In conclusion, we introduce a synthetic method for preparing C_3_-modified *N*1-*t*Bu-1,2,4-triazinium salts. This strategy, which combines an optimized L–S cross-coupling reaction of C_5_-aryl and C_3_-thiomethyl triazinium salts with boronic acids, enables the incorporation of diverse aryl groups at position C_3_. We also prepared an analogous H-triazinium derivative substituted with a hydrogen atom in the same position using a one-pot Pd-catalyzed reduction–oxidation reaction sequence. We investigated the reactivity and stability of a small series of derivatives. Surprisingly, H-substituted **HTrz**^**+**^**3** not only displayed relatively low reactivity with BCN but also proved to be unstable in the serum-containing full cell growth medium. In contrast, the C_3_-phenyl and especially the 3,4-dihydropyrane-substituted triazinium salts displayed excellent reactivity and stability. Our synthetic approach yields functionalized triazinium salts, as exemplified by the preparation of triazinium–coumarin conjugates. These dye-containing probes exhibit fluorogenic properties in reaction with BCN. This fluorogenicity is well-preserved under biological conditions, enabling no-wash fluorescent labeling in live cells. We expect that the method introduced in this work will facilitate the preparation of novel triazinium probes, the properties of which will be useful in a wide range of biological applications.

## Data Availability

The data underlying this study are available in the published article and its Supporting Information.

## Supporting Information Available

Synthetic procedures, characterization data of all compounds, kinetic measurements, additional cellular experiments, and fluorescence measurements (PDF)
^1^H, ^13^C, and ^19^F NMR spectra (PDF)
FAIR data, including the primary NMR FID files, for compounds **ArTrz**, **ArTrz^+^2a**–**ArTrz^+^2u**, **ArTrz^+^4**, **Coum-BA1**, **Coum-BA2**, **HTrz^+^3**, **RedHTrz^+^3**, **SMeTrz^+^1**, **SMeTrz^+^5**, **Trz^+^Coum6**, **Trz^+^Coum7**, and **Trz^+^Coum8** (ZIP)
