# Supplementary material for: Synthesis of C3-Substituted N1-tert-Butyl 1,2,4-Triazinium Salts via the Liebeskind–Srogl Reaction for Fluorogenic Labeling of Live Cells
Source: J Org Chem. 2024 Jan 15;89(20):14634–40. doi: 10.1021/acs.joc.3c02454 (PMC11494656; doi:10.1021/acs.joc.3c02454)
Supplement: Supplementary file 1 — jo3c02454_si_001.pdf [file jo3c02454_si_001.pdf]

Supporting Information

Synthesis of C3-Substituted *N*1-*tert*-Butyl 1,2,4-Triazinium Salts via the Liebeskind–Srogl Reaction for Fluorogenic Labeling of Live Cells

Veronika Šlachtová, Simona Bellová, and Milan Vrabel*

Institute of Organic Chemistry and Biochemistry of the Czech Academy of Sciences, Flemingovo nám. 2, 16000 Prague, Czech Republic

E-mail: vrabel@uochb.cas.cz

Table of Contents

General information S2

Optimization of ArTrz⁺ synthesis *via* Liebeskind-Srogl cross-coupling S2

Optimization of HTrz⁺ synthesis....S3

Synthetic procedures S4

Synthesis of precursor **Trz⁺1** on larger scale S4

Synthesis of **ArTrz⁺2s** *via* Liebeskind-Srogl cross coupling with boronic acids....S5

Synthesis of model **ArTrz⁺2u** *via* Liebeskind-Srogl cross coupling reaction with stannane S17

Synthesis of reduced C5-monosubstituted **red HTrz⁺3**....S18

Synthesis of aromatic C5-monosubstituted **arom HTrz⁺4** S19

Synthesis of fluorogenic triaziniums S19

Synthesis of various 1-(*tert*-butyl)-5-(4-methoxyphenyl)-**Trz⁺s** S19

Synthesis of coumarin-boronic acids S21

Synthesis of fluorogenic Trz⁺-coumarin compounds S23

Fluorescent properties....S25

HPLC-MS analysis of the reagents and the respective click products....S26

Absorbance of **SMeTrz⁺5** and the click products S27

Fluorescence turn-on measurements....S28

Reaction kinetics S31

Second order rate constants of differently C3-substituted **Trz+1** S31

Stability of *N*1-alkyl-1,2,4-triaziniums in cell growth medium DMEM containing FBS (fetal bovine serum) S32

Calculated log*P* values S33

Calculations of log*P*....................S33

BCN-TPP cell labeling experiments....................S34

Literature....................S37

General information

The chemicals were obtained from commercial suppliers and were used without further purification. ***endo*-BCN** (CAS 1263166-90-0) was obtained from SiChem, tributy(5,6-dihydro-*4H*-2-yl)stannane (CAS 109669-45-6) from Merck, 7-(diethylamino)coumarin-3-carboxylic acid NHS ester (CAS 139346-57-9) from abcr GmbH. Reactions with air- and moisture-sensitive reactants were performed in anhydrous solvents under argon atmosphere. Solutions were concentrated on a rotary evaporator *Heidolph* equipped with a PC3001 VARIOpro pump from *Vacuubrand*. Column chromatography was carried out on silica gel 60A (particle size: 40-60 µm) from *Acros Organics*. Mixtures of solvents are each stated as volume fractions. For flash column chromatography or preparative HPLC purification a Combi*Flash*® Rf+ from *Teledyne ISCO* or puri*Flash*® 5.250 from *Interchim* was used. Thin-layer chromatography was performed on aluminum sheets from *Merck* (silica gel 60 F254, 20 × 20 cm). Chromatograms were visualized by UV light (λ = 254 or 366 nm). ^{1}H- and ^{13}C-NMR spectra were measured at rt (20 °C) on a Bruker Avance III™ HD 400 MHz NMR system equipped with Prodigy cryo-probe or on a Bruker Avance III^{TM} 500 MHz NMR spectrometer (^{1}H at 500.0 MHz, ^{13}C at 125.7 MHz) and Bruker Avance III^{TM} 600 MHz NMR spectrometer (^{1}H at 600.1 MHz, ^{13}C at 150.9 MHz). Chemical shifts δ are quoted in ppm in relation to the chemical shift of the residual non-deuterated solvent peak MeOH-d_4: δ(^{1}H) = 3.31, δ(^{13}C) = 49.0; DMSO-d_6: δ(^{1}H) = 2.50; δ(^{13}C) = 39.4; CD_3CN-d_3: δ(^{1}H) = 1.94, δ(^{13}C) = 1.32). The structures were generally assigned based on 2D NMR experiments (HSQC, HMBC). High-resolution mass spectra were recorded on an *Agilent* 5975C MSD Quadrupol or LTQ Orbitrap XL from *Thermo Fisher Scientific*. UV/VIS spectroscopy was performed on a Cary 60 UV/VIS spectrophotometer from *Agilent Technologies*. The reaction progress was followed by HPLC-MS measurements on an LCMS-2020 system from *Shimadzu* equipped with a CORTECS C18 column (2.7 µm, 50 × 4.6 mm). Gradient of MeCN + 0.05% HCOOH in water + 0.05% HCOOH was used for the analysis. Fluorescence measurements were performed on a FluoroMax 4 spectrofluorometer (Jobin Yvon, Horiba) from Perkin Elmer equipped with a 450 W xenon lamp and a single cuvette reader. Pictures from the microscope were processed in ZEN software from ZEISS or in FIJI. Flow analysis data was processed in FlowJo.

Optimization of ArTrz$^+$ synthesis *via* Liebeskind-Srogl cross-coupling

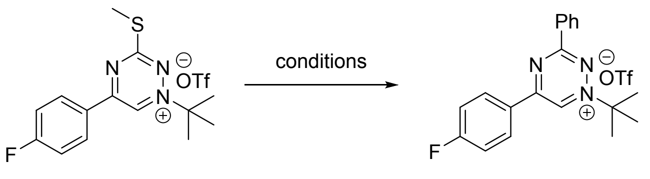

Table S1: Conditions explored for optimization of the Liebeskind-Srogl cross-coupling of **SMeTrz$^+$1**.

Entry	Nucleophile (eq.)	Ligand	Mediator	Solvent	T (°C)	ArTrz$^+$2 (%)[a]
**1**	$PhB(OH)_2$ (2.5)	$Pd(PPh_3)_4$	CuTC	dioxane	95	74
**2**	$PhB(OH)_2$ (2.5)	$Pd(PPh_3)_4$	CuTC	dioxane	60	81
**3**	$PhB(OH)_2$ (2.0)	$Pd(PPh_3)_4$	CuTC	dioxane	60	89
**4**	$PhB(OH)_2$ (1.5)	$Pd(PPh_3)_4$	CuTC	dioxane	60	77
**5**	$PhB(OH)_2$ (2.0)	$Pd(PPh_3)_4$	CuMeSal	dioxane	60	70
**6**	$PhB(OH)_2$ (2.0)	$Pd(PPh_3)_4$	Ag_2O	dioxane	60	13
**7**	$PhB(OH)_2$ (2.0)	$Pd(PPh_3)_4$	Cu_2O	dioxane	60	2
**8**	$PhB(OH)_2$ (2.0)	$Pd(dppf)Cl_2$.DCM	CuTC	dioxane	60	50
**9**	$PhB(OH)_2$ (2.0)	Pd_2dba_3	CuTC	dioxane	60	53
**10**	$PhB(OH)_2$ (2.0)	Pd_2dba_3 + $(P(2\text{-furyl})_3)$[b]	CuTC	dioxane	60	44
**11**	$PhB(OH)_2$ (2.0)	$Pd(PPh_3)_4$	CuTC	DMF	60	62
**12**	$PhB(OH)_2$ (2.0)	$Pd(PPh_3)_4$	CuTC	THF	60	78
**13**	PhBPin (2.0)	$Pd(PPh_3)_4$	CuTC	dioxane	60	3
**14**	$PhBF_3^-K^+$ (2.0)	$Pd(PPh_3)_4$	CuTC	dioxane	60	28
**15**	$PhSnBu_3$ (2.0)	$Pd(PPh_3)_4$	CuTC	dioxane	60	10

Conditions: (0.02 mM) **SMeTrz$^+$1**, nucleophile (eq.), ligand (10 mol%) + , mediator (2.2 eq.), solvent (1.0 mL), temp (°C), 4 h. [a]Yields determined by HPLC-MS using caffeine as an internal standard. Integrated areas from HPLC-MS chromatogram detected at λ = 278 nm. [b]Ligand $(P(2\text{-furyl})_3)$ (50 mol%).

Optimization of HTrz$^+$ synthesis

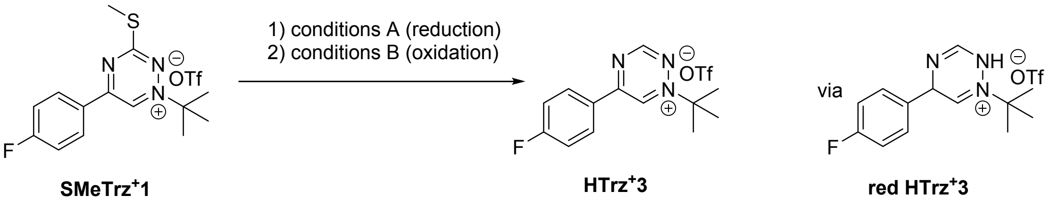

Table S2: Conditions explored for optimization of **HTrz$^+$3** synthesis.

Entry	Silane (eq.)	Catalyst (mol%)	Oxidant (eq.)	Solvent	T (°C)	Arom HTrz$^+$4 (%)[a]
**1**[b]	TES (6.0)	Pd(II)Cl_2 (20)	DDQ (1.5)	dioxane	55	43
**2**[b]	TES (4.0)	Pd(II)Cl_2 (20)	DDQ (1.5)	dioxane	55	9
**3**	TES (6.0)	Pd(II)Cl_2 (20)	DDQ (1.5)	dioxane	75	23
**4**	TIPS (6.0)	Pd(II)Cl_2 (20)	DDQ (1.5)	dioxane	75	3
**5**	TES (6.0)	Pd$(OAc)_2$ (20)	DDQ (1.5)	dioxane	75	23
**6**	TES (6.0)	Pd(II)Cl_2 (20)	DDQ (1.5)	dioxane/toluene (4:1)	75	23
**7**[b]	TES (6.0)	Pd(II)Cl_2 (20)	MnO_2 (10.0)	dioxane	55	28
**8**	TES (6.0)	Pd(II)Cl_2 (20)	PIDA (1.5)	dioxane	75	15
**9**	TES (6.0)	Pd(II)Cl_2 (20)	Isoamylnitrit (1.5)	dioxane	75	28
**10**	TES (6.0)	Pd(II)Cl_2 (20)	DMP (1.5)	dioxane	75	8
**11**	TES (6.0)	Pd(II)Cl_2 (20)	MnO_2 (10.0)	dioxane	75	14

Conditions (A): (0.03 mM) **SMeTrz$^+$1**, silane (eq.), catalyst (20 mol%), solvent (1.0 mL), temp (°C), 4 h. Conditions (B): oxidant (eq.), rt, 2 h. [a]Yields over one-pot two steps determined by HPLC-MS using caffeine as an internal standard. [b]Prolonged reaction time of 17 h. Integrated areas from HPLC-MS chromatogram detected at λ = 278 nm.

Synthetic procedures

Synthesis and characterization of the following 1,2,4-triazines, 1,2,4-triaziniums, coumarins and BCN-TPP were reported previously:

SMeTrz1[1], Trz2[1], SMeTrz$^+$1,[2] GJPV517B[3], GJPV573B[3], GJPV503[3], BCN-TPP[4].

Synthesis of precursor Trz$^+$1 on larger scale

1-(*tert*-Butyl)-5-(4-fluorophenyl)-3-(methylthio)-1,2,4-triazin-1-ium trifluoromethanesulfonate (SMeTrz$^+$1)

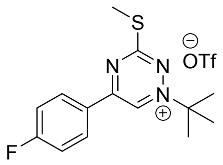

SMeTrz1[1] (1.0 equiv., 6.454 mmol, 1.43 g) was dissolved in dry DCM (65 mL) under argon and cooled to 0 °C. Isobutene gas was condensed in a Schlenk tube at -78 °C (dry ice/acetone bath). Subsequently, an excess of liquid isobutene (3.2 mL) was added followed by dropwise addition of trifloromethanesulfonic acid (1.2 equiv., 7.745 mmol, 685 µL) at 0 °C and the mixture was stirred at rt for 1 h. Crude product was concentrated under reduced pressure and purified by RP flash chromatography (RediSep Gold C18Aq 275 g column). MeCN/H_2O gradient (flow rate 100 mL/min) 0:100 → 60:40 for 20 min. Product detection at λ = 275 and 335 nm, lyophilization.

Yield: 2.17 g (5.08 mmol, 97%) of a yellow amorphous solid.

^{1}H NMR (400 MHz, CD_3CN): δ 9.46 (s, 1H), 8.50 – 8.42 (m, 2H), 7.49 – 7.39 (m, 2H), 2.77 (s, 3H), 1.85 (s, 9H).

^{19}F NMR (376 MHz, CD_3CN): δ -79.2, -102.7 (tt, J = 8.6, 5.2 Hz).

^{13}C{1H} NMR (101 MHz, CD_3CN): δ 179.6, 168.2 (d, $^1J_{C,F}$ = 257, C-F), 164.8, 135.0, 133.5 (d, $^3J_{C,F}$ = 9.9, 2×CH), 128.4 (d, $^4J_{C,F}$ = 3.0, C), 118.1 (d, $^2J_{C,F}$ = 22.7, 2×CH), 78.8, 28.6 (3×CH_3), 14.8 (S$\underline{CH_3}$).

HRMS (ESI): m/z calcd. for $C_{14}H_{17}N_3FS$ [M^+] 278.1121, found 278.1121.

Synthesis of **ArTrz⁺2s** *via* Liebeskind-Srogl cross coupling with boronic acids

General procedure A: SMeTrz⁺1[2] (1.0 equiv., 0.117 mmol), boronic acid (2.0 equiv., 0.234 mmol), copper(I) thiophene-2-carboxylate (2.2 equiv., 0.257 mmol) and $Pd(PPh_3)_4$ (10 mol%, 0.012 mmol) were placed in an argon flushed flask and anhydrous 1,4-dioxane (6.0 mL) was added. The mixture was stirred under argon at 60 °C (heating mantle) for 4-8 h (LC-MS analysis). After cooling to rt, the mixture was diluted with DCM (50 mL), washed with sat. aq. $NaHCO_3$ (50 mL) and extracted with DCM (2×20 mL). The combined organic extracts were dried over Na_2SO_4, filtered, and concentrated under reduced pressure. The residue was then re-dissolved in (9:1) MeCN/H_2O (+ 0.1% TFA) (15 mL), filtered through a short pad of sand/C18® (Santiago; 230-400 MESH) and washed with (9:1) MeCN/H_2O (+ 0.1% TFA) (3×15 mL). The filtrate was concentrated under reduced pressure and the crude product was purified by preparative HPLC (Arion Plus (21.2 × 250 mm, 5 µm) or YMC-Actus Triart (20.0 × 250 mm, 5 µm) column: flow rate of 15 mL/min). The solvent system and characteristic absorption are shown for each derivative separately.

1-(*tert*-Butyl)-5-(4-fluorophenyl)-3-phenyl-1,2,4-triazin-1-ium 2,2,2-trifluoroacetate (ArTrz⁺2a)

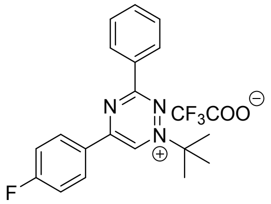

Purification: MeCN/H_2O (+ 0.1% TFA) gradient 15:85 → 60:40 for 30 min. Product detection at λ = 278 and 338 nm.

Yield: 22.0 mg (0.052 mmol, 45%) of a light yellow amorphous solid.

^{1}H NMR (401 MHz, CD_3CN): δ 9.99 (s, 1H), 8.75 – 8.67 (m, 2H), 8.64 – 8.58 (m, 2H), 7.82 – 7.76 (m, 1H), 7.74 – 7.66 (m, 2H), 7.52 – 7.44 (m, 2H), 1.95 (s, 9H).

^{19}F NMR (377 MHz, CD_3CN): δ -75.87, -103.06 (tt, J = 8.2, 5.2 Hz).

^{13}C{1H} NMR (101 MHz, CD_3CN): δ 168.2 (d, $^{1}J_{C,F}$ = 257, C-F), 167.4, 166.5, 137.7, 135.4, 133.6 (d, $^{3}J_{C,F}$ = 10.1, 2×CH), 132.9, 130.6 (2×CH), 130.1 (2×CH), 129.1 (d, $^{4}J_{C,F}$ = 3.1, C), 118.1 (d, $^{2}J_{C,F}$ = 23.5, 2×CH), 78.8, 28.8 (3×CH_3).

HRMS (ESI): m/z calcd. for $C_{19}H_{19}N_3F$ [M^+] 308.1558, found 308.1556.

1-(*tert*-Butyl)-5-(4-fluorophenyl)-3-(*p*-tolyl)-1,2,4-triazin-1-ium 2,2,2-trifluoroacetate (ArTrz$^+$2b)

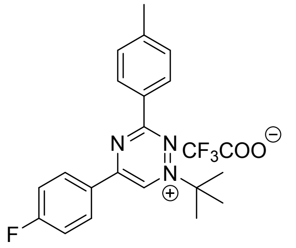

Purification: MeCN/H_2O (+ 0.1% TFA) gradient 15:85 → 75:25 for 30 min. Product detection at λ = 294 nm.

Yield: 37.4 mg (0.086 mmol, 73%) of a light brown amorphous solid.

^{1}H NMR (400 MHz, CD_3CN): δ 9.72 (s, 1H), 8.67 – 8.60 (m, 2H), 8.54 – 8.48 (m, 2H), 7.55 – 7.45 (m, 4H), 2.50 (s, 3H), 1.94 (s, 9H).

^{19}F NMR (376 MHz, CD_3CN): δ -76.28, -102.63 – -103.41 (m).

^{13}C{1H} NMR (101 MHz, CD_3CN): δ 168.2 (d, $^{1}J_{C,F}$ = 257, C-F), 167.7, 166.3, 147.0, 136.7, 133.4 (d, $^{3}J_{C,F}$ = 9.9, 2×CH), 131.3 (2×CH), 130.2 (2×CH), 130.1, 128.9 (d, $^{4}J_{C,F}$ = 2.2, C), 118.0 (2×CH), 78.8, 28.8 (3×CH_3), 21.8 (CH_3).

HRMS (ESI): m/z calcd. for $C_{20}H_{21}N_3F$ [M^+] 322.1714, found 322.1715.

1-(*tert*-Butyl)-3-(4-chlorophenyl)-5-(4-fluorophenyl)-1,2,4-triazin-1-ium 2,2,2-trifluoroacetate (ArTrz$^+$2c)

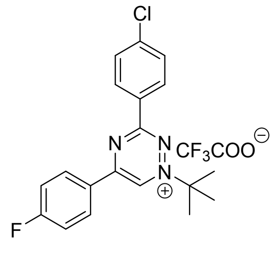

Purification: MeCN/H_2O (+ 0.1% TFA) gradient 15:85 → 75:25 for 30 min. Product detection at λ = 290 nm.

Yield: 34.0 mg (0.075 mmol, 64%) of a light greenish amorphous solid.

^1H NMR (400 MHz, MeOD): δ 10.13 (s, 1H), 8.79 – 8.72 (m, 2H), 8.66 – 8.59 (m, 2H), 7.76 – 7.69 (m, 2H), 7.54 – 7.46 (m, 2H), 2.01 (s, 9H).

^{19}F NMR (376 MHz, MeOD): δ -76.83, -102.99 – -103.44 (m).

$^{13}C\{1H\}$ NMR (101 MHz, MeOD): δ 168.7 (d, $^1J_{C,F}$ = 259, C-F), 167.0 (2×C), 141.8, 138.1, 133.8 (d, $^3J_{C,F}$ = 10.0, 2×CH), 132.1, 131.8 (2×CH), 130.9 (2×CH), 129.5 (d, $^4J_{C,F}$ = 3.0, C), 118.2 (d, $^2J_{C,F}$ = 22.7, 2×CH), 78.9, 28.9 (3×CH_3).

HRMS (ESI): m/z calcd. for $C_{19}H_{18}N_3ClF$ [M^+] 342.1165, found 342.1167.

1-(*tert*-Butyl)-5-(4-fluorophenyl)-3-(4-(trifluoromethyl)phenyl)-1,2,4-triazin-1-ium 2,2,2-trifluoroacetate (ArTrz⁺2d)

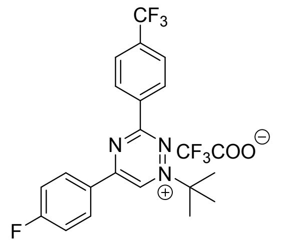

Purification: MeCN/H_2O (+ 0.1% TFA) gradient 15:85 → 75:25 for 30 min. Product detection at λ = 344 nm. The isolated product was re-purified by silica gel column chromatography (solvent system: MeOH/DCM (+0.1% TFA) gradient 0:100 → 10:90.

Yield: 36.0 mg (0.074 mmol, 63%) of a light yellow amorphous solid.

^1H NMR (401 MHz, CD_3CN): δ 9.99 (s, 1H), 8.81 – 8.75 (m, 2H), 8.75 – 8.68 (m, 2H), 8.03 – 7.98 (m, 2H), 7.53 – 7.46 (m, 2H), 1.96 (s, 9H).

^{19}F NMR (377 MHz, CD_3CN): δ -63.75, -76.23, -102.42 (tt, J = 8.6, 5.4 Hz).

$^{13}C\{1H\}$ NMR (101 MHz, CD_3CN): δ 168.4 (d, $^1J_{C,F}$ = 257, C-F), 166.8, 166.3, 138.4, 136.5, 135.5 (q, $^2J_{C,F}$ = 32.6 Hz, C-CF_3), 133.8 (d, $^3J_{C,F}$ = 10.1, 2×CH), 130.8 (2×CH), 128.8 (d, $^4J_{C,F}$ = 3.1, C), 127.5 (q, $^3J_{C,F}$ = 4.0 Hz, 2×CH), 124.7 (q, $^1J_{C,F}$ = 272 Hz, CF_3), 118.2 (d, $^2J_{C,F}$ = 23.5, 2×CH), 79.2, 28.8 (3×CH_3).

HRMS (ESI): m/z calcd. for $C_{20}H_{18}N_3F_4$ [M^+] 376.1431, found 376.1428.

3-(4-((*tert*-Butoxycarbonyl)amino)phenyl)-1-(*tert*-butyl)-5-(4-fluorophenyl)-1,2,4-triazin-1-ium 2,2,2-trifluoroacetate (ArTrz⁺2e)

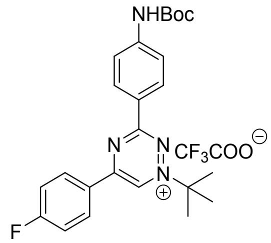

Purification: MeCN/H_2O (+ 0.1% TFA) gradient 15:85 → 75:25 for 30 min. Product detection at λ = 342 nm. Re-purified by column chromatography on silica gel in HILIC mode (puri*Flash*® from *InterChim,* SiO_2 (40 g) column). H_2O/MeCN (+ 0.1% TFA) gradient 0:100 → 5:95 and flow rate 35 mL/min for 20 min and lyophilized.

Yield: 12.0 mg (0.022 mmol, 20%) of a bright yellow amorphous solid.

^1H NMR (401 MHz, CD_3CN): δ 9.69 (br s, 1H), 8.65 – 8.58 (m, 2H), 8.55 – 8.49 (m, 2H), 8.29 (br s, 1H), 7.76 – 7.70 (m, 2H), 7.51 – 7.44 (m, 2H), 1.92 (s, 9H), 1.52 (s, 9H).

^{19}F NMR (377 MHz, CD_3CN): δ -76.24, -103.23 (tt, *J* = 8.2, 5.2 Hz).

$^{13}C\{1H\}$ NMR (101 MHz, CD_3CN): δ 168.1 (d, $^1J_{C,F}$ = 257, C-F), 167.2, 166.1, 153.6, 146.6, 136.1, 133.3 (d, $^3J_{C,F}$ = 9.9, 2×CH), 131.5 (2×CH), 129.0 (d, $^4J_{C,F}$ = 2.9, C), 126.0, 119.2 (2×CH), 117.2 (d, $^2J_{C,F}$ = 22.7, 2×CH), 81.5, 78.6, 28.8 (3×CH_3), 28.4 (3×CH_3).

HRMS (ESI): m/z calcd. for $C_{24}H_{28}O_2N_4F$ [M^+] 423.2190, found 423.2190.

3-(4-(((tert-butoxycarbonyl)amino)methyl)phenyl)-1-(tert-butyl)-5-(4-fluorophenyl)-1,2,4-triazin-1-ium 2,2,2-trifluoroacetate (ArTrz⁺2f)

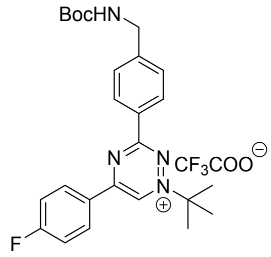

Purification: MeCN/H_2O (+ 0.1% TFA) gradient 15:85 → 75:25 for 30 min. Product detection at λ = 288 nm.

Yield: 42.0 mg (0.076 mmol, 65%) of a light brown amorphous solid.

^1H NMR (401 MHz, CD_3CN): δ 9.81 (s, 1H), 8.71 – 8.62 (m, 2H), 8.60 – 8.53 (m, 2H), 7.61 – 7.55 (m, 2H), 7.49 (t, *J* = 8.7 Hz, 2H), 6.00 (br s, 1H), 4.36 (d, *J* = 6.1 Hz, 2H), 1.94 (s, 9H), 1.44 (s, 9H).

^{19}F NMR (377 MHz, CD_3CN): δ -75.29, -102.95 (dq, *J* = 8.2, 4.3 Hz).

$^{13}C\{1H\}$ NMR (101 MHz, CD_3CN): δ 168.2 (d, $^{1}J_{C,F}$ = 257, C-F), 167.5, 166.4, 148.6, 137.0, 133.4 (d, $^{3}J_{C,F}$ = 9.9, 2×CH), 131.6, 130.3 (2×CH), 129.1 (2×CH), 128.9 (d, $^{4}J_{C,F}$ = 2.2, C), 118.0 (2×CH), 79.7, 78.8, 44.6 (C$\underline{C}H_2$NH), 28.8 (3×CH_3), 28.6 (3×CH_3). Signal of one quart. carbon was not found.

HRMS (ESI): m/z calcd. for $C_{25}H_{30}O_2N_4F$ [M^+] 437.2347, found 437.2344.

1-(*tert*-Butyl)-5-(4-fluorophenyl)-3-(4-(hydroxymethyl)phenyl)-1,2,4-triazin-1-ium 2,2,2-trifluoroacetate (ArTrz$^+$2g)

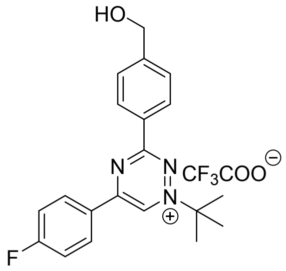

After stirring the reaction mixture under argon at 60 °C for 4 h, the mixture was cooled to rt and additional boronic acid (2.0 equiv., 0.234 mmol) was added. Stirring under argon then continued at 90 °C for 4 more hours. Purification: MeCN/H_2O (+ 0.1% TFA) gradient 15:85 → 55:45 for 30 min. Product detection at λ = 290 nm. Re-purified by column chromatography on silica gel in HILIC mode (puri*Flash*® from *InterChim,* SiO_2 (40 g) column). H_2O/MeCN (+ 0.1% TFA) gradient 0:100 → 5:95 and flow rate 35 mL/min for 20 min and lyophilized.

Yield: 14.0 mg (0.031 mmol, 26%) of a light brown amorphous solid.

^{1}H NMR (400 MHz, CD_3CN): 9.69 (s, 1H), 8.65 – 8.60 (m, 2H), 8.59 – 8.56 (m, 2H), 7.67 – 7.62 (m, 2H), 7.52 – 7.46 (m, 2H), 4.74 (s, 2H), 1.93 (s, 9H).

^{19}F NMR (376 MHz, CD_3CN): -76.54, -102.89 (tt, *J* = 8.6, 5.2 Hz).

$^{13}C\{1H\}$ NMR (101 MHz, CD_3CN): δ 168.0 (d, $^{1}J_{C,F}$ = 257, C-F), 167.7, 166.4, 150.6, 136.8, 133.4 (d, $^{3}J_{C,F}$ = 9.9, 2×CH), 131.3, 130.2 (2×CH), 128.9 (d, $^{4}J_{C,F}$ = 2.2, C), 128.3 (2×CH), 118.0 (2×CH), 78.9, 64.0 (C$\underline{C}H_2$OH), 28.8 (3×CH_3).

HRMS (ESI): m/z calcd. for $C_{20}H_{21}ON_3F$ [M^+] 338.1662, found 338.1663.

1-(*tert*-Butyl)-5-(4-fluorophenyl)-3-(4-methoxyphenyl)-1,2,4-triazin-1-ium 2,2,2-trifluoroacetate (ArTrz$^+$2h)

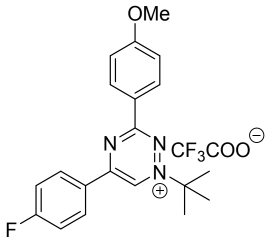

Purification: MeCN/H_2O (+0.1% TFA) gradient 5:95 → 70:30 for 30 min. Product detection at λ = 330 nm.

Yield: 38.5 mg (0.085 mmol, 73%) of a bright orange amorphous solid.

^{1}H NMR (400 MHz, CD_3CN): δ 9.64 (s, 1H), 8.65 – 8.55 (m, 4H), 7.52 – 7.44 (m, 2H), 7.23 – 7.18 (m, 2H), 3.95 (s, 3H), 1.93 (s, 9H).

^{19}F NMR (376 MHz, CD_3CN): δ -75.97, -103.23 (tt, J = 8.4, 5.2 Hz).

^{13}C{1H} NMR (101 MHz, CD_3CN): δ 168.2 (d, $^1J_{C,F}$ = 257, C-F), 167.4, 166.17, 166.12, 136.0, 133.3 (d, $^3J_{C,F}$ = 10.0, 2×CH), 132.4 (2×CH), 129.5 (d, $^4J_{C,F}$ = 3.0, C), 125.0, 118.2 (d, $^2J_{C,F}$ = 22.7, 2×CH), 116.5 (2×CH), 78.7, 56.6 (O$\underline{C}H_3$), 28.8 (3×CH_3).

HRMS (ESI): m/z calcd. for $C_{20}H_{21}ON_3F$ [M^+] 338.1663, found 338.1663.

1-(*tert*-Butyl)-5-(4-fluorophenyl)-3-(3-methoxyphenyl)-1,2,4-triazin-1-ium 2,2,2-trifluoroacetate (ArTrz$^+$2i)

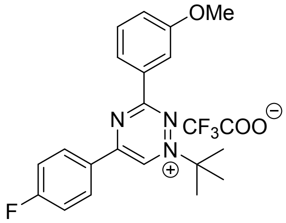

Purification: MeCN/H_2O (+ 0.1% TFA) gradient 15:85 → 70:30 for 30 min. Product detection at λ = 340 nm.

Yield: 42.0 mg (0.093 mmol, 80%) of a bright yellow amorphous solid.

^{1}H NMR (400 MHz, CD_3CN): 9.74 (s, 1H), 8.69 – 8.61 (m, 2H), 8.21 (ddd, J = 7.7, 1.7, 0.9 Hz, 1H), 8.09 (dd, J = 2.8, 1.7 Hz, 1H), 7.62 (t, J = 8.1 Hz, 1H), 7.54 – 7.45 (m, 2H), 7.35 (dd, J = 8.3, 1.0 Hz, 1H), 3.95 (s, 3H), 1.95 (s, 9H).

^{19}F NMR (376 MHz, CD_3CN): δ -76.08, -102.75 (tt, J = 8.6, 5.4 Hz).

^{13}C{1H} NMR (101 MHz, CD_3CN): δ 168.3 (d, $^1J_{C,F}$ = 257, C-F), 167.5, 166.4, 161.6, 137.2, 134.1, 133.5 (d, $^3J_{C,F}$ = 10.2, 2×CH), 131.9, 128.8 (d, $^4J_{C,F}$ = 2.9, C), 122.5, 121.1, 118.1 (2×CH), 115.0, 79.0, 56.4 (O$\underline{C}H_3$), 28.8 (3×CH_3).

HRMS (ESI): m/z calcd. for $C_{20}H_{21}ON_3F$ [M^+] 338.1663, found 338.1660.

1-(*tert*-Butyl)-5-(4-fluorophenyl)-3-(2-methoxyphenyl)-1,2,4-triazin-1-ium 2,2,2-trifluoroacetate (ArTrz⁺2j)

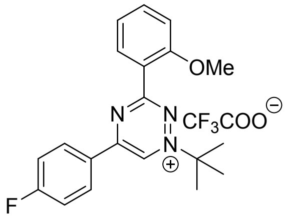

Purification: MeCN/H_2O (+ 0.1% TFA) gradient 15:85 → 70:30 for 30 min. Product detection at λ = 340 nm.

Yield: 36.0 mg (0.080 mmol, 69%) of a bright yellow amorphous solid.

^{1}H NMR (400 MHz, CD_3CN): δ 9.77 (s, 1H), 8.62 – 8.55 (m, 2H), 8.18 (dd, *J* = 7.8, 1.8 Hz, 1H), 7.72 (ddd, *J* = 8.4, 7.3, 1.8 Hz, 1H), 7.51 – 7.43 (m, 2H), 7.30 (dd, *J* = 8.5, 1.0 Hz, 1H), 7.27 – 7.21 (m, 1H), 3.98 (s, 3H), 1.92 (s, 9H).

^{19}F NMR (376 MHz, CD_3CN): δ -76.12, -103.01 – -103.81 (m).

^{13}C{1H} NMR (101 MHz, CD_3CN): δ 168.2, 168.1 (d, $^{1}J_{C,F}$ = 257, C-F), 165.9, 160.5, 136.3, 136.2, 133.7, 133.3 (d, $^{3}J_{C,F}$ = 9.9. 2×CH), 129.1 (d, $^{4}J_{C,F}$ = 2.9, C), 122.2, 122.0, 118.1 (d, $^{2}J_{C,F}$ = 22.7, 2×CH), 114.1, 78.6, 57.0 (O<u>C</u>H_3), 28.7 (3×CH_3).

HRMS (ESI): m/z calcd. for $C_{20}H_{21}ON_3F$ [M^+] 338.1663, found 338.1661.

1-(*tert*-Butyl)-5-(4-fluorophenyl)-3-(4-nitrophenyl)-1,2,4-triazin-1-ium 2,2,2-trifluoroacetate (ArTrz⁺2k)

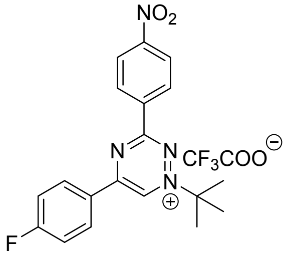

Purification: MeCN/H_2O (+ 0.1% TFA) gradient 15:85 → 70:30 for 30 min. Product detection at λ = 342 nm.

Yield: 47.0 mg (0.101 mmol, 86%) of a bright yellow amorphous solid.

^{1}H NMR (400 MHz, MeOD): δ 10.24 (s, 1H), 8.94 – 8.84 (m, 2H), 8.83 – 8.76 (m, 2H), 8.61 – 8.50 (m, 2H), 7.59 – 7.48 (m, 2H), 2.03 (s, 9H).

^{19}F NMR (376 MHz, MeOD): δ --77.09, -102.50 (tt, *J* = 8.6, 5.2 Hz).

$^{13}C\{1H\}$ NMR (101 MHz, MeOD): δ 169.0 (d, $^{1}J_{C,F}$ = 257, C-F), 167.4, 166.2, 152.6, 139.1, 138.7, 133.9 (d, $^{3}J_{C,F}$ = 9.9. 2×CH), 131.5 (2×CH), 129.3 (d, $^{4}J_{C,F}$ = 2.9, C), 125.5 (2×CH), 118.3 (d, $^{2}J_{C,F}$ = 22.7, 2×CH), 79.3, 28.9 (3×CH_3).

HRMS (ESI): m/z calcd. for $C_{19}H_{18}O_2N_4F$ [M^+] 353.1408, found 353.1407.

1-(tert-Butyl)-3-(4-cyanophenyl)-5-(4-fluorophenyl)-1,2,4-triazin-1-ium 2,2,2-trifluoroacetate (ArTrz$^+$2l)

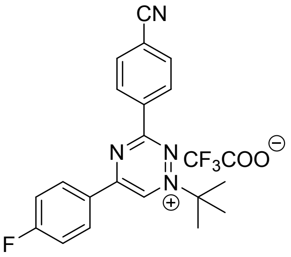

After stirring under argon at 60 °C for 4 h, the mixture was cooled to rt, and additional boronic acid (2.0 equiv., 0.234 mmol) was added. Stirring under argon then continued at 90 °C for 4 more hours. Purification: MeCN/H_2O (+ 0.1% TFA) gradient 15:85 → 65:35 for 30 min. Product detection at λ = 345 nm.

Yield: 17.0 mg (0.038 mmol, 33%) of a light brown amorphous solid.

^{1}H NMR (400 MHz, CD_3CN): δ 9.92 (s, 1H), 8.77 – 8.72 (m, 2H), 8.72 – 8.66 (m, 2H), 8.08 – 8.01 (m, 2H), 7.54 – 7.46 (m, 2H), 1.95 (s, 9H).

^{19}F NMR (376 MHz, CD_3CN): δ -76.25, -102.21 (tt, J = 8.6, 5.2 Hz).

$^{13}C\{1H\}$ NMR (101 MHz, CD_3CN): δ 168.5 (d, $^{1}J_{C,F}$ = 257, C-F), 166.8, 166.2, 138.3, 136.7, 134.3 (2×CH), 133.8 (d, $^{3}J_{C,F}$ = 10.3, 2×CH), 130.6 (2×CH), 128.7 (d, $^{4}J_{C,F}$ = 2.9, C), 118.9 (CN), 118.1 (2×CH), 79.3, 28.7 (3×CH_3). Signal of one quart. carbon was not found.

HRMS (ESI): m/z calcd. for $C_{20}H_{18}N_4F$ [M^+] 333.1508, found 333.1510.

1-(*tert*-Butyl)-3-(4-(dihydroxymethyl)phenyl)-5-(4-fluorophenyl)-1,2,4-triazin-1-ium 2,2,2-trifluoroacetate (ArTrz$^+$2m)

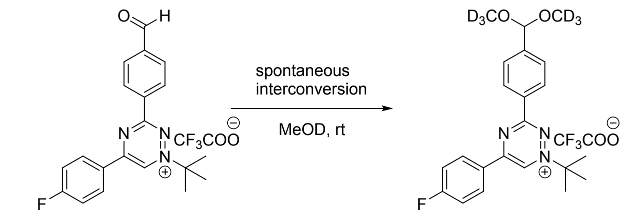

C3: formylPh Trz+ (ArTrz$^+$2m)   C3: diOCD_3Ph Trz+ (ArTrz$^+$2m')

Purification: MeCN/H_2O (+ 0.1% TFA) gradient 15:85 → 70:30 for 30 min. Product detection at λ = 285 and 342 nm. Re-purified by column chromatography on silica gel in HILIC mode (puri*Flash*® from *InterChim,* SiO_2 (40 g) column). H_2O/MeCN (+ 0.1% TFA) gradient 0:100 → 5:95 and flow rate 35 mL/min for 20 min

and lyophilized. The originally isolated **C3: formylPh Trz⁺ (ArTrz⁺2m)** spontaneously converted to the corresponding acetal **C3: diOCD$_3$Ph Trz$^+$ (ArTrz$^+$2m')** during NMR characterization in CD_3OD.

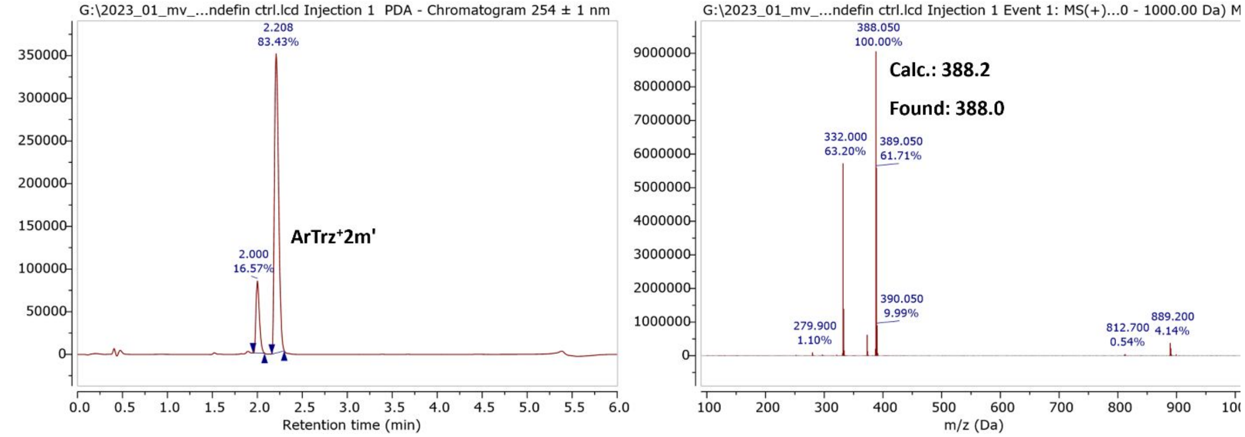

Figure S1: Exported HPLC chromatogram of the mixture of **diOCD3Ph Trz⁺ (ArTrz⁺2m')** and the parent **formylPh Trz⁺ (ArTrz⁺2m)** (mixed after NMR analysis, chromatogram at λ = 254 nm) and the corresponding MS spectrum of the acetal. Signal at 2 min corresponds to the original aldehyde and signal at 2.21 min to the acetal. Solvent system: solvent A: H_2O + 0.05% HCOOH; solvent B: MeCN + 0.05% HCOOH. Gradient: 5% B → 95% B (4 min), then 95% B (0.5 min), then 95% B → 5% B (0.5 min) and 5% B (1 min).

Yield: 16.0 mg (0.034 mmol, 30%) of a light yellow amorphous solid.

^{1}H NMR (401 MHz, MeOD): δ 10.12 (s, 1H), 7.83 – 7.74 (m, 2H), 8.69 – 8.59 (m, 2H), 7.83 – 7.74 (m, 2H), 7.53 – 7.48 (m, 2H), 5.53 (s, 1H), 2.00 (s, 9H).

^{19}F NMR (377 MHz, MeOD): δ -76.82, -103.31 (tt, J = 8.6, 5.4 Hz).

^{13}C{1H} NMR (101 MHz, MeOD): δ 168.7 (d, $^1J_{C,F}$ = 258, C-F), 167.4, 166.9, 146.1, 138.0, 133.6 (d, $^3J_{C,F}$ = 9.9. 2×CH), 133.4, 130.2 (2×CH), 129.6 (d, $^4J_{C,F}$ = 2.9, C), 129.1 (2×CH), 118.2 (d, $^2J_{C,F}$ = 22.7, 2×CH), 103.6 (C<u>C</u>H(OH)$_2$), 78.8, 28.9 (3×CH_3).

HRMS analysis corresponds to the parent aldehyde. HRMS (ESI): m/z calcd. for $C_{20}H_{19}ON_3F$ [M$^+$] 336.1506, found 336.1505.

3-(4-Acetylphenyl)-1-(*tert*-butyl)-5-(4-fluorophenyl)-1,2,4-triazin-1-ium 2,2,2-trifluoroacetate (ArTrz⁺2n)

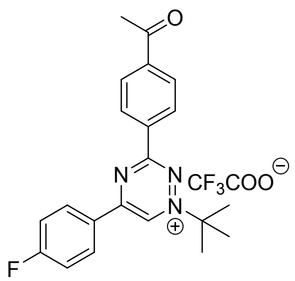

Purification: MeCN/H_2O (+ 0.1% TFA) gradient 15:85 → 60:40 for 30 min. Product detection at λ = 282 and 342 nm. Complete spectral characterization required prolonged incubation in MeOD at rt for

approximately 35 h leading to equilibrated mixture of the expected **ArTrz⁺2n** and its corresponding hydrate in ratio 3:2.

Yield: 30.0 mg (0.065 mmol, 55%) of a bright orange amorphous solid.

^{1}H NMR (400 MHz, MeOD): δ 10.19 (s, 1H), 8.82 – 8.72 (m, 4H), 8.32 – 8.26 (m, 2H), 7.57 – 7.49 (m, 2H), 2.72 (s, 3H), 2.02 (s, 9H).

^{19}F NMR (376 MHz, MeOD): δ -77.05, -102.82 – -103.02 (m).

The hydration turned out to be reversible, which was subsequently confirmed by repeated NMR analyses in DMSO-d_6 providing clear spectra of the parent **ArTrz$^+$2n**.

^{1}H NMR (400 MHz, DMSO-d_6): δ 10.27 (s, 1H), 8.88 – 8.80 (m, 2H), 8.77 – 8.70 (m, 2H), 8.30 – 8.20 (m, 2H), 7.72 – 7.62 (m, 2H), 2.71 (s, 3H), 1.92 (s, 9H).

^{19}F NMR (376 MHz, DMSO-d_6): δ -73.47, -102.08 (dt, J = 14.3, 7.2 Hz).

^{13}C{1H} NMR (101 MHz, DMSO-d_6): δ 197.7, 167.8, 165.2, 164.0, 140.5, 138.41, 135.6, 132.99 (2×CH), 132.89 (2×CH), 129.1 (d, $^4J_{C,F}$ = 3.7, C), 128.2 (d, $^4J_{C,F}$ = 2.6, C), 117.0 (d, $^2J_{C,F}$ = 22.3, 2×CH), 76.8, 28.1 (3×CH_3), 27.1 (COO$\underline{C}H_3$).

HRMS (ESI): m/z calcd. for $C_{21}H_{21}ON_3F$ [M^+] 350.1663, found 350.1664.

1-(*tert*-Butyl)-5-(4-fluorophenyl)-3-(4-(methoxycarbonyl)phenyl)-1,2,4-triazin-1-ium 2,2,2-trifluoroacetate (ArTrz$^+$2o)

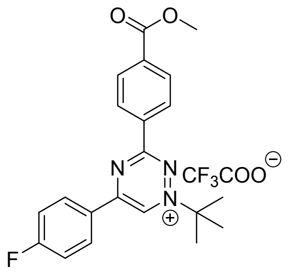

Purification: MeCN/H_2O (+ 0.1% TFA) gradient 15:85 → 65:35 for 30 min. Product detection at λ = 342 nm.

Yield: 35.0 mg (0.073 mmol, 62%) of a bright yellow amorphous solid.

Comment: The synthesis of **ArTrz$^+$2o** was also successfully up-scaled using general procedure A. **SMeTrz$^+$1** (1.170 mmol) afforded 400 mg (0.834 mmol, 71%) of a bright yellow amorphous solid.

^{1}H NMR (401 MHz, MeOD): δ 10.19 (s, 1H), 8.81 – 8.72 (m, 4H), 8.35 – 8.29 (m, 2H), 7.56 – 7.48 (m, 2H), 3.99 (s, 3H), 2.02 (s, 9H).

^{19}F NMR (377 MHz, MeOD): δ -76.45, -102.91 (tt, J = 8.2, 5.2 Hz).

$^{13}C\{1H\}$ NMR (101 MHz, MeOD): δ 168.9 (d, $^{1}J_{C,F}$ = 258, C-F), 167.3, 167.2, 167.0, 138.6, 137.2, 136.2, 133.8 (d, $^{3}J_{C,F}$ = 9.9, 2×CH), 131.4 (2×CH), 130.3 (2×CH), 129.5 (d, $^{4}J_{C,F}$ = 2.6, C), 118.3 (d, $^{2}J_{C,F}$ = 22.7, 2×CH), 79.0, 53.1 (COO$\underline{C}H_3$), 28.9 (3×CH_3).

HRMS (ESI): m/z calcd. for $C_{21}H_{21}O_2N_3F$ [M^+] 366.1612, found 366.1612.

1-(*tert*-Butyl)-3-(4-(dimethylamino)phenyl)-5-(4-fluorophenyl)-1,2,4-triazin-1-ium 2,2,2-trifluoroacetate (ArTrz⁺2p)

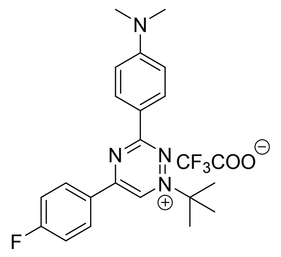

Purification: MeCN/H_2O (+ 0.1% TFA) gradient 15:85 → 65:35 for 30 min. Product detection at λ = 407 nm. Re-purified by column chromatography on silica gel in HILIC mode (puri*Flash*® from *InterChim,* SiO_2 (40 g) column). H_2O/MeCN (+ 0.1% TFA) gradient 0:100 → 5:95 and flow rate 35 mL/min for 20 min and lyophilized.

Yield: 9.5 mg (0.020 mmol, 18%) of a deep brown amorphous solid.

^{1}H NMR (401 MHz, CD_3CN): δ 9.34 (s, 1H), 8.56 – 8.49 (m, 2H), 8.44 – 8.38 (m, 2H), 7.50 – 7.42 (m, 2H), 6.92 – 6.86 (m, 2H), 3.14 (s, 6H), 1.88 (s, 9H).

^{19}F NMR (377 MHz, CD_3CN): δ -76.60, -104.07 (tt, *J* = 8.6, 5.2 Hz).

$^{13}C\{1H\}$ NMR (101 MHz, CD_3CN): δ 168.4 (d, $^{1}J_{C,F}$ = 254, C-F), 167.8, 165.4, 155.8, 133.7, 132.8 (d, $^{3}J_{C,F}$ = 9.9, 2×CH), 132.1 (2×CH), 129.3 (d, $^{4}J_{C,F}$ = 3.0, C), 118.3 (d, $^{2}J_{C,F}$ = 22.7, 2×CH), 112.9 (2×CH), 78.0, 40.3 (N($\underline{C}H_3$)$_2$), 28.7 (3×CH_3). Signal of one quart. carbon was not found.

HRMS (ESI): m/z calcd. for $C_{21}H_{24}N_4F$ [M^+] 351.1979, found 351.1981.

(*E*)-1-(*tert*-Butyl)-5-(4-fluorophenyl)-3-styryl-1,2,4-triazin-1-ium1,2,4-triazin-1-ium 2,2,2-trifluoroacetate (ArTrz⁺2q)

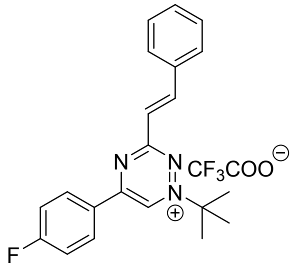

After stirring under argon at 60 °C for 4 h, the mixture was cooled to rt and additional boronic acid (2.0 equiv., 0.234 mmol) was added. Stirring under argon then continued at 90 °C for 4 more hours.

Purification: MeCN/H_2O (+ 0.1% TFA) gradient 15:85 → 70:30 for 30 min. Product detection at λ = 335 nm. The contaminated crude product was re-purified by column chromatography on silica gel (MeOH/DCM gradient 2:98 → 15:85 + 0.1% TFA).

Yield: 50.0 mg (0.112 mmol, 96%) of a bright yellow amorphous solid.

^{1}H NMR (401 MHz, MeOD): δ 9.96 (s, 1H), 8.73 – 8.66 (m, 2H), 8.50 (d, J = 15.9 Hz, 1H), 7.89 – 7.83 (m, 2H), 7.56 (d, J = 15.9 Hz, 1H), 7.53 – 7.46 (m, 5H), 1.94 (s, 9H).

^{19}F NMR (377 MHz, MeOD): δ -77.07, -103.45 – -104.21 (m).

^{13}C{1H} NMR (101 MHz, MeOD): δ 168.6 (d, $^1J_{C,F}$ = 258, C-F), 168.4, 166.1, 147.2, 137.3, 135.9, 133.4 (d, $^3J_{C,F}$ = 9.9, 2×CH), 132.3, 130.2 (2×CH), 129.8 (2×CH), 129.6 (d, $^4J_{C,F}$ = 2.6, C), 122.2, 118.1 (d, $^2J_{C,F}$ = 22.7, 2×CH), 78.2, 28.8 (3×CH_3).

HRMS (ESI): m/z calcd. for $C_{21}H_{21}N_3F$ [M^+] 334.1714, found 334.1714.

1-(*tert*-Butyl)-5-(4-fluorophenyl)-3-(thiophen-3-yl)-1,2,4-triazin-1-ium 2,2,2-trifluoroacetate (ArTrz$^+$2r)

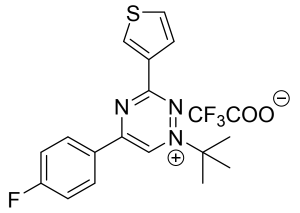

Purification: MeCN/H_2O (+ 0.1% TFA) gradient 15:85 → 75:25 for 30 min. Product detection at λ = 340 nm.

Yield: 32.0 mg (0.075 mmol, 64%) of a light yellow amorphous solid.

^{1}H NMR (401 MHz, CD_3CN): δ 9.74 (s, 1H), 8.85 (dd, J = 3.0, 1.3 Hz, 1H), 8.67 – 8.60 (m, 2H), 8.03 (dd, J = 5.2, 1.3 Hz, 1H), 7.72 (dd, J = 5.2, 3.0 Hz, 1H), 7.51 – 7.44 (m, 2H), 1.92 (s, 9H).

^{19}F NMR (377 MHz, CD_3CN): δ -76.36, -103.03 (tt, J = 8.6, 5.4 Hz).

^{13}C{1H} NMR (101 MHz, MeOD): δ 168.2 (d, $^1J_{C,F}$ = 257, C-F), 166.5, 164.2, 136.8, 136.3, 135.5, 133.4 (d, $^3J_{C,F}$ = 9.9. 2×CH), 130.0, 128.8 (d, $^4J_{C,F}$ = 2.9, C), 127.7, 118.1 (d, $^2J_{C,F}$ = 22.7, 2×CH), 78.7, 28.7 (3×CH_3).

HRMS (ESI): m/z calcd. for $C_{17}H_{17}N_3FS$ [M^+] 314.1121, found 314.1123.

1-(*tert*-Butyl)-5-(4-fluorophenyl)-3-(4-(methylsulfonyl)phenyl)-1,2,4-triazin-1-ium 2,2,2-trifluoroacetate (ArTrz$^+$2s)

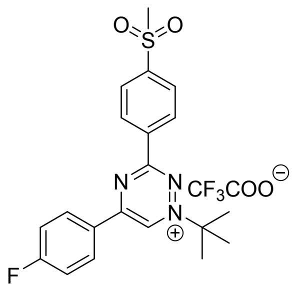

Purification: MeCN/H_2O (+ 0.1% TFA) gradient 10:90 → 65:35 for 30 min. Product detection at λ = 345 nm. Re-purified by column chromatography on silica gel in HILIC mode (puri*Flash*® from *InterChim,* SiO_2 (40 g) column). H_2O/MeCN (+ 0.1% TFA) gradient 0:100 → 5:95 and flow rate 35 mL/min for 20 min and lyophilized.

Yield: 48.0 mg (0.096 mmol, 82%) of a white amorphous solid.

^{1}H NMR (401 MHz, CD_3CN): δ 9.83 (s, 1H), 8.85 – 8.78 (m, 2H), 8.71 – 8.64 (m, 2H), 8.24 – 8.17 (m, 2H), 7.56 – 7.47 (m, 2H), 3.18 (s, 3H), 1.96 (s, 9H).

^{19}F NMR (377 MHz, CD_3CN): δ -76.19, -102.05 (tt, *J* = 8.2, 5.0 Hz).

^{13}C{1H} NMR (101 MHz, CD_3CN): δ 168.5 (d, $^1J_{C,F}$ = 258, C-F), 166.8, 166.3, 146.6, 138.1, 137.3, 133.7 (d, $^3J_{C,F}$ = 10.3. 2×CH), 131.1 (2×CH), 129.3 (2×CH), 128.6 (d, $^4J_{C,F}$ = 2.9, C), 118.3 (d, $^2J_{C,F}$ = 22.7, 2×CH), 79.4, 44.3 (CH_3), 28.8 (3×CH_3).

HRMS (ESI): m/z calcd. for $C_{20}H_{21}O_2N_3FS$ [M^+] 386.1333, found 386.1331.

1-(*tert*-Butyl)-5-(4-fluorophenyl)-3-(4-morpholinophenyl)-1,2,4-triazin-1-ium 2,2,2-trifluoroacetate (ArTrz$^+$2t)

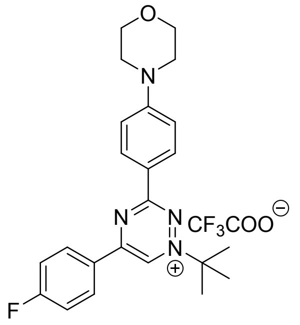

Purification: MeCN/H_2O (+ 0.1% TFA) gradient 15:85 → 75:25 for 30 min. Product detection at λ = 304 and 387 nm. Re-purified by column chromatography on silica gel in HILIC mode (puri*Flash*® from *InterChim,* SiO_2 (40 g) column). H_2O/MeCN (+ 0.1% TFA) gradient 0:100 → 5:95 and flow rate 35 mL/min for 20 min and lyophilized.

Yield: 30.0 mg (0.059 mmol, 50%) of a dark brown amorphous solid.

^{1}H NMR (401 MHz, CD_3CN): δ 9.48 (s, 1H), 8.62 – 8.55 (m, 2H), 8.50 – 8.45 (m, 2H), 7.53 – 7.46 (m, 2H), 7.15 – 7.10 (m, 2H), 3.86 – 3.80 (m, 4H), 3.48 – 3.42 (m, 4H), 1.93 (s, 9H).

^{19}F NMR (377 MHz, CD_3CN): δ -76.60, -103.71 (tt, *J* = 8.6, 5.2 Hz).

^{13}C{1H} NMR (101 MHz, CD_3CN): δ 168.0 (d, $^{1}J_{C,F}$ = 257, C-F), 167.5, 165.7, 156.5, 134.6, 133.0 (d, $^{3}J_{C,F}$ = 9.9. 2×CH), 132.0 (2×CH), 129.2 (d, $^{4}J_{C,F}$ = 2.6, C), 121.1, 118.0 (d, $^{2}J_{C,F}$ = 22.7, 2×CH), 114.8 (2×CH), 78.2, 67.0 (2×CH_2), 47.8 (2×CH_2), 28.8 (3×CH_3).

HRMS (ESI): m/z calcd. for $C_{23}H_{26}ON_4F$ [M^+] 393.2090, found 393.2087.

Synthesis of model **ArTrz$^+$2u** *via* Liebeskind-Srogl cross coupling reaction with stannane

1-(*tert*-Butyl)-3-(3,4-dihydro-2*H*-pyran-6-yl)-5-(4-fluorophenyl)-1,2,4-triazin-1-ium 2,2,2-trifluoroacetate (ArTrz$^+$2u)

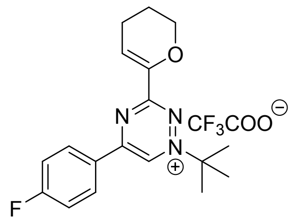

Trz$^+$1[2] (50.0 mg, 0.117 mmol), tributy(5,6-dihydro-*4H*-2-yl)stannane (2.0 equiv., 78.0 µL, 0.234 mmol), copper(I) thiophene-2-carboxylate (2.2 equiv., 49.1 mg, 0.257 mmol) and $Pd(PPh_3)_4$ (10 mol%, 13.5 mg, 0.012 mmol) were placed in an argon flushed flask and anhydrous 1,4-dioxane (6.0 mL) was added. The mixture was stirred under argon at 60 °C (heating mantle) for 4 h. After cooling to rt, the mixture was diluted with DCM (50 mL) and washed with sat. aq. $NaHCO_3$ (50 mL) and extracted with DCM (2×20 mL). The combined organic extracts were dried over Na_2SO_4, filtered, and concentrated under reduced pressure. It was then re-dissolved in (9:1) MeCN/H_2O (+ 0.1% TFA) (15 mL), filtered through a short pad of sand/C18® (Santiago; 230-400 MESH) and washed with (9:1) MeCN/H_2O (+ 0.1% TFA) (3×15 mL). The filtrate was concentrated under reduced pressure and the crude product was purified by preparative HPLC (Arion Plus (21.2 × 250 mm, 5 µm) column): MeCN/H_2O (+ 0.1% TFA) gradient 5:95 → 75:25 and flow rate 16 mL/min for 30 min and lyophilized. Product detection at λ = 325 nm.

Yield: 27.0 mg (0.063 mmol, 54%) of a bright orange amorphous solid.

^{1}H NMR (401 MHz, CD_3CN): δ 9.77 (s, 1H), 8.57 – 8.50 (m, 2H), 7.48 – 7.40 (m, 2H), 6.95 (t, *J* = 4.4 Hz, 1H), 4.31 – 4.26 (m, 2H), 2.44 (td, *J* = 6.4, 4.3 Hz, 2H), 2.05 – 1.97 (m, 2H), 1.86 (s, 9H).

^{19}F NMR (377 MHz, CD_3CN): δ -75.95, -103.31 (tt, *J* = 8.6, 5.2 Hz).

^{13}C{1H} NMR (101 MHz, CD_3CN): δ 168.1 (d, $^{1}J_{C,F}$ = 257, C-F), 165.5, 163.8, 147.6, 137.7, 133.2 (d, $^{3}J_{C,F}$ = 9.9. 2×CH), 128.8 (d, $^{4}J_{C,F}$ = 2.9, C), 118.0 (d, $^{2}J_{C,F}$ = 22.7, 2×CH), 117.0, 78.5, 68.0, 28.7 (3×CH_3), 22.2, 22.1.

HRMS (ESI): m/z calcd. for $C_{18}H_{21}ON_3F$ [M^+] 314.1663, found 314.1662.

Synthesis of reduced C5-monosubstituted **red HTrz⁺3**

1-(*tert*-Butyl)-5-(4-fluorophenyl)-2,5-dihydro-1,2,4-triazin-1-ium 2,2,2-trifluoroacetate (red HTrz⁺3)

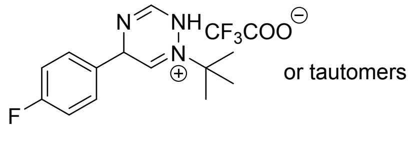

red HTrz⁺3

SMeTrz⁺1[2] (35.0 mg, 0.082 mmol) and Pd(II)Cl_2 (20 mol%, 2.90 mg, 0.016 mmol) were placed in an argon flushed flask and anhydrous 1,4-dioxane (2.7 mL) was added. Then, TES (6.0 equiv., 78 µL, 0.491 mmol) was added dropwise at rt and the mixture was stirred under argon at 55 °C (heating mantle) for 17 h.

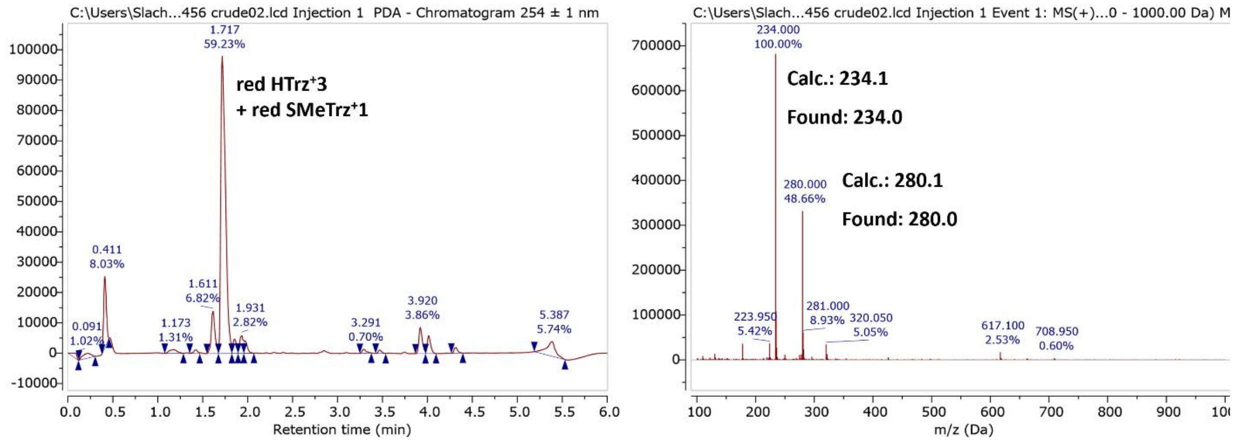

Figure S2: Exported HPLC chromatogram of crude **red HTrz⁺3** (λ = 254 nm) and the corresponding MS spectrum. Solvent system: solvent A: H_2O + 0.05% HCOOH; solvent B: MeCN + 0.05% HCOOH. Gradient: 5% B → 95% B (4 min), then 95% B (0.5 min), then 95% B → 5% B (0.5 min) and 5% B (1 min).

After cooling to rt, the crude was filtered through Celite® 545 and washed with AcOEt (2×10 mL) and MeCN (2×10 mL). Combined organic fractions were concentrated under reduced pressure and the crude product was purified by preparative HPLC (Arion Plus (21.2 × 250 mm, 5 µm) column): MeCN/H_2O (+ 0.1% TFA) gradient 5:95 → 65:35 and flow rate 16 mL/min for 20 min and lyophilized. Product detection at λ = 266 nm. Yield: 3.0 mg (0.009 mmol, 11%) of a white amorphous solid contaminated with small impurities, presumably, from residual over-reduced **SMeTrz⁺1** analogue. The product **red HTrz⁺3** formed possibly as a mixture of tautomers.

^{1}H NMR (401 MHz, CD_3CN): δ 7.69 – 7.63 (m, 2H), 7.47 – 7.40 (br m, 2H), 7.26 – 7.14 (m, 4H), 1.62 (s, 9H).

^{19}F NMR (377 MHz, CD_3CN): δ -76.24, -114.87 (tt, J = 8.9, 5.4 Hz).

HRMS (ESI): m/z calcd. for $C_{13}H_{17}N_3F$ [M^+] 234.1401, found 234.1401.

Synthesis of aromatic C5-monosubstituted **arom HTrz⁺4**

1-(*tert*-Butyl)-5-(4-fluorophenyl)-1,2,4-triazin-1-ium 2,2,2-trifluoroacetate (HTrz⁺3)

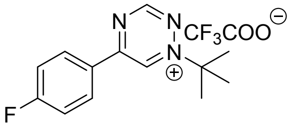

HTrz⁺3

SMeTrz⁺1[2] (51.3 mg, 0.120 mmol) and Pd(II)Cl_2 (20 mol%, 4.30 mg, 0.024 mmol) were placed in an argon flushed flask and anhydrous 1,4-dioxane (4.0 mL) was added. Then, TES (6.0 equiv., 115 µL, 0.720 mmol) was added dropwise at rt and the mixture was stirred under argon at 55 °C (heating mantle) for 16 h. After cooling to rt, Mn(IV)O_2 (10.0 equiv, 104 mg, 1.2 mmol) was added in one portion and the mixture was sonicated at rt (sonicator) for 3 h. Finally, the crude was filtered through Celite® 545 and washed with AcOEt (2×10 mL) and MeCN (2×10 mL). Combined organic fractions were concentrated under reduced pressure and the crude product was purified by preparative HPLC (Arion Plus (21.2 × 250 mm, 5 µm) column): MeCN/H_2O (+ 0.1% TFA) gradient 5:95 → 60:40 and flow rate 16 mL/min for 30 min and lyophilized. Product detection at λ = 339 nm.

Yield: 9.0 mg (0.026 mmol, 22%) of a white amorphous solid.

^{1}H NMR (401 MHz, CD_3CN): δ 10.08 (br d, *J* = 1.7 Hz, 1H), 9.92 (br d, *J* = 1.6 Hz, 1H), 8.58 (dd, *J* = 9.0, 5.3 Hz, 2H), 7.50 – 7.40 (m, 2H), 1.88 (s, 9H).

^{19}F NMR (377 MHz, CD_3CN): δ -75.60, -102.80 (td, *J* = 8.6, 4.1 Hz).

^{13}C{1H} NMR (101 MHz, CD_3CN): δ 169.5, 166.8, 160.1, 140.9, 133.6 (d, $^3J_{C,F}$ = 10.2. 2×CH), 127.6, 118.0 (2×CH), 78.5, 28.6 (3×CH_3). Signal of one tert. carbon was not found.

HRMS (ESI): m/z calcd. for $C_{13}H_{15}N_3F$ [M^+] 232.1244, found 232.1244.

Synthesis of fluorogenic triaziniums

Synthesis of various 1-(*tert*-butyl)-5-(4-methoxyphenyl)-**Trz⁺s**

1-(*tert*-Butyl)-5-(4-methoxyphenyl)-3-(methylthio)-1,2,4-triazin-1-ium 2,2,2-trifluoroacetate (SMeTrz⁺5)

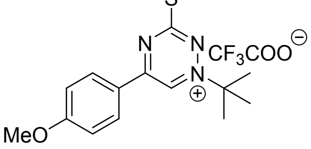

SMeTrz2[1] (1.0 equiv., 0.500 mmol, 117.0 mg) was dissolved in dry DCM (5.0 mL) under argon and cooled to 0 °C. Isobutene gas was condensed in a Schlenk tube at -78 °C (dry ice/acetone bath). Subsequently, an excess of liquid isobutene (1.0 mL) was added followed by dropwise addition of trifloromethanesulfonic acid (1.5 equiv., 0.750 mmol, 66.0 µL) at 0 °C and the mixture was stirred at rt for 1 h. The crude product

was diluted with DCM (50 mL) and washed with sat. aq. $NaHCO_3$ (50 mL) and extracted with DCM (2×20 mL). The combined organic extracts were dried over Na_2SO_4, filtered, and concentrated under reduced pressure. The crude mixture was purified by RP flash chromatography (RediSep Gold C18Aq 50 g column). MeCN/H_2O (+ 0.1% TFA) gradient (flow rate 40 mL/min) 0:100 → 50:50 for 20 min. Product detection at λ = 390 nm, lyophilization.

Yield: 120.5 mg (0.299 mmol, 60%) of a yellow amorphous solid.

^1H NMR (400 MHz, CD_3CN): δ 9.46 (s, 1H), 8.45 – 8.38 (m, 2H), 7.23 – 7.16 (m, 2H), 3.96 (s, 3H), 2.74 (s, 3H), 1.83 (s, 9H).

^{19}F NMR (377 MHz, CD_3CN): δ -76.06.

$^{13}C\{1H\}$ NMR (101 MHz, CD_3CN): δ 179.0, 167.3, 164.6, 134.7, 133.0 (2×CH), 124.1, 116.5 (2×CH), 78.2, 56.9 (O$\underline{C}H_3$), 28.6 (3×CH_3), 14.8 (S$\underline{C}H_3$).

HRMS (ESI): m/z calcd. for $C_{15}H_{20}ON_3S$ [M^+] 290.1321, found 290.1324.

***tert*-Butyl (4-(5-(4-methoxyphenyl)-1,2,4-triazin-3-yl)benzyl)carbamate (ArTrz)**

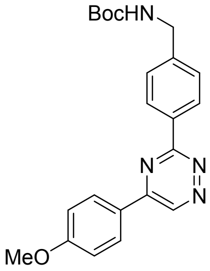

SMeTrz2[1] (1.0 equiv., 0.900 mmol, 210.0 mg), (4-(((tert-butoxycarbonyl)amino)methyl)phenyl)boronic acid (2.0 equiv., 1.800 mmol, 452.0 mg), copper(I) thiophene-2-carboxylate (2.2 equiv., 1.980 mmol, 377.5 mg) and $Pd(PPh_3)_4$ (10 mol%, 0.090 mmol, 104 mg) were placed in an argon flushed flask and anhydrous 1,4-dioxane (3.6 mL) was added. The mixture was stirred under argon at 95 °C for 2 h. After cooling to rt, the mixture was diluted with DCM (100 mL) and washed subsequently with 2 M aq. NaOH (100 mL) and sat. aq. NH_4Cl (100 mL). The organic phase was dried over Na_2SO_4, filtered, and concentrated under reduced pressure. The crude product was purified by silica gel column chromatography (EA/DCM gradient 10:90 → 20:80).

Yield: 250.0 mg (0.637 mmol, 71%) of a yellow amorphous solid.

^1H NMR (401 MHz, $CDCl_3$): δ 9.51 (s, 1H), 8.65 – 8.52 (m, 2H), 8.32 – 8.20 (m, 2H), 7.54 – 7.40 (m, 2H), 7.14 – 7.02 (m, 2H), 4.98 (br s, 1H), 4.42 (d, *J* = 6.2 Hz, 2H), 3.91 (s, 3H), 1.48 (s, 9H).

$^{13}C\{1H\}$ NMR (101 MHz, $CDCl_3$): δ 167.0, 163.4, 154.7, 147.9, 143.8, 142.8, 134.5, 129.5 (2×CH), 128.7 (2×CH), 127.8 (2×CH), 126.0, 114.9 (2×CH), 80.6, 55.6, 44.6, 28.5 (3×CH_3).

HRMS (ESI): m/z calcd. for $C_{22}H_{25}O_3N_4$ $[M+H]^+$ 393.1921, found 393.1921.

3-(4-(ammoniomethyl)phenyl)-1-(tert-butyl)-5-(4-methoxyphenyl)-1,2,4-triazin-1-ium 2,2,2-trifluoroacetate (ArTrz⁺4)

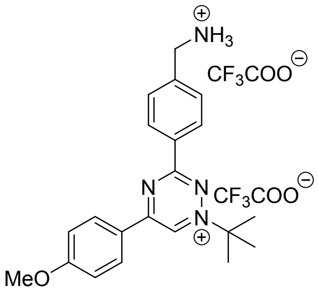

ArTrz (1.0 equiv., 0.322 mmol, 126.4 mg) was dissolved in dry DCM (5.0 mL) under argon and cooled to 0 °C. Isobutene gas was condensed in a Schlenk tube at -78 °C (dry ice/acetone bath). Subsequently, an excess of liquid isobutene (700 µL) was added followed by dropwise addition of trifloromethanesulfonic acid (3.3 equiv., 1.063 mmol, 94.0 µL) at 0 °C and the mixture was stirred at rt for 2 h. The crude product was diluted with AcOEt (50 mL) and washed with sat. aq. $NaHCO_3$ (50 mL) and extracted with AcOEt (2×20 mL). The combined organic extracts were dried over Na_2SO_4, filtered, and concentrated under reduced pressure. The crude mixture was purified by RP flash chromatography (RediSep Gold C18Aq 50 g column). MeCN/H_2O (+ 0.1% TFA) gradient (flow rate 20 mL/min) 0:100 → 35:65 for 20 min. Product detection at λ = 392 nm, lyophilization.

Yield: 104.0 mg (0.180 mmol, 56%) of a yellow amorphous solid.

^{1}H NMR (400 MHz, CD_3CN/D_2O): δ 10.31 (s, 1H), 9.23 – 9.16 (m, 2H), 9.15 – 9.09 (m, 2H), 8.29 (dd, J = 8.4, 1.8 Hz, 2H), 7.88 – 7.78 (m, 2H), 4.83 (s, 2H), 4.53 (s, 3H), 2.48 (s, 9H).

^{19}F NMR (377 MHz, CD_3CN/D2O): δ -75.09.

^{13}C{1H} NMR (101 MHz, CD_3CN/D_2O): δ 167.1, 166.8, 166.3, 139.8, 136.5, 133.4, 132.8 (2×CH), 130.8 (2×CH), 130.5 (2×CH), 124.5, 116.5 (2×CH), 78.4, 56.9 (O$\underline{C}H_3$), 43.4 (C$\underline{C}H_2NH_3^+$), 28.7 (3×CH_3).

HRMS (ESI): m/z calcd. for $C_{21}H_{25}ON_4$ [M$^+$] 349.2022, found 349.2025.

Synthesis of coumarin-boronic acids

General procedure B: Bromo phenyl coumarin **BrPhCoum1**[3] or **BrPhCoum2**[3] (1.0 equiv., 0.500 mmol), benzene-1,4-diboronic acid or benzene-1,3-diboronic acid (3.0 equiv., 1.500 mmol; 248.5 mg), K_2CO_3 (2.0 equiv. 1.000 mmol; 138.1 mg) and $PdCl_2$(dppf).DCM (10 mol%, 0.050 mmol; 40.8 mg) were suspended in a 1,4-dioxane/H_2O mixture (6 + 2 mL) and stirred at 100 °C for 1-2 hour. After cooling to rt, the mixture was concentrated under reduced pressure. The crude product was purified (shown for each derivative separately).

(3-(7-(Azetidin-1-yl)-4-methyl-2-oxo-2*H*-chromen-3-yl)phenyl)boronic acid (Coum-BA1)

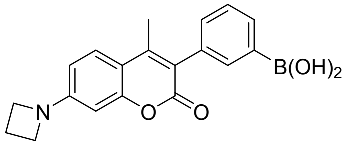

Bromo phenyl coumarin **BrPhCoum1**[3] (0.250 mmol) was used and a purification performed as follows: Silica gel column chromatography MeOH/DCM gradient 1:99 → 4:98. The obtained mixture was additionally purified by RP flash chromatography (RediSep Gold C18Aq 50 g column). MeCN/H_2O (+ 0.1% TFA) gradient (flow rate 25 mL/min) 30:70 → 95:5 for 20 min. Product detection at λ = 367 nm, lyophilization. Yield: 28.0 mg (0.084 mmol, 34%) of a light yellow amorphous solid.

^{1}H NMR (500 MHz DMSO-*d*$_6$): δ 7.78 (dt, *J* = 7.3, 1.3 Hz, 1H), 7.67 (t, *J* = 1.7 Hz, 1H), 7.59 (d, *J* = 8.8 Hz, 1H), 7.39 (t, *J* = 7.5 Hz, 1H), 7.30 (dt, *J* = 7.6, 1.6 Hz, 1H), 6.42 (dd, *J* = 8.8, 2.3 Hz, 1H), 6.28 (d, *J* = 2.3 Hz, 1H), 3.96 (t, *J* = 7.3 Hz, 4H), 2.42 – 2.31 (m, 2H), 2.17 (s, 3H).

^{13}C{1H} NMR (126 MHz, DMSO-*d*$_6$): δ 160.5, 154.1, 153.6, 148.5, 136.1, 134.2, 133.2, 132.1, 127.1, 126.6, 120.8, 109.8, 108.0, 96.0, 51.5 (2×CH_2), 16.3 (CH_3)., 16.0 (CH_2). Signal of one quat. carbon was not found.

HRMS (ESI): m/z calcd. for $C_{19}H_{19}O_4NB$ $[M+H]^+$ 336.1402, found 336.1401.

(3-(7-(diethylamino)-4-methyl-2-oxo-2*H*-chromen-3-yl)phenyl)boronic acid (Coum-BA2)

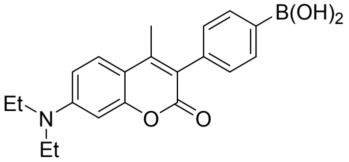

Silica gel column chromatography MeOH/DCM gradient 2:98 → 6:94. The obtained mixture was additionally purified by RP flash chromatography (RediSep Gold C18Aq 50 g column). MeCN/H_2O (+ 0.1% TFA) gradient (flow rate 25 mL/min) 30:70 → 90:10 for 20 min. Product detection at λ = 383 nm, lyophilization. Yield: 85.5 mg (0.243 mmol, 49%) of a yellow amorphous solid.

^{1}H NMR (401 MHz, DMSO-*d*$_6$): δ 7.86 – 7.79 (m, 2H), 7.57 (d, *J* = 9.0 Hz, 1H), 7.26 – 7.20 (m, 2H), 6.74 (dd, *J* = 9.2, 2.6 Hz, 1H), 6.56 (d, *J* = 2.7 Hz, 1H), 3.44 (q, *J* = 7.1 Hz, 4H), 2.17 (s, 3H), 1.13 (t, *J* = 7.0 Hz, 6H).

^{13}C{1H} NMR (101 MHz, DMSO-*d*$_6$): δ 160.5, 154.6, 149.9, 148.3, 137.0, 133.7 (2×CH), 129.5 (2×CH), 126.8, 119.9, 108.9, 108.7, 96.7, 44.1 (2×CH_2), 16.1 (CH_3)., 12.3 (2×CH_3). Signal of one quat. carbon was not found.

HRMS (ESI): m/z calcd. for $C_{20}H_{21}O_4NB$ $[M-H]^+$ 350.1570, found 350.1569.

Synthesis of fluorogenic Trz$^+$-coumarin compounds

Additional general note: We have observed partial decomposition of fluorogenic triazinium-comarin compounds upon prolonged exposure to air (e.g. during solvent removal by a flow of air). Therefore, some compounds were re-purified by preparative HPLC (Supelco (25 cm × 10 mm, 5 µm) column). MeCN/H_2O

(+ 0.1% TFA) gradient (flow rate 5 mL/min), for 30 min as indicated for each derivative separately. Product detection is shown for each compound below. The products were finally lyophilized.

1-(*tert*-Butyl)-3-(4-((7-(diethylamino)-2-oxo-2*H*-chromene-3-carboxamido)methyl)phenyl)-5-(4-methoxyphenyl)-1,2,4-triazin-1-ium 2,2,2-trifluoroacetate (Trz⁺Coum6)

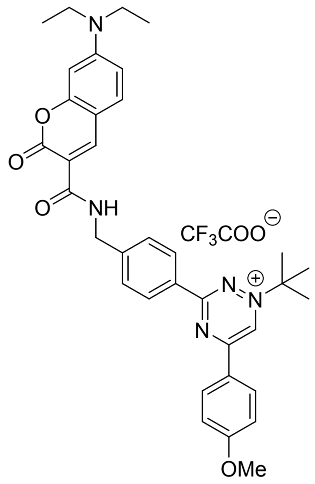

ArTrz⁺4 (1.0 equiv., 28.2 mg, 0.049 mmol) and commercial 7-(diethylamino)coumarin-3-carboxylic acid NHS ester (1.1 equiv., 19.3 mg, 0.054 mmol) were dissolved in dry MeCN (1.30 mL) and the solution was cooled to 0 °C. DIPEA (5.0 equiv., 43.0 µL, 0.245 mmol) was then added dropwise. After 1 h of stirring at rt, the reaction was diluted with 1:1 MeCN/H_2O (3.70 mL) mixture, acidified with TFA (7.0 equiv., 26.0 µL, 0.343 mmol) and the crude product was purified by preparative HPLC (Arion Plus (21.2 × 250 mm, 5 µm) column). MeCN/H_2O (+ 0.1% TFA) gradient (flow rate 15 mL/min) 20:80 → 90:10 for 25 min. Product detection at λ = 290 and 422 nm, lyophilization.

Yield: 20.0 mg (0.028 mmol, 58%) of a dark yellow solid.

^{1}H NMR (400 MHz, CD_3CN): δ 9.62 (s, 1H), 9.20 (t, *J* = 6.0 Hz, 1H), 8.63 (d, *J* = 0.9 Hz, 1H), 8.58 – 8.50 (m, 4H), 7.65 – 7.57 (m, 2H), 7.51 (d, *J* = 9.0 Hz, 1H), 7.27 – 7.19 (m, 2H), 6.76 (dd, *J* = 9.0, 2.4 Hz, 1H), 6.54 (dd, *J* = 2.4, 0.7 Hz, 1H), 4.69 (d, *J* = 6.1 Hz, 2H), 3.97 (s, 3H), 3.47 (q, *J* = 7.0 Hz, 4H), 1.92 (s, 9H), 1.19 (t, *J* = 7.1 Hz, 6H).

^{19}F NMR (376 MHz, CD_3CN): δ -76.5.

^{13}C{1H} NMR (101 MHz, CD_3CN): δ 167.2, 166.1, 164.2, 163.5, 158.8, 153.9, 148.9, 147.6, 136.3, 132.8 (2×CH), 132.3, 131.8, 130.2 (2×CH), 129.3 (2×CH), 124.6, 116.5 (2×CH), 111.2, 110.8, 109.0, 97.0, 78.3, 56.9 (O$\underline{C}H_3$), 45.7 (2×CH_2), 43.7 (CONH$\underline{C}H_2$), 28.8 (3×CH_3), 12.7 (2×CH_3). Signal of one quat. carbon was not found.

HRMS (ESI): m/z calcd. for $C_{35}H_{38}O_4N_5$ [M^+] 595.2918, found 595.2917.

3-(3-(7-(Azetidin-1-yl)-4-methyl-2-oxo-2*H*-chromen-3-yl)phenyl)-1-(*tert*-butyl)-5-(4-methoxyphenyl)-1,2,4-triazin-1-ium 2,2,2-trifluoroacetate (Trz⁺Coum7)

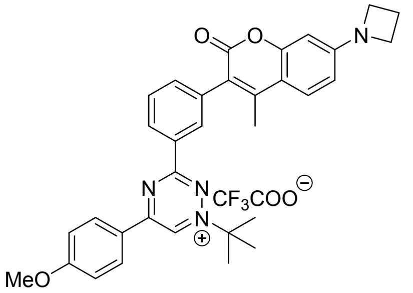

SMeTrz⁺5 (12.0 mg, 0.030 mmol), boronic acid **Coum-BA1** (1.3 equiv., 13.0 mg, 0.039 mmol), copper(I) thiophene-2-carboxylate (2.2 equiv., 12.6 mg, 0.066 mmol) and $Pd(PPh_3)_4$ (10 mol%, 3.5 mg, 0.003 mmol) were placed in an argon flushed flask and anhydrous 1,4-dioxane (600 µL) was added. Anhydrous DMF (100 µL) was then added for better solubilization of the reagents. The mixture was stirred under argon at 60 °C (heating mantle) for 6 h. After cooling to rt, the mixture was diluted with DCM (20 mL) and washed with sat. aq. $NaHCO_3$ (20 mL) and extracted with DCM (2×20 mL). The combined organic extracts were dried over Na_2SO_4, filtered, and concentrated under reduced pressure. The mixture was then re-dissolved in (9:1) MeCN/H_2O (+ 0.1% TFA) (5.0 mL), filtered through a short pad of sand/C18® (Santiago; 230-400 MESH) and washed with (9:1) MeCN/H_2O (+ 0.1% TFA) (3×10 mL). The filtrate was concentrated under reduced pressure and the crude product was purified by preparative HPLC chromatography (Arion Plus (21.2 × 250 mm, 5 µm) column): MeCN/H_2O (+ 0.1% TFA) gradient 30:70 → 90:10 and flow rate 16 mL/min for 25 min and lyophilized. Product detection at λ = 380 nm. Obtained compound had to be re-purified by column chromatography on silica gel in HILIC mode (puri*Flash*® from *Interchim,* SiO_2 (25 g) column). H_2O/MeCN (+ 0.1% TFA) gradient 0:100 → 5:95 and flow rate 20 mL/min for 20 min and lyophilized.

Yield: 6.0 mg (0.009 mmol, 31%) of a bright orange amorphous solid.

^{1}H NMR (401 MHz, CD_3CN): δ 9.58 (s, 1H), 8.59 (ddd, *J* = 7.8, 2.0, 1.3 Hz, 1H), 8.57 – 8.54 (m, 2H), 8.53 (td, *J* = 1.8, 0.6 Hz, 1H), 7.75 (td, *J* = 7.7, 0.6 Hz, 1H), 7.69 (dt, *J* = 7.6, 1.3 Hz, 1H), 7.62 (d, *J* = 8.8 Hz, 1H), 7.27 – 7.22 (m, 2H), 6.45 (dd, *J* = 8.7, 2.3 Hz, 1H), 6.32 (d, *J* = 2.3 Hz, 1H), 4.06 – 3.99 (m, 4H), 3.97 (s, 3H), 2.27 (s, 3H overlapped with deuterated solvent), 1.91 (s, 9H).

^{19}F NMR (376 MHz, CD_3CN): δ -76.25.

^{13}C{1H} NMR (101 MHz, CD_3CN): δ 167.38, 167.37, 166.2, 155.8, 155.3, 150.4, 138.5, 137.4, 136.4, 133.1, 132.9 (2×CH), 132.2, 130.4, 129.0, 127.6, 124.5, 120.9, 116.5 (2×CH), 111.0, 109.0, 97.2, 78.4, 56.9 (O$\underline{C}H_3$), 52.6 (2×CH_2), 28.7 (3×CH_3), 17.1 (CH_2), 16.8 (CH_3).

HRMS (ESI): m/z calcd. for $C_{33}H_{33}O_3N_4$ [M^+] 533.2547, found 533.2549.

1-(*tert*-Butyl)-3-(4-(7-(diethylamino)-4-methyl-2-oxo-2*H*-chromen-3-yl)phenyl)-5-(4-methoxyphenyl)-1,2,4-triazin-1-ium 2,2,2-trifluoroacetate (Trz⁺Coum8)

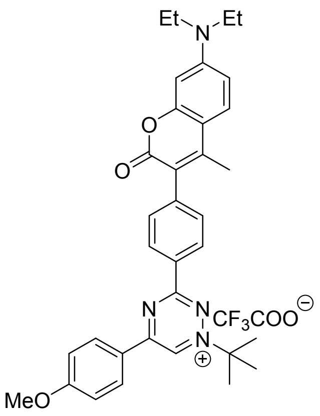

SMeTrz⁺5 (15.0 mg, 0.037 mmol), boronic acid **Coum-BA2** (1.3 equiv., 17.0 mg, 0.048 mmol), copper(I) thiophene-2-carboxylate (2.2 equiv., 15.6 mg, 0.082 mmol) and $Pd(PPh_3)_4$ (10 mol%, 4.3 mg, 0.004 mmol) were placed in an argon flushed flask and anhydrous 1,4-dioxane (600 µL) was added. Anhydrous DMF (100 µL) was then added for better solubilization of the reagents. The mixture was stirred under argon at 60 °C (heating mantle) for 2 h. After cooling to rt, the mixture was diluted with DCM (20 mL) and washed with sat. aq. $NaHCO_3$ (20 mL) and extracted with DCM (2×20 mL). The combined organic extracts were dried over Na_2SO_4, filtered, and concentrated under reduced pressure. The mixture was then re-dissolved in (9:1) MeCN/H_2O (+ 0.1% TFA) (5.0 mL), filtered through a short pad of sand/C18® (Santiago; 230-400 MESH) and washed with (9:1) MeCN/H_2O (+ 0.1% TFA) (3×10 mL). The filtrate was concentrated under reduced pressure and the crude product was purified by preparative HPLC (Arion Plus (21.2 × 250 mm, 5 µm) column): MeCN/H_2O (+ 0.1% TFA) gradient 30:70 → 90:10 and flow rate 16 mL/min for 30 min and lyophilized. Product detection at λ = 400 nm. Yield: 9.0 mg (0.014 mmol, 37%) of a bright orange amorphous solid.

^{1}H NMR (401 MHz, CD_3CN): δ 9.58 (s, 1H), 8.69 – 8.61 (m, 2H), 8.61 – 8.54 (m, 2H), 7.65 – 7.57 (m, 3H), 7.30 – 7.22 (m, 2H), 6.77 (dd, *J* = 9.1, 2.6 Hz, 1H), 6.60 (d, *J* = 2.6 Hz, 1H), 3.99 (s, 3H), 3.48 (q, *J* = 7.0 Hz, 4H), 2.28 (s, 3H), 1.94 (s, 12H overlapped with deuterated solvent), 1.20 (t, *J* = 7.0 Hz, 6H).

^{19}F NMR (376 MHz, CD_3CN): δ -76.67.

^{13}C{1H} NMR (101 MHz, CD_3CN): δ 167.36, 167.34, 166.2, 162.1, 156.2, 151.6, 150.3, 143.4, 136.2, 132.97 (2×CH), 132.91 (2×CH), 132.0, 129.7 (2×CH), 127.8, 124.6, 120.3, 116.5 (2×CH), 110.1, 98.0, 78.4, 56.9 (O<u>C</u>H_3), 42.5 (2×CH_2), 28.8 (3×CH_3), 16.7 (CH_3), 12.7 (2×CH_3). Signal of one quat. carbon was not found.

HRMS (ESI): m/z calcd. for $C_{34}H_{37}O_3N_4$ [M^+] 549.2860, found 549.2862.

Fluorescent properties

To investigate the fluorogenic properties of the coumarin-modified triaziniums (**Trz⁺Coum**) in their IEDDA reaction with ***endo*-BCN**, we first confirmed formation of the corresponding click products between each of the coumarin-substituted heterodiene and ***endo*-BCN** by HPLC-MS analysis (Figure S3 – S5). Subsequently, the absorbance and fluorescence emission spectra before and after addition of an excess ***endo*-BCN** were recorded.

HPLC-MS analysis of the reagents and the respective click products

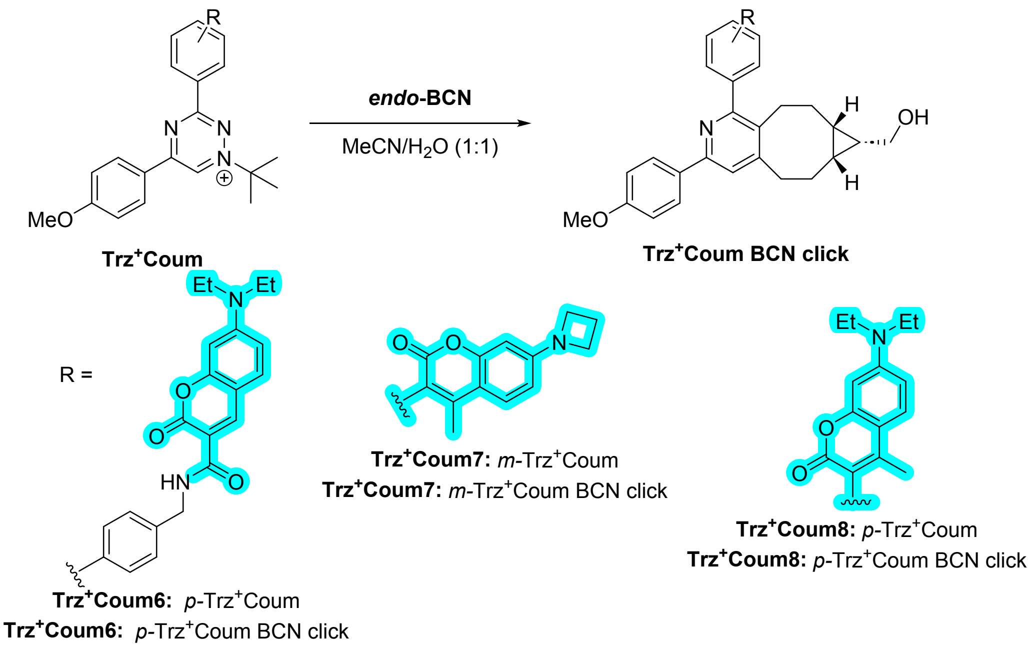

10 mM solution of **Trz+Coum** (5 µL) in DMSO/H_2O (1:1) (Figure S2, S3 and S4) was combined in 95 µL of MeCN/H_2O (1:1) with 100 mM solution of ***endo*-BCN** (2.5 µL) in MeCN/H_2O (1:1). The final concentration of each coumarin derivative was 500 µM and ***endo*-BCN** was used in 5-molar excess. The resulting mixture was incubated in a shaker at rt for 15 min and then analyzed by HPLC-MS (Figure S2, S3 and S4).

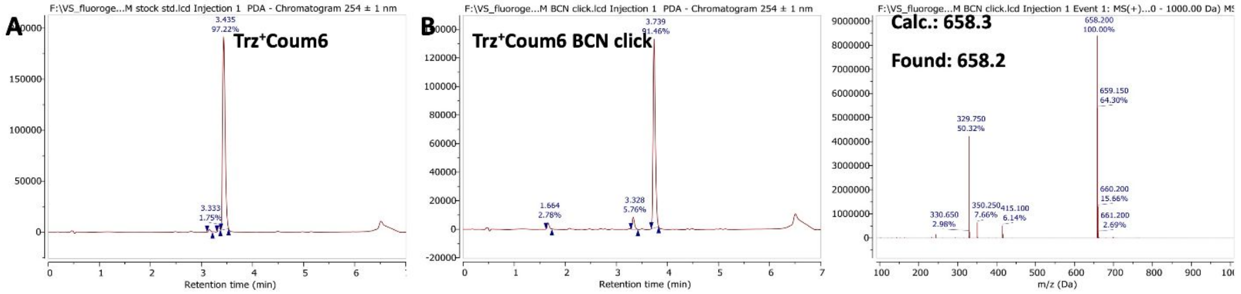

Figure S3 : Exported HPLC-MS chromatogram of **Trz+Coum6** before (A) and after the reaction with ***endo*-BCN** (B) with the corresponding MS chromatogram of the click product on the right (λ = 254 nm) after 15 min. Solvent system: solvent A: H_2O + 0.05% HCOOH; solvent B: MeCN + 0.05% HCOOH. Gradient: 5% B → 95% B (4.0 min), then 95% B (0.5 min), then 95% B → 5% B (0.5 min) and 5% B (1.0 min).

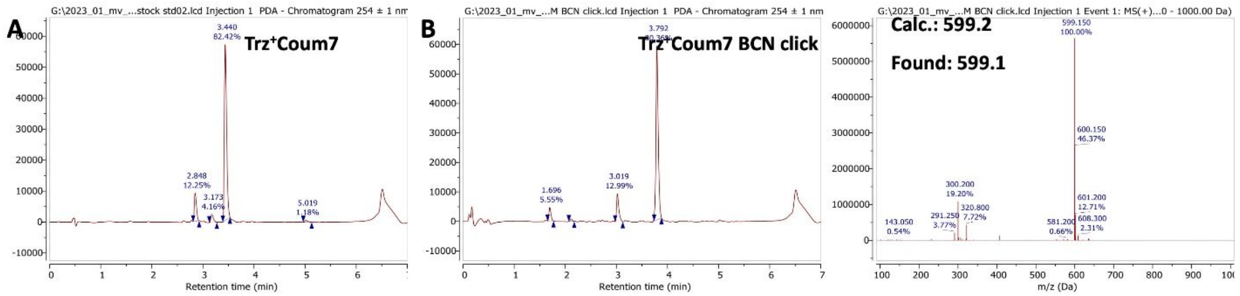

Figure S4: Exported HPLC-MS chromatogram of **Trz⁺Coum7** before (A) and after the reaction with ***endo*-BCN** (B) with the corresponding MS chromatogram of the click product on the right (λ = 254 nm) after 15 min. Solvent system: solvent A: H_2O + 0.05% HCOOH; solvent B: MeCN + 0.05% HCOOH. Gradient: 5% B → 95% B (4.0 min), then 95% B (0.5 min), then 95% B → 5% B (0.5 min) and 5% B (1.0 min). Signal eluting at 2.8 min is a residual impurity from the **Trz⁺Coum7**.

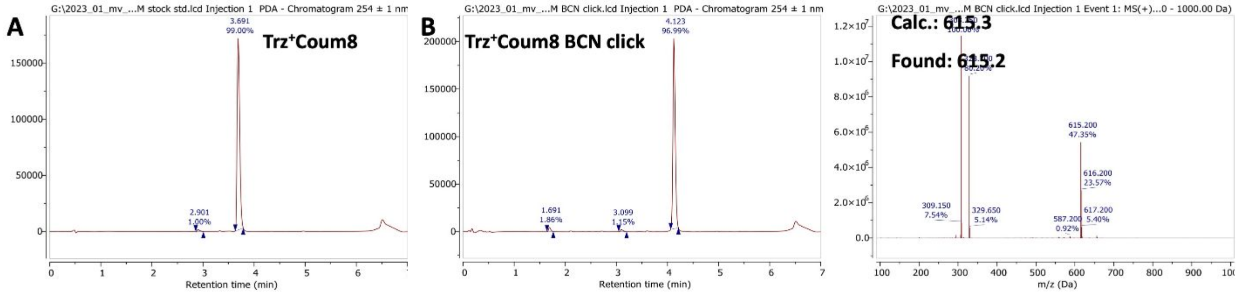

Figure S5: Exported HPLC-MS chromatogram of **Trz⁺Coum8** before (A) and after the reaction with ***endo*-BCN** (B) with the corresponding MS chromatogram of the click product on the right (λ = 254 nm) after 15 min. Solvent system: solvent A: H_2O + 0.05% HCOOH; solvent B: MeCN + 0.05% HCOOH. Gradient: 5% B → 95% B (4.0 min), then 95% B (0.5 min), then 95% B → 5% B (0.5 min) and 5% B (1.0 min).

Absorbance of SMeTrz⁺5 and the click products

Absorbance spectra of all click products formed in the reaction of **Trz⁺Coums** with ***endo*-BCN** were measured at 10 µM final click product concentration in PBS buffer (pH = 7.4) or MeCN/H_2O (1:1). The respective click products were prepared by mixing the respective **Trz⁺Coums** with 10 equiv. ***endo*-BCN**. Absorption maxima of the click products are summarized in Table S3. Absorbance spectra of the parent **SMeTrz⁺5** was recorded at 10 µM final concentration in PBS buffer (pH = 7.4) or MeCN/H_2O (1:1) for a comparison. The plots are depicted in Figure S6.

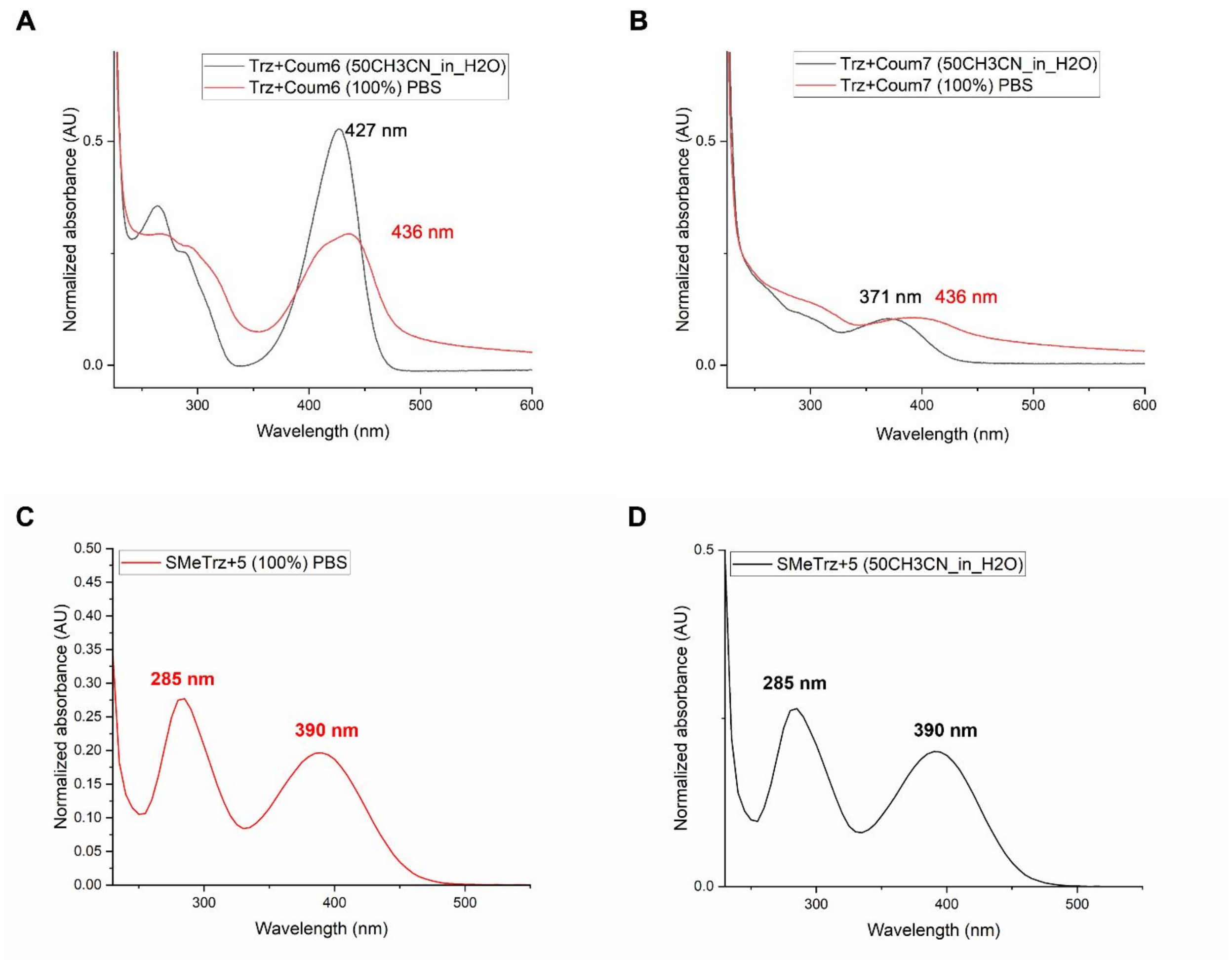

Figure S6: Absorbance spectra A) and B) of the click products formed in the reaction of relevant **Trz⁺Coums** with ***endo*-BCN** or C) and D) of the parent **SMeTrz⁺5** at 10 µM final click product concentration in PBS buffer or MeCN/H_2O (1:1). *Due to low solubility of **Trz⁺Coum8 BCN click** product, we could not measure the UV-Vis spectra of this product.

Fluorescence turn-on measurements

All triaziniums were purified by analytical HPLC prior to turn-on fluorescence measurements since traces of fluorescent impurities can significantly influence the maximum observable ratio. Analytically pure samples were used directly for the measurements and were stored in the dark on ice before analysis. The impurities can be best seen using fluorescence detection on the HPLC. Representative HPLC analysis before and after purification of the dyes is depicted in Figure S7 (showing compound **Trz⁺Coum8** as an example).

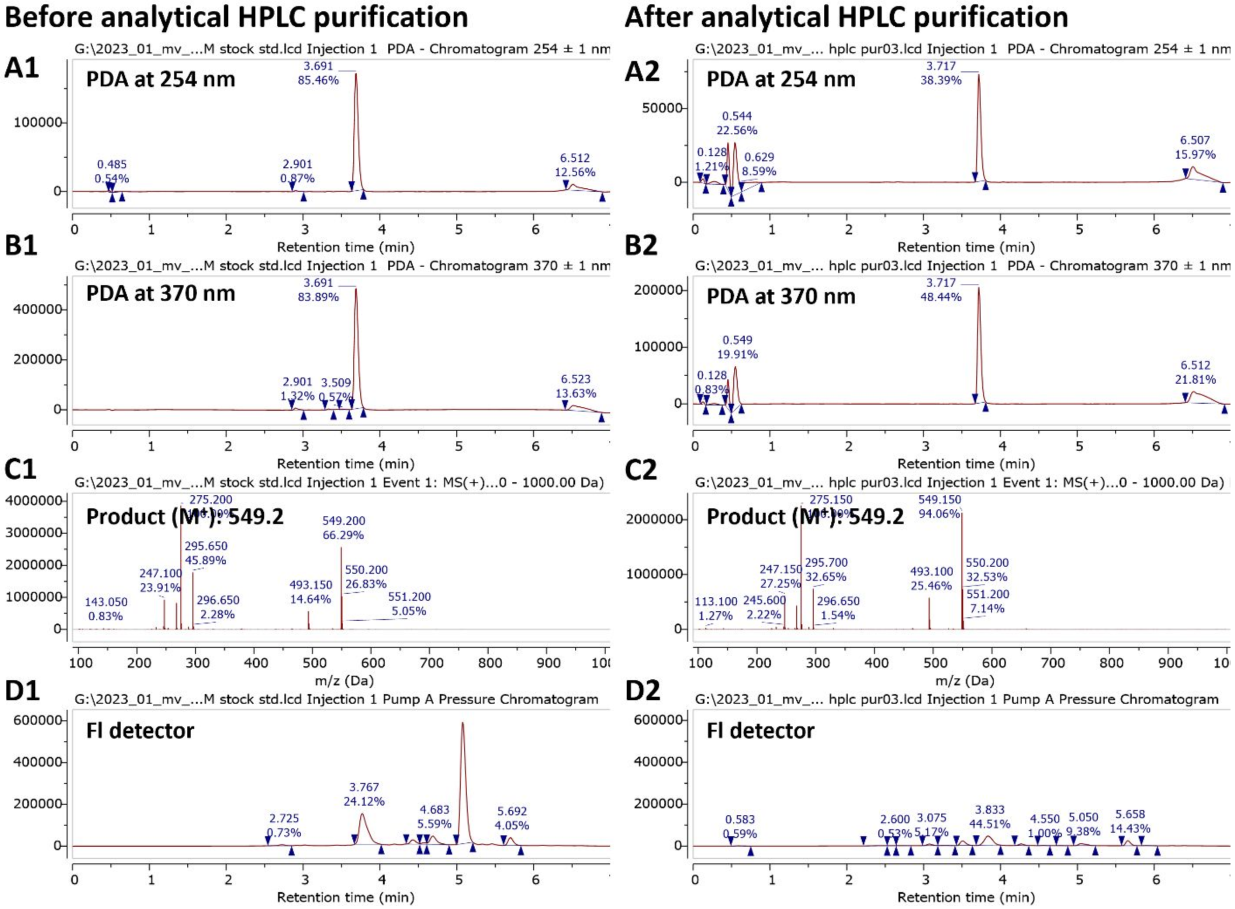

Figure S7: Exported HPLC-MS chromatograms of the selected **Trz⁺Coum8** before (left) and after (right) the analytical HPLC purification recorded at λ = 254 nm (A) or at characteristic absorption λ = 370 nm (B) with the corresponding MS chromatogram (C) including the corresponding analysis from fluorescence detector (D).

The turn-on fluorescence measurements were performed as follows: 7 µL of the sample collected from the analytical HPLC (typical concentration approximately 50 µM) was diluted in 1 mL PBS buffer (1×, pH = 7.4) or MeCN/H_2O (1:1) and solution of ***endo*-BCN** (7 µL from 100 mM fresh stock in MeCN/H_2O (1:1)) was added. The cuvette was immediately inserted into fluorescence spectrophotometer and the measurement was started. All probes were excited at their characteristic absorption (370 nm for **Trz⁺Coum7** and **Trz⁺Coum8** or 420 nm for **Trz⁺Coum6**) and fluorescence was collected from 390 nm or 450 nm, respectively, to 650 nm. All spectra were recorded at several time points and all measurements were typically repeated three-four times. The data were processed using OriginPro 9.1 software. All spectra were subtracted from the baseline (PBS buffer or MeCN/H_2O (1:1) as the blank). The turn-on values were finally calculated from the observed fluorescence intensities of the click products at the emission maximum divided by the highest residual fluorescence of the quenched **Trz⁺Coum** dyes. These values are summarized in Table S3. The plots are depicted in Figure S8. The highest fluorescence signal intensities for reaction with the ***endo*-BCN** are listed.

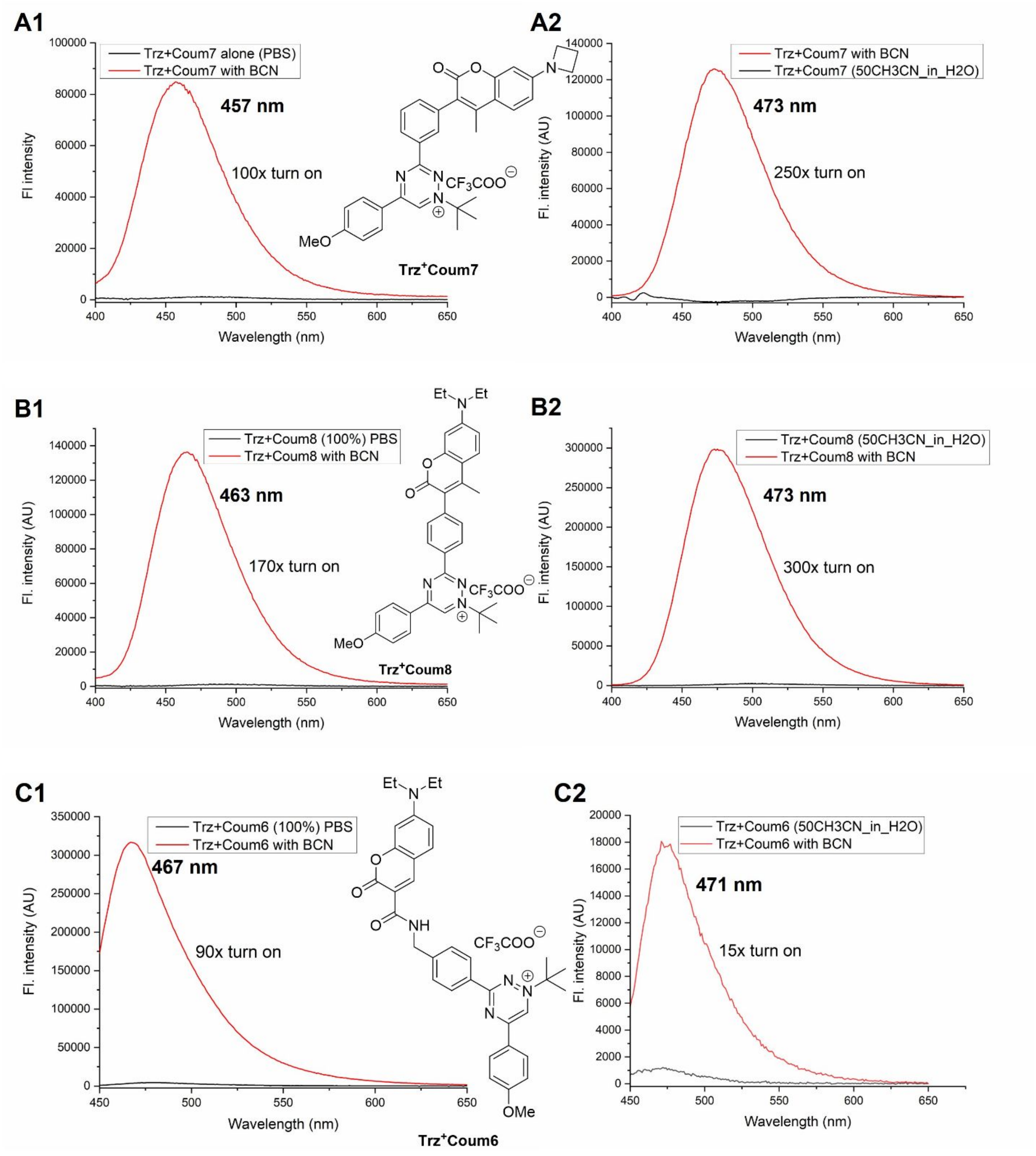

Figure S8: Fluorescence spectra of the click products formed in the reaction of **Trz⁺Coums** with ***endo*-BCN** (indicated as number-x = x-fold) in PBS buffer or MeCN/H_2O (1:1).

Table S3: Summary of the Abs. and Em. maxima and of the turn-on values for the click products

Trz^+Coum	Solvent system	λ_{Abs} (nm)	λ_{Em} (nm)	Fluorescence turn-on

**Trz⁺Coum7**	PBS	436	457	100×
**Trz⁺Coum7**	MeCN/H_2O (1:1)	371	473	250×
**Trz⁺Coum8**	PBS	ND*	463	170×
**Trz⁺Coum8**	MeCN/H_2O (1:1)	ND*	473	300×
**Trz⁺Coum6**	PBS	436	467	90×
**Trz⁺Coum6**	MeCN/H_2O (1:1)	427	471	15×

*Non-detected.

Reaction kinetics

General procedure for the kinetic study by UV/VIS used for all triazinium salts: The reaction kinetics were monitored by following the decrease in the concentration (absorbance) of the starting triazinium in time after addition of excess dienophile under pseudo first order conditions. A typical procedure: a 10 mM solution of the triazinium in MeCN/H_2O (1:1) and 100 mM solution of ***endo*-BCN** in MeCN/H_2O (1:1) was added to MeCN/PBS (1:9) mixture in the cuvette inserted in the spectrophotometer to give a final volume of 3 mL and the measurement was immediately started. The final concentration of triaziniums was 10 µM using 10-20 equiv. of ***endo*-BCN**. The decrease in the absorption of triazinium salt was followed over 10-35 min in intervals of 0.15 or 1.00 min. The time-dependent measurements were performed at the corresponding characteristic absorption wavelengths of each triazinium, which was determined by UV/VIS spectroscopy before each measurement. The measured intensity of the absorption was plotted against time. Fitting the curves with single exponential equation ($y = y_0 + Ae^{-k/t}$) provided the observed rate constants. The second order rate constants were obtained by plotting the observed rate constants *vs* concentration of the dienophile and fitting the data with a linear function which provided the second order rate constants from the slope of the plot. All data were processed using OriginPro 9.1 or MS Excel 365 and are summarized in Table S4 and Figure S9.

Second order rate constants of differently C3-substituted Trz⁺1

Table S4: Second-order rate constants (in M^{-1} s^{-1}) for IEDDA of **SMeTrz⁺1**, **ArTrz⁺2a, ArTrz⁺2u** and **HTrz⁺3** with ***endo*-BCN** determined at room temperature in MeCN/PBS (1:9) mixture.

Entry	Triazinium	Second-order rate constant (M^{-1} s^{-1})
**1**	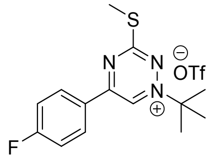 **SMeTrz⁺1**	18.7 ± 0.2
**2**	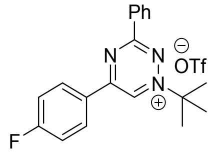 **ArTrz⁺2a**	59 ± 1

**3**	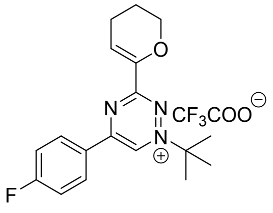 **ArTrz⁺2u**	111.6 ± 0.4
**4**	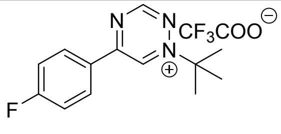 **HTrz⁺3**	17.83 ± 0.09

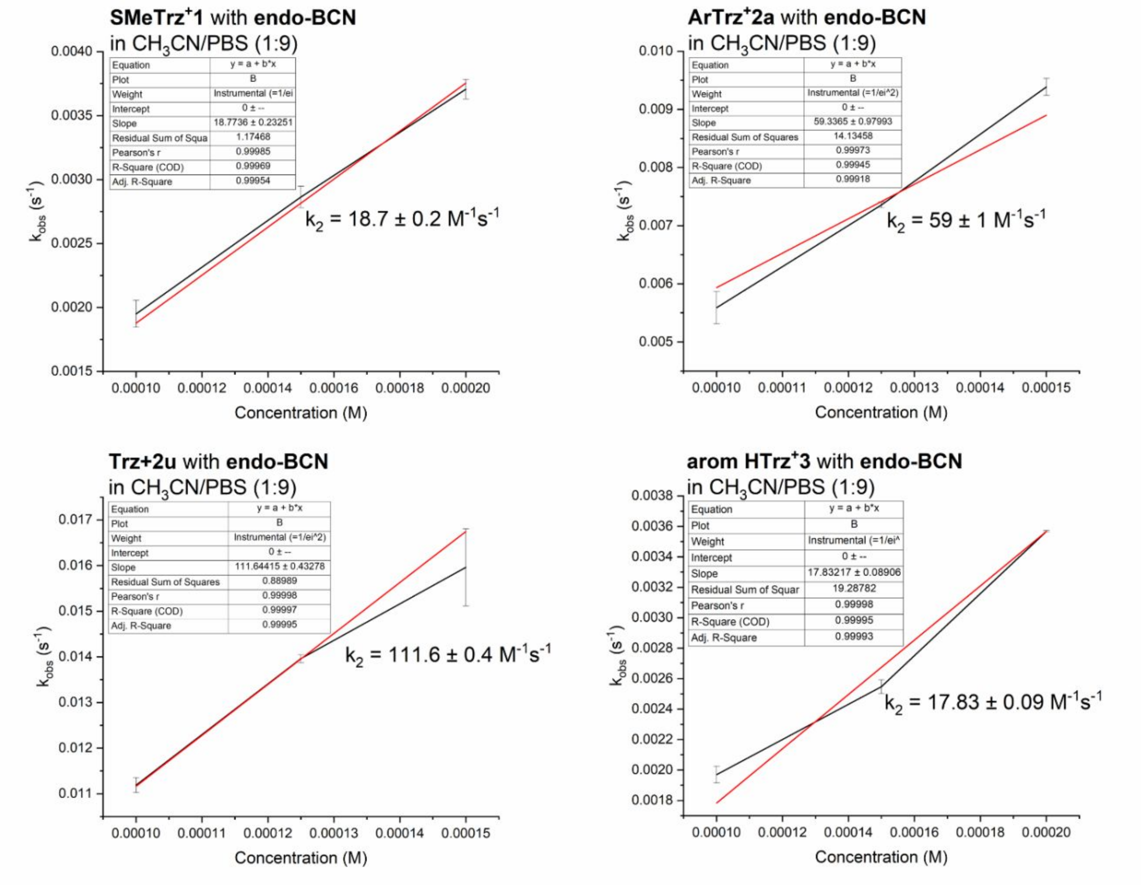

Figure S9: Data from kinetic measurements. Shown are the fitted data, reagents used, conditions and the respective second order rate constants.

Stability of *N*1-alkyl-1,2,4-triaziniums in cell growth medium DMEM containing FBS (fetal bovine serum)

Stability of differently substituted *tert*-butyl triazinium salts was examined in full cell growth medium (10% FBS in L-15) and monitored by HPLC-MS (Figure S10). 10 µL of a 10 mM solution of the corresponding triazinium in MeCN/H_2O (1:1) were diluted with 90 µL of 10% FBS in L-15. The final concentration of each triazinium was 1 mM. The integral of the absorption signal of each triazinium at the corresponding absorption maximum of 278 nm for **SMeTrz⁺1** and **ArTrz⁺2a,** 325 nm for **ArTrz⁺2u** and 340 nm **for HTrz⁺3** was measured immediately and then after 6, 12 and 24 h of incubation at 37 °C, respectively. The samples for HPLC-MS measurements were diluted at 1:1 ratio with MeCN, centrifuged (5 min, 10000 rpm) and 5 µL of the resulting sample were injected. All experiments were performed in duplicate. The initial integral from sample processed in the same way right after dilution in FBS (10% in L-15) was taken as 100%. The measurements were performed on CORTECS C18 column (2.7 µm, 50 × 4.6 mm) using solvent A: H_2O + 0.05% HCOOH; solvent B: MeCN + 0.05% HCOOH forming the following gradient 5% B → 95% B (2.5 min), then 95% B (0.5 min), then 95% B → 5% B (0.5 min) and 5% B (1.0 min) at a flow rate of 2.00 mL/min.

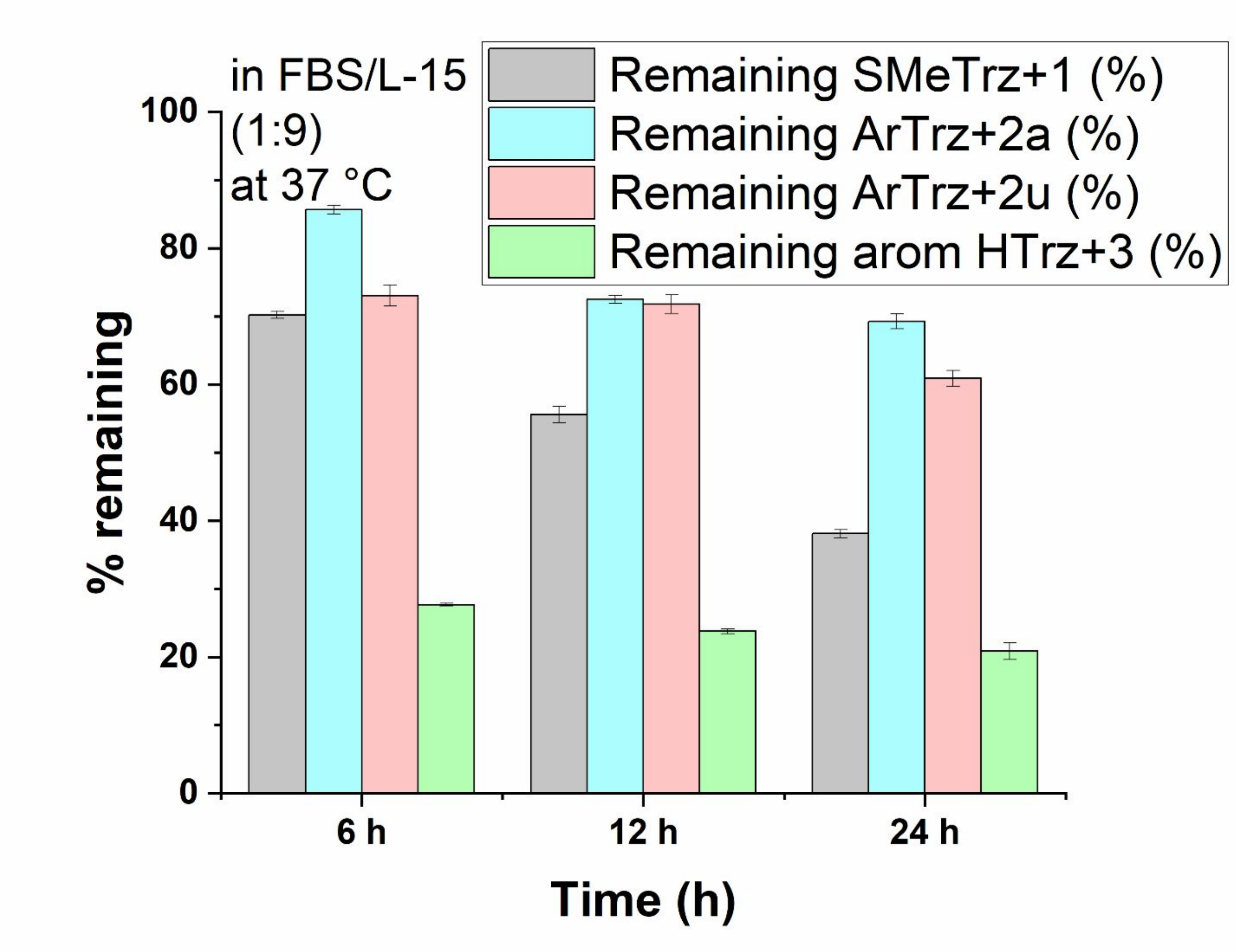

Figure S10: Graphical representation of stability of four differently substituted triaziniums in full cell growth medium (10% FBS in L-15) determined by HPLC after incubation at 37 °C.

Calculated log*P* values

Calculations of log*P*

Computational log*P*(comp): In addition to using StarDrop software, we also calculated the log*P* values by the online application ALOGPS 2.1 which can be found in the following link: http://www.vcclab.org/lab/alogps/.

Table S5: The calculated log*P* values of various triaziniums. Values for diphenyl tetrazine (diPhTz) are indicated for comparison

Entry	Compound	log*P* StarDrop	log*P* ALOGPS
**1**	**diPhTz**	2.152	1.90
**2**	**SMeTrz$^+$1**	5.152	1.17
**3**	**ArTrz$^+$2a**	5.596	1.88
**4**	**ArTrz$^+$2u**	5.261	1.74
**5**	**HTrz$^+$3**	4.294	0.16

BCN-TPP cell labeling experiments

HeLa cells were seeded on a 96-well plate at a density of 17 500 cells per well. After 48 hours, the medium was exchanged for fresh medium containing 5 µM **BCN-TPP**[4] and then the cells were incubated for 10 min. After 10 min, the cells were 3 times washed with complete DMEM. After that, **Trz$^+$Coum7**, **Trz$^+$Coum8** and **Trz$^+$Coum6** were added to the cells in concentrations 1 µM or 0.1 µM in LP-15 medium with 10% FBS and DRAQ5 (diluted 1:1000) for 15 min. The cells without prior incubation with **BCN-TPP** were incubated with **Trz$^+$Coum7**, **Trz$^+$Coum8** and **Trz$^+$Coum6** and were used as a negative control. The previously reported **TzCoum** was used in the experiments as a positive control.[3] The cells were imaged on a confocal microscope without additional washing (Figure S12 - S14). Microscope setup: sequential scanning, Coumarin – laser 405 nm, emission detector (410 – 676 nm); DRAQ5 – laser 639 nm, emission detector (642 – 756 nm). The same experiment was performed for flow cytometry (Figure S12I).

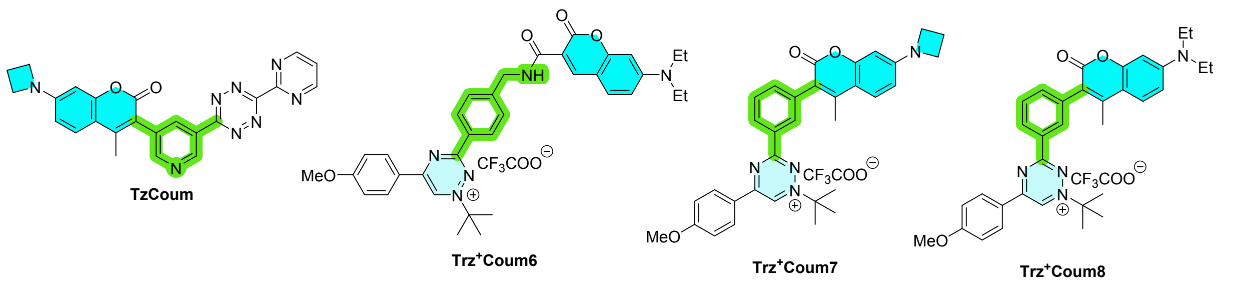

Figure S11: Structures of fluorogenic probes for BCN-TPP intracellular labeling experiments.

Microscope setup: 405 nm laser intensity: 1.5 %, detector: 650 V

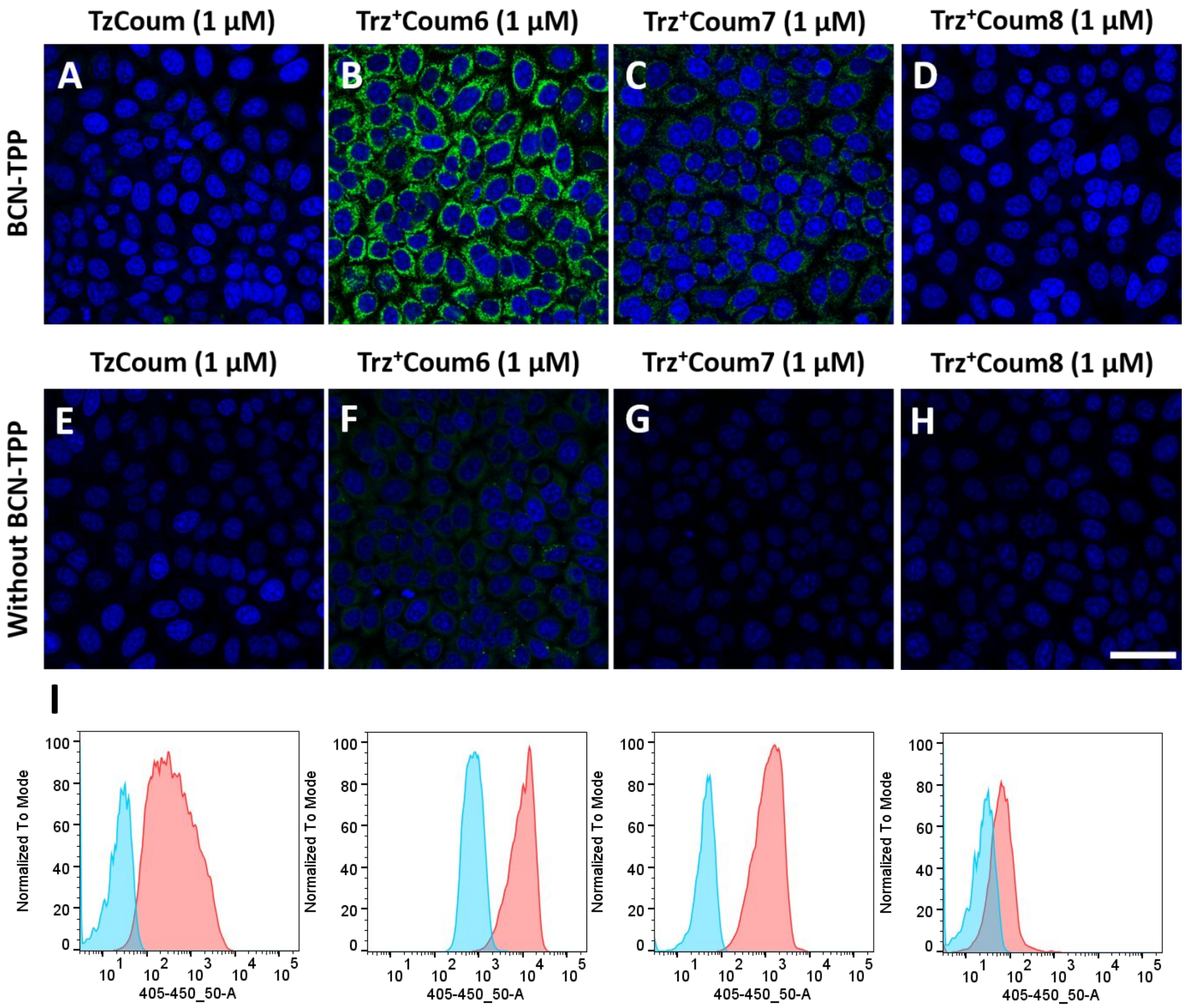

Figure S12: Live cell labeling experiment in which 1 µM concentration of the compounds was used in all cases. The cells were first incubated with 5 µM **BCN-TPP** (10 min), washed and then with **TzCoum** (A), **Trz⁺Coum6** (B), **Trz⁺Coum7** (C) and **Trz⁺Coum8** (D) in 1 µM concentration for 15 min. Control cells (E, F, G, H) were treated in the same way but without prior incubation with **BCN-TPP**. Click labeling is shown in green. Nuclei were stained with DRAQ5 and are shown in blue. Scale bar: 50 µm. The same experiment was performed and the cells were analyzed by flow cytometry. Microscope setup – 405 nm laser intensity: 1.5 %, detector: 650 V. (I) Corresponding histograms from FACS analysis.

Microscope setup: 405 nm laser intensity: 2.5 %, detector: 750 V

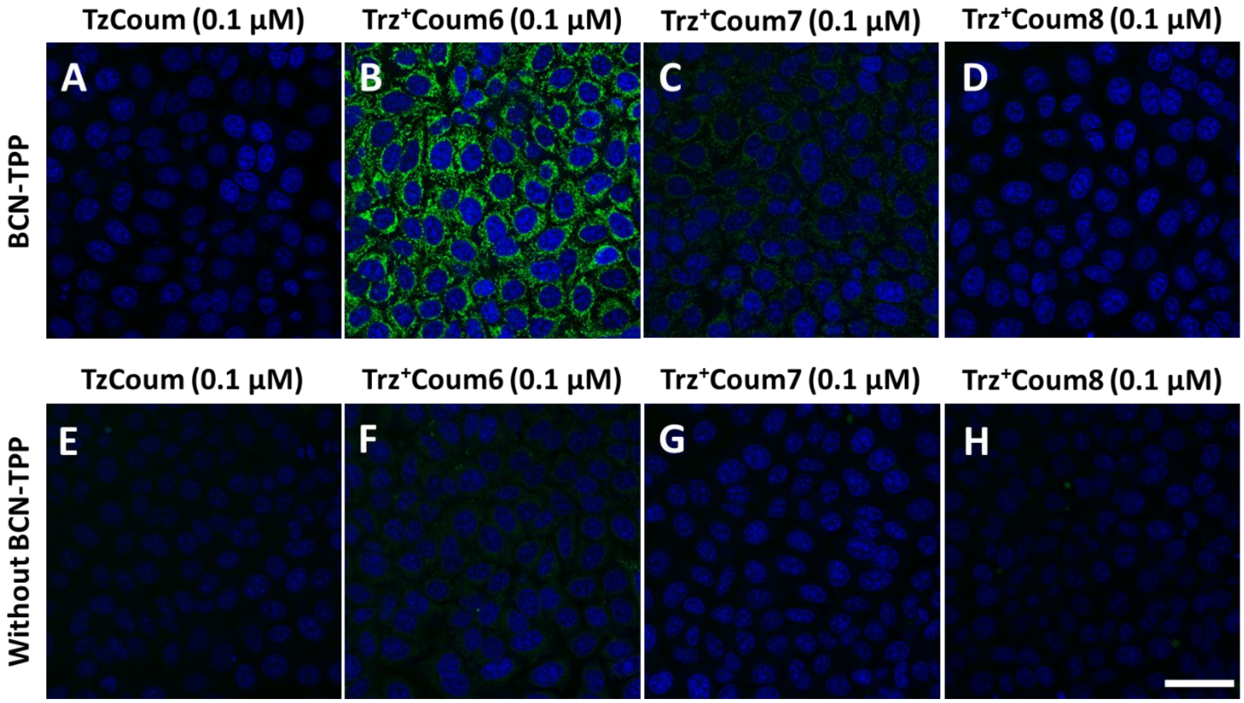

Figure S13: Fig. 2 Live cell labeling experiment in which 0.1 µM concentration was used in all samples. The cells were first incubated with 5 µM **BCN-TPP** (10 min) and then with **TzCoum** (A), **Trz$^+$Coum6** (B), **Trz$^+$Coum7** (C) and **Trz$^+$Coum8** (D) in 0.1 µM concentration for 15 min. Control cells (E, F, G, H) were treated in the same way but without prior incubation with **BCN-TPP**. Click labeling is shown in green. Nuclei were stained with DRAQ5 and are shown in blue. Scale bar: 50 µm. Microscope setup – 405 nm laser intensity: 2,5 %, detector: 750 V.

*Different microscope setup: **TzCoum, Trz⁺Coum7**: 405 nm laser intensity: 1.5 %, detector: 750 V; **Trz⁺Coum8:** 405 nm laser intensity: 3.0 %, detector: 780 V.*

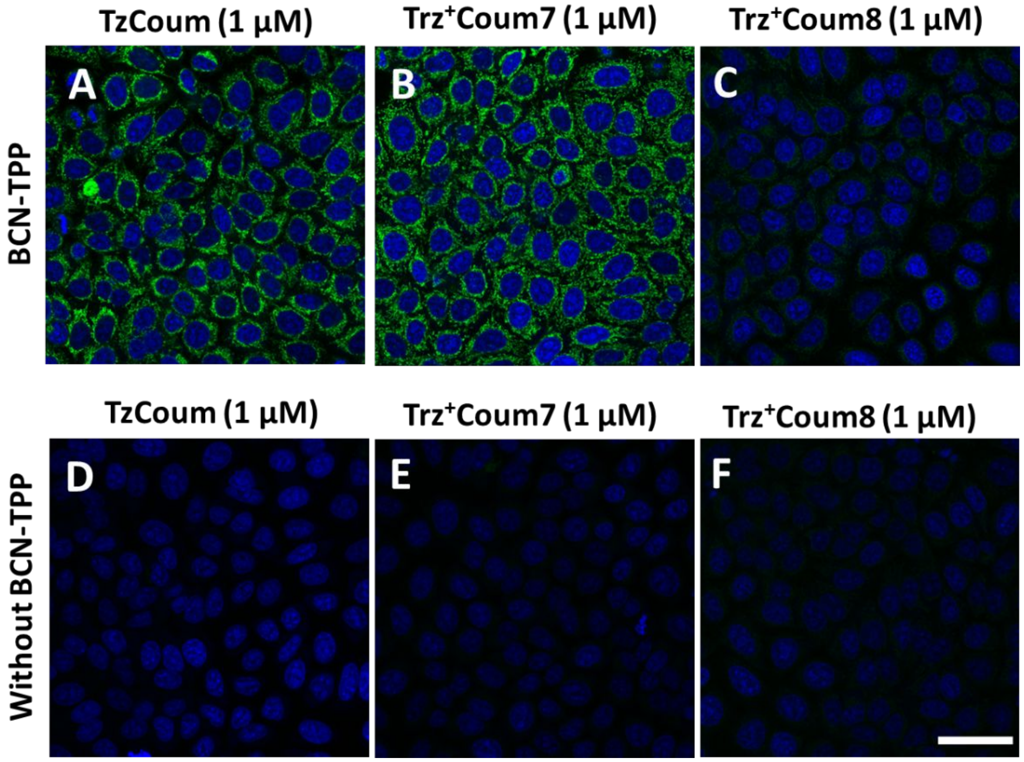

Figure S14: Live cell labelling experiment. Selected compounds **TzCoum**, **Trz⁺Coum7** and **Trz⁺Coum8** at 1 µM concentration were imaged with different laser intensity and different detector settings. The cells were first incubated with 5 µM **BCN-TPP** (10 min) and then with **TzCoum** (A), **Trz⁺Coum7** (B) and **Trz⁺Coum8** (C) at 1 µM concentration for 15 min. Control cells (D, E, F) were treated in the same way but without prior incubation with **BCN-TPP**. Click labeling is shown in green. Nuclei were stained with DRAQ5 and are shown in blue. Scale bar: 50 µm. Microscope setup for **TzCoum** and **Trz⁺Coum7:** 405 nm laser intensity: 1.5 %, detector: 750 V, and for **Trz⁺Coum8**: 405 nm laser intensity: 3 %, detector: 780 V.

Literature

(1) Pellegatti, L.; Vedrenne, E.; Leger, J.-M.; Jarry, C.; Routier, S. *Tetrahedron* **2010**, *66*, 4383.

(2) Šlachtová, V.; Bellová, S.; La-Venia, A.; Galeta, J.; Dračínský, M.; Chalupský, K.; Dvořáková, A.; Mertlíková-Kaiserová, H.; Rukovanský, P.; Dzijak, R.; Vrabel, M. *Angewandte Chemie International Edition* **2023**, *62*, e202306828.

(3) Galeta, J.; Dzijak, R.; Obořil, J.; Dračínský, M.; Vrabel, M. *Chem. Eur. J.* **2020**, *26*, 9945.

(4) Siegl, S. J.; Galeta, J.; Dzijak, R.; Dračínský, M.; Vrabel, M. *Chempluschem* **2019**, *84*, 493.
