# Supplementary material for: Synthesis of C3-Substituted N1-tert-Butyl 1,2,4-Triazinium Salts via the Liebeskind–Srogl Reaction for Fluorogenic Labeling of Live Cells
Source: J Org Chem. 2024 Jan 15;89(20):14634–40. doi: 10.1021/acs.joc.3c02454 (PMC11494656; doi:10.1021/acs.joc.3c02454)

Compound SMeTrz+1 ^{1}H NMR (400 MHz, CD_3CN)

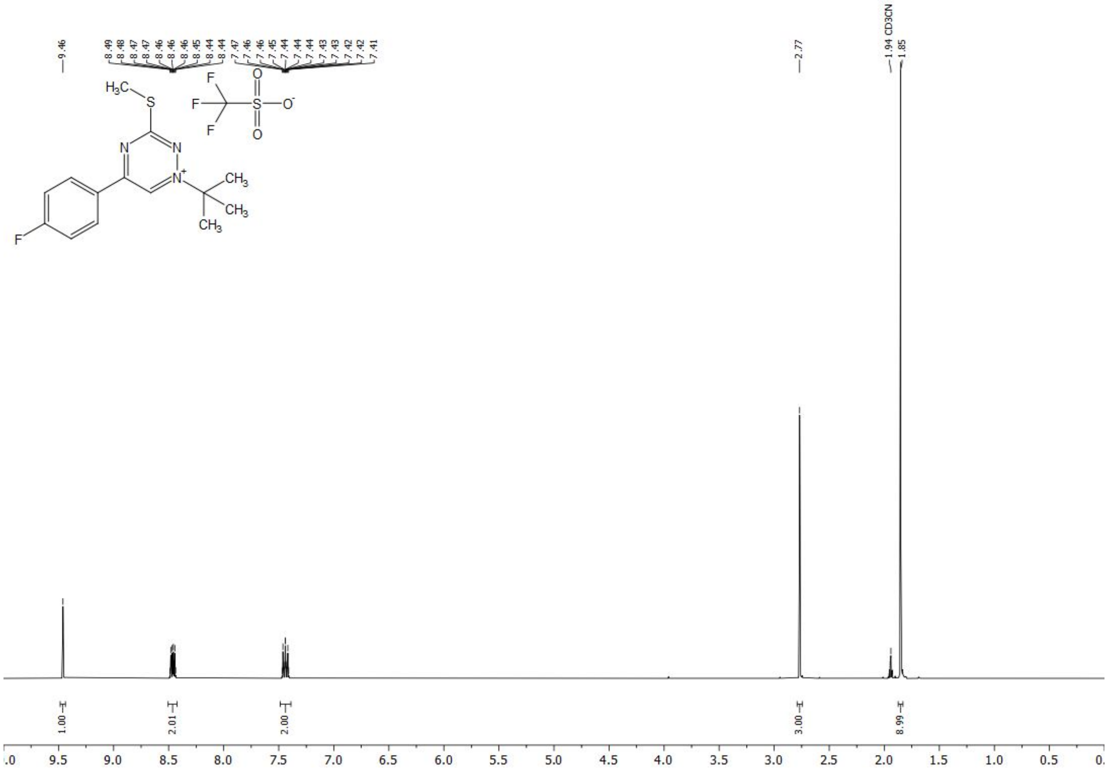

Compound SMeTrz+1 ^{19}F NMR (376 MHz, CD_3CN)

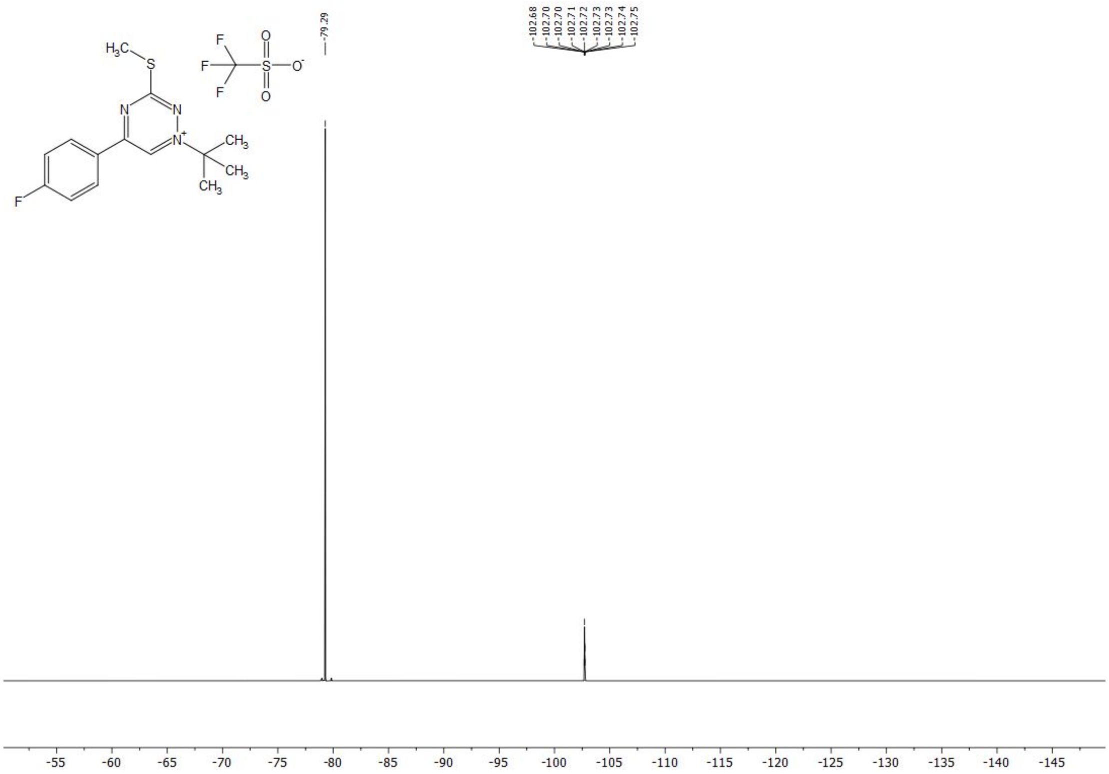

SMeTrz$^+$1 ^{13}C NMR (101 MHz, CD_3CN)

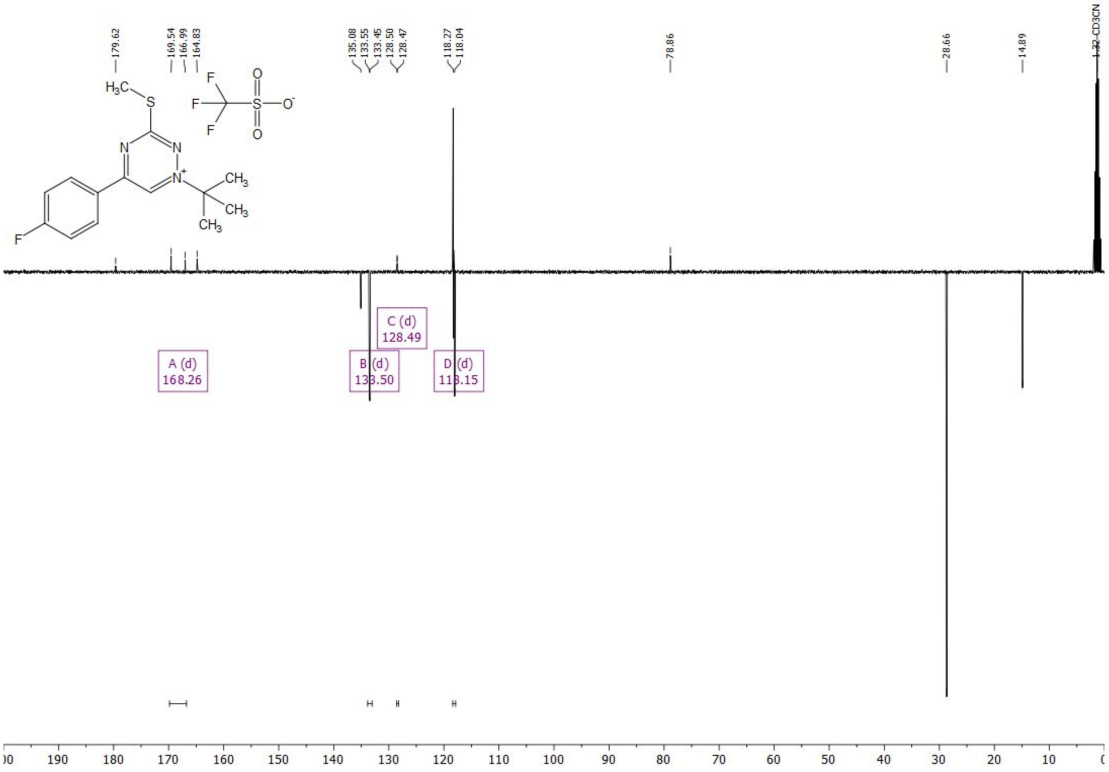

ArTrz$^+$2a ^{1}H NMR (400 MHz, CD_3CN)

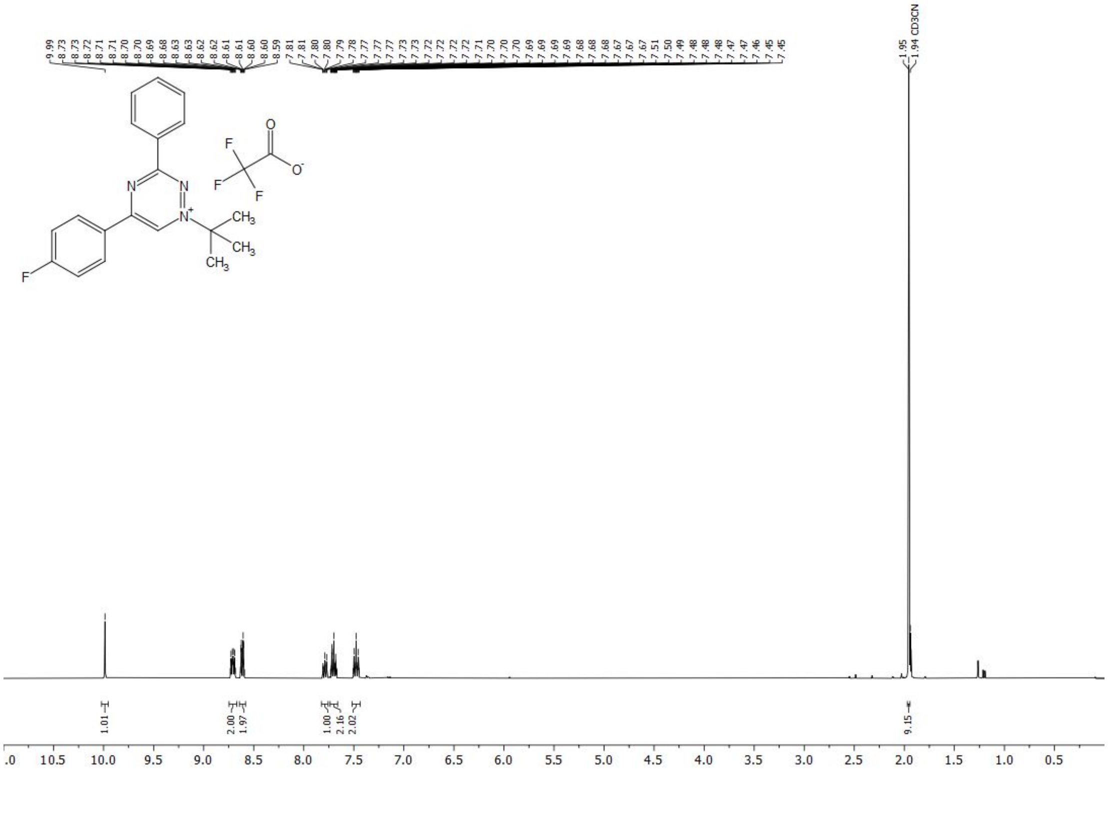

ArTrz⁺2a ^{19}F NMR (376 MHz, CD_3CN)

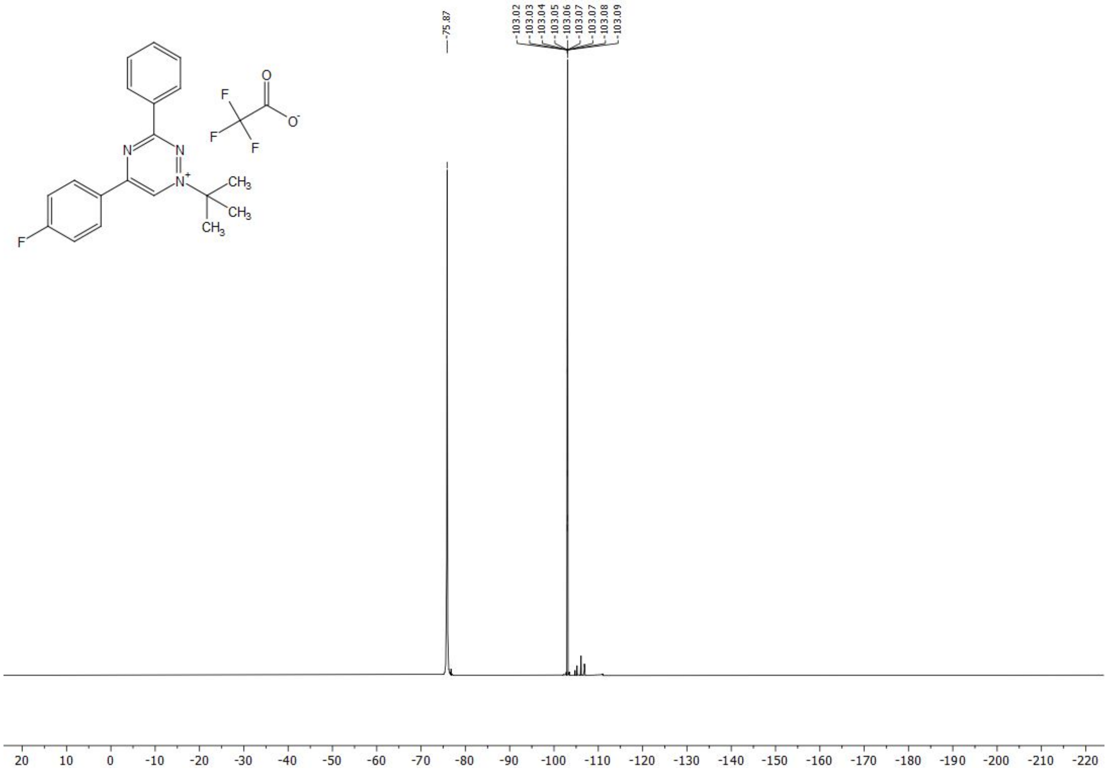

ArTrz⁺2a ^{13}C NMR (101 MHz, CD_3CN)

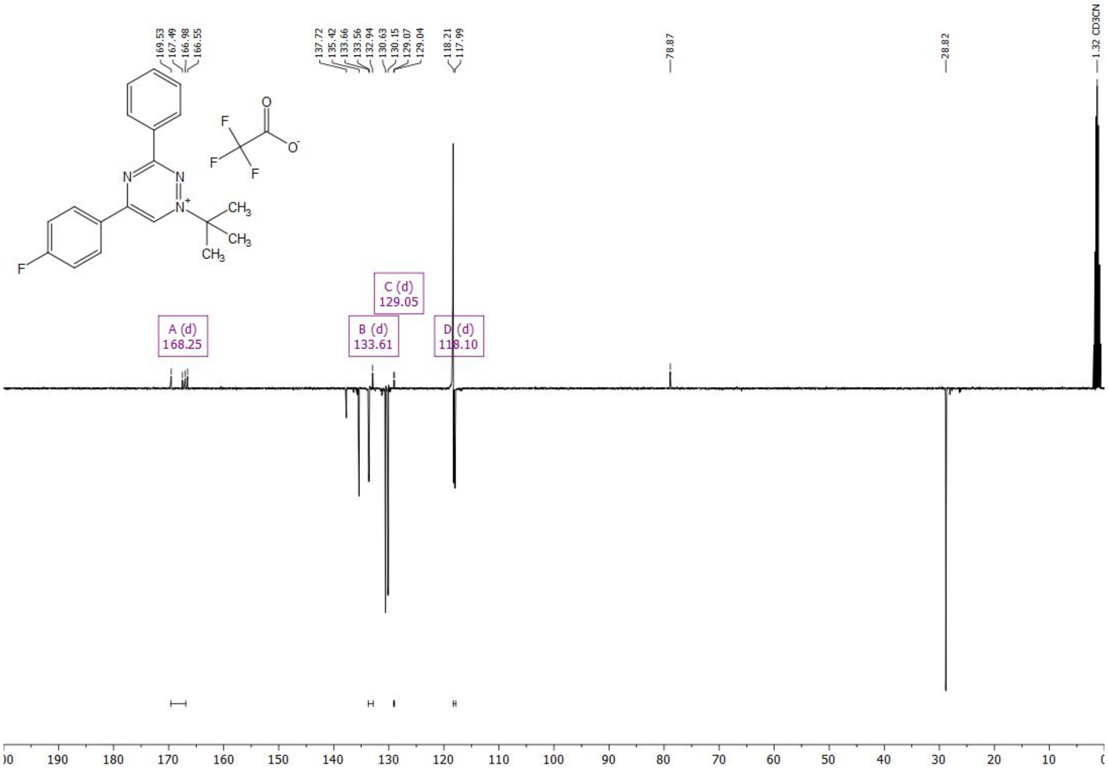

ArTrz⁺2b ^{1}H NMR (400 MHz, CD_3CN)

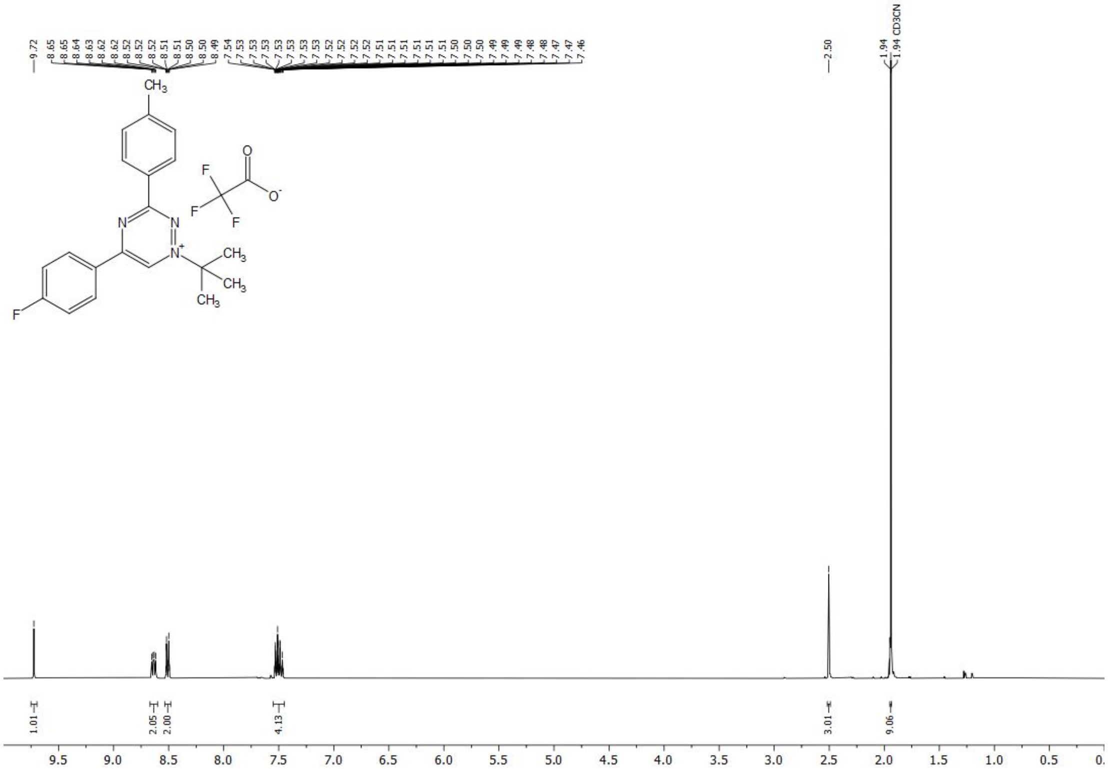

ArTrz⁺2b ^{19}F NMR (376 MHz, CD_3CN)

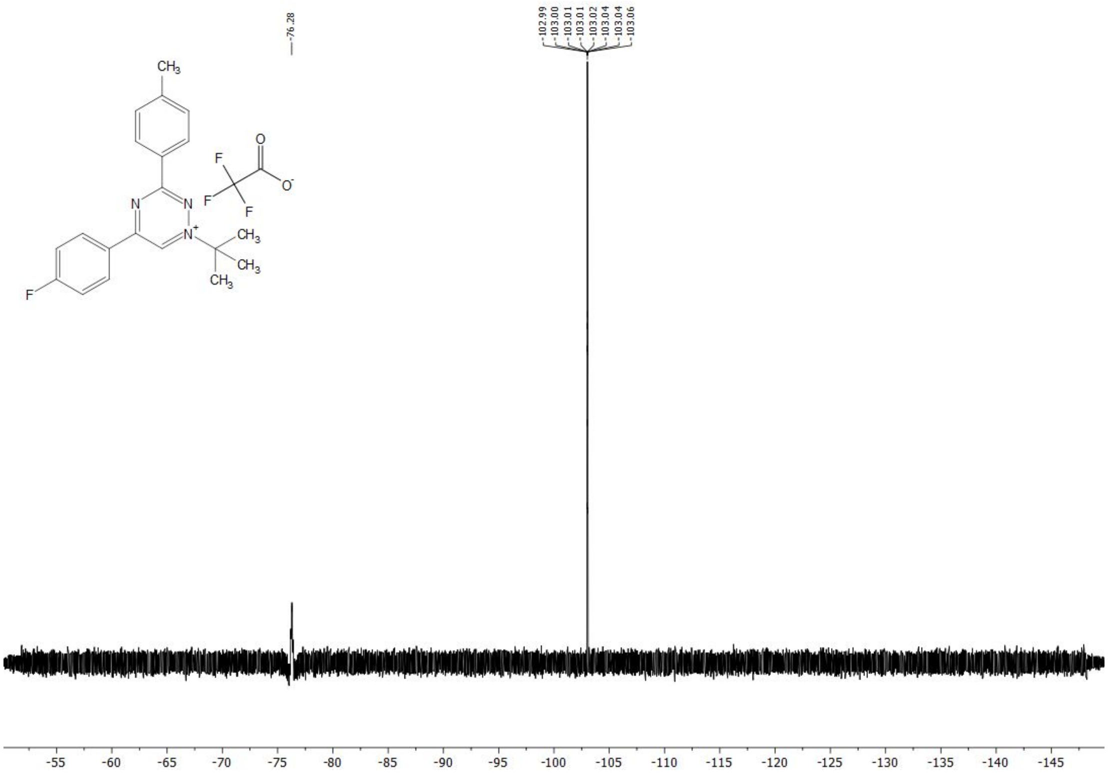

ArTrz⁺2b ^{13}C NMR (101 MHz, CD_3CN)

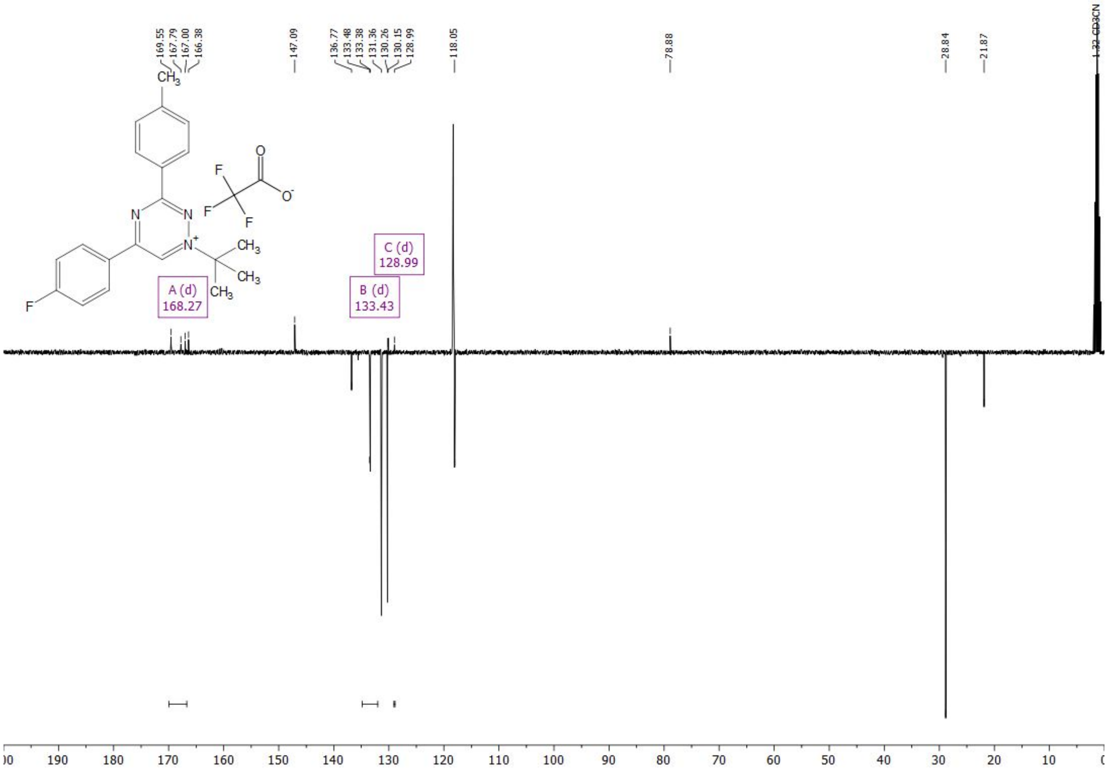

ArTrz⁺2c ^{1}H NMR (400 MHz, MeOH-d_4)

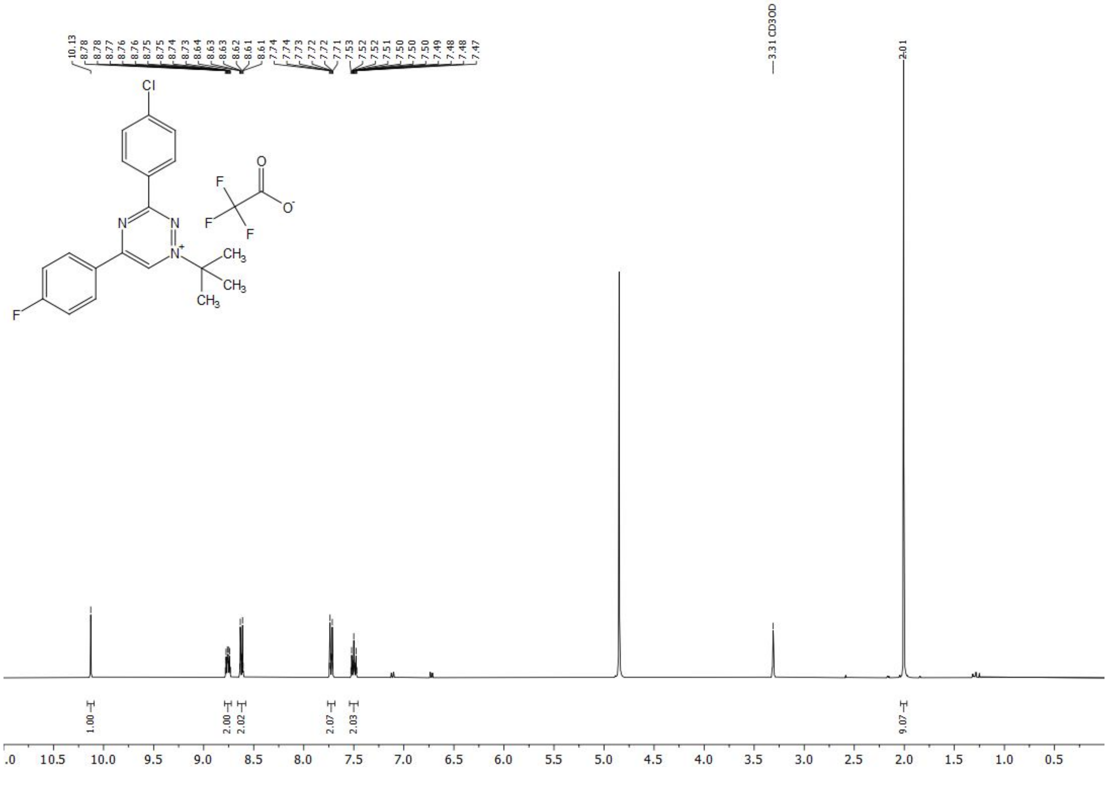

ArTrz$^+$2c ^{19}F NMR (376 MHz, MeOH-d_4)

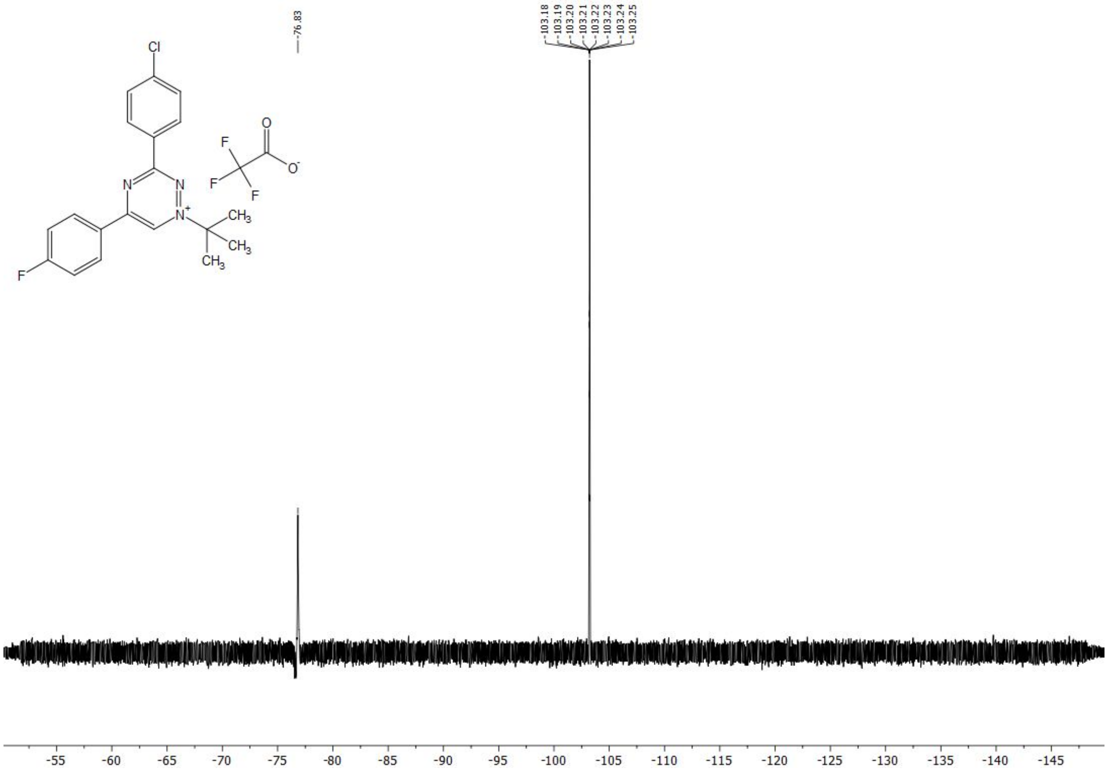

ArTrz$^+$2c ^{13}C NMR (101 MHz, MeOH-d_4)

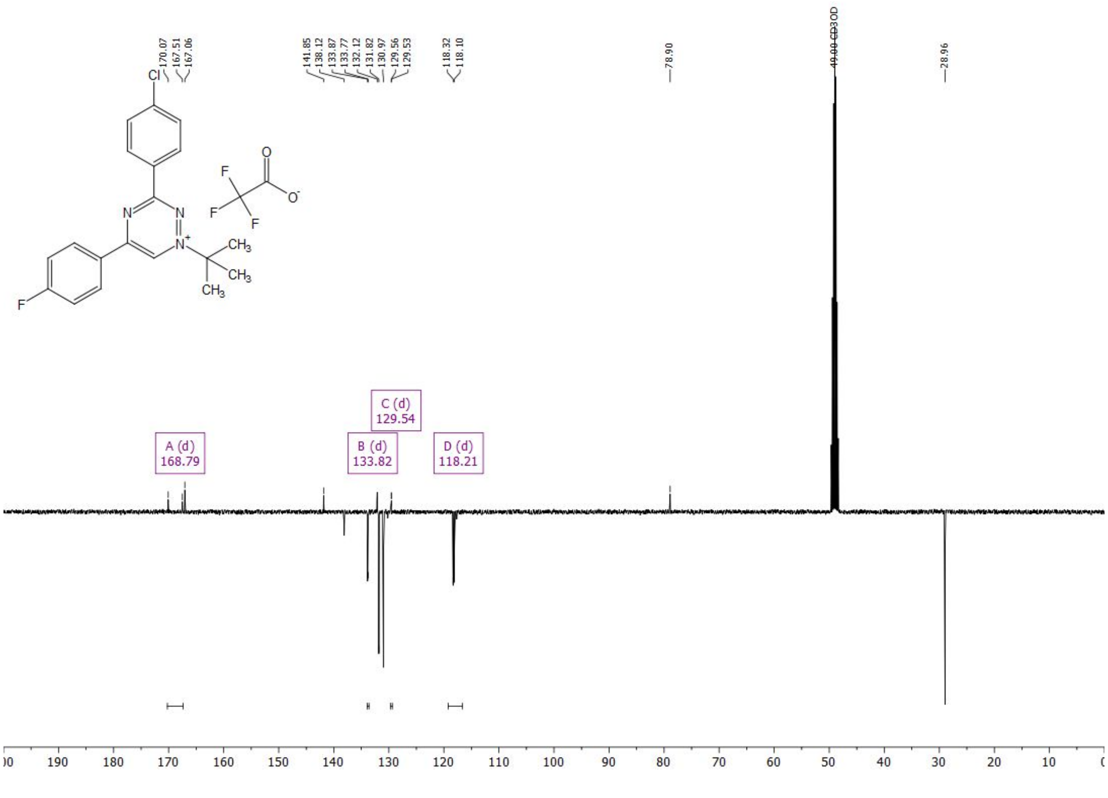

ArTrz^{+}2d ^{1}H NMR (400 MHz, CD_3CN)

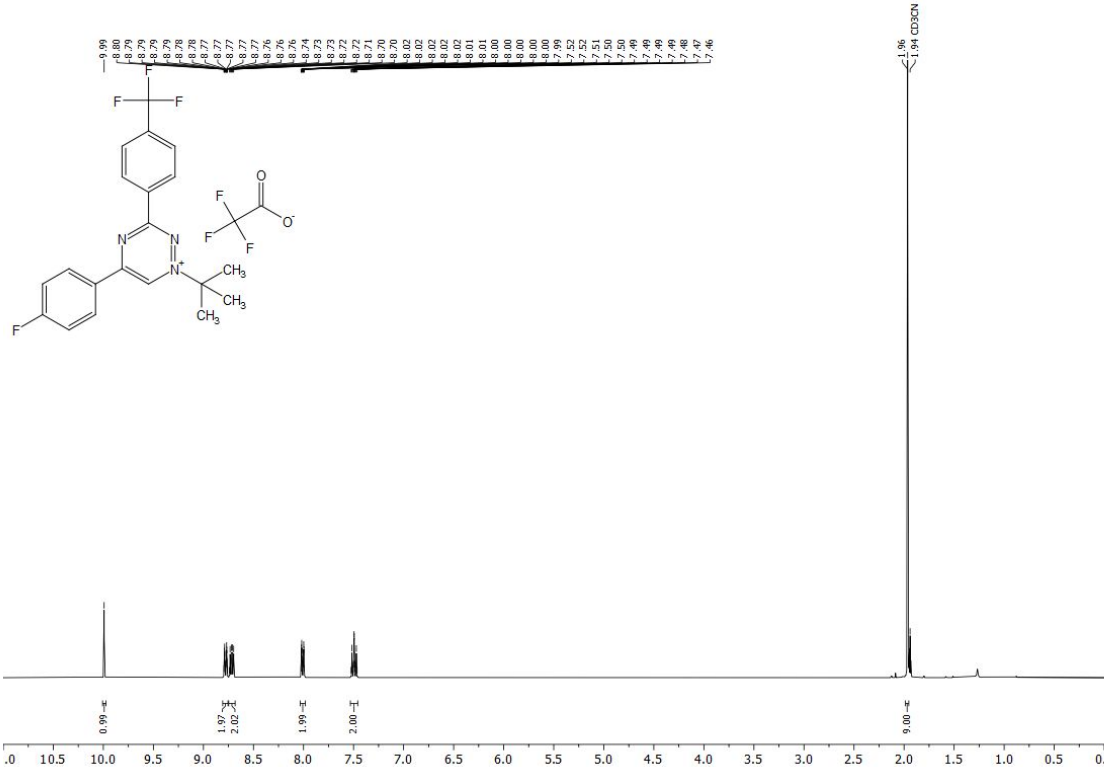

ArTrz^{+}2d ^{19}F NMR (376 MHz, CD_3CN)

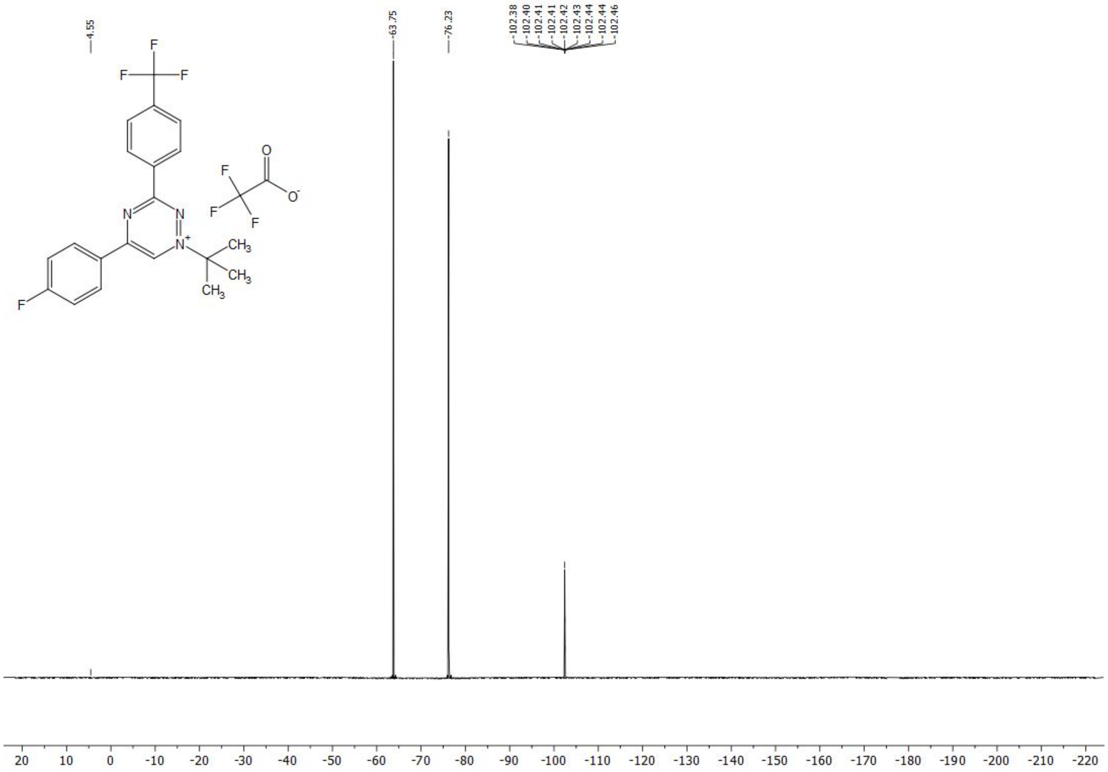

ArTrz⁺2d ^{13}C NMR (101 MHz, CD_3CN)

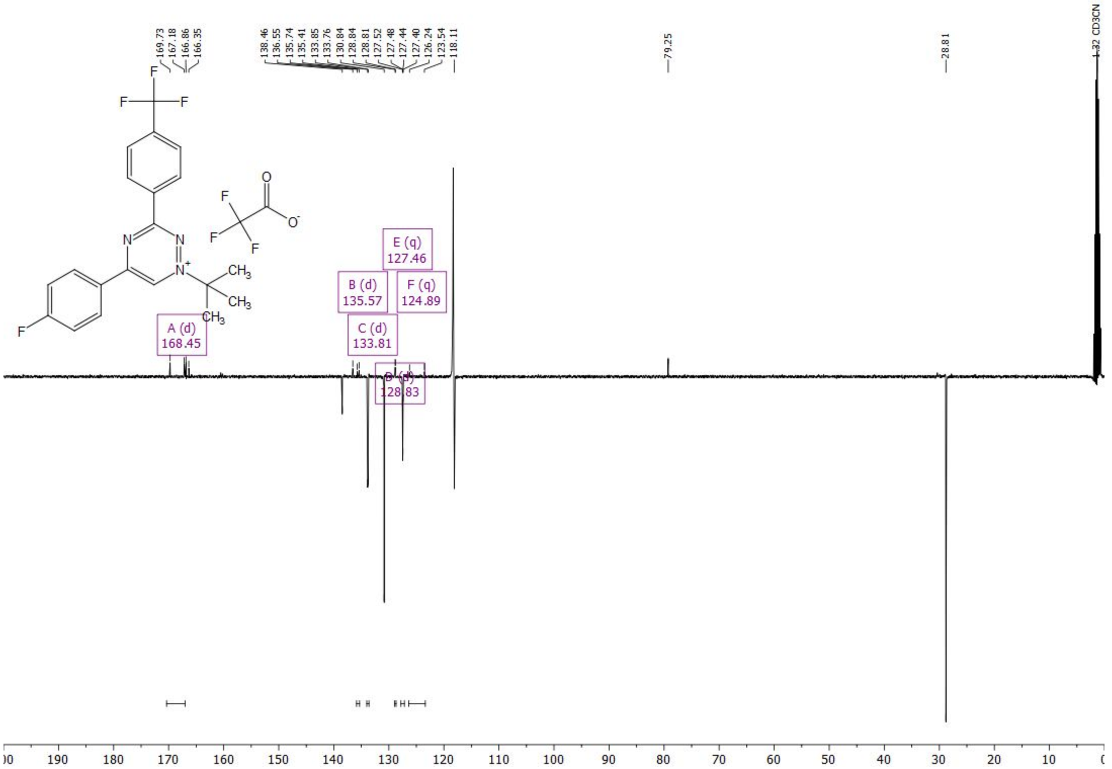

ArTrz⁺2e ^{1}H NMR (400 MHz, CD_3CN)

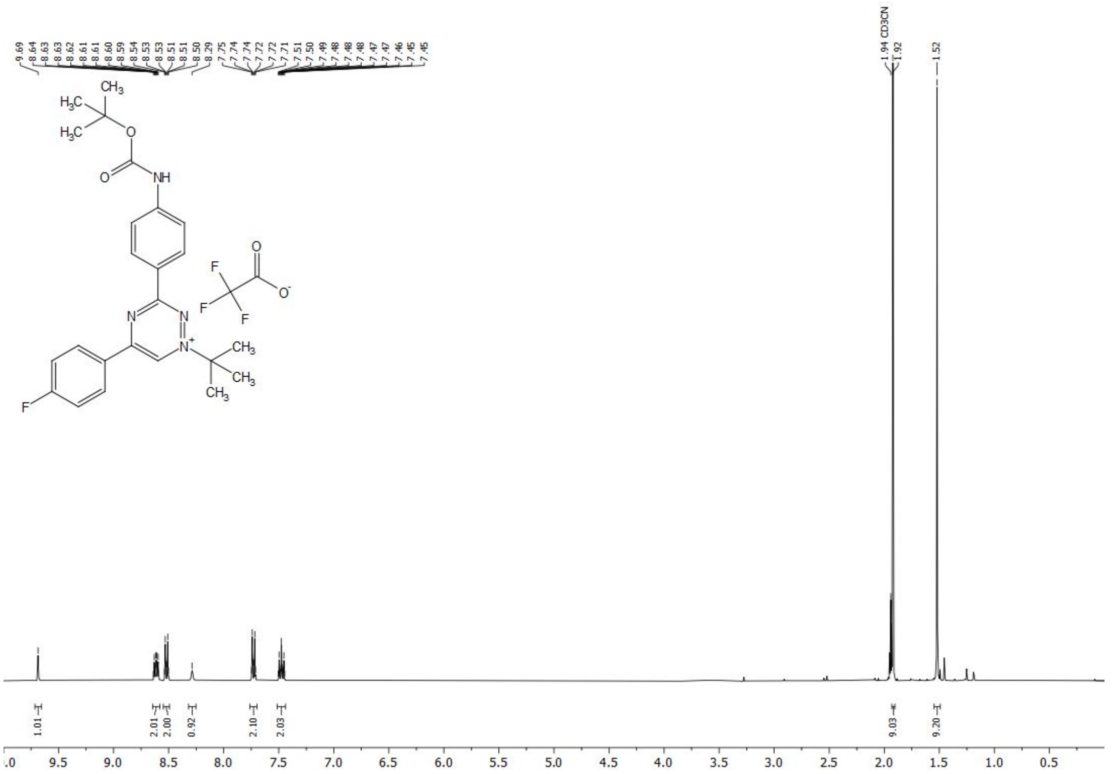

ArTrz⁺2e ^{19}F NMR (376 MHz, CD_3CN)

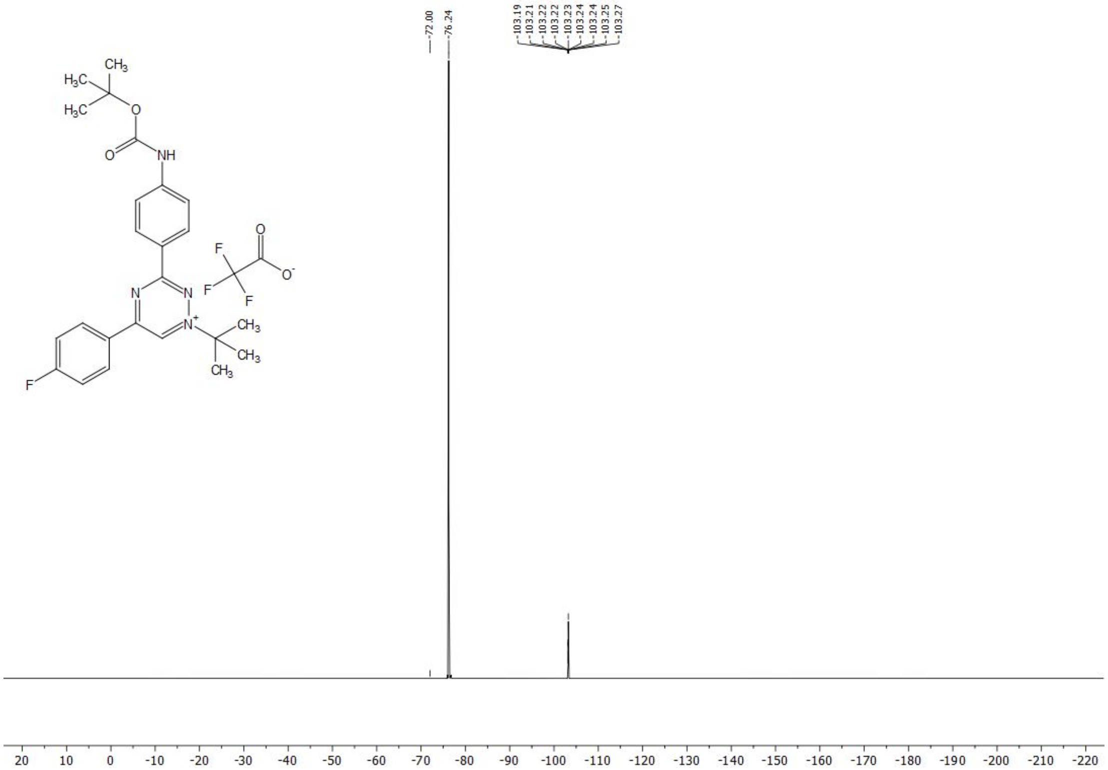

ArTrz⁺2e ^{13}C NMR (101 MHz, CD_3CN)

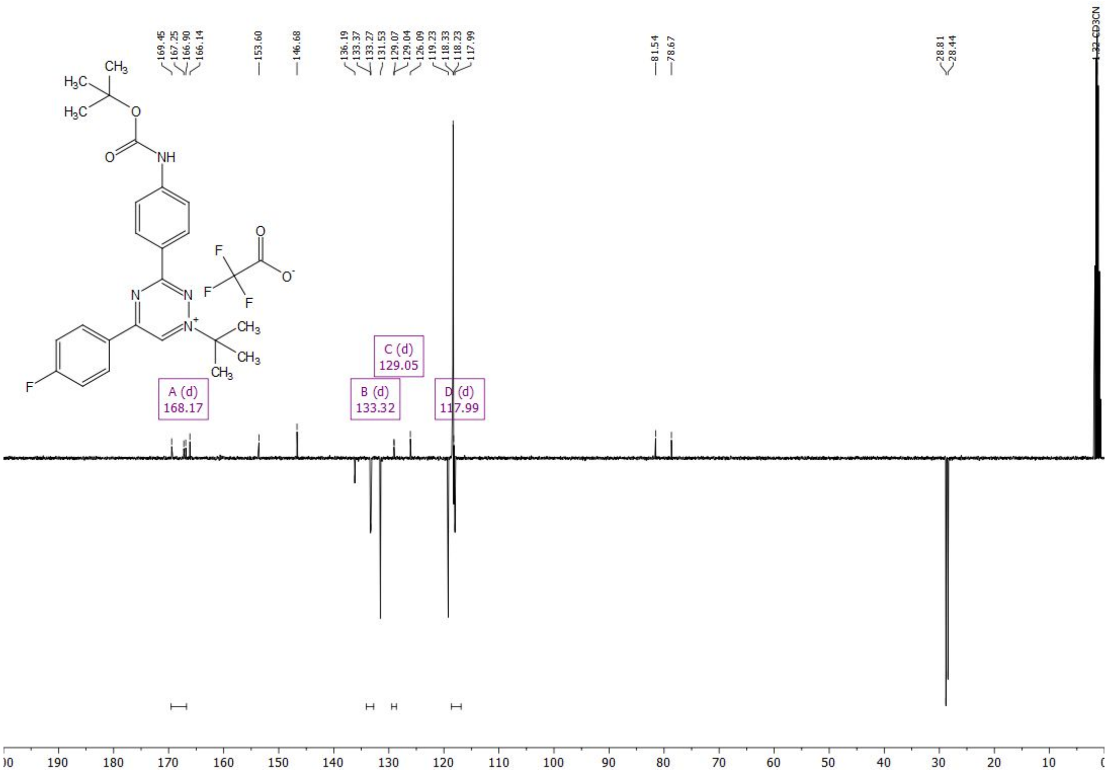

ArTrz⁺2f ¹H NMR (400 MHz, CD₃CN)

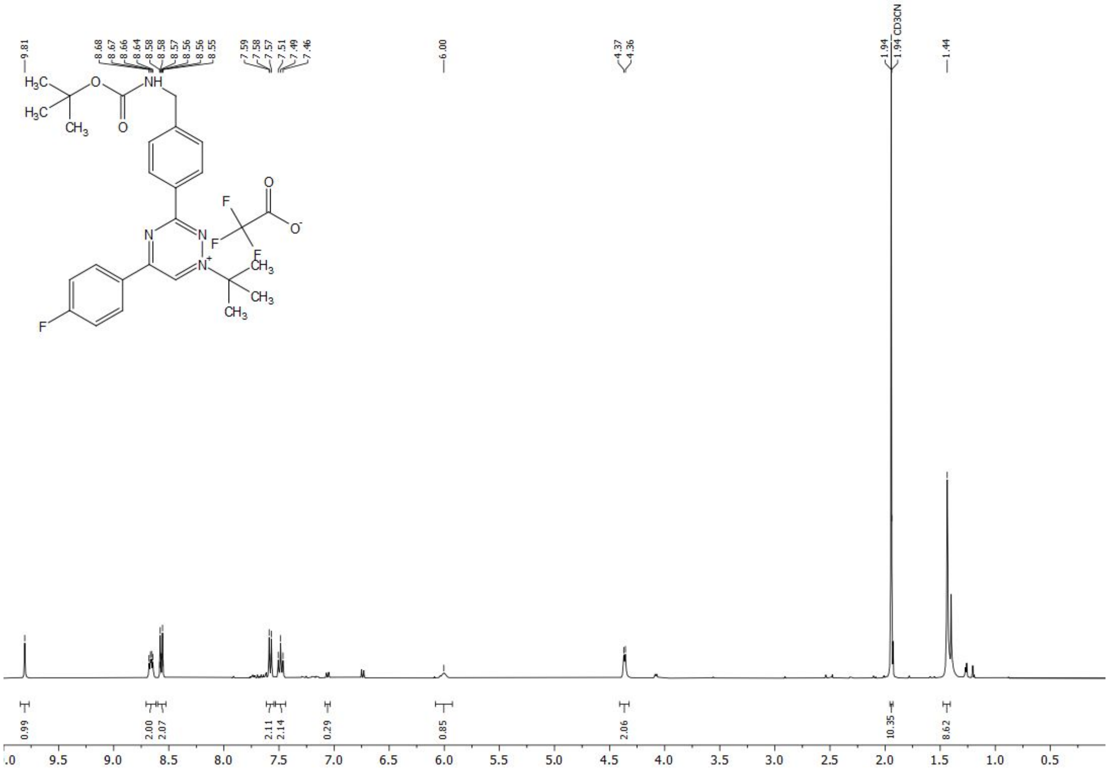

ArTrz⁺2f ¹⁹F NMR (376 MHz, CD₃CN)

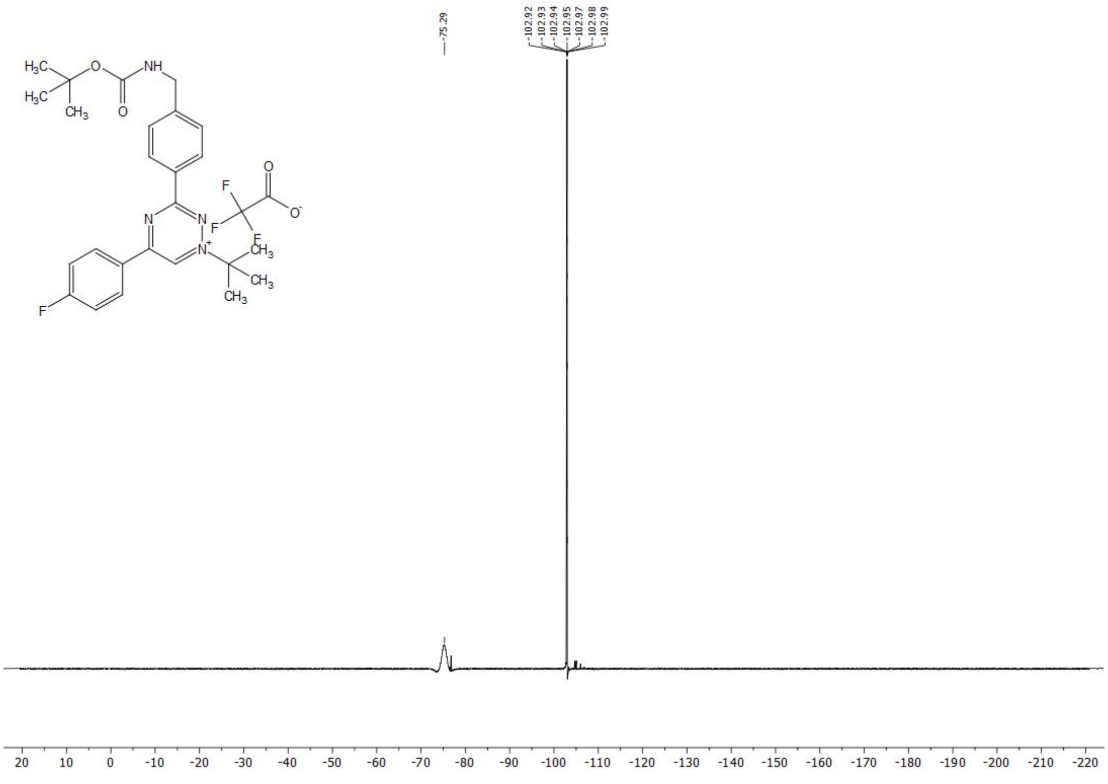

ArTrz⁺2f ^{13}C NMR (101 MHz, CD_3CN)

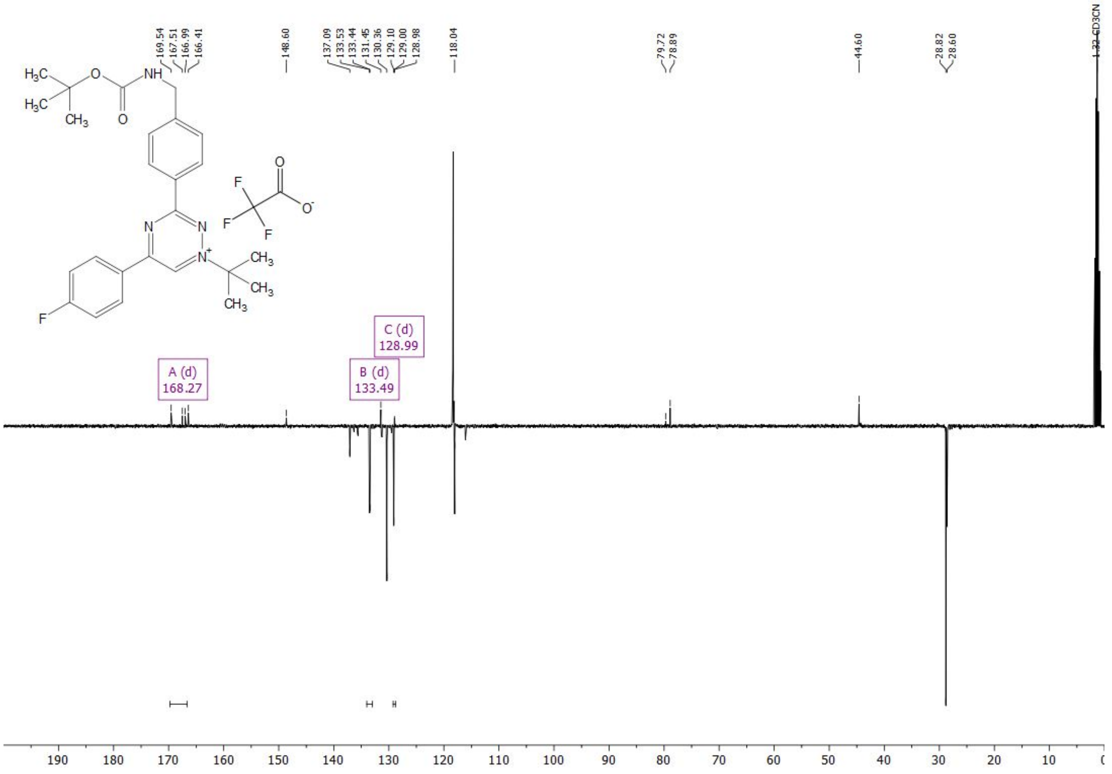

ArTrz⁺2g ^{1}H NMR (400 MHz, CD_3CN)

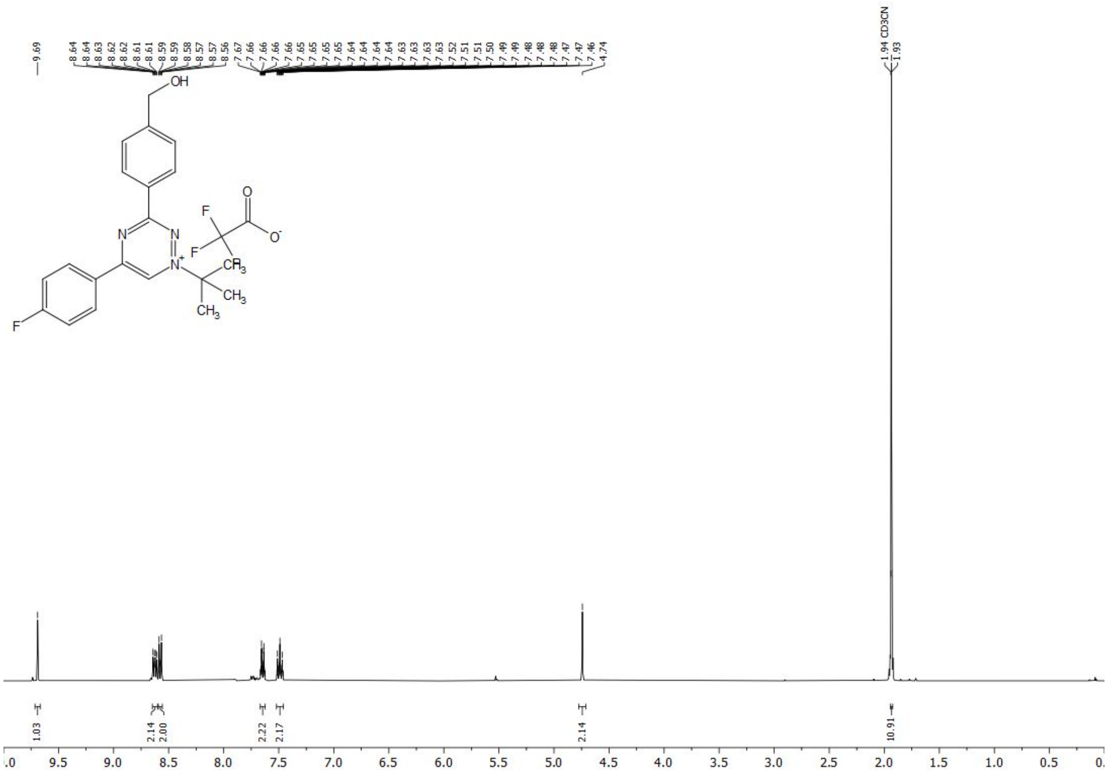

ArTrz+2g ^{19}F NMR (376 MHz, CD_3CN)

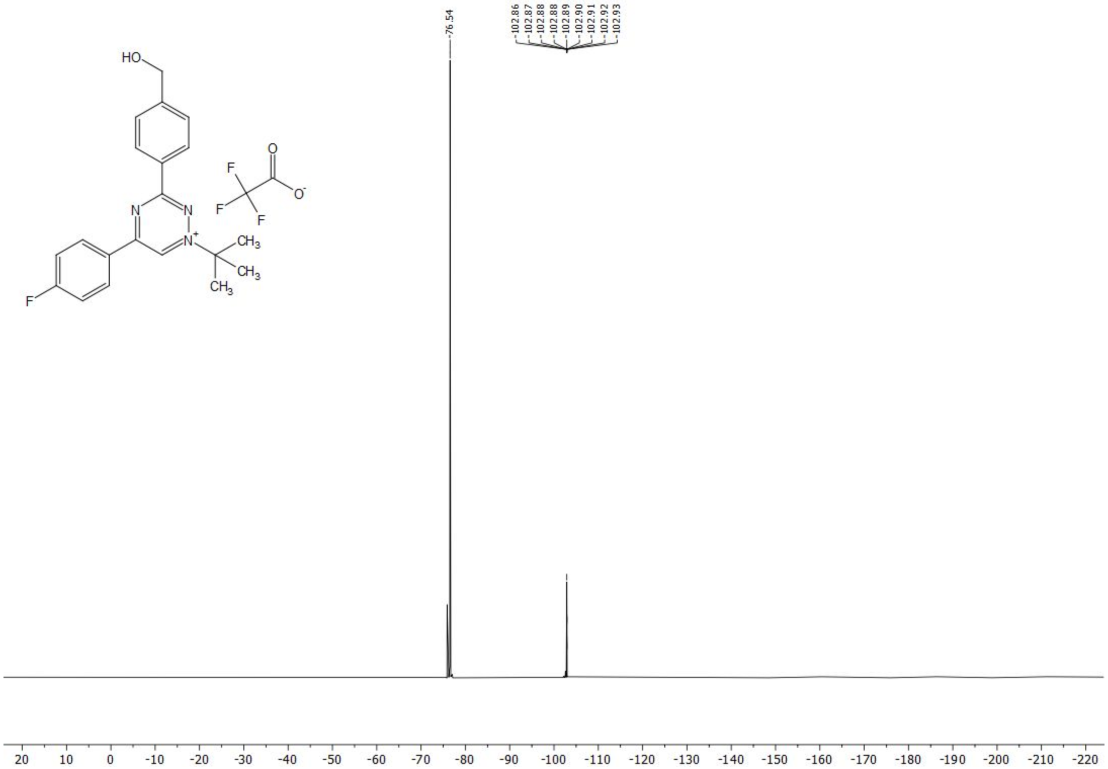

ArTrz+2g ^{13}C NMR (101 MHz, CD_3CN)

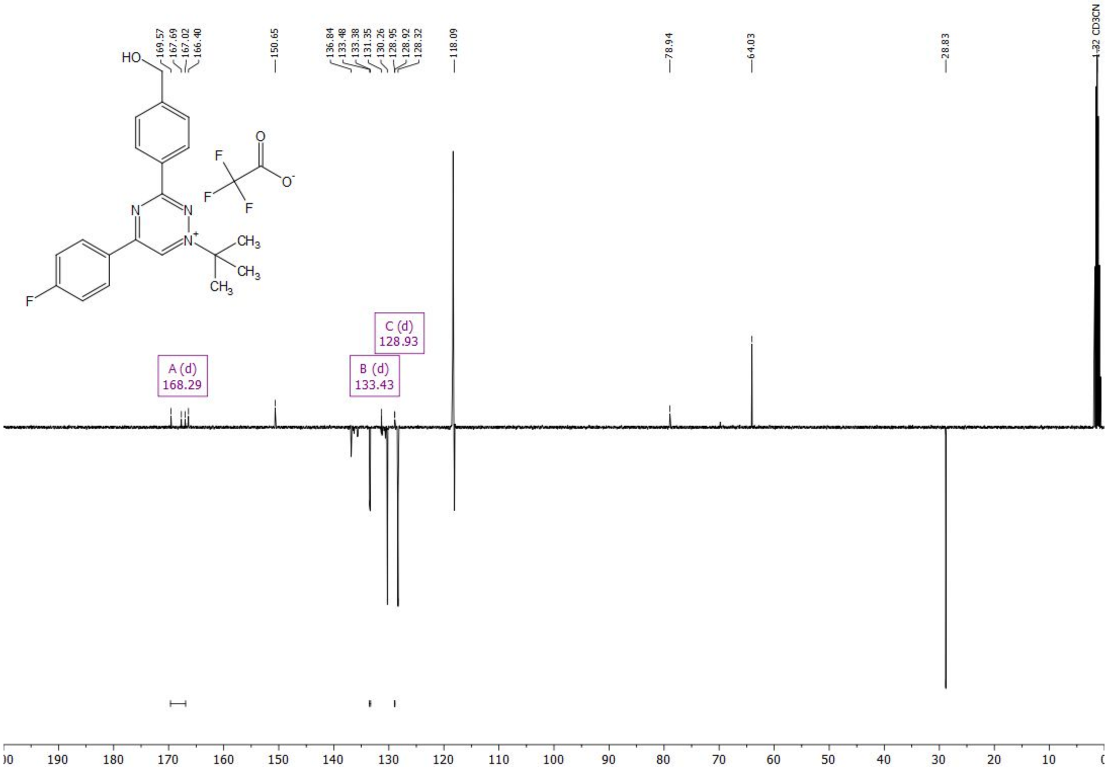

ArTrz⁺2h ^{1}H NMR (400 MHz, CD_3CN)

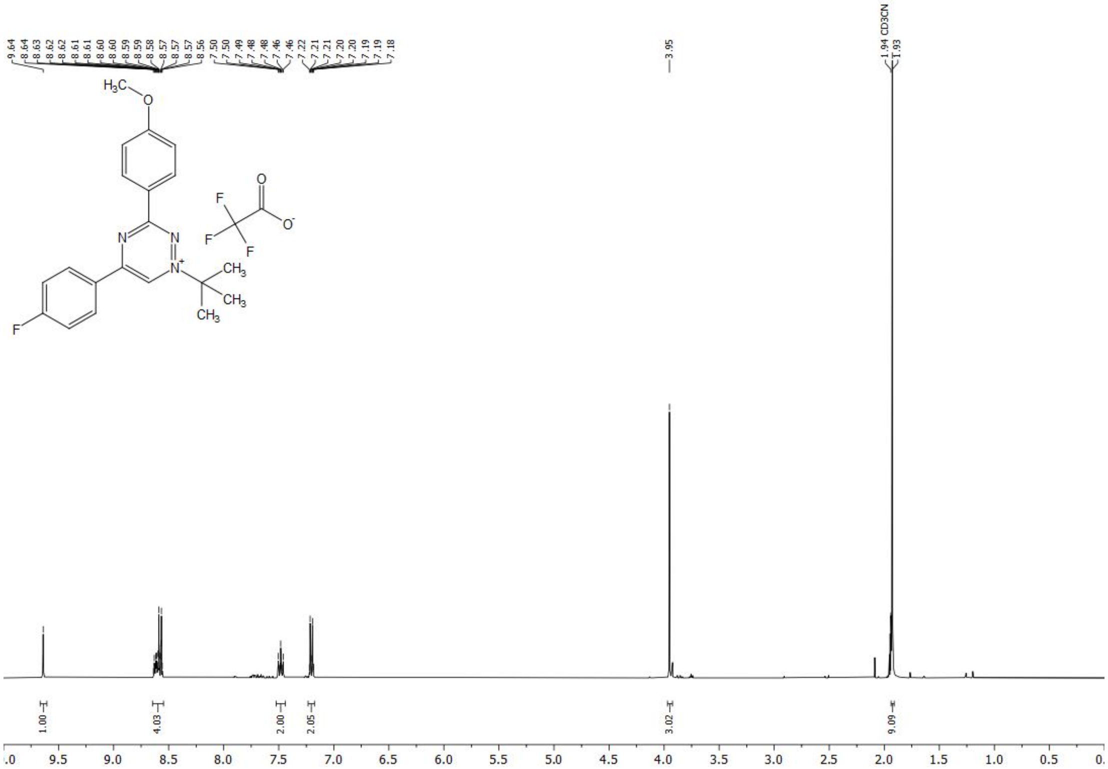

ArTrz⁺2h ^{19}F NMR (376 MHz, CD_3CN)

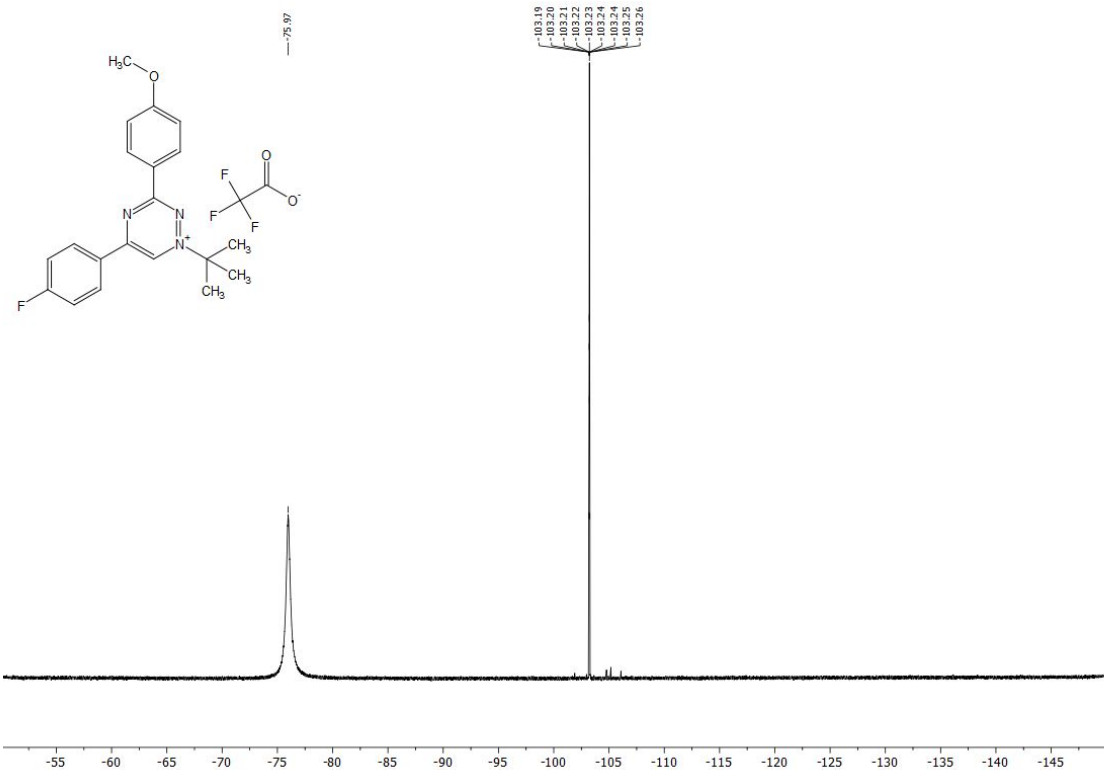

ArTrz⁺2h ^{13}C NMR (101 MHz, CD_3CN)

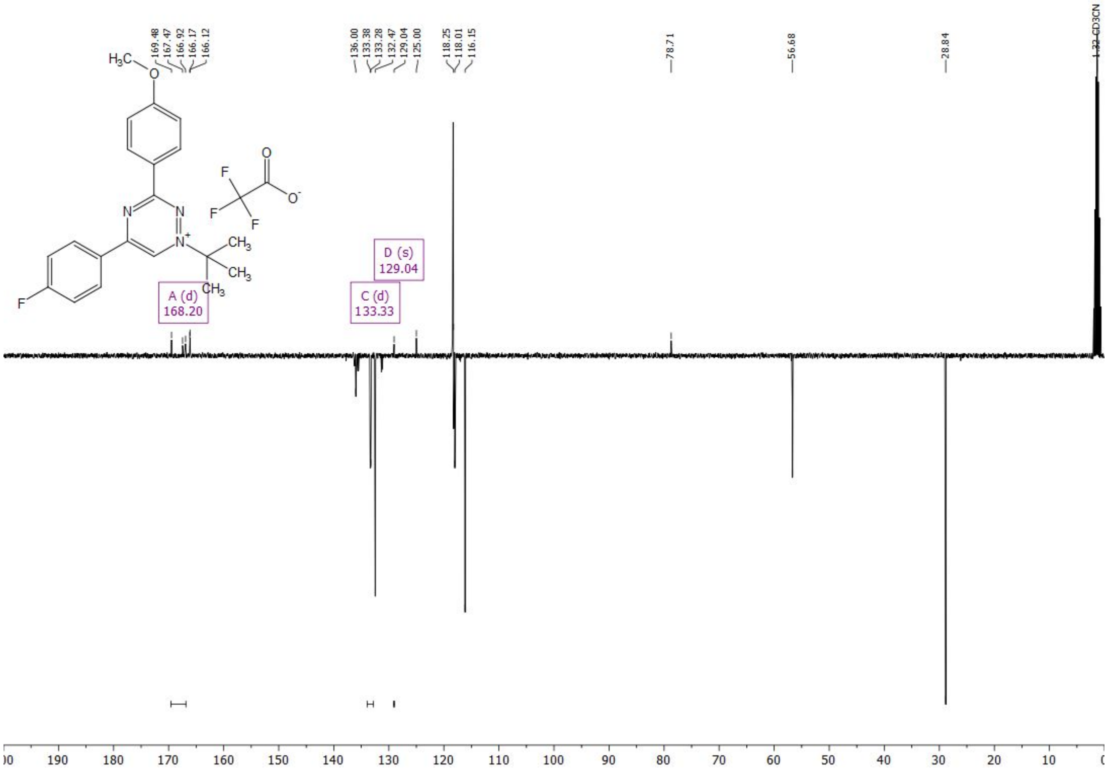

ArTrz⁺2i ^{1}H NMR (400 MHz, CD_3CN)

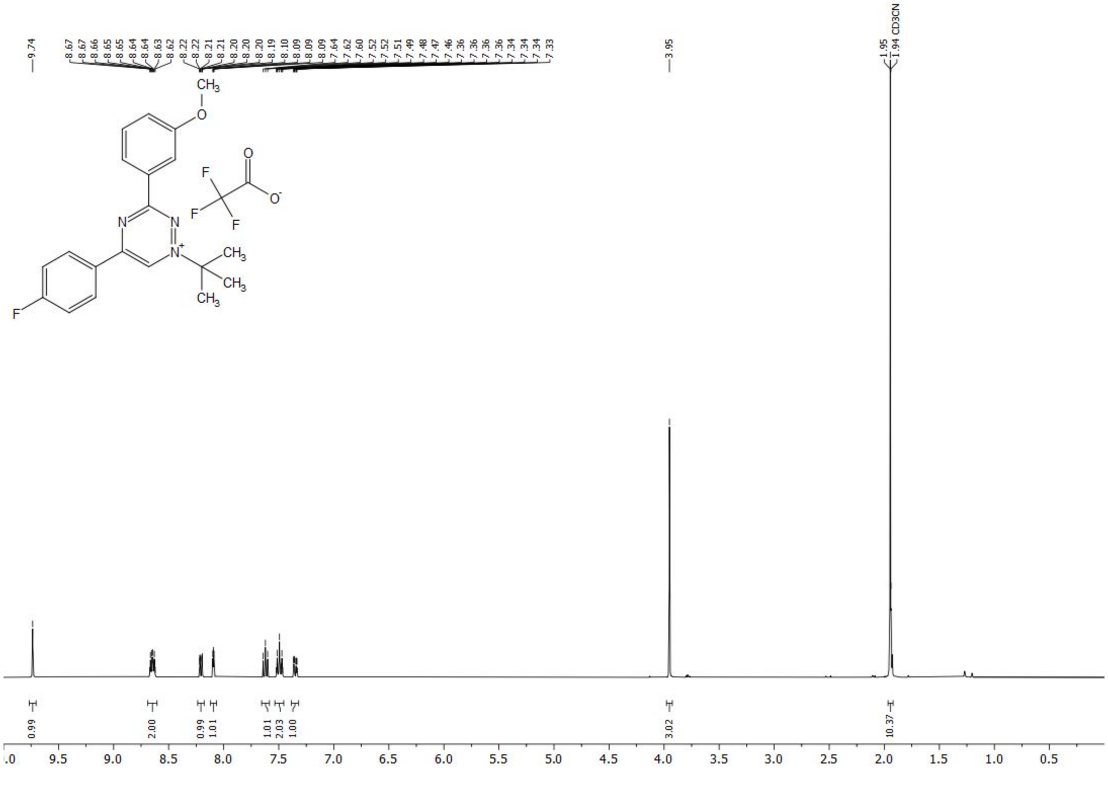

ArTrz⁺2i ^{19}F NMR (376 MHz, CD_3CN)

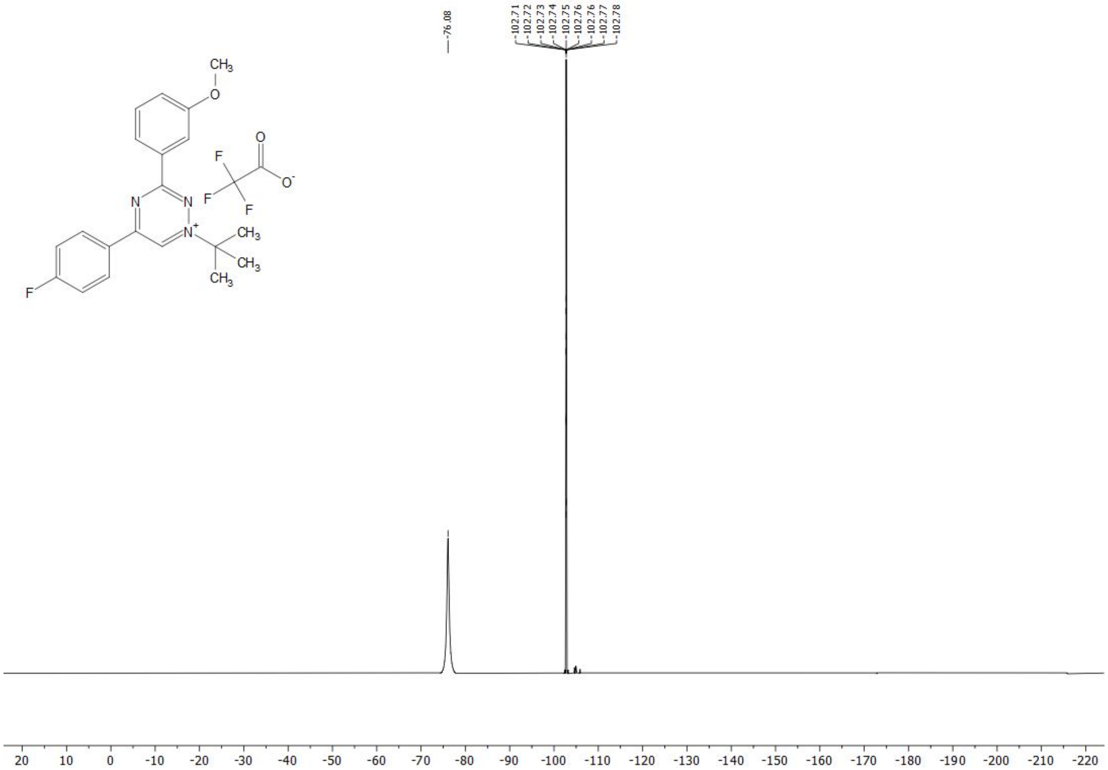

ArTrz⁺2i ^{13}C NMR (101 MHz, CD_3CN)

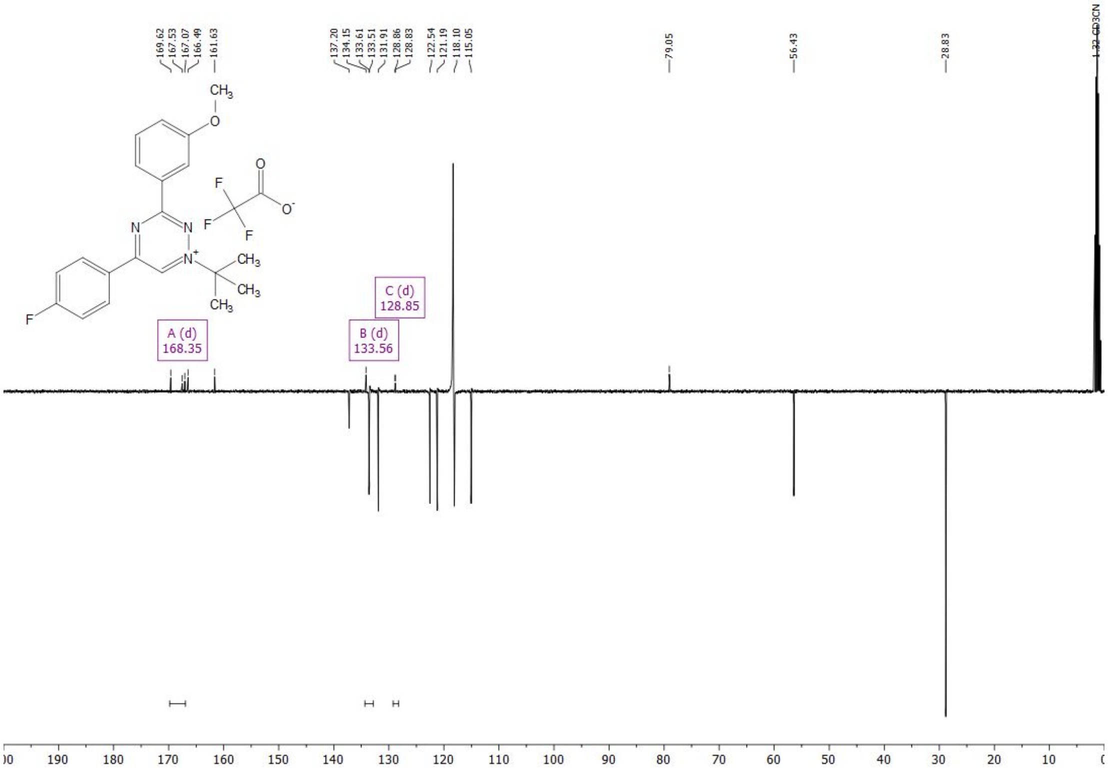

ArTrz⁺2j ^{1}H NMR (400 MHz, CD_3CN)

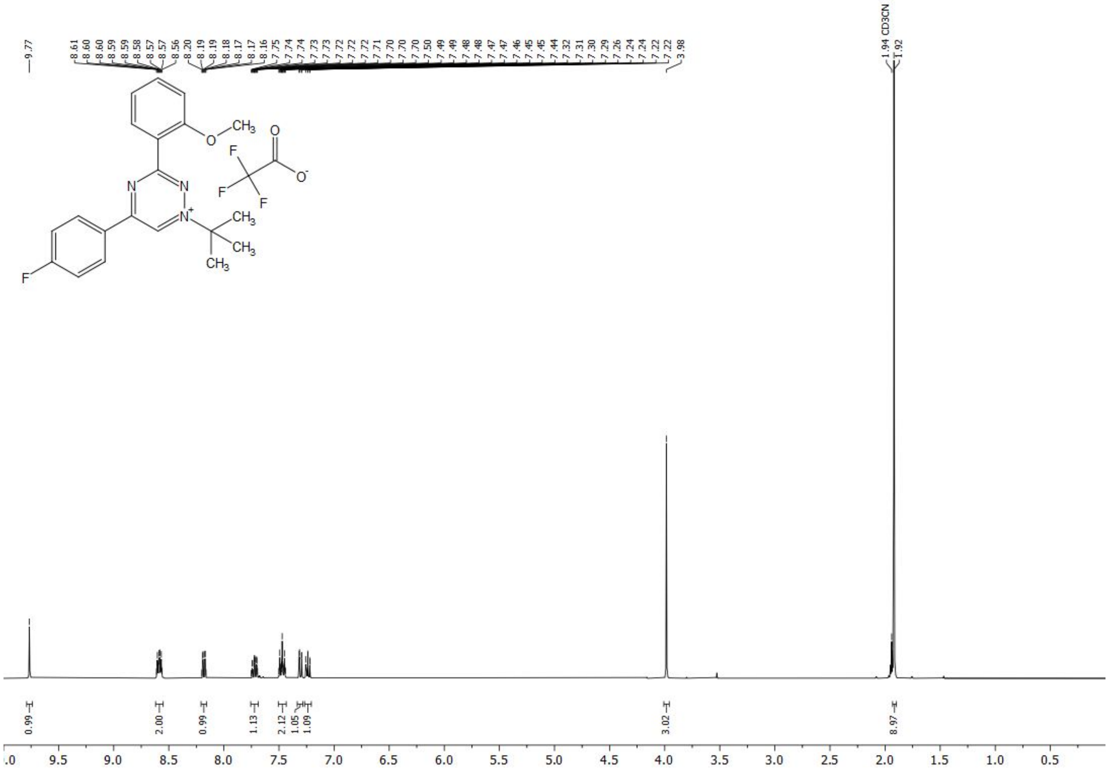

ArTrz⁺2j ^{19}F NMR (376 MHz, CD_3CN)

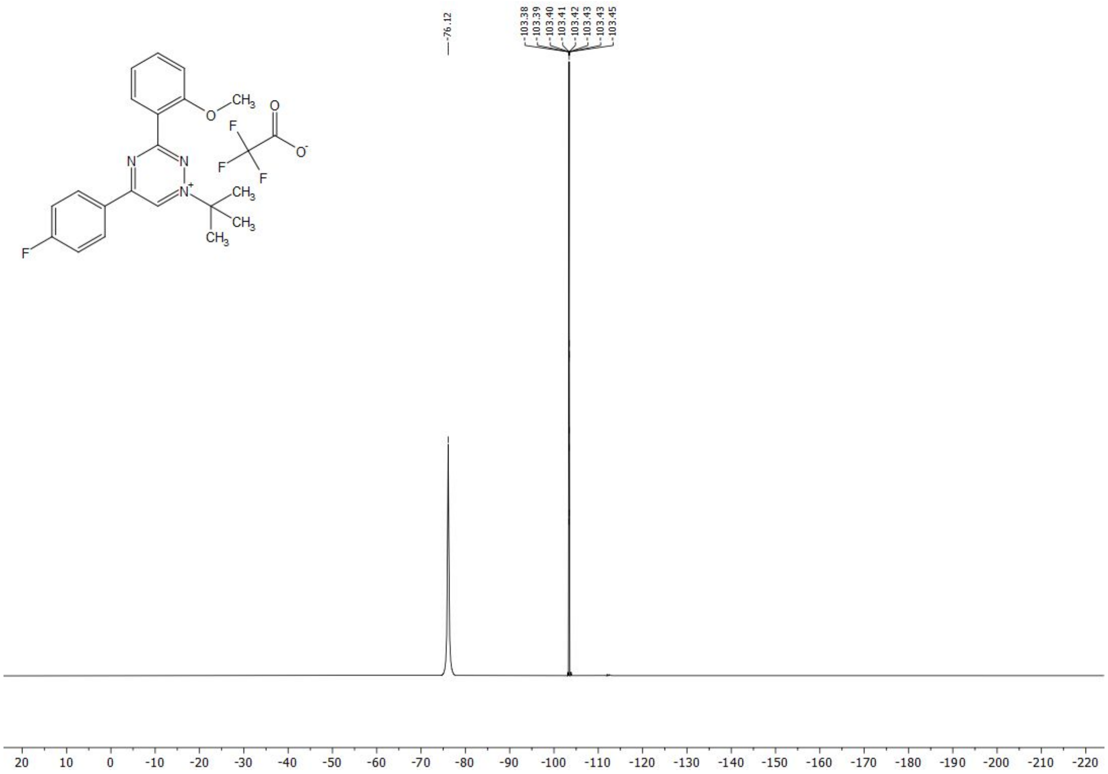

ArTrz⁺2j ^{13}C NMR (101 MHz, CD_3CN)

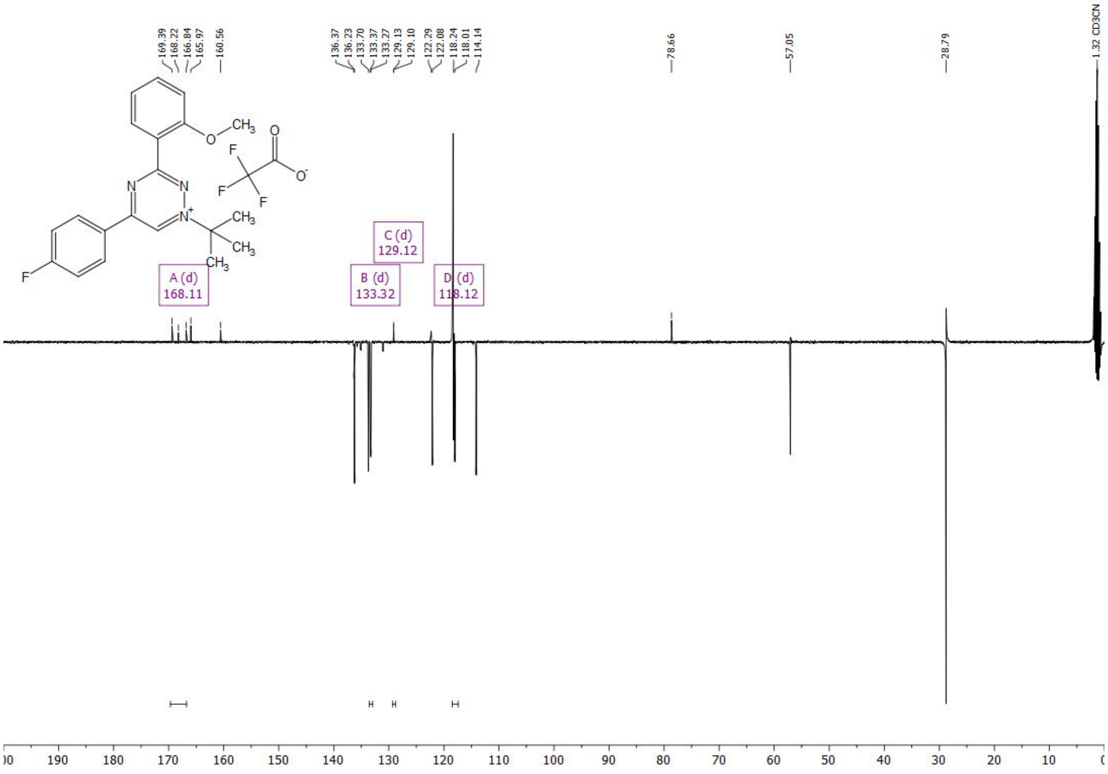

ArTrz⁺2k ^{1}H NMR (400 MHz, MeOH-d_4)

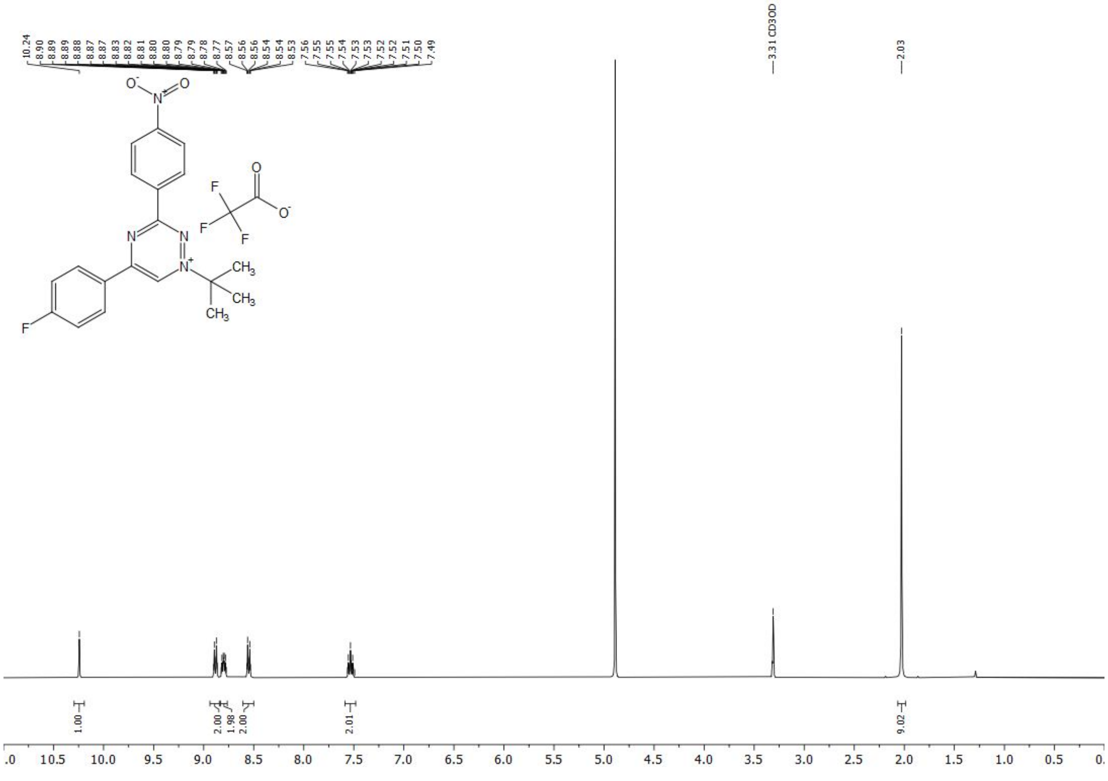

ArTrz⁺2k ^{19}F NMR (376 MHz, MeOH-d_4)

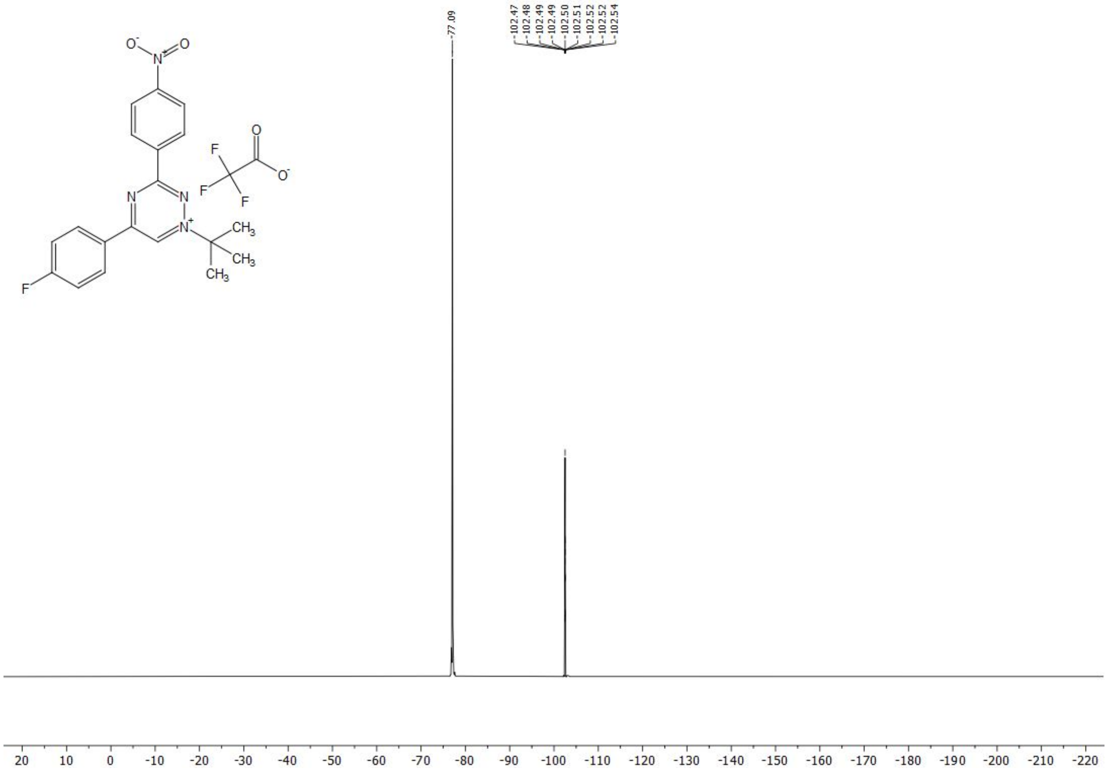

ArTrz⁺2k ^{13}C NMR (101 MHz, MeOH-d_4)

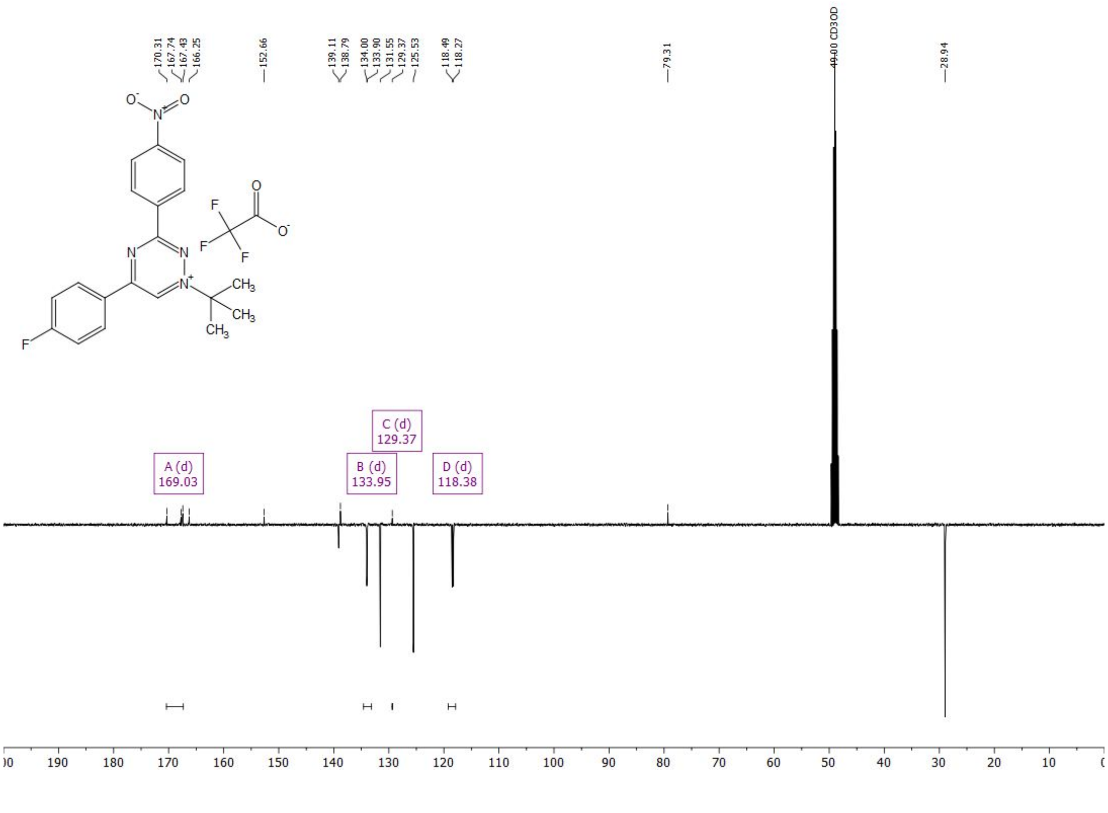

ArTrz⁺2l ^{1}H NMR (400 MHz, CD_3CN)

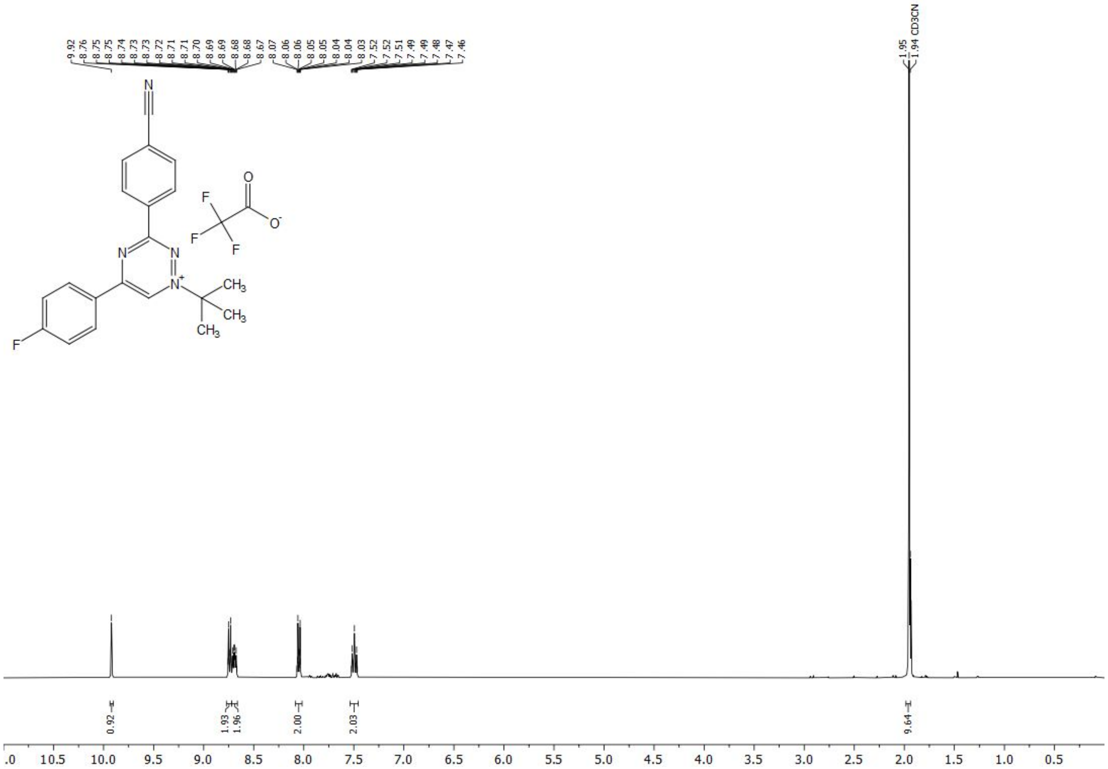

ArTrz⁺2l ^{19}F NMR (376 MHz, CD_3CN)

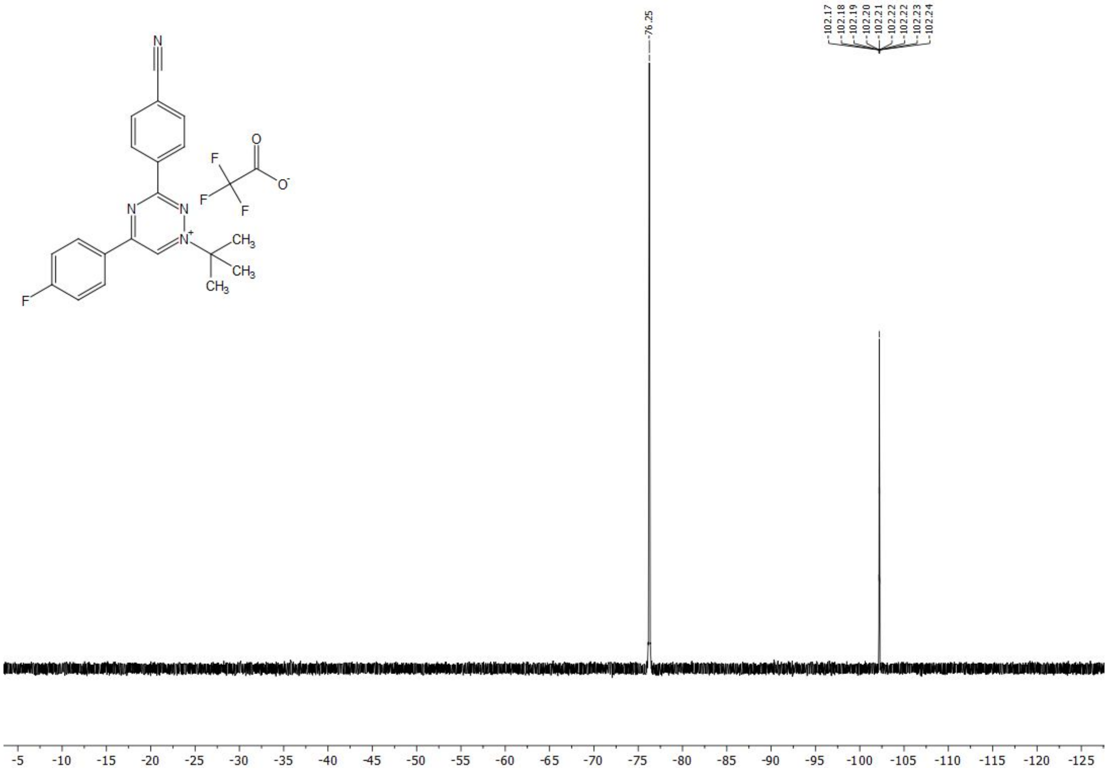

ArTrz⁺2l ^{13}C NMR (101 MHz, CD_3CN)

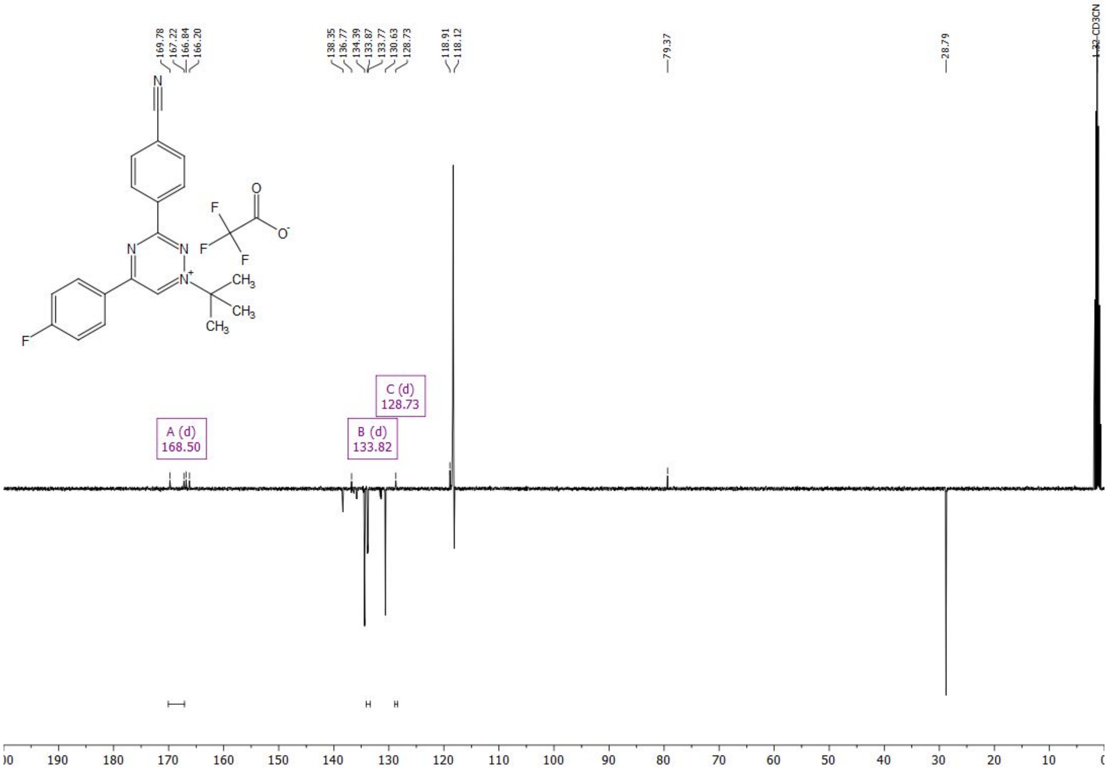

ArTrz⁺2m ^{1}H NMR (400 MHz, MeOH-d_4)

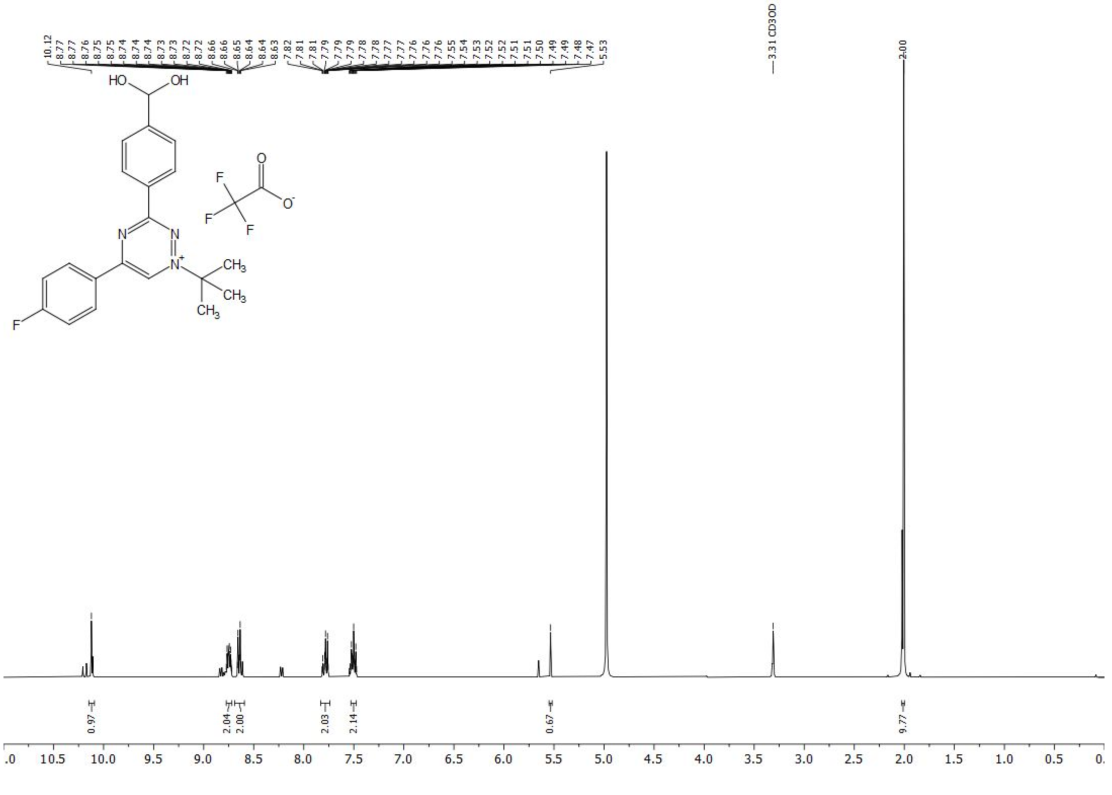

ArTrz⁺2m ^{19}F NMR (376 MHz, MeOH-d_4)

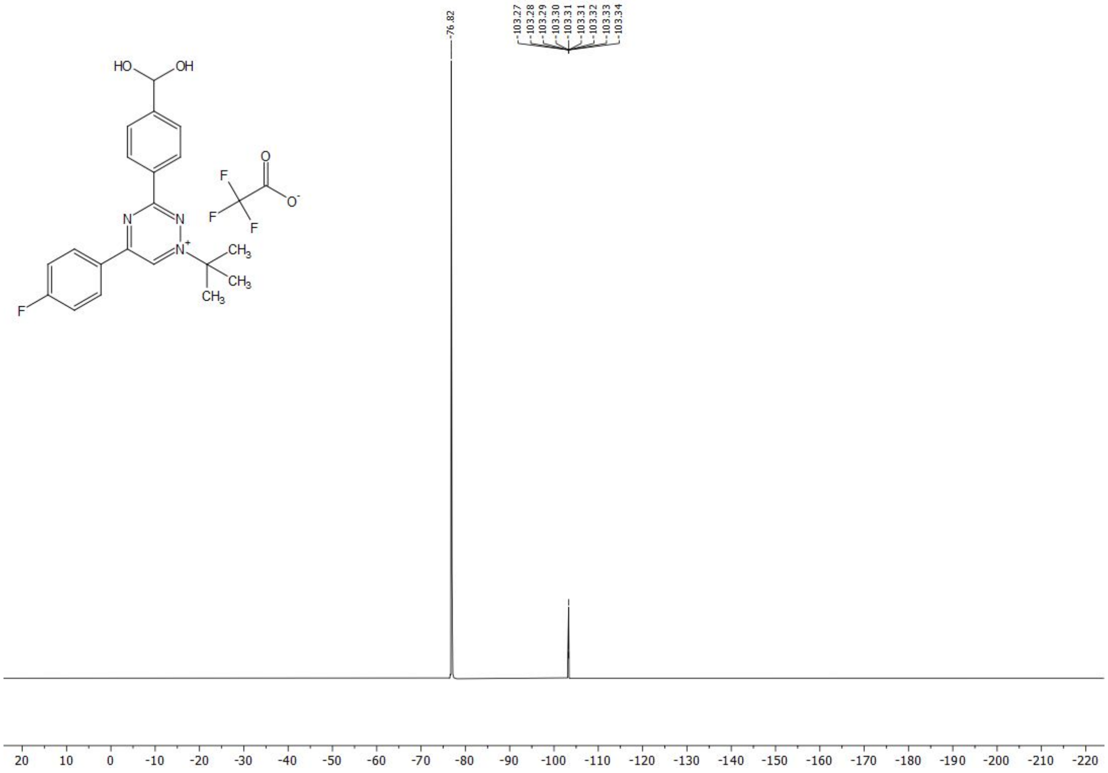

ArTrz⁺2m ^{13}C NMR (101 MHz, MeOH-d_4)

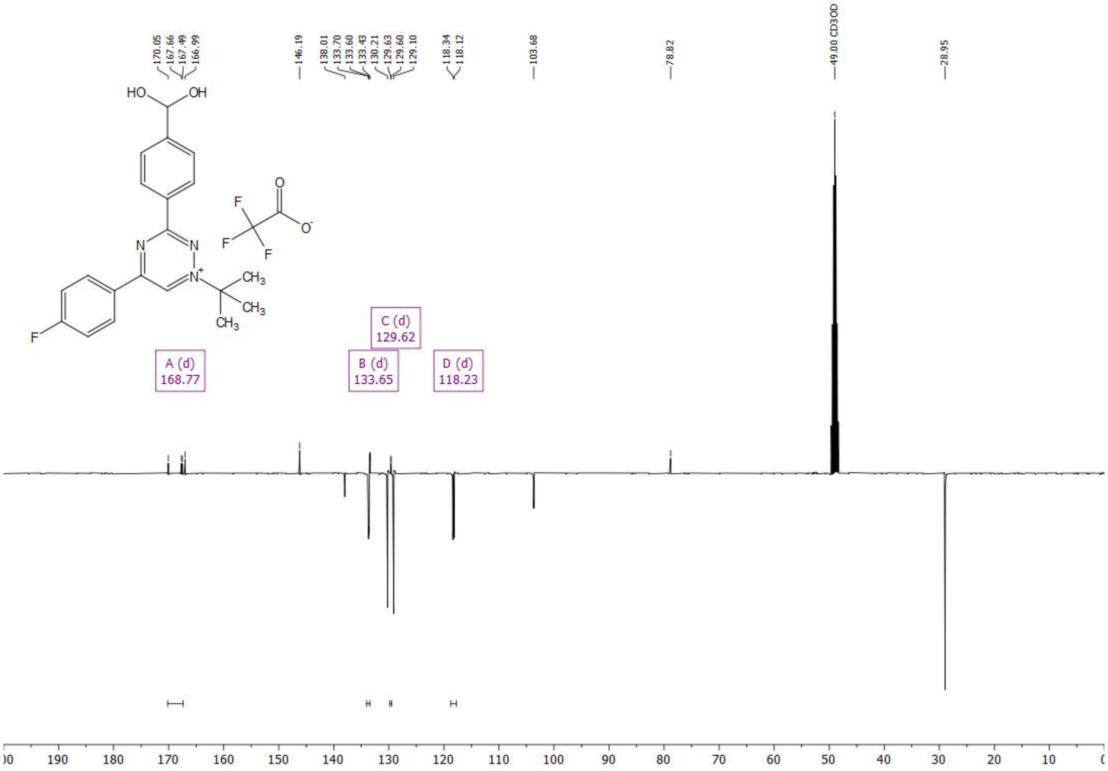

ArTrz+2n ^{1}H NMR (400 MHz, CD_3CN)

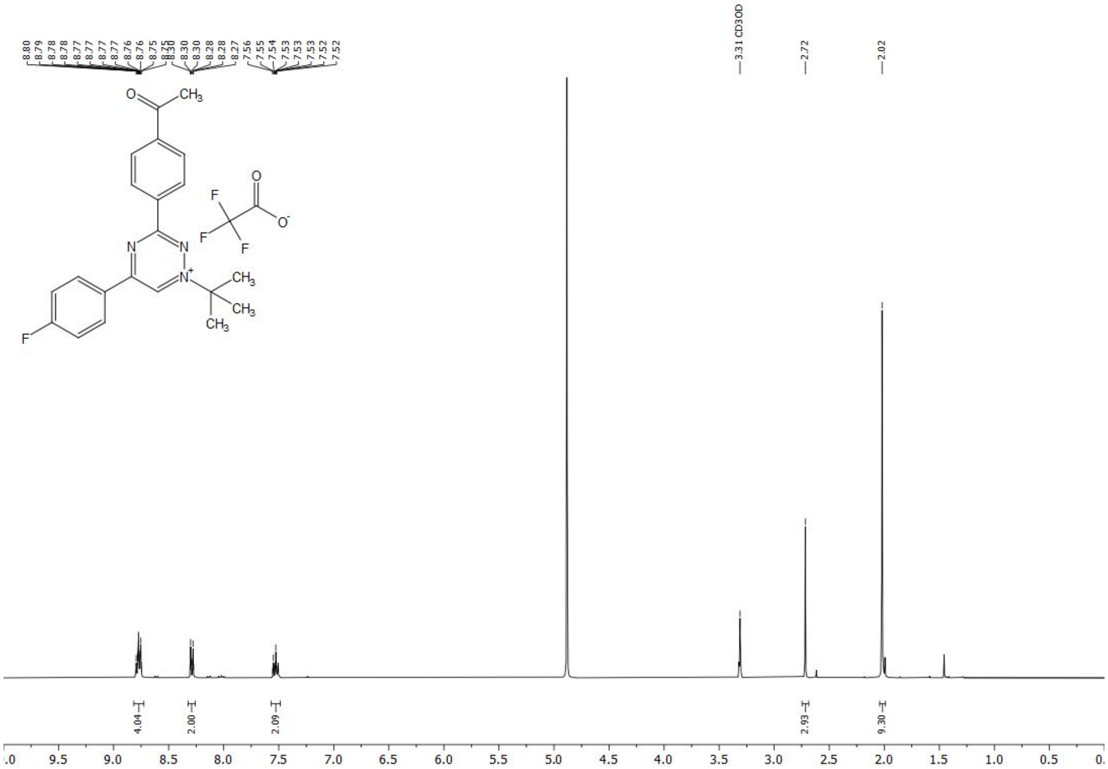

ArTrz+2n ^{19}F NMR (376 MHz, CD_3CN)

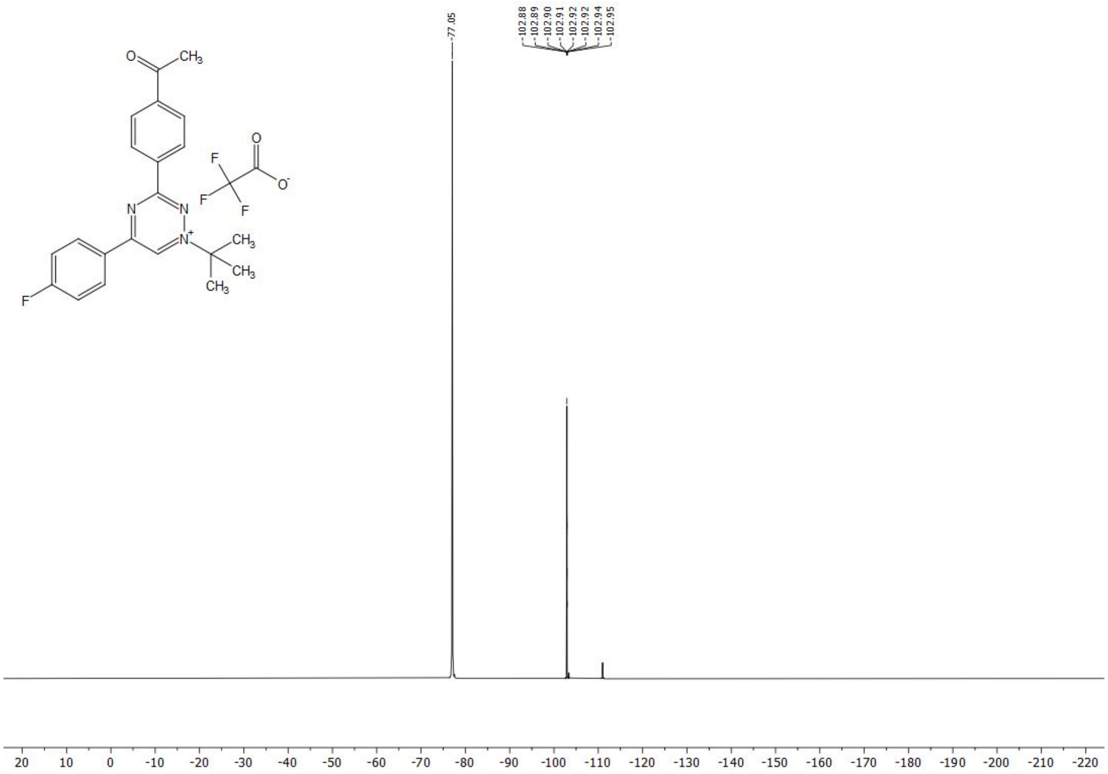

ArTrz+2n (ketal equilibration) ^{1}H NMR (400 MHz, CD_3CN)

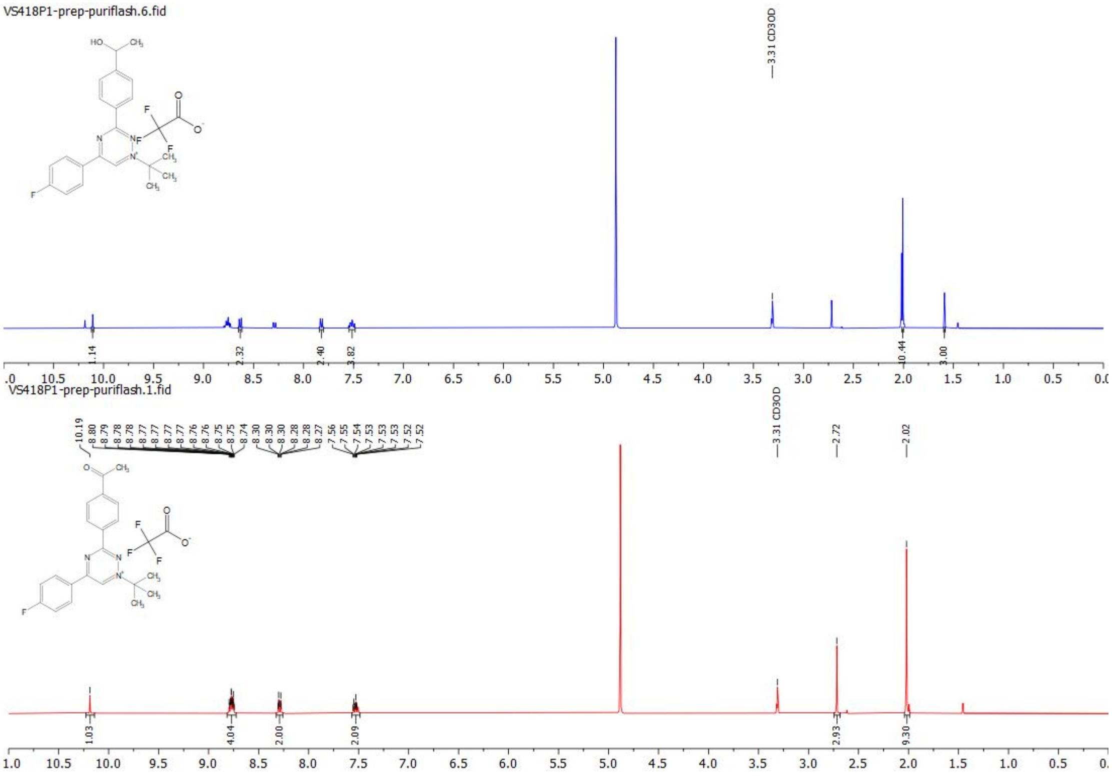

ArTrz+2o ^{1}H NMR (400 MHz, MeOH-d_4)

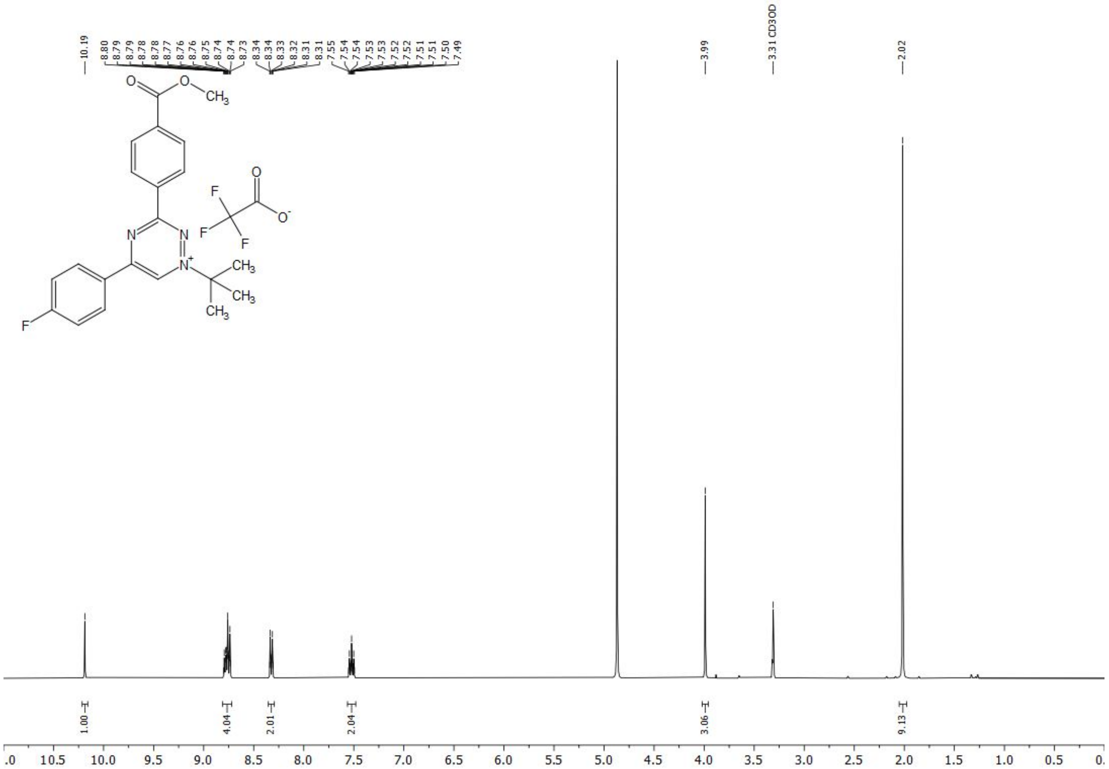

ArTrz+2o ^{19}F NMR (376 MHz, MeOH-d_4)

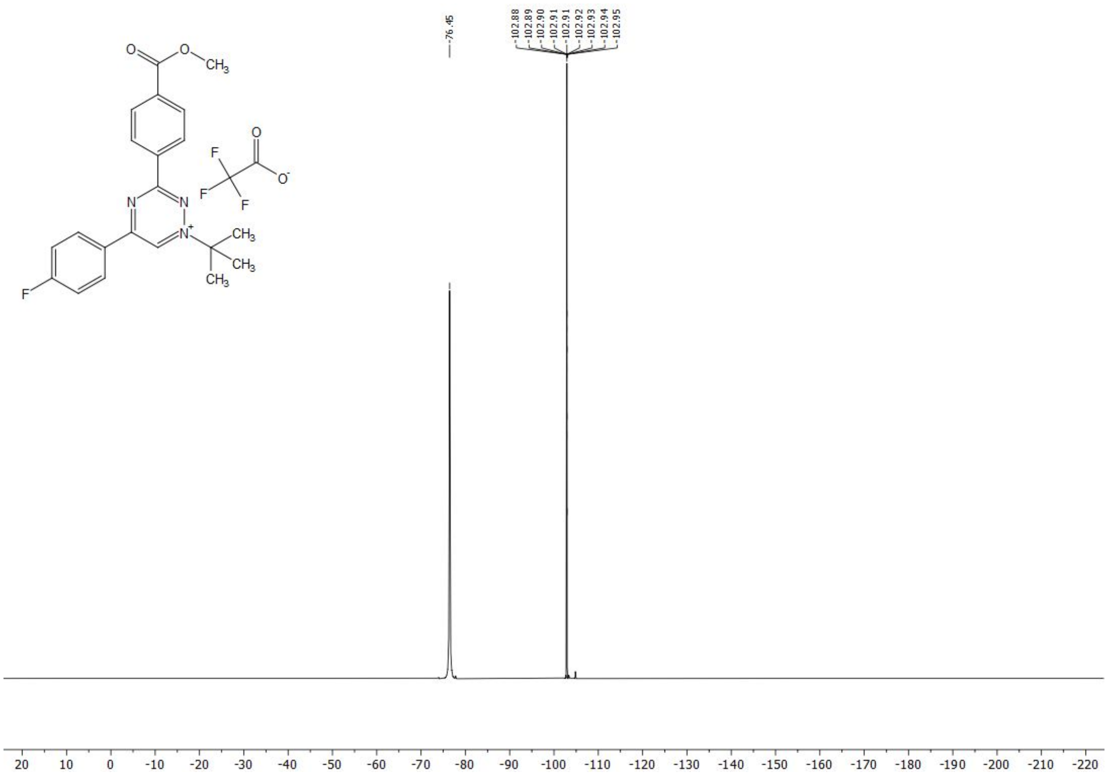

ArTrz+2o ^{13}C NMR (101 MHz, MeOH-d_4)

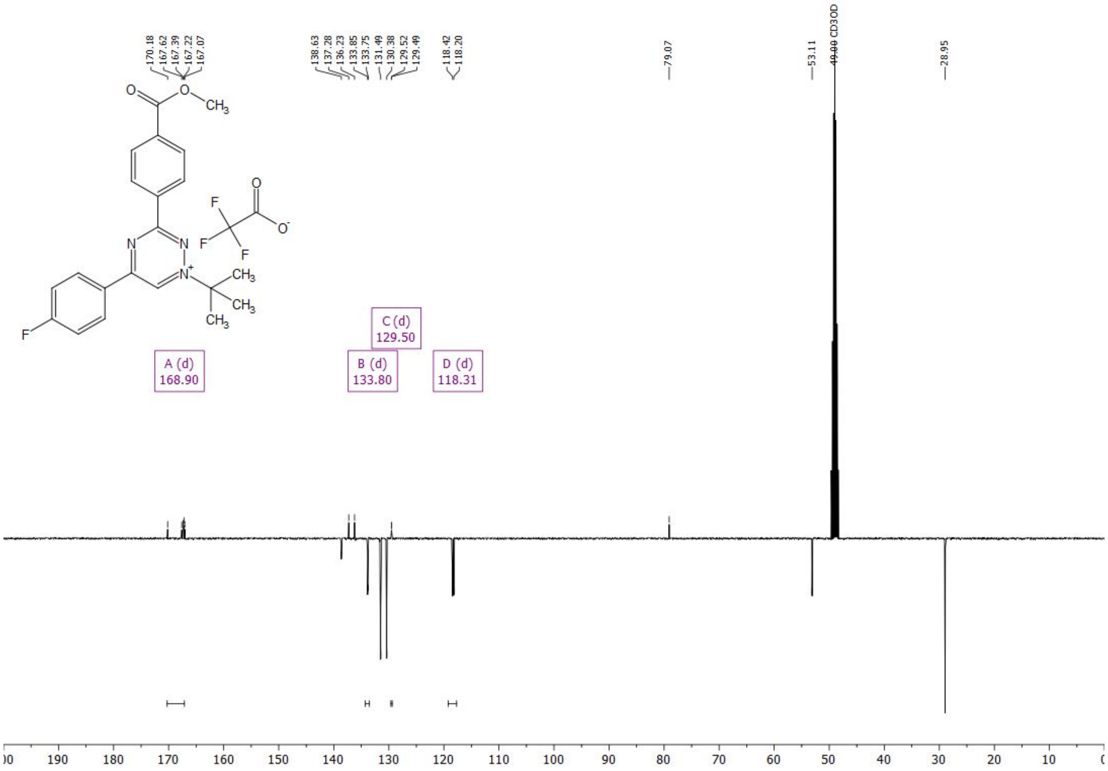

ArTrz⁺2p ^{1}H NMR (400 MHz, CD_3CN)

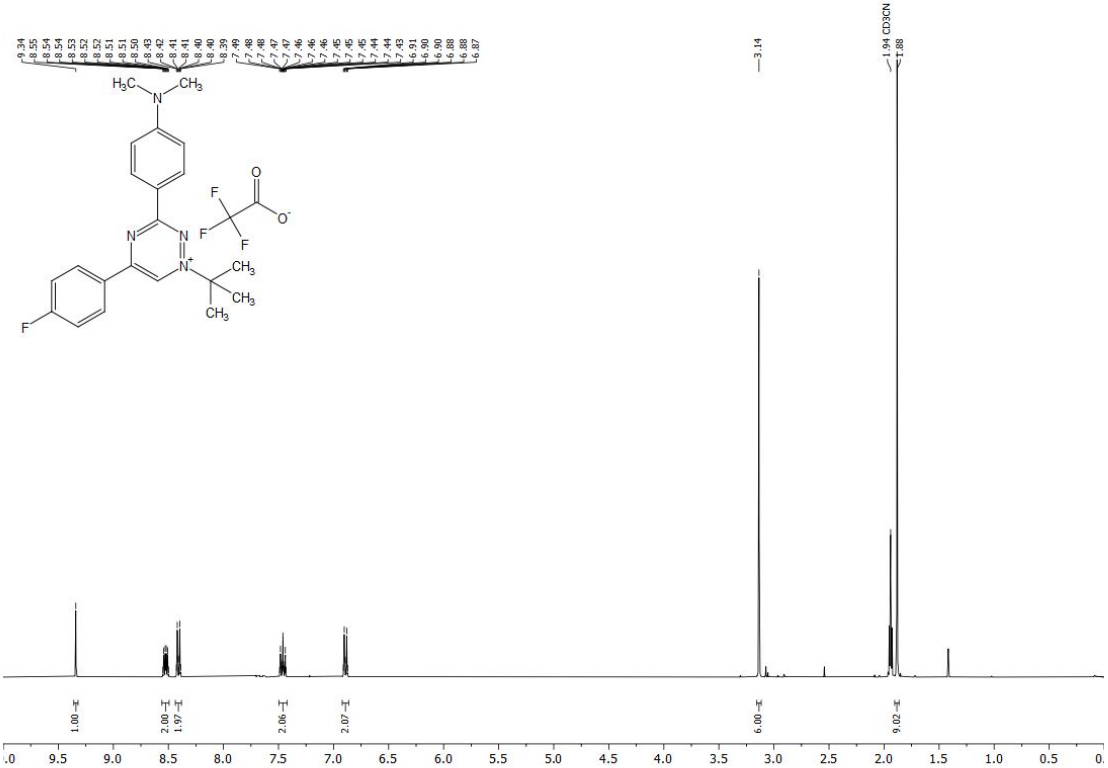

ArTrz⁺2p ^{19}F NMR (376 MHz, CD_3CN)

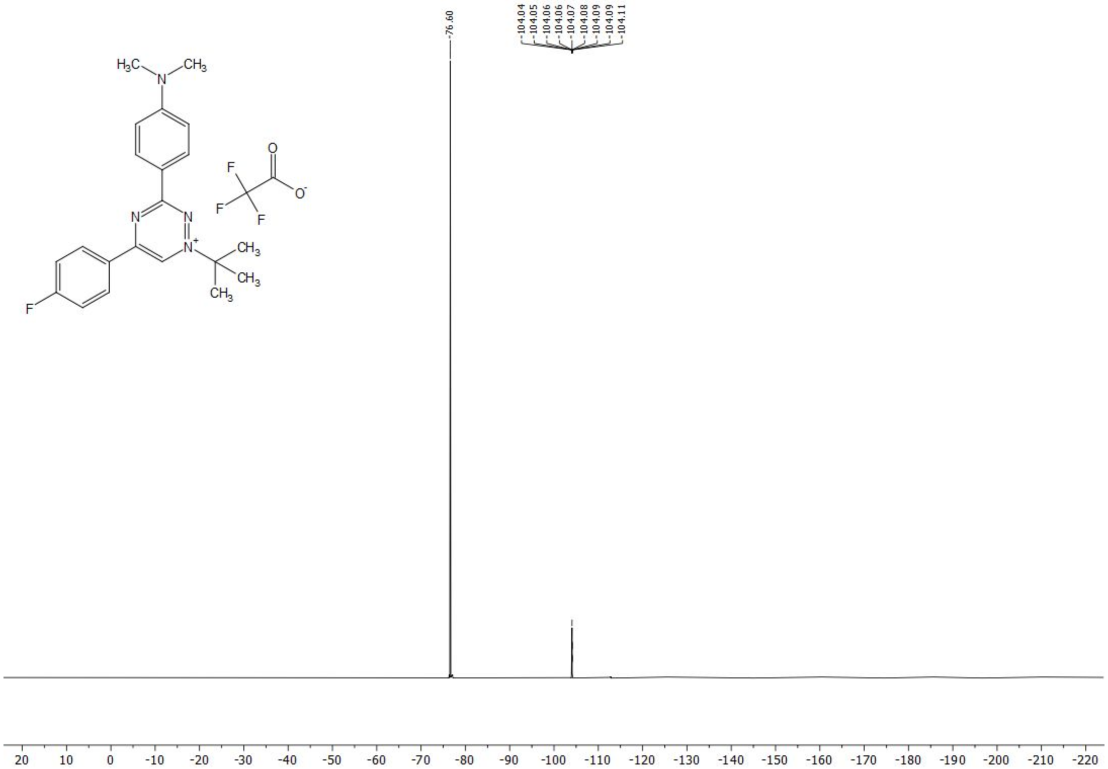

ArTrz⁺2p ^{13}C NMR (101 MHz, CD_3CN)

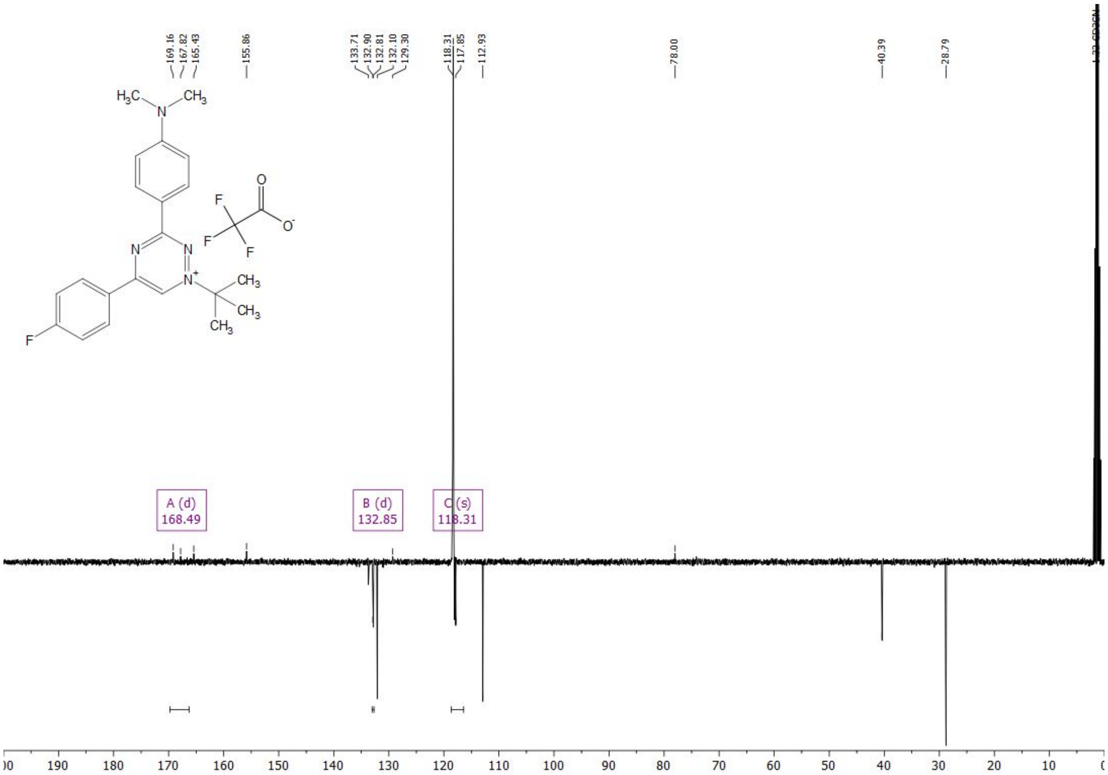

ArTrz⁺2q ^{1}H NMR (400 MHz, MeOH-d_4)

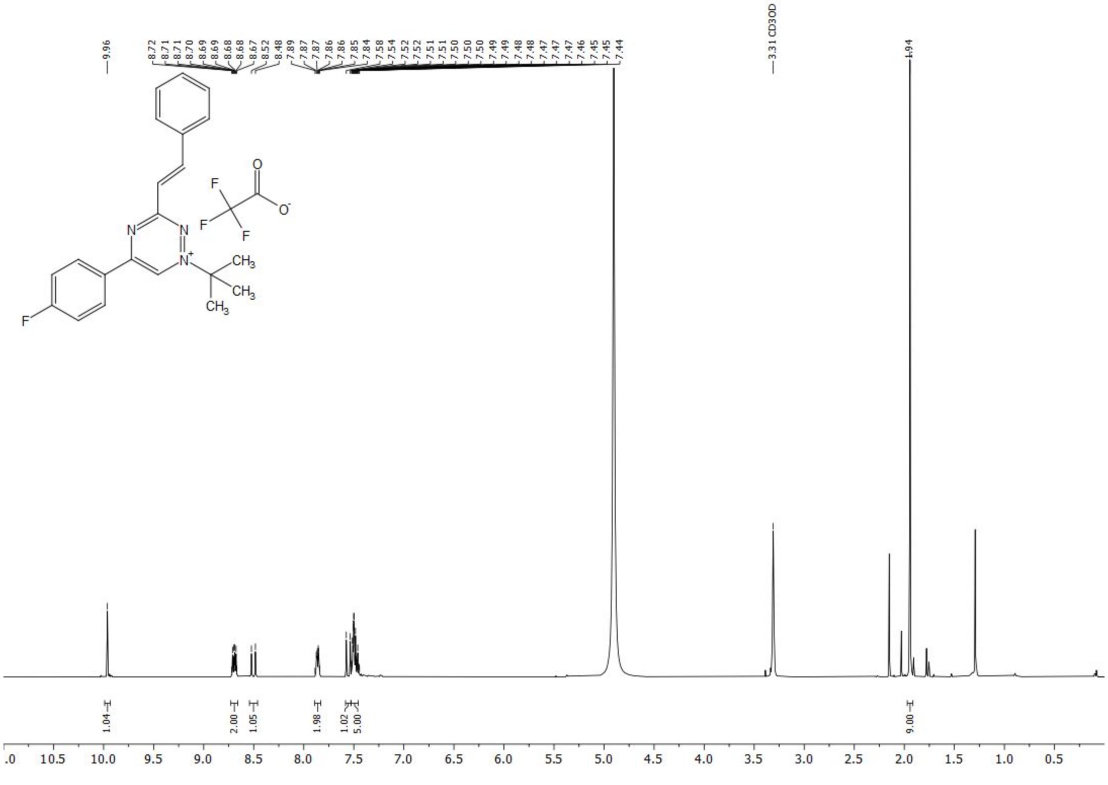

ArTrz+2q ^{19}F NMR (376 MHz, MeOH-d_4)

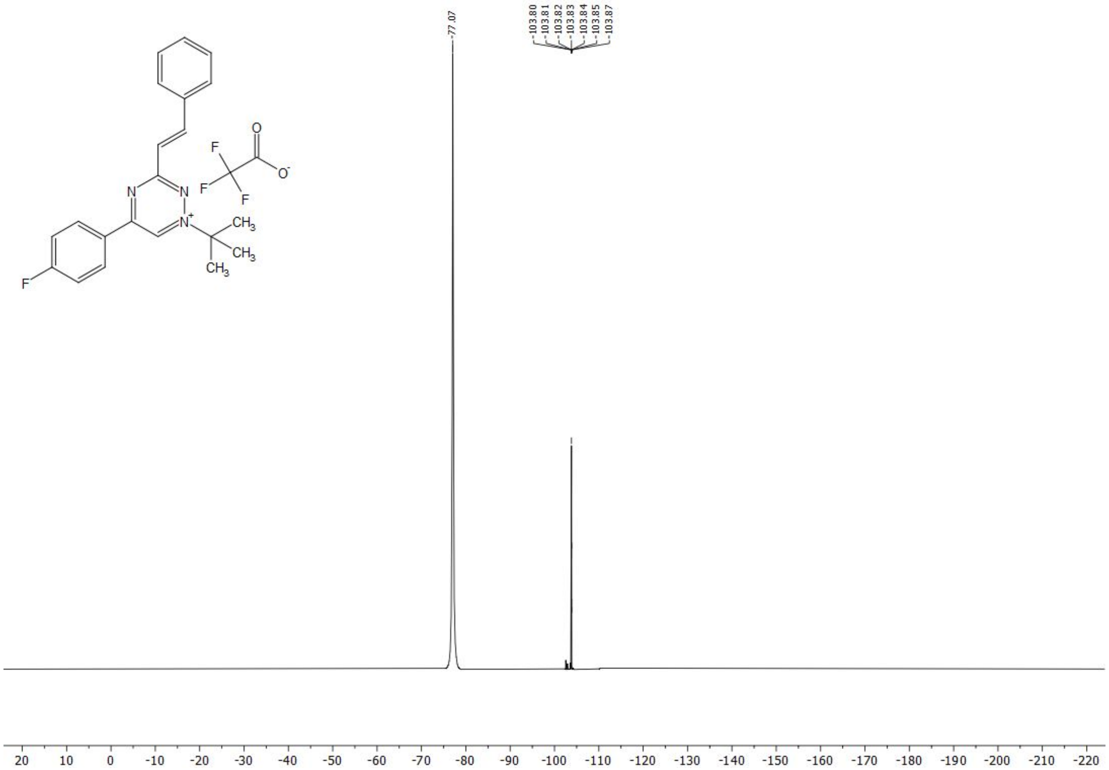

ArTrz+2q ^{13}C NMR (101 MHz, MeOH-d_4)

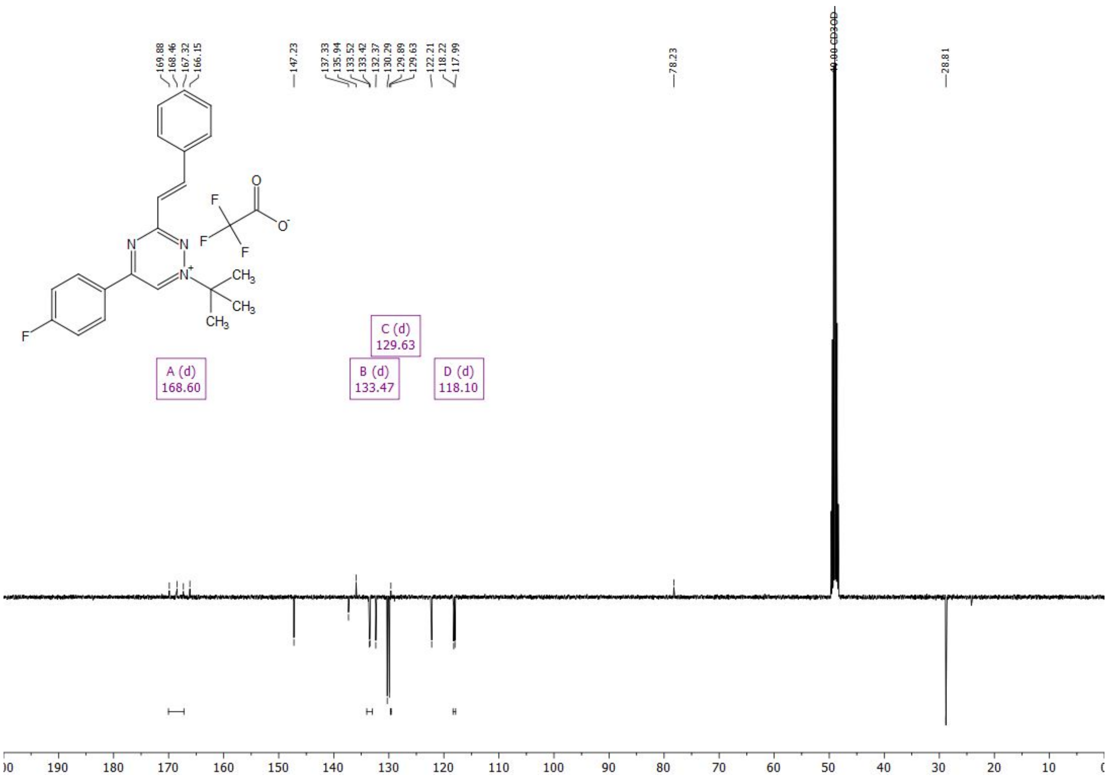

ArTrz⁺2r ^{1}H NMR (400 MHz, CD_3CN)

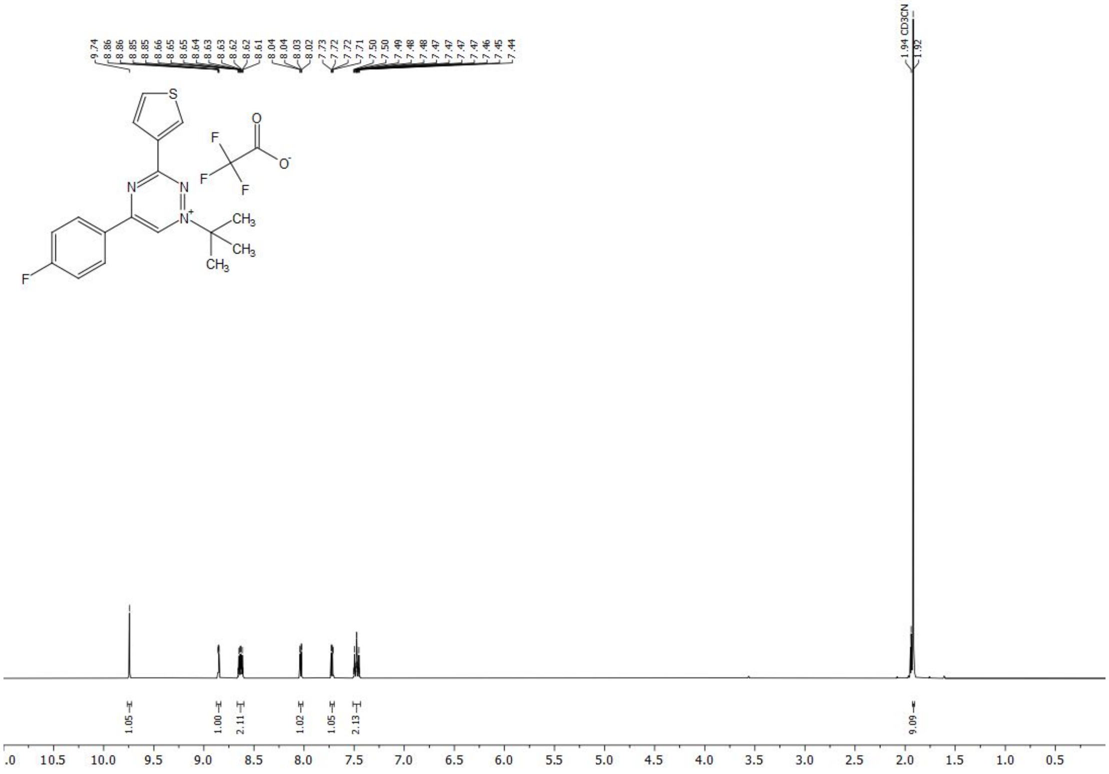

ArTrz⁺2r ^{19}F NMR (376 MHz, CD_3CN)

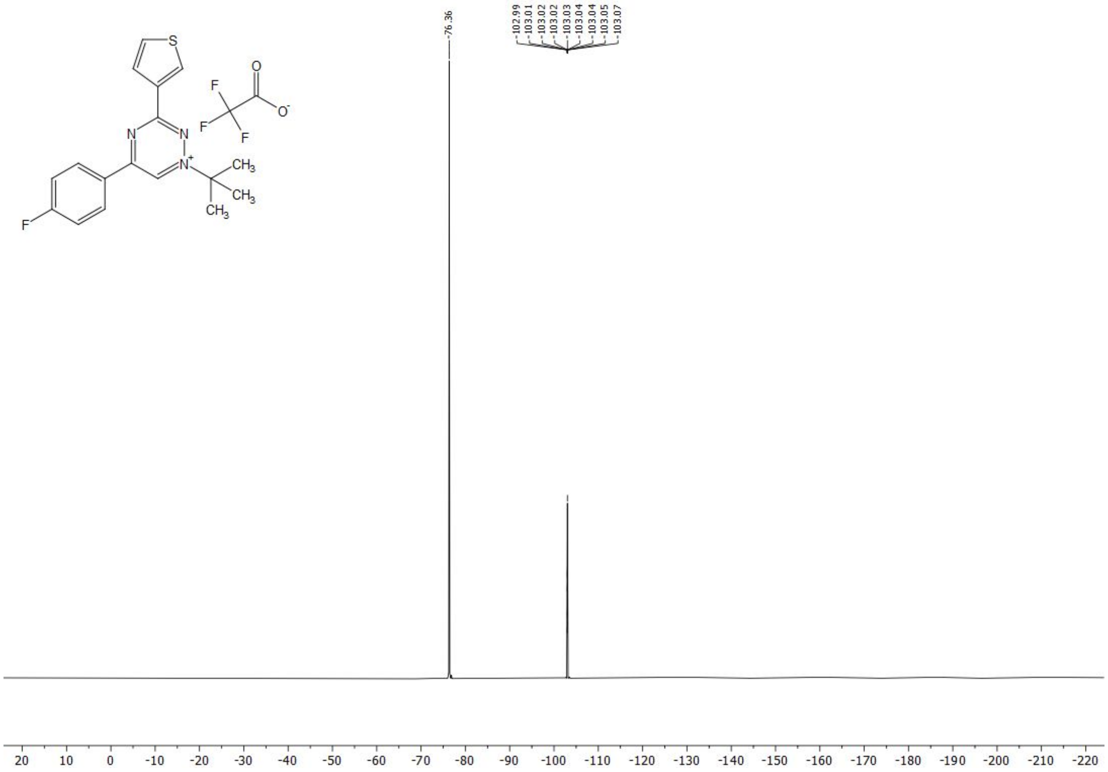

ArTrz⁺2r ^{13}C NMR (101 MHz, CD_3CN)

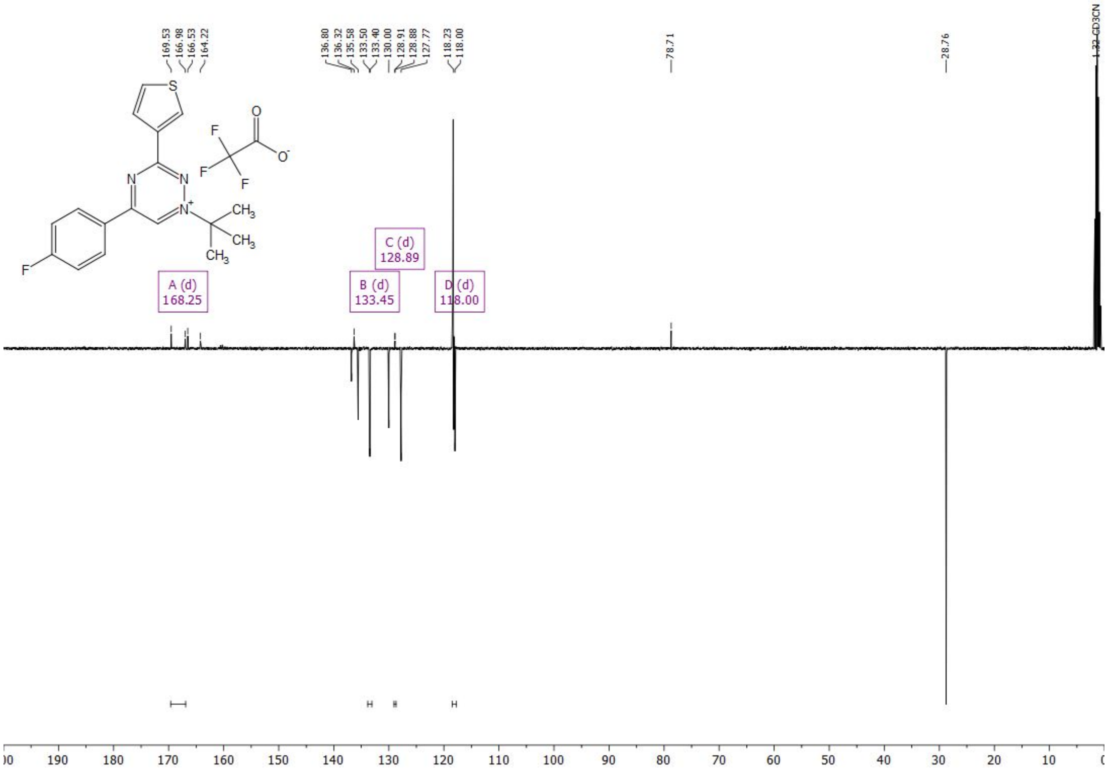

ArTrz⁺2s ^{1}H NMR (400 MHz, CD_3CN)

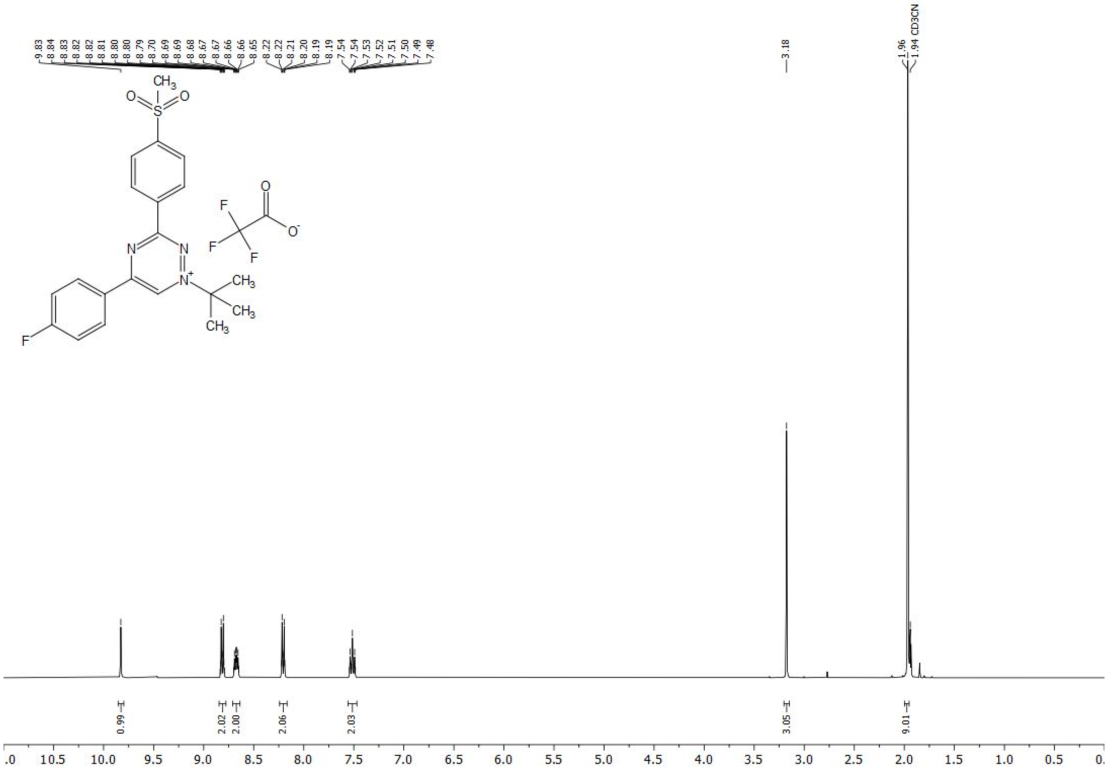

ArTrz⁺2s ^{19}F NMR (376 MHz, CD_3CN)

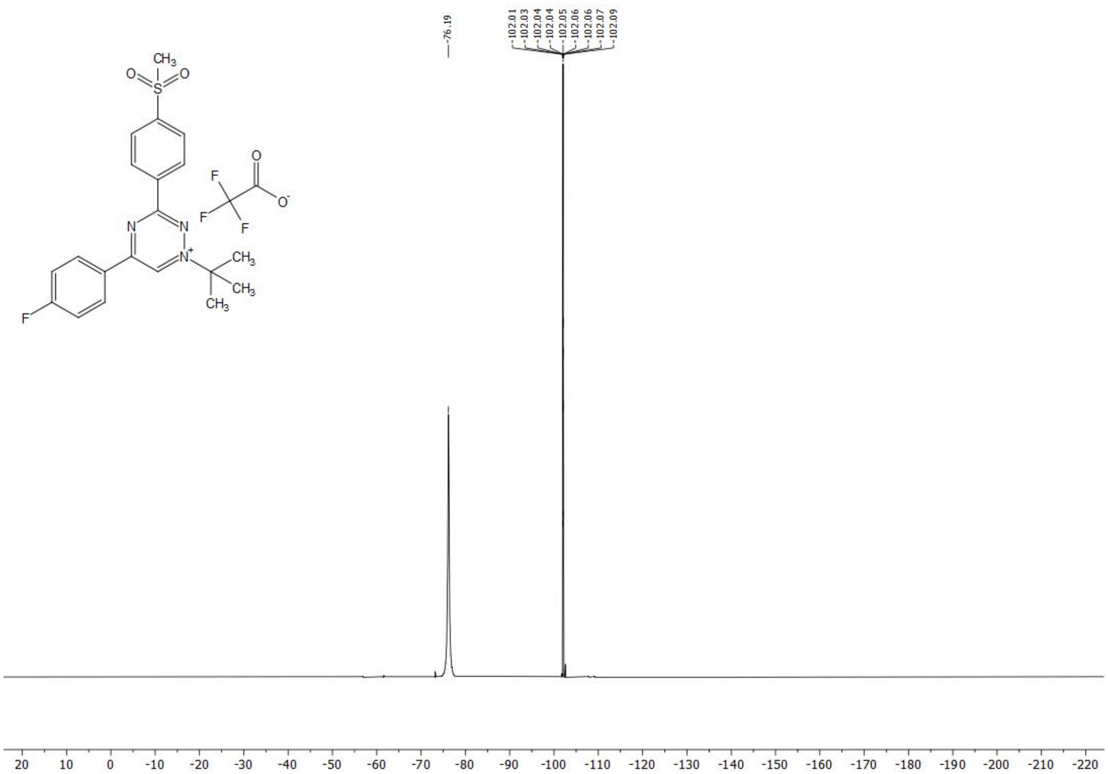

ArTrz⁺2s ^{13}C NMR (101 MHz, CD_3CN)

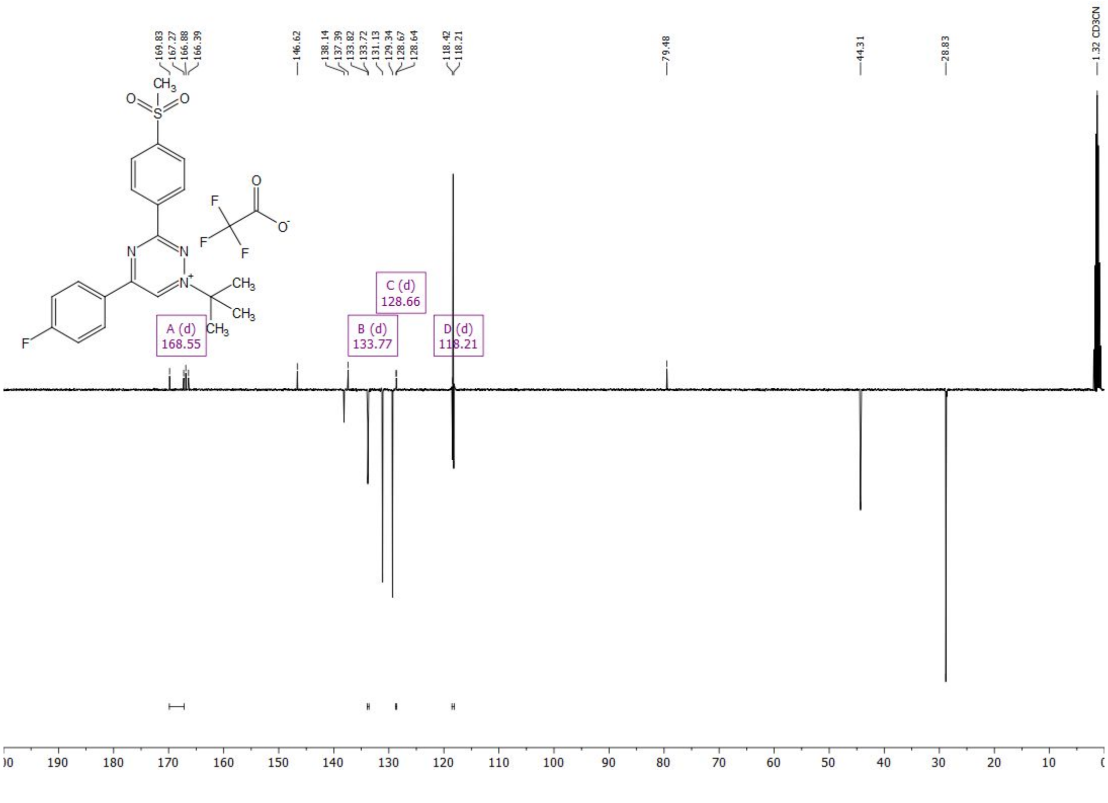

ArTrz⁺2t ¹H NMR (400 MHz, CD_3CN)

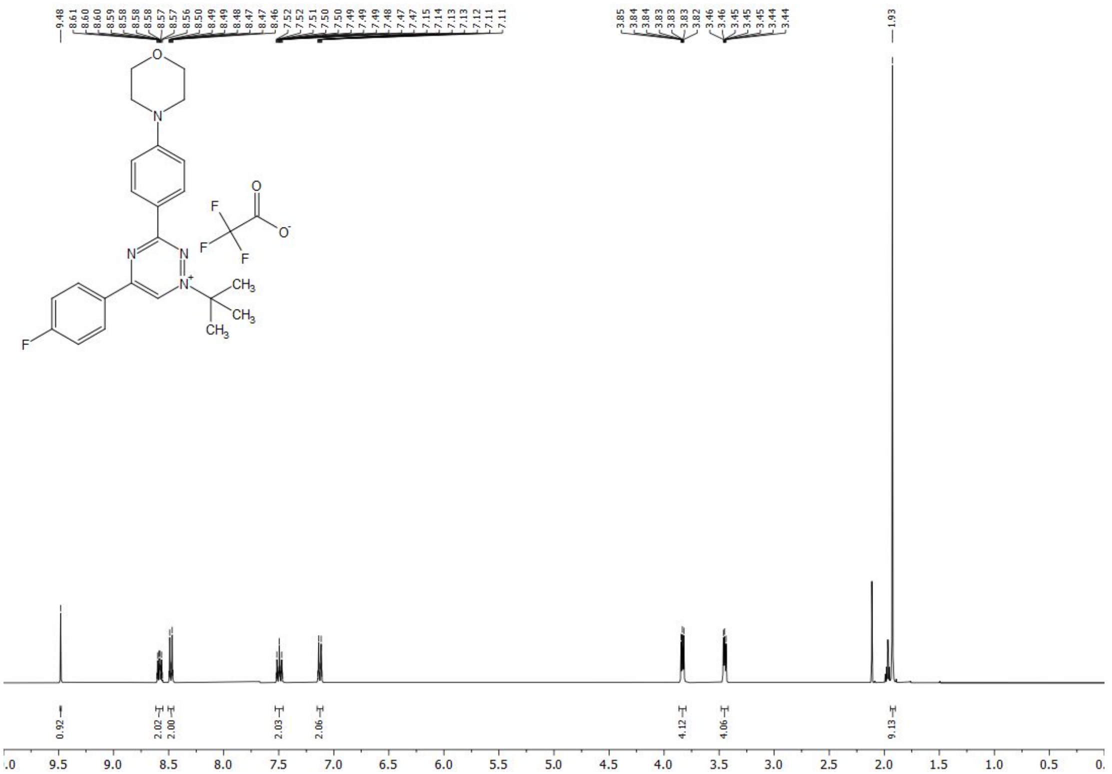

ArTrz⁺2t ¹⁹F NMR (376 MHz, CD_3CN)

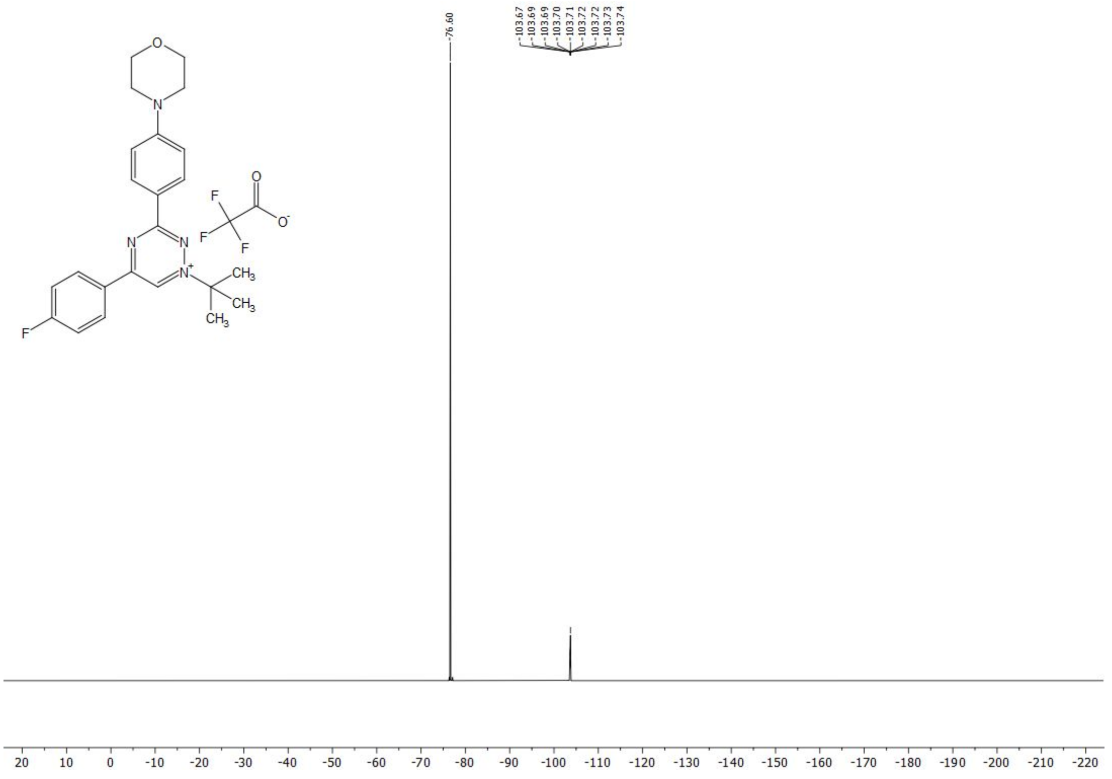

ArTrz⁺2t ^{13}C NMR (101 MHz, CD_3CN)

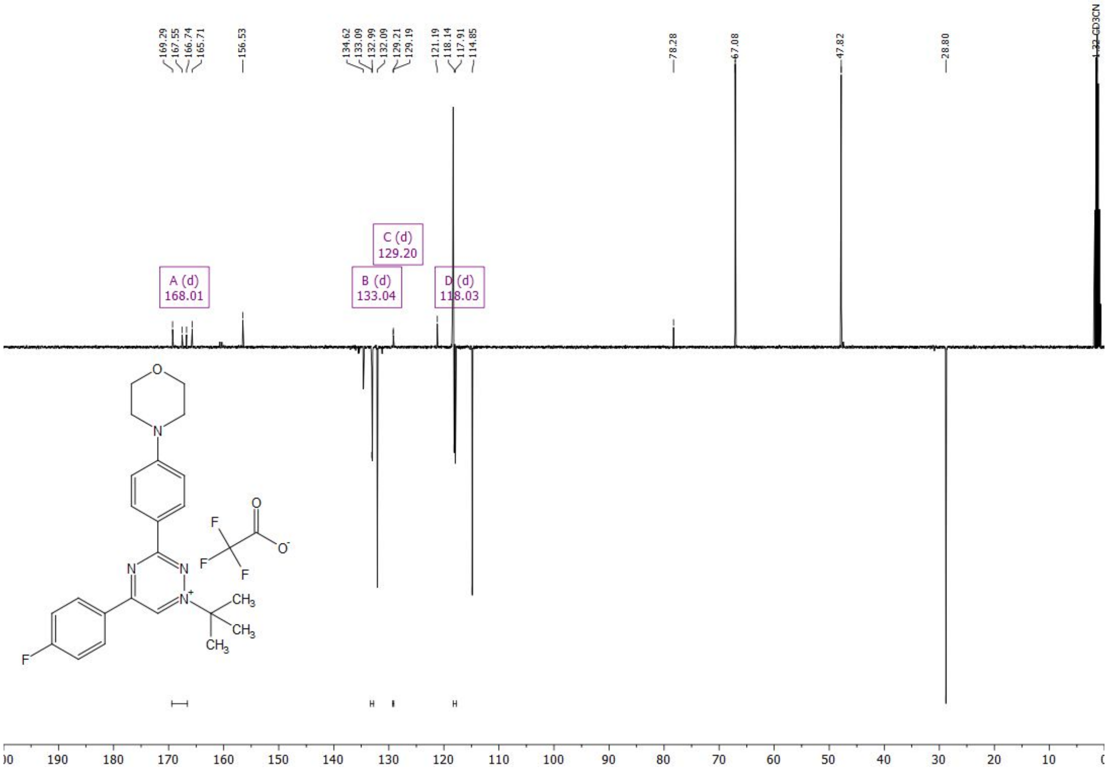

ArTrz⁺2u ^{1}H NMR (400 MHz, CD_3CN)

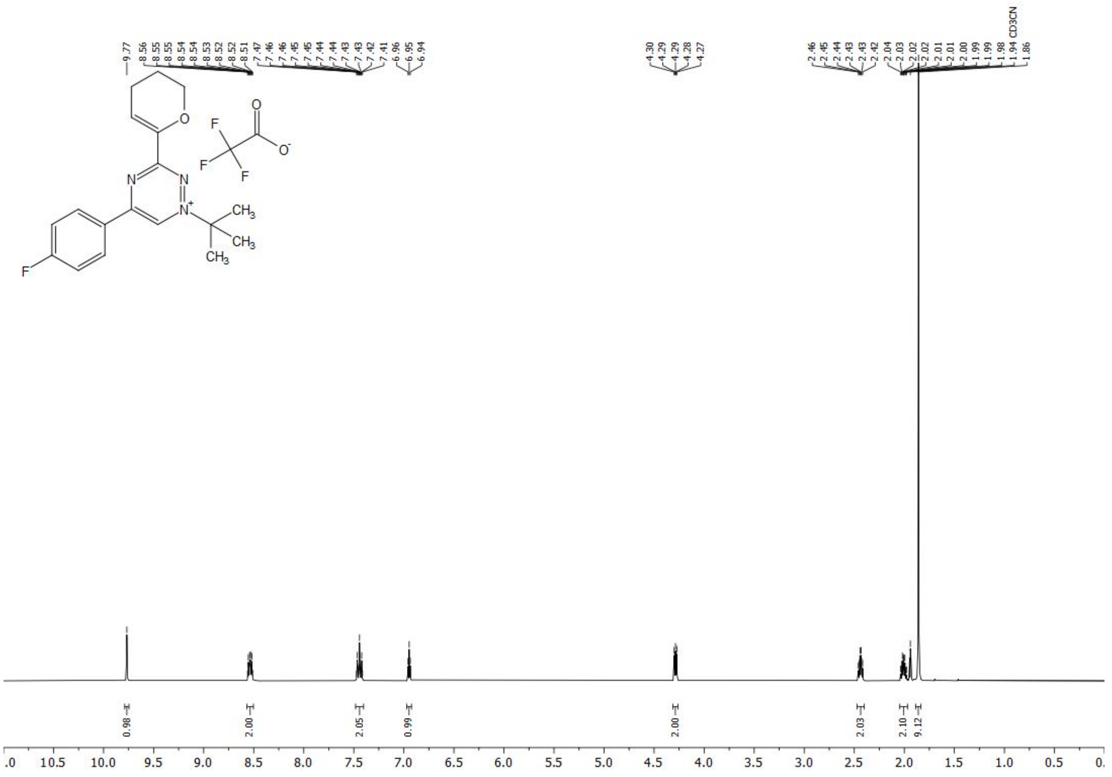

ArTrz⁺2u ^{19}F NMR (376 MHz, CD_3CN)

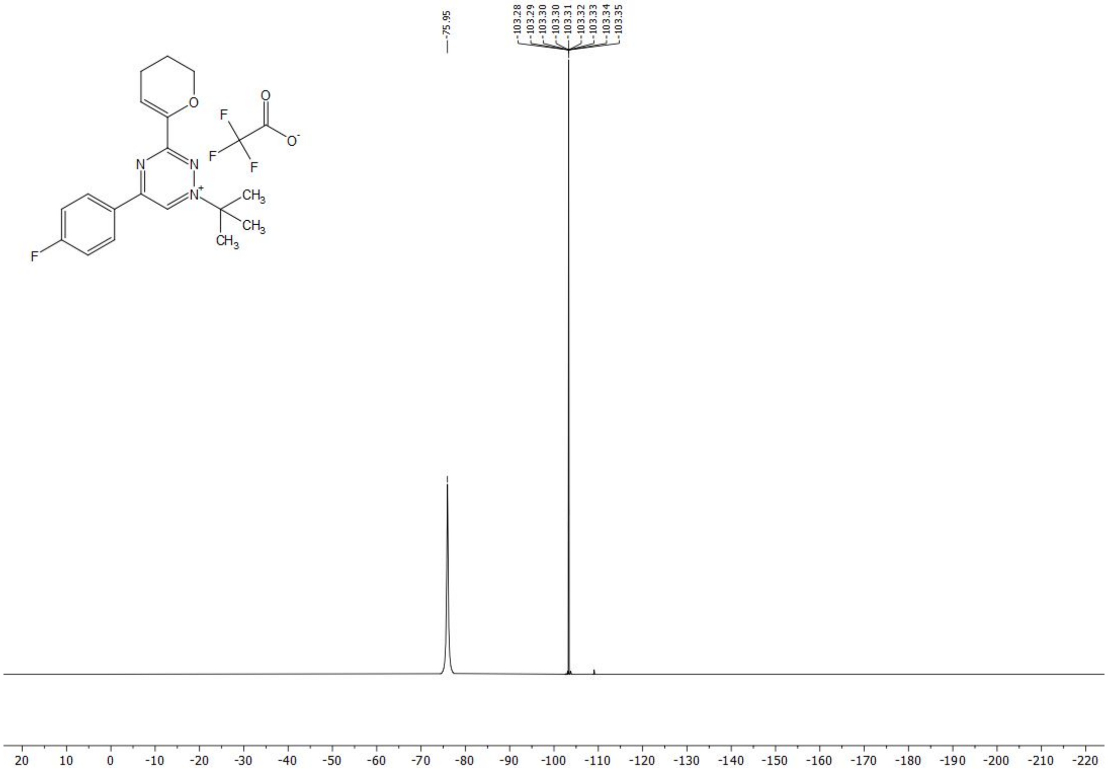

ArTrz⁺2u ^{13}C NMR (101 MHz, CD_3CN)

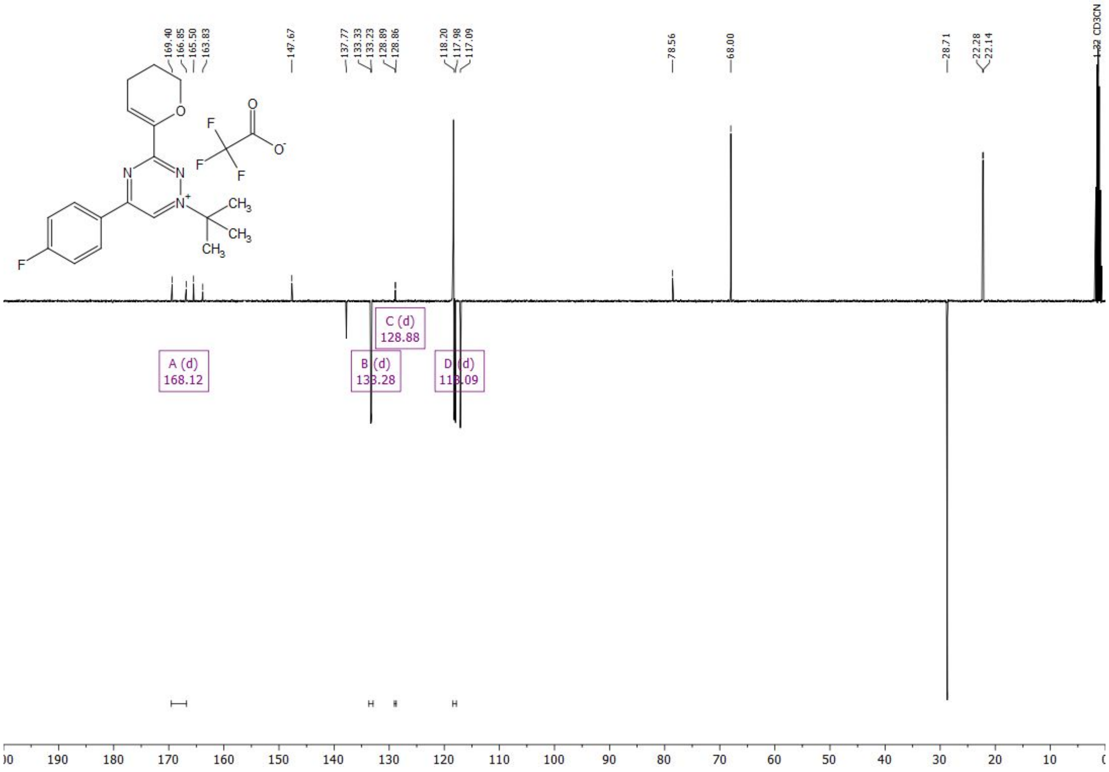

Red HTrz^{+}3 ^{1}H NMR (400 MHz, CD_3CN)

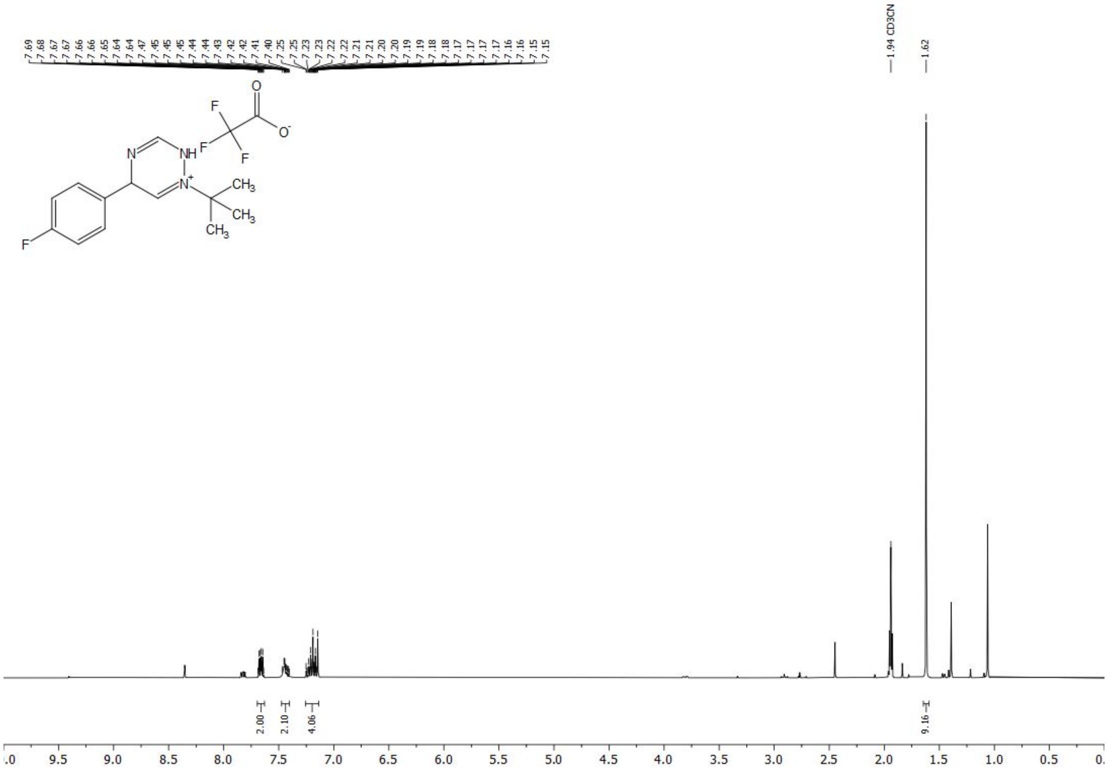

Red HTrz^{+}3^{19}F NMR (376 MHz, CD_3CN)

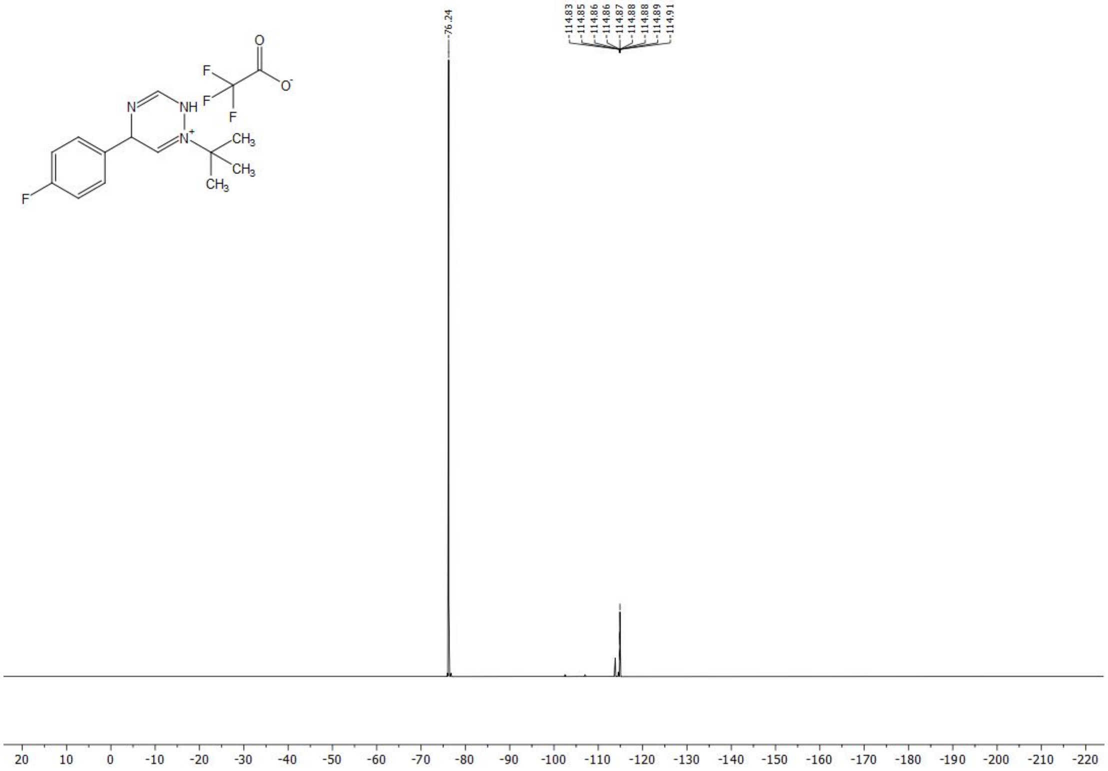

Arom HTrz⁺3 ^{1}H NMR (400 MHz, CD_3CN)

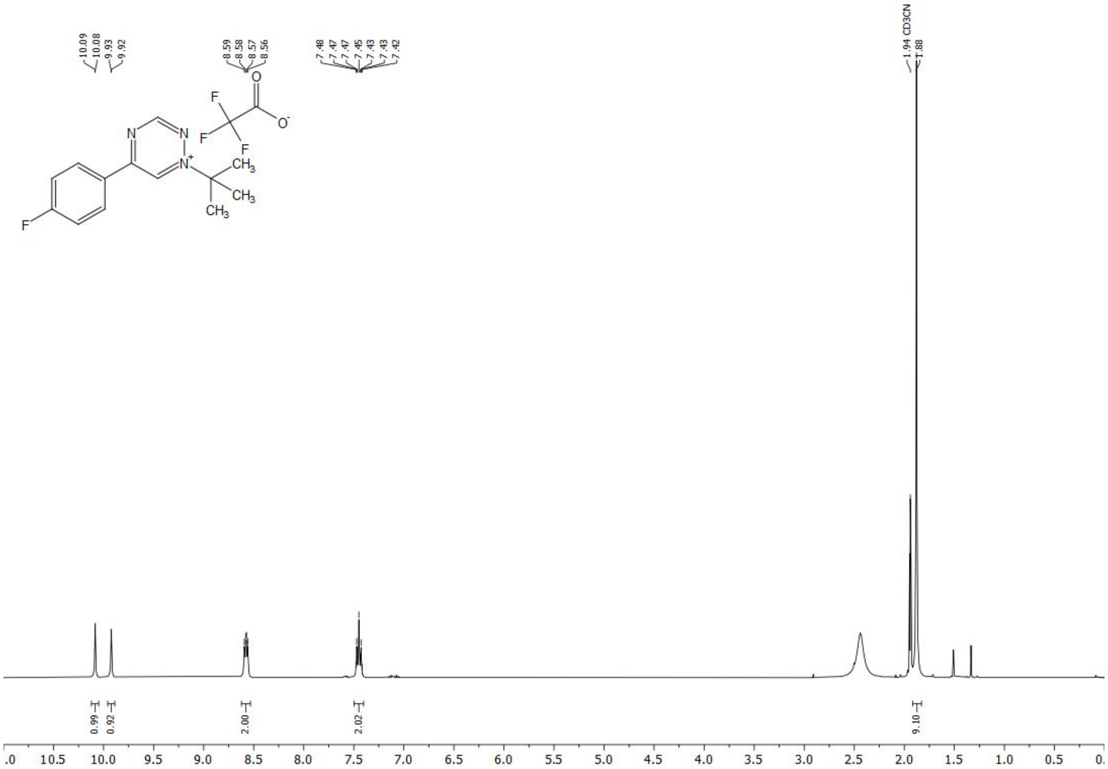

Arom HTrz⁺3 ^{19}F NMR (376 MHz, CD_3CN)

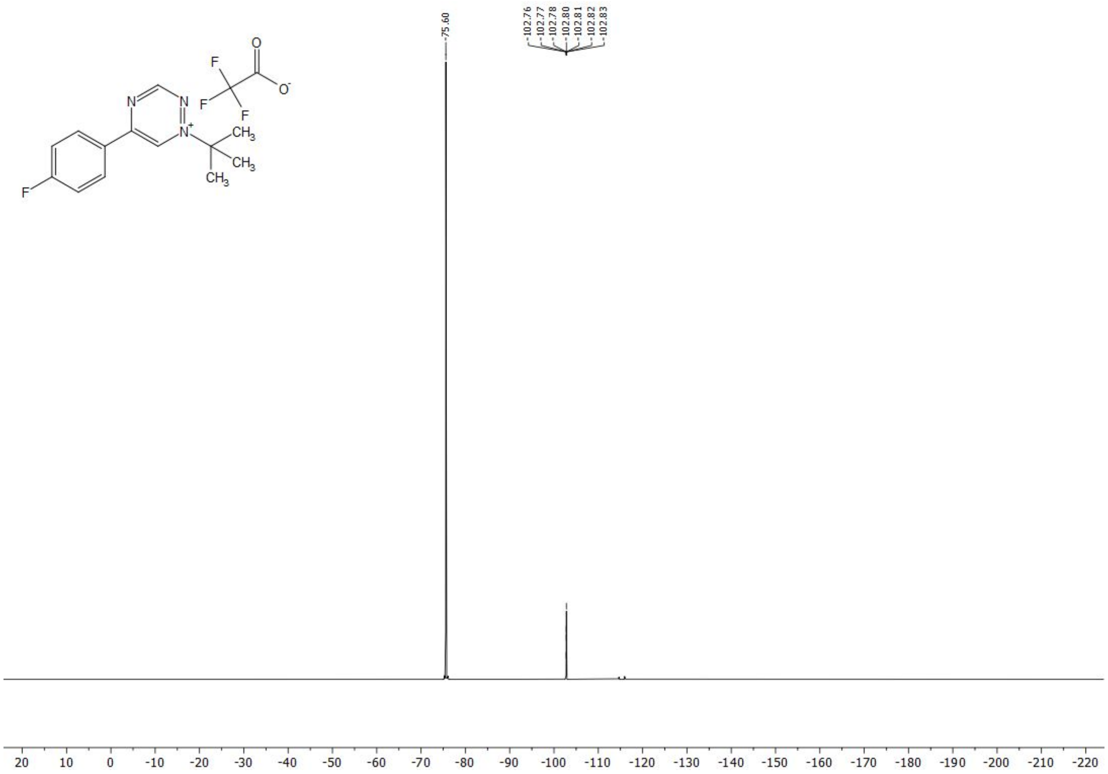

Arom HTrz⁺3 ^{13}C NMR (101 MHz, CD_3CN)

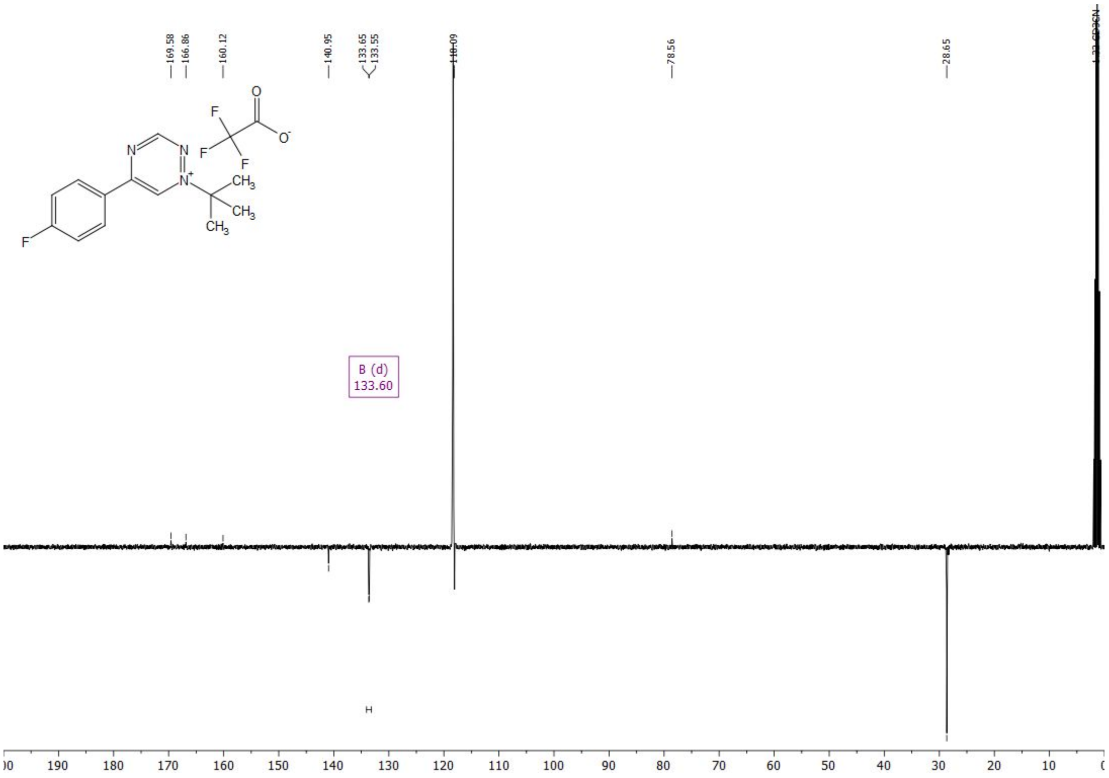

SMeTrz⁺5 ^{1}H NMR (400 MHz, CD_3CN)

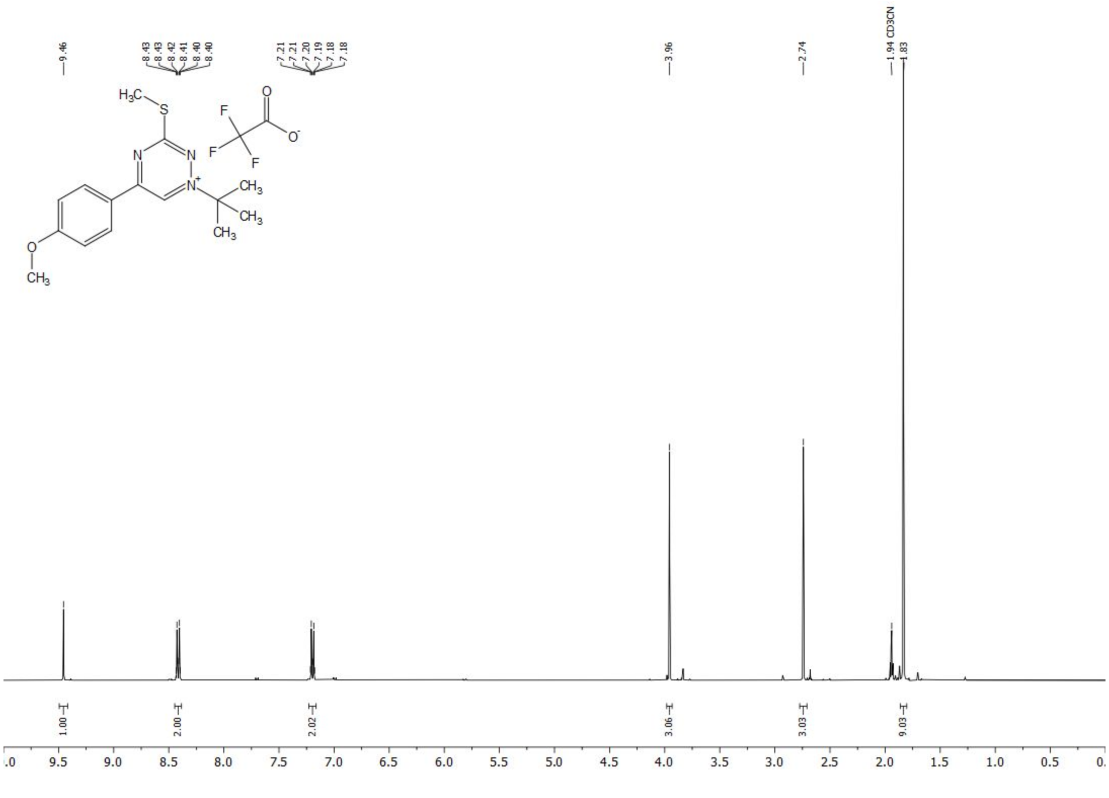

SMeTrz⁺5 ^{19}F NMR (376 MHz, CD_3CN)

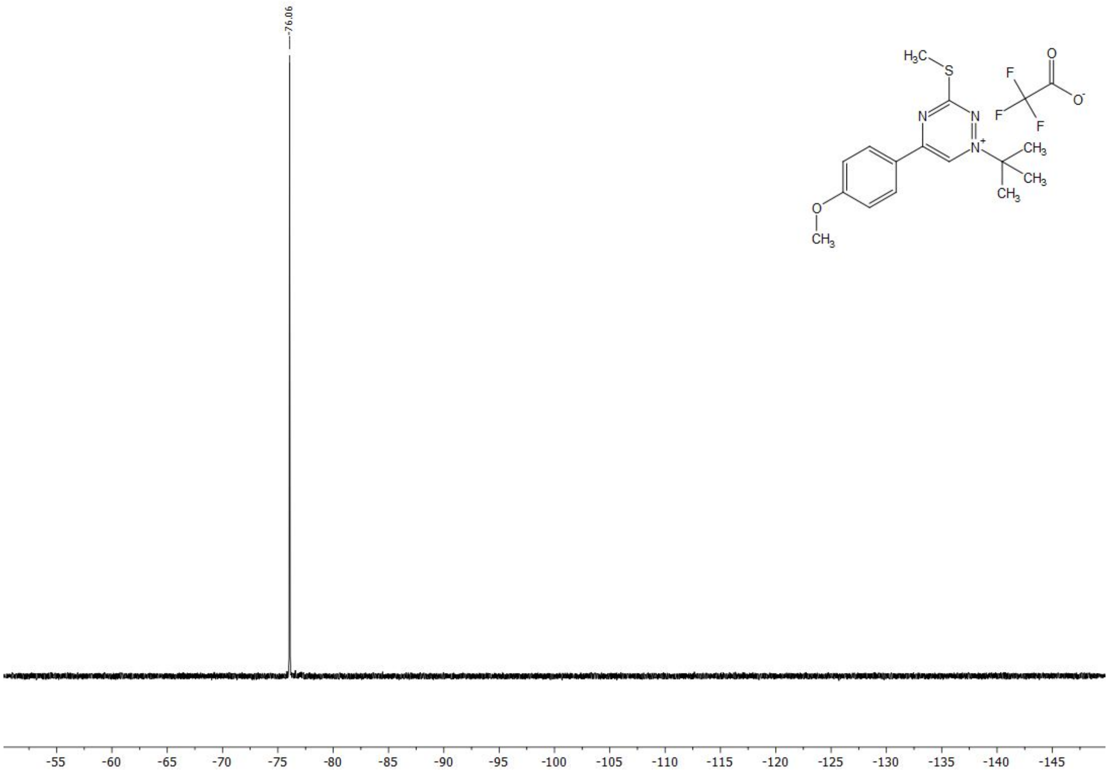

SMeTrz⁺5 ^{13}C NMR (101 MHz, CD_3CN)

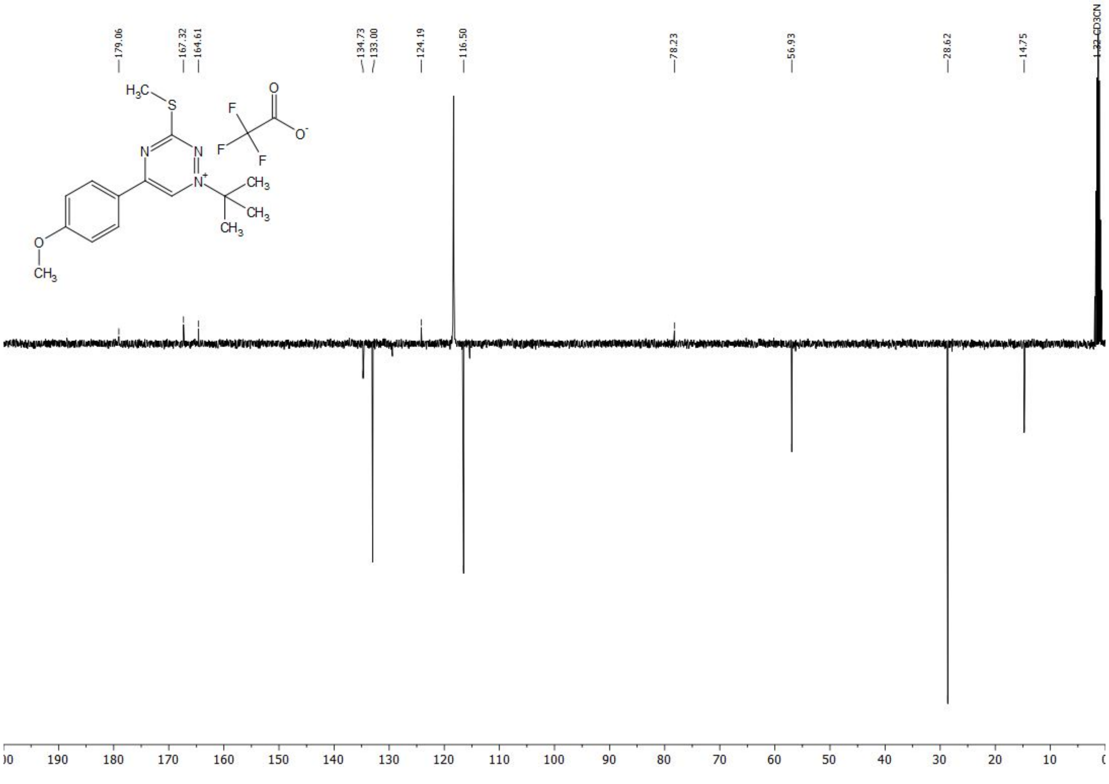

ArTrz ^{1}H NMR (400 MHz, CDCl$_3$)

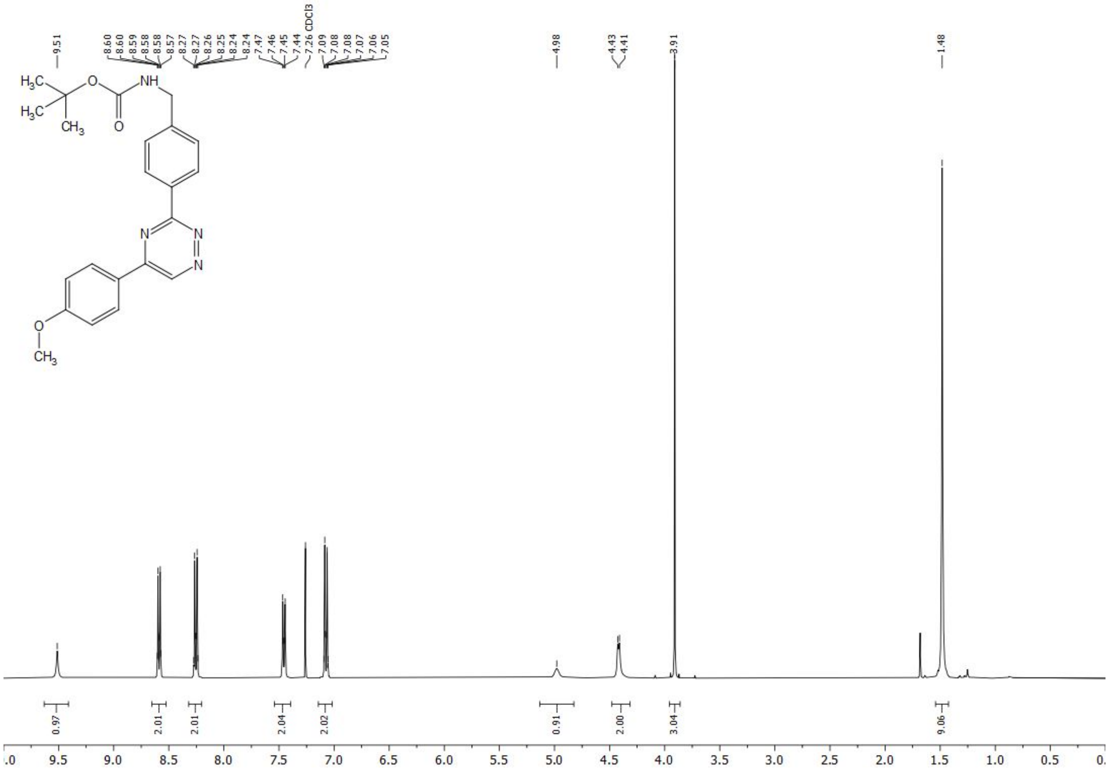

ArTrz ^{13}C NMR (101 MHz, CDCl$_3$)

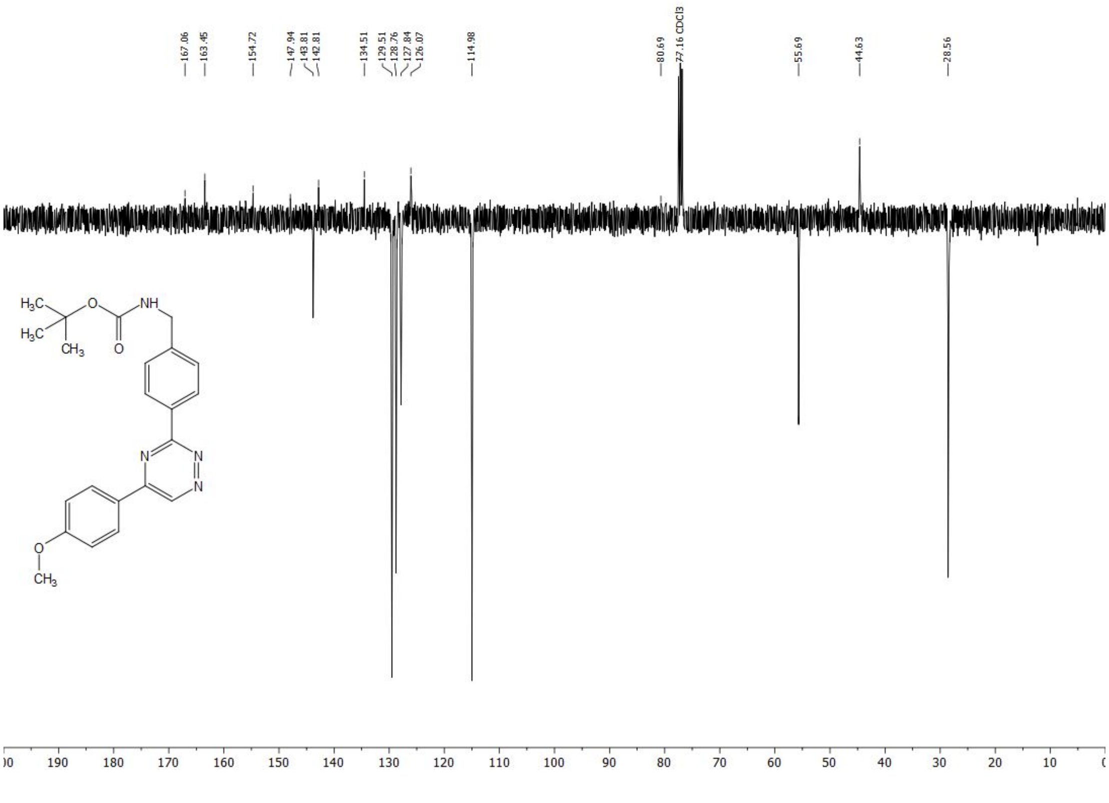

ArTrz⁺4 ^{1}H NMR (400 MHz, CD_3CN/D_2O)

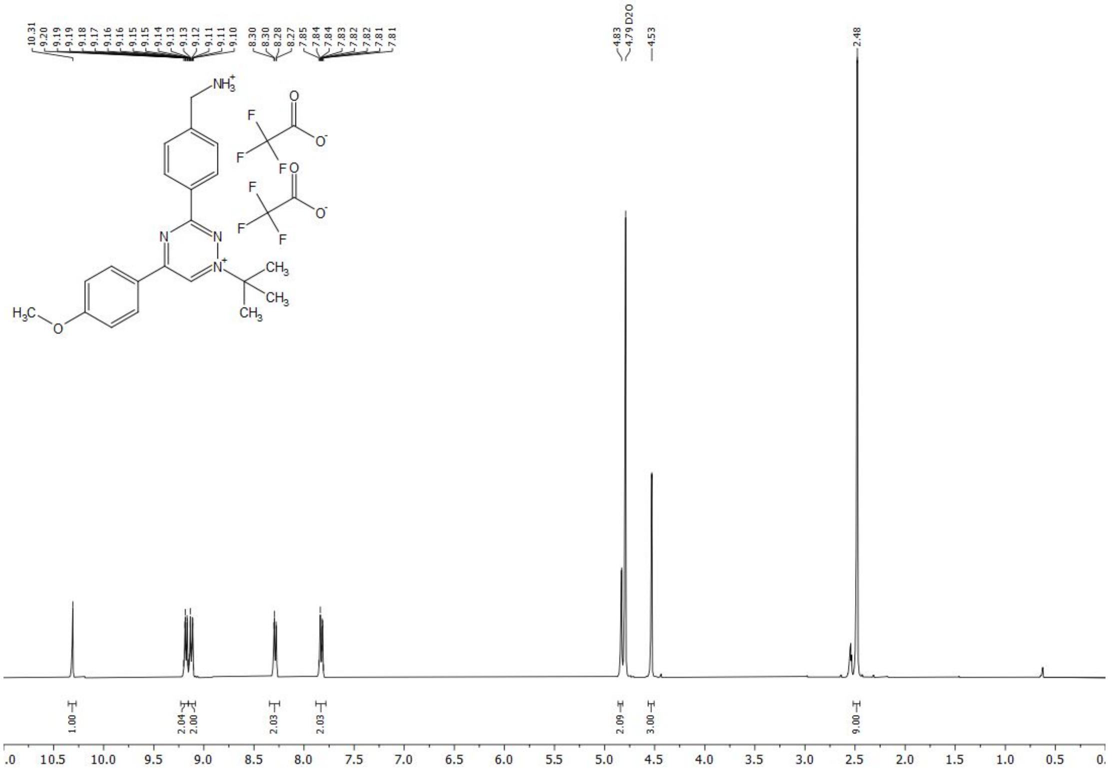

ArTrz⁺4 ^{19}F NMR (376 MHz, CD_3CN/D_2O)

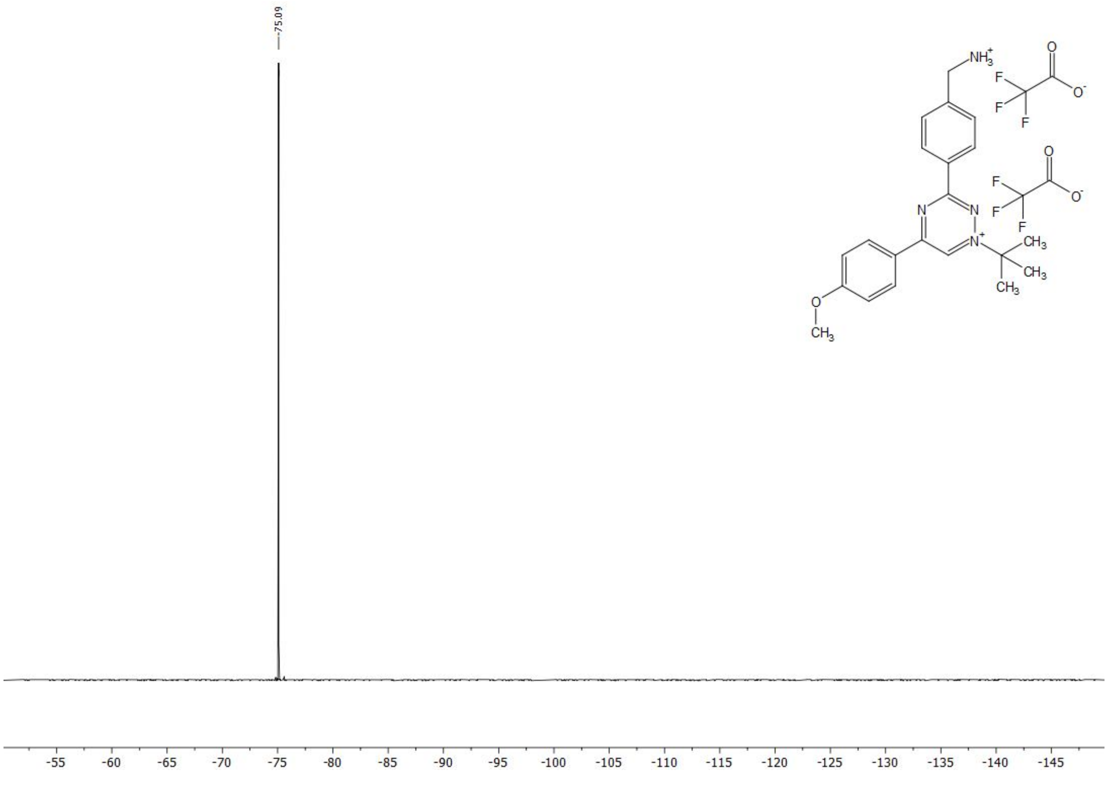

ArTrz⁺4 ^{13}C NMR (101 MHz, CD_3CN/D_2O)

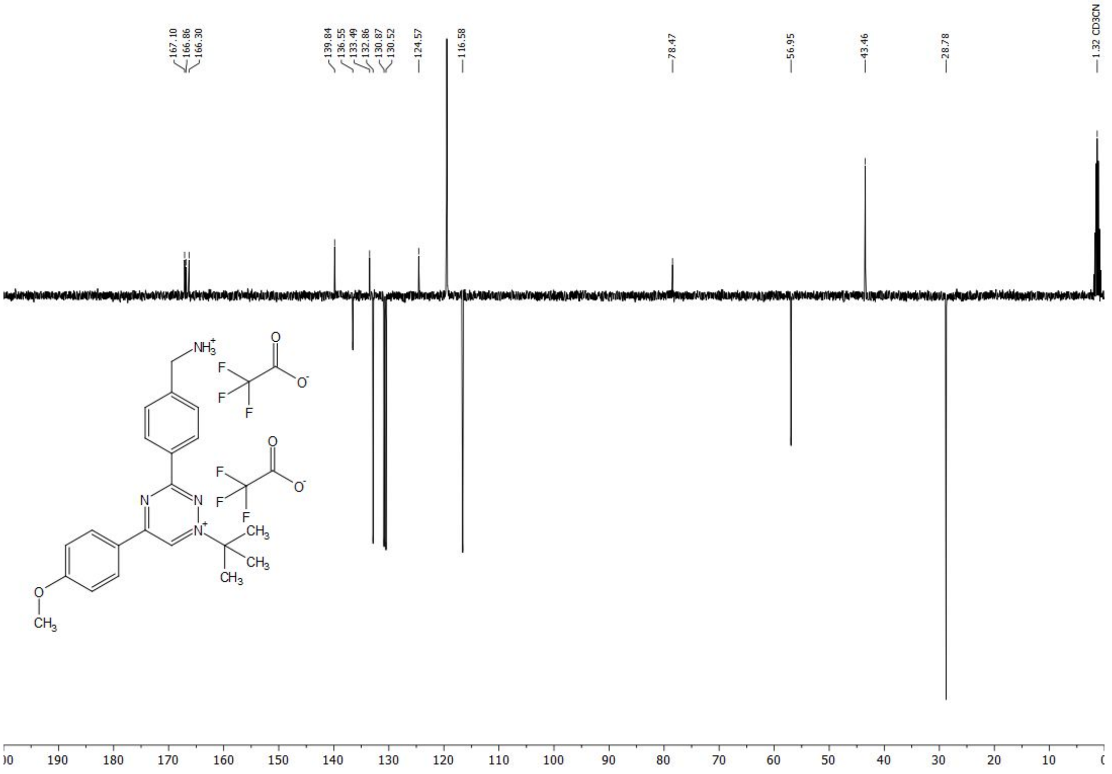

Coum-BA1 ^{1}H NMR (500 MHz, DMSO-d_6)

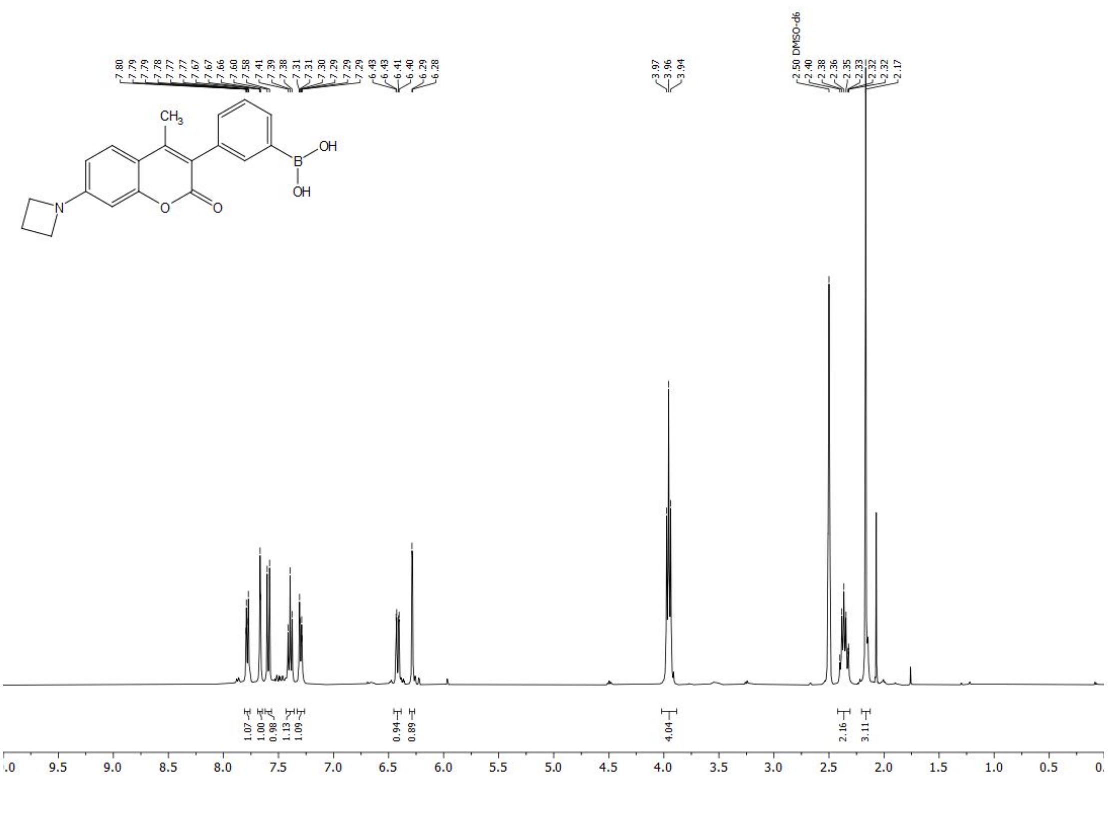

Coum-BA1 ^{13}C NMR (126 MHz, DMSO-d_6)

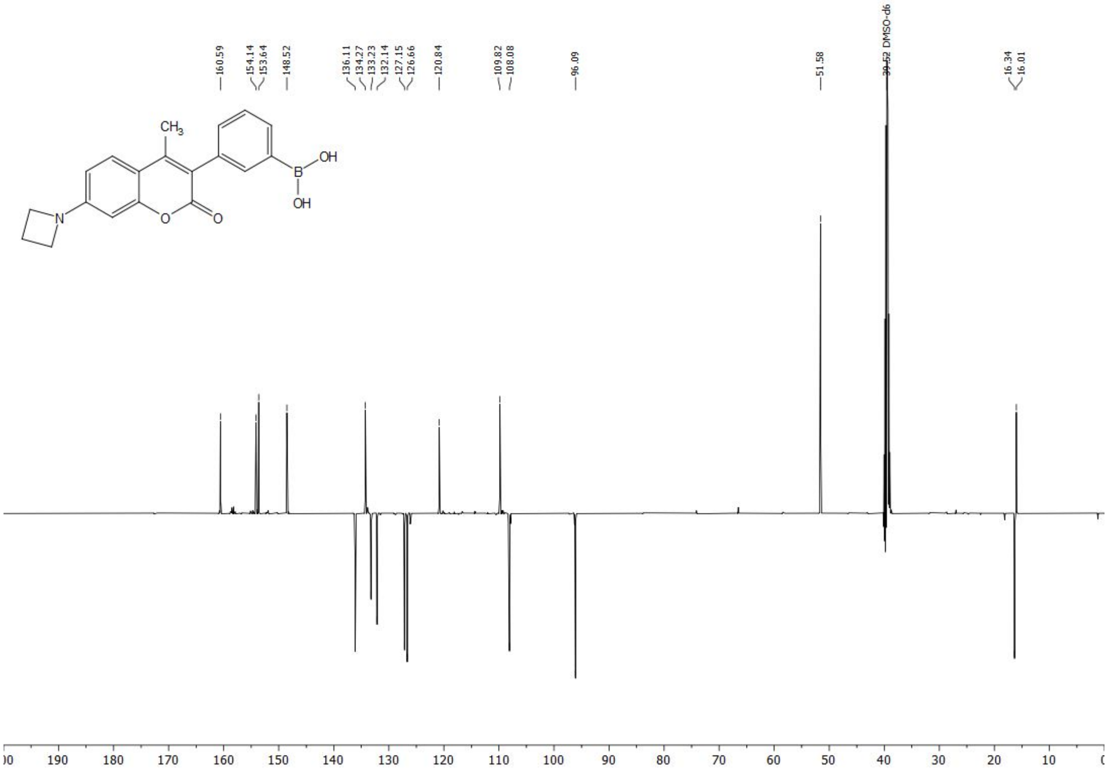

Coum-BA2 ^{1}H NMR (500 MHz, DMSO-d_6)

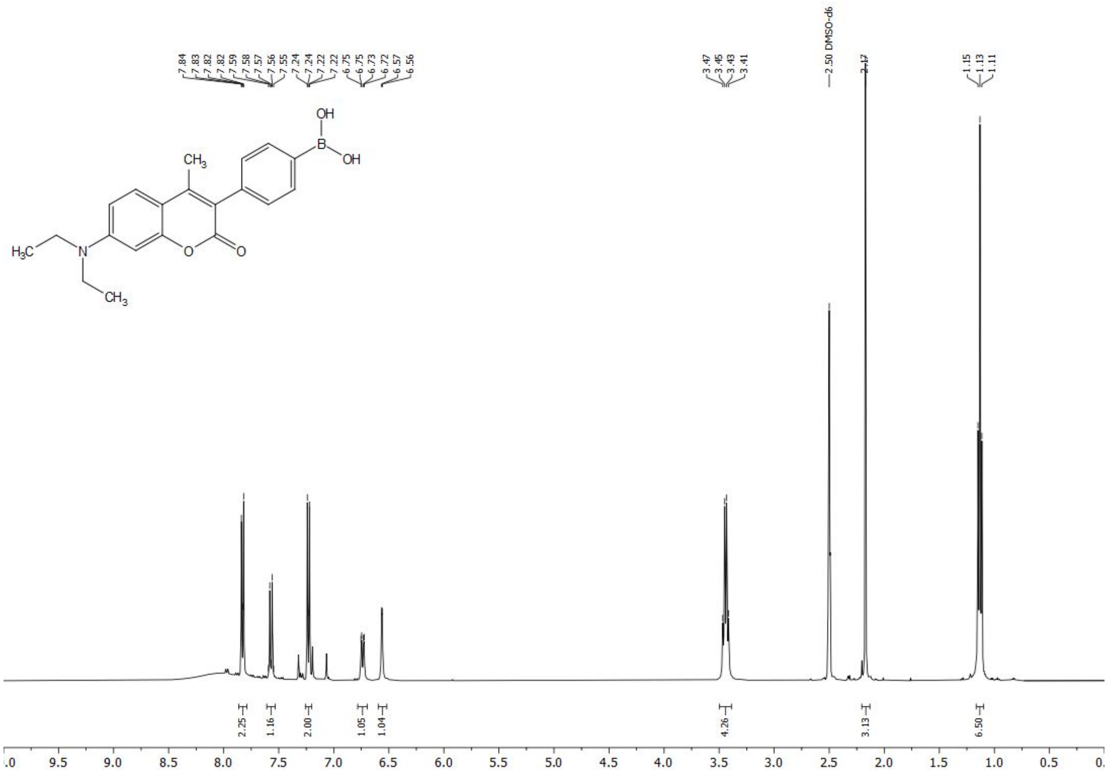

Coum-BA2 ^{13}C NMR (126 MHz, DMSO-d_6)

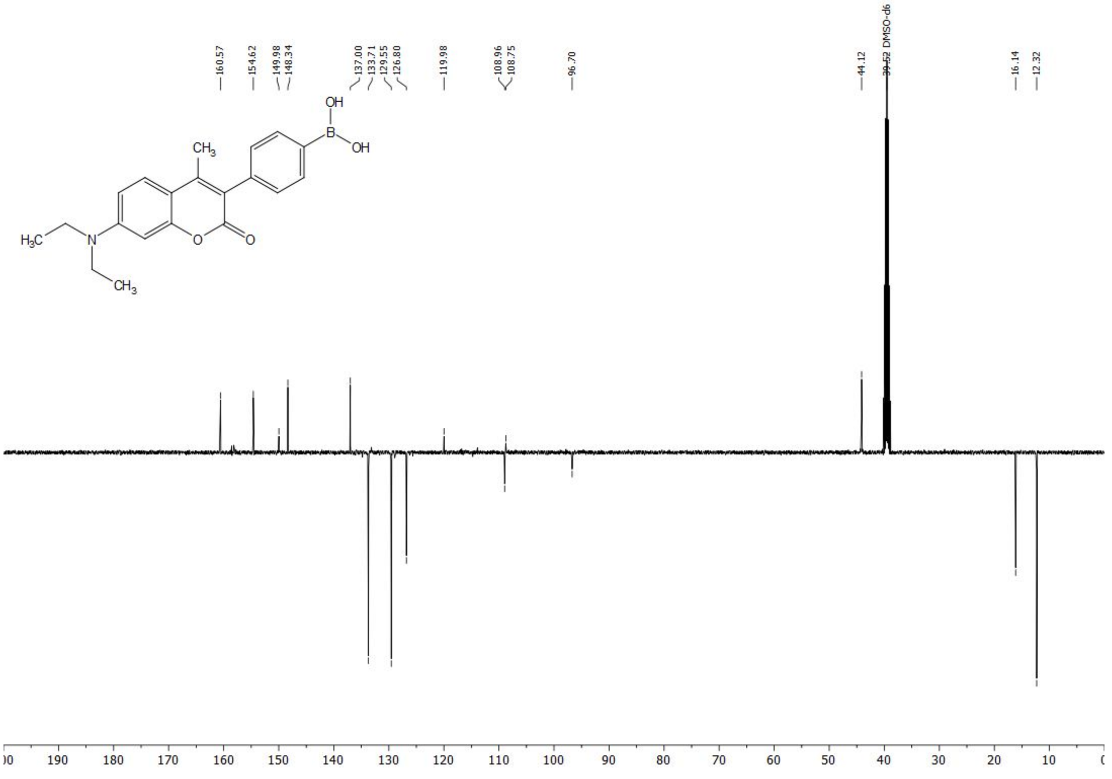

Trz^{+}Coum6 ^{1}H NMR (400 MHz, CD_3CN)

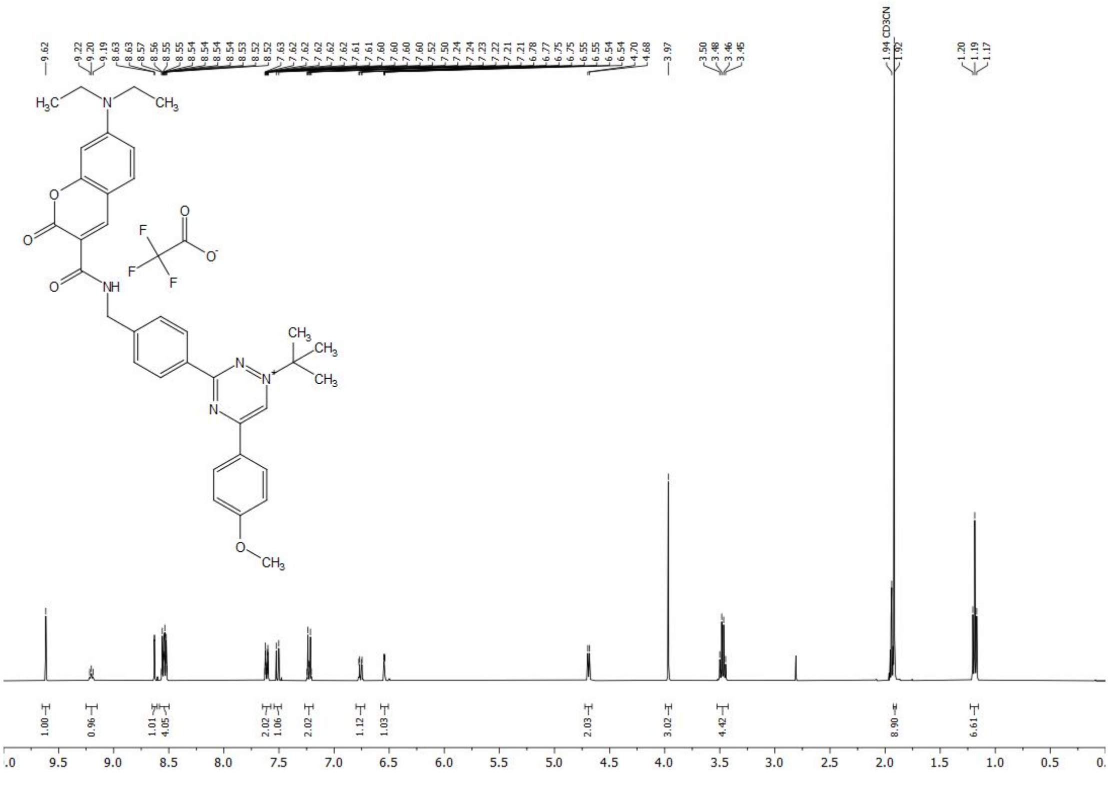

Trz+Coum6 ^{19}F NMR (376 MHz, CD_3CN)

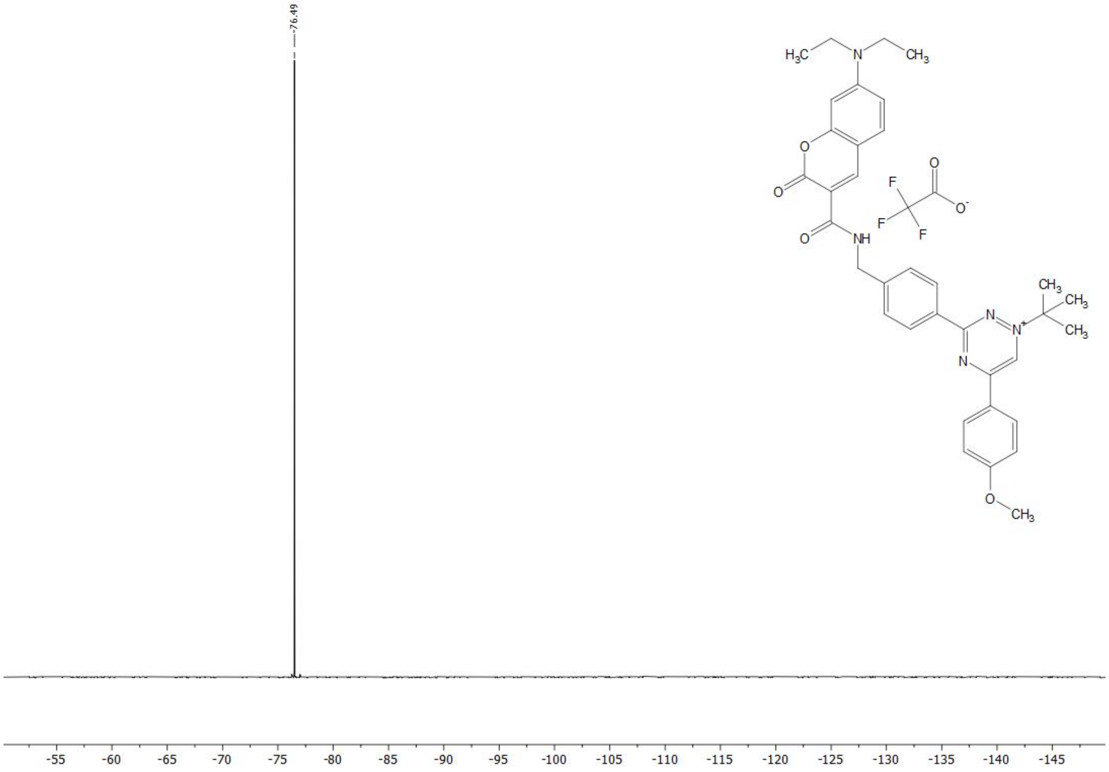

Trz+Coum6 ^{13}C NMR (101 MHz, CD_3CN)

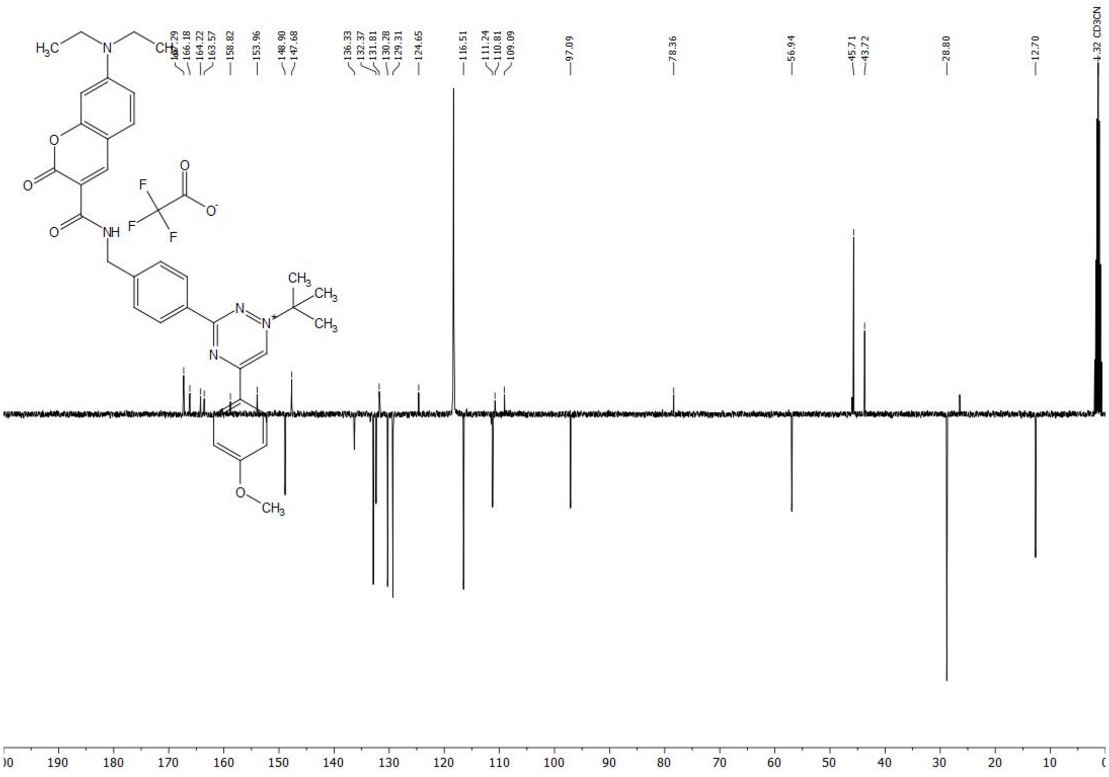

Trz+Coum7 ^{1}H NMR (400 MHz, CD_3CN)

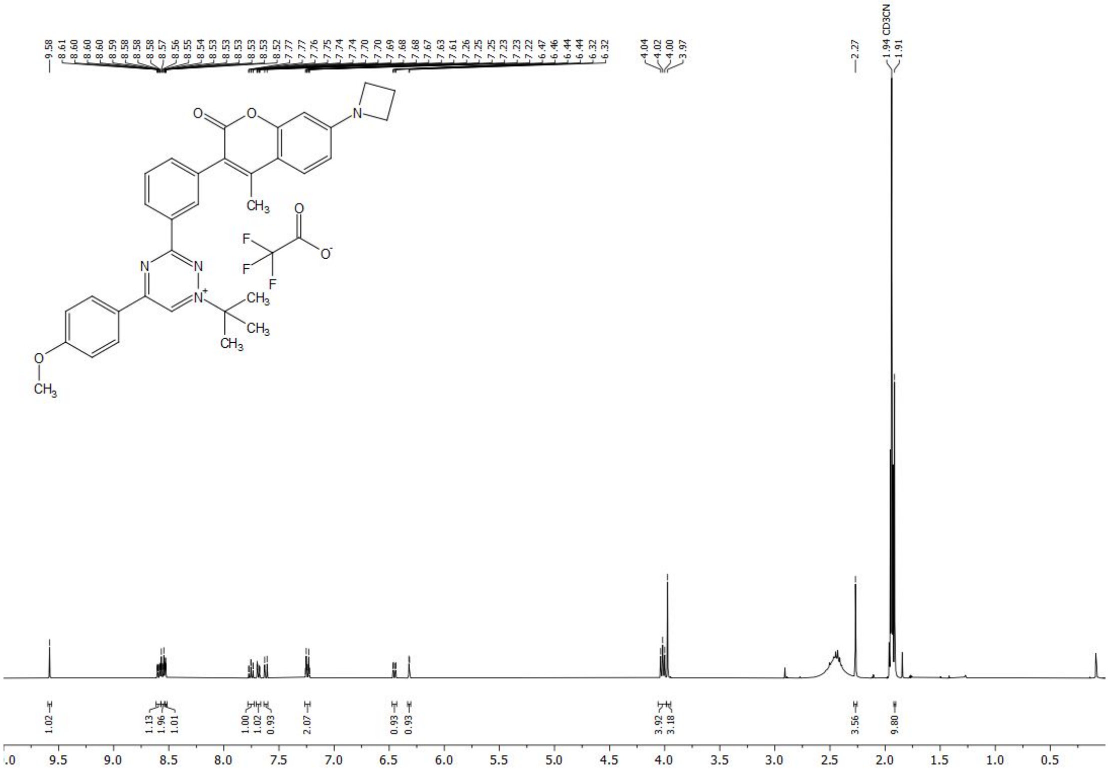

Trz+Coum7 ^{19}F NMR (376 MHz, CD_3CN)

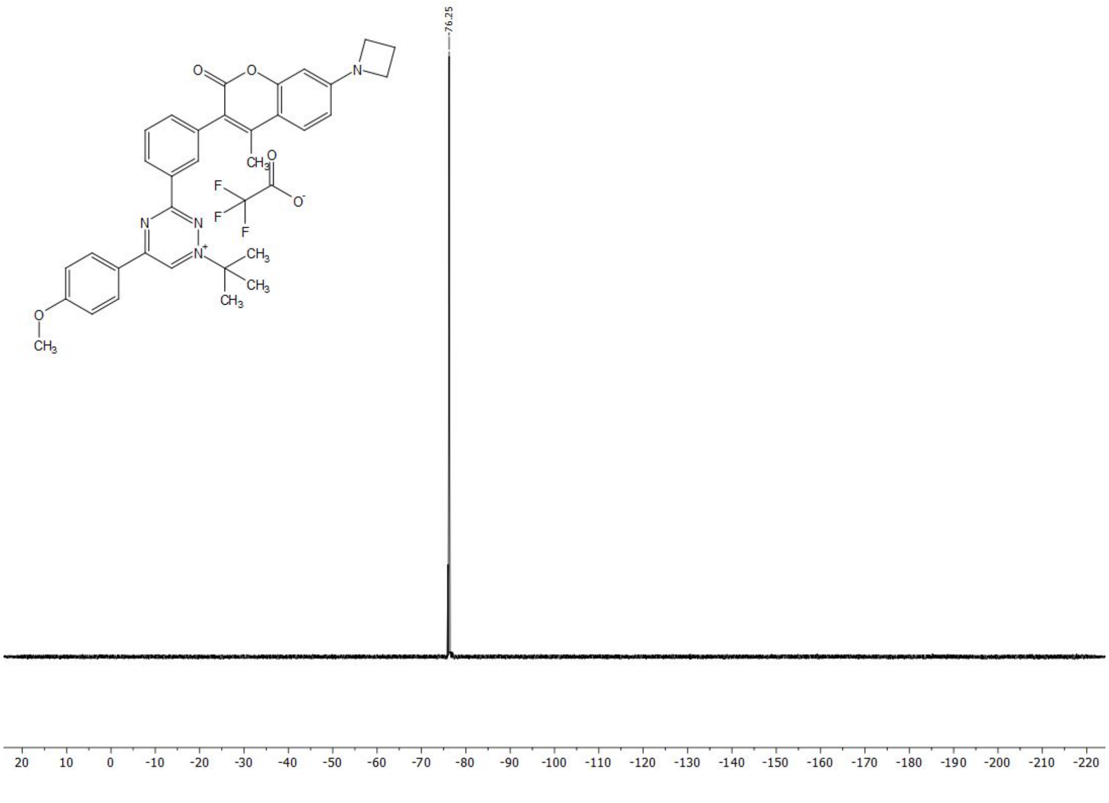

Trz⁺Coum7 ^{13}C NMR (101 MHz, CD_3CN)

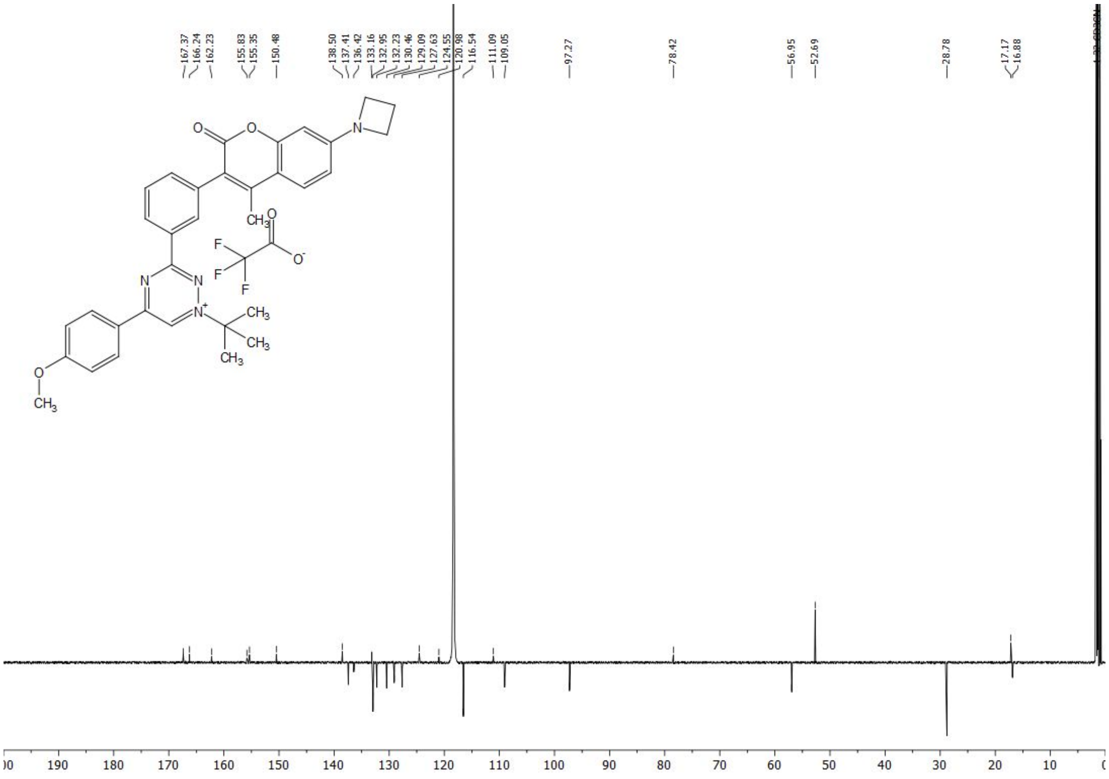

Trz⁺Coum8 ^{1}H NMR (400 MHz, CD_3CN)

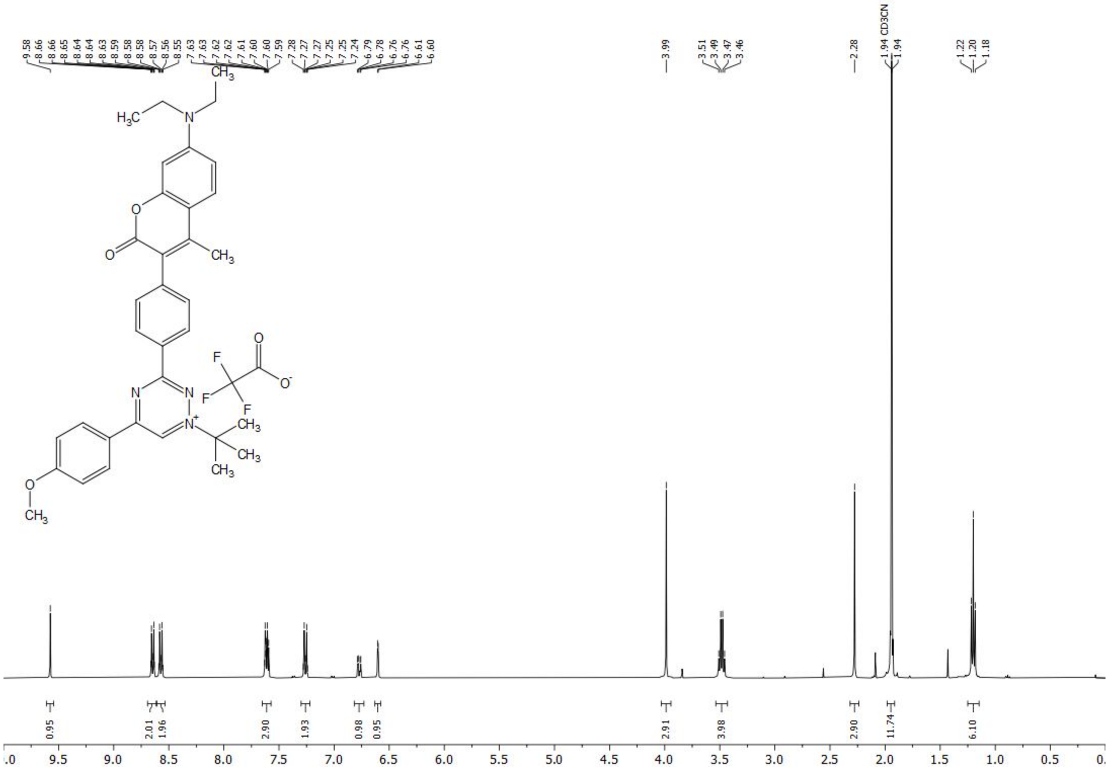

Trz⁺Coum8 ^{19}F NMR (376 MHz, CD_3CN)

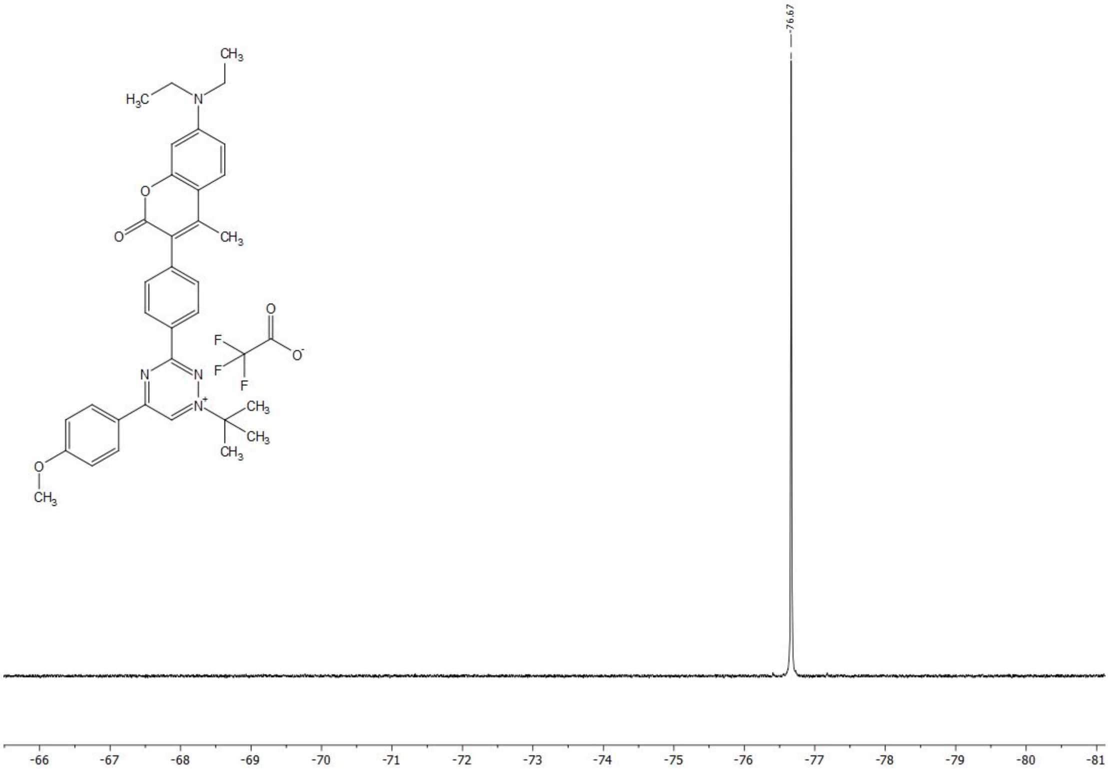

Trz⁺Coum8 ^{13}C NMR (101 MHz, CD_3CN)

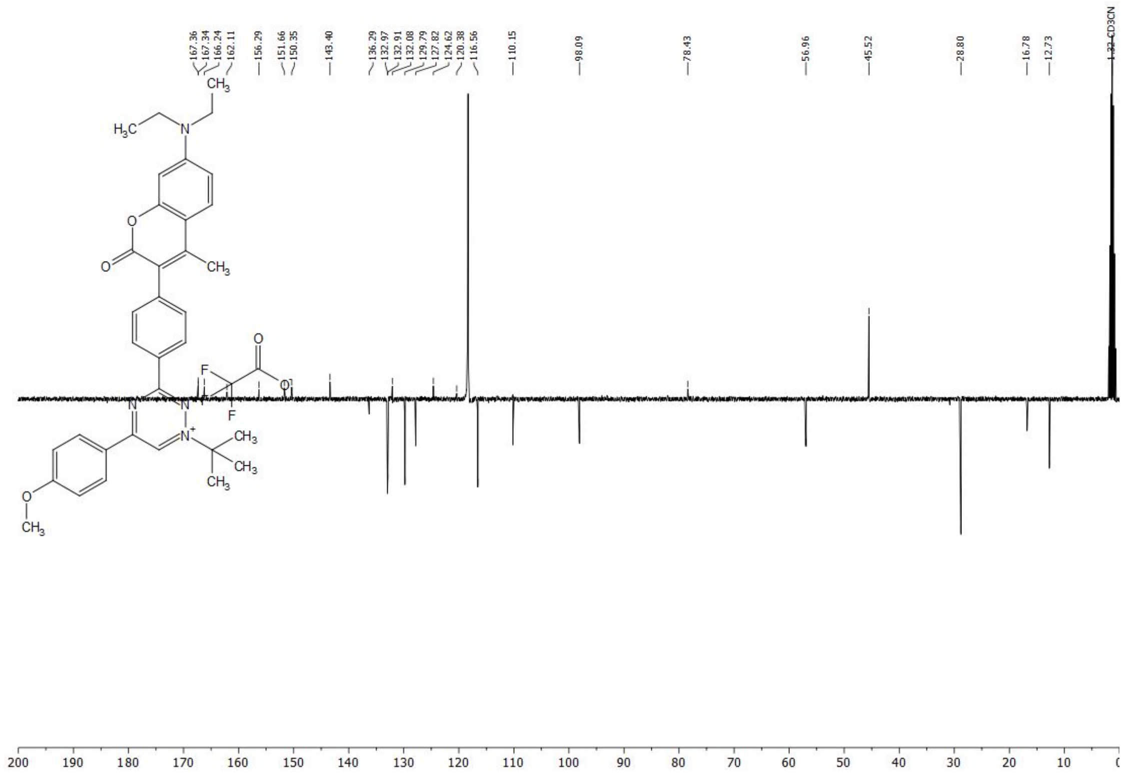

Supplement: Supplementary file 2 — jo3c02454_si_002.pdf [file jo3c02454_si_002.pdf]
